# Charge Relocation Enables a Modular and Diastereoselective Synthesis of *cis*‐Substituted Tetrahydrofurans

**DOI:** 10.1002/anie.202503750

**Published:** 2025-06-04

**Authors:** Zhi‐Jie Niu, Bogdan R. Brutiu, Margaux Riomet, Daniel Kaiser, Nuno Maulide

**Affiliations:** ^1^ Institute of Organic Chemistry University of Vienna Währinger Straße 38 Vienna 1090 Austria

**Keywords:** Charge relocation, Diastereoselectivity, Hantzsch esters, Oxocarbenium ions, Tetrahydrofurans

## Abstract

Diastereoselective construction of organic scaffolds from simple and commercial materials is a powerful strategy in contemporary organic synthesis. Herein, we report a novel disconnection for the direct preparation of tetrahydrofurans (THFs) and corresponding derivatives from acyl chlorides and alkenes using charge relocation. Newly synthesised Hantzsch ester derivatives, functioning both as hydride sources and selectivity modulators, enabled exceptional diastereoselectivity. The wide range of alkenes tolerated is a highlight of the method, enabling the construction of structurally distinct THF‐containing skeletons.

## Graphical abstract

A disconnection model in which acylium ions generated from acyl chlorides are treated with variety of alkenes is reported for the synthesis of 2,5‐*cis*‐disubstituted tetrahydrofurans (THFs) and other THF derivatives. A charge relocation process leads to the formation of oxocarbenium ions, which are reduced to the desired THF structures. The development of sterically encumbered Hantzsch esters was crucial to the success of this highly diastereoselective process.

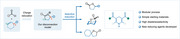

The direct transformation of simple starting materials into complex and biologically significant compounds remains a cornerstone of modern organic synthesis. Methodologies that enable this are highly sought after, particularly in drug discovery and development, where the speed with which structurally diverse libraries of bioactive compounds can be generated directly impacts the identification of new potential therapeutics. One class of compounds that exemplifies this principle is the tetrahydrofuran (THF) ring system,^[^ ^1^, ^2^, ^3^, ^4^, ^5^, ^6^ ^]^ a versatile and prevalent scaffold found in numerous natural products (especially in marine natural products such as lucidumone^[^ ^4^ ^]^ or nonactin^[^ ^1^ ^]^), pharmaceuticals (enadoline^[^ ^5^ ^]^) and agrochemicals (spirodiclofen^[^ ^6^ ^]^) (Scheme 1a), its presence often imparting crucial biological activity.^[^ ^7^, ^8^, ^9^ ^]^

**Scheme 1:**
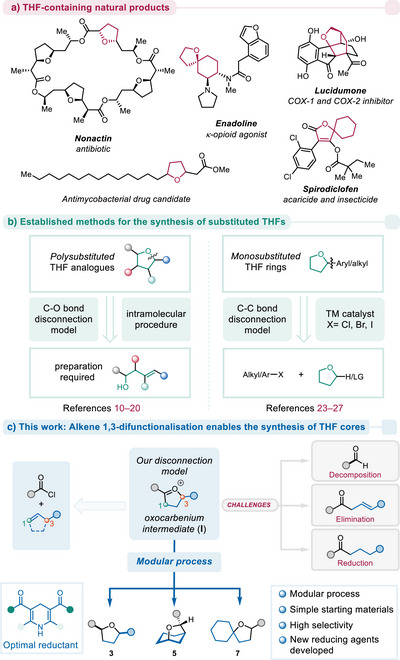
a) Selected examples of THF‐containing natural products, drug candidates and agrochemicals. b) Established disconnection models for the synthesis of THF analogues. c) This work: unexplored alkene 1,3‐difunctionalisation model for the modular preparation of THF‐containing building blocks.

Despite the significance of THF‐containing compounds, traditional synthetic routes to polysubstituted tetrahydrofurans often involve cyclisation strategies (Scheme 1b, left),^[^ ^10^, ^11^, ^12^, ^13^, ^14^, ^15^, ^16^, ^17^, ^18^, ^19^, ^20^ ^]^ typically relying on hydroxy group‐substituted alkenes. Although being effective approaches, the abundance of substitution patterns encountered in naturally occurring THFs almost invariably mandates multistep precursor syntheses. Given the flourishing of transition‐metal catalysed cross‐coupling and C–H bond functionalisation chemistry,^[^ ^21^, ^22^ ^]^ the direct modification of THF skeletons has also been studied in recent years (Scheme 1b, right),^[^ ^23^, ^24^, ^25^, ^26^, ^27^ ^]^ exemplified by Doyle's direct arylation of tetrahydrofuran under blue light irradiation^[^ ^23^ ^]^ and Martin's alkylation of tetrahydrofuran derivatives.^[^ ^24^ ^]^ More recently, Kong developed an enantioselective C–H bond functionalisation approach delivering exclusively monosubstituted THF products.^[^ ^25^ ^]^ In comparison, the diastereoselective preparation of *cis*‐2,5‐disubstituted THF rings remains underexplored.^[^ ^28^, ^29^, ^30^, ^31^ ^]^

Seeking to address this dearth of methods, we envisaged a modular process for the construction of THF building blocks based on our recent discovery of charge relocation‐guided reactivity (Scheme 1c).^[^ ^32^ ^]^ Concretely, we considered that deploying the union of acylium ions and alkenes to form five‐membered cyclic oxocarbenium ion intermediates (**I**) in combination with a stereoselective reduction event might result in the formation of 2,5‐disubstituted tetrahydrofurans. The identification of a suitable reducing agent, allowing high conversions and high levels of diastereoselectivity, promised to be a challenging obstacle for this endeavour. A particularly exciting aspect of this plan a priori was the potential to use a wide range of alkenes to construct structurally distinct types of THF‐containing skeletons. For example, although linear alkenes were expected to result in the ultimate formation of 2,5‐disubstituted THF rings (**3**), cyclic alkenes might deliver oxa‐bridged bicyclic scaffolds (**5**), and spirocyclic ethers (**7**) might be expected upon deployment of vinyl cyclohexane. We, herein, present the culmination of our studies.

A critical aspect of the hypothesis presented in Scheme 1c (left) was the identification of a suitable reductant capable of selectively reducing the intermediate oxocarbenium ion while simultaneously minimising ring‐opening reactions that could lead to ketone (or diol) by‐products. Initial attempts, in which NaBH_3_CN was used as the reductant, led to the detection of the desired THF product in 39% NMR yield and with a diastereomeric ratio (d.r.) of only 1.2:1 (see Supporting Information).^[^ ^33^ ^]^ Further exploration using Et_3_SiH as a reducing agent delivered unsatisfactory results as benzaldehyde was found as the only detectable product, with no trace of the desired THF.^[^ ^33^ ^]^ A more promising result was obtained when using Bu_3_SnH, as the 2,5‐disubstituted THF was obtained in 59% NMR yield, albeit still with low diastereoselectivity (d.r. 1.7:1).^[^ ^33^ ^]^ These unsuccessful results confirmed our suspicion that the determination of conditions allowing for a highly selective reduction event would not be straightforward. A first improvement of the stereoselectivity of the reduction was achieved when the reaction mixture containing oxocarbenium ion **I** was treated with Hantzsch ester (**HEH**) **RA 1**,^[^ ^34^ ^]^ yielding product **3a** in 70% NMR yield and with a d.r. of 5:1, favouring the *cis*‐configured product, as confirmed by NOESY NMR analysis.^[^ ^35^ ^]^ However, further highlighting the challenges inherent to this process (see Scheme 1c), we also observed the formation of β,γ‐unsaturated ketones (19% NMR yield), resulting from oxocarbenium ion **I** having undergone elimination rather than reduction. Pleasingly, a survey of other **HEH** analogues,^[^ ^36^ ^]^ some of which were prepared for the first time in the context of this study, allowed us to suppress by‐product formation.

Thus, under optimised conditions, **3a** was obtained in 70% yield (d.r. 15:1), using an unreported 2,6‐di‐*tert*‐butylphenyl‐substituted Hantzsch ester (**RA 4**) (Scheme 2, top). Offering a favourable compromise between yield and (stereo)selectivity, **RA 4** was selected for the synthesis of the 2,5‐*cis*‐disubstituted THF products.

**Scheme 2:**
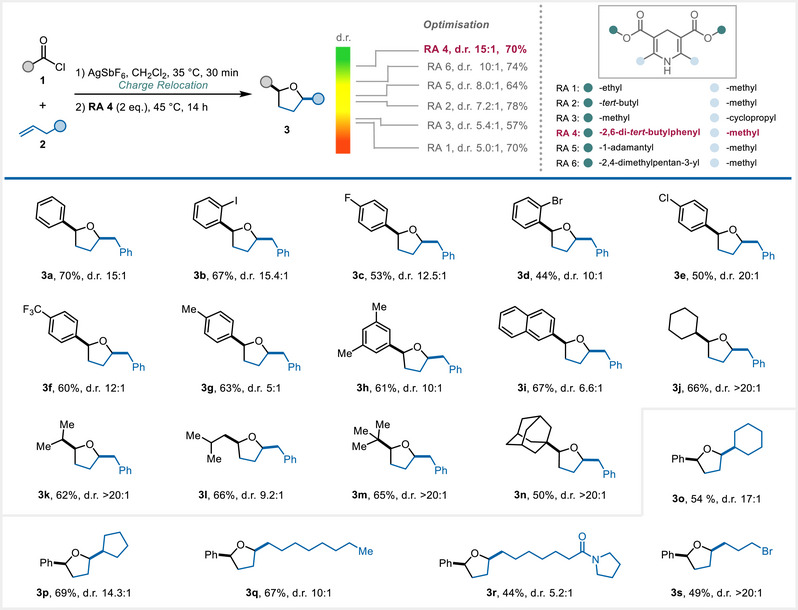
Synthesis of 2,5‐*cis*‐disubstituted THF products. Top: optimisation of the reaction conditions. Bottom: scope of 2,5‐*cis*‐disubstituted THF products. Conditions: alkene **2** (0.20 mmol, 1.00 equiv), acid chloride **1** (0.21 mmol, 1.05 equiv), AgSbF_6_ (0.22 mmol, 1.10 equiv), CH_2_Cl_2_, 35 °C, 30 min, then filtration and addition of **RA 4** (0.4 mmol, 2.00 equiv), 45 °C, 14 h; see Supporting Information (Section 6.1) for the detailed experimental procedure.

To explore the scope of this transformation, 4‐phenylbut‐1‐ene **2a** was initially employed as the standard alkene substrate in combination with different acyl chlorides (Scheme 2). At first, benzoyl chlorides bearing various functional groups, including halo (**3b**–**3e**), trifluoromethyl (**3f**) and methyl groups (**3** **g**, **3** **h**), as well as naphthoyl chloride (**3i**) were surveyed, all of which afforded the desired products with generally high levels of stereoselectivity. Aliphatic acyl chlorides were found to also provide the desired products in good yields and with excellent diastereoselectivity (**3j**–**3n**). We further expanded the scope by exploring various other linear alkenes, which showed compatibility with varying substitution patterns (**3o**–**3r**). Pleasingly, the use of 6‐bromohexene also produced the desired THF product in a yield of 49% and with outstanding selectivity (**3s**, d.r. > 20:1).

After evaluating the scope of this transformation with linear alkenes, we next investigated cyclic olefins, which deliver oxa‐bridged bicyclic products (Scheme 3). To the best of our knowledge, a simple and general procedure for the construction of such oxa‐bridged bicyclic skeletons has not been reported.^[^ ^37^, ^38^, ^39^, ^40^ ^]^ We initially used cyclohexene as the alkene substrate, obtaining **5a** in 66% yield from the reaction with benzoyl acylium ion, followed by reduction with reducing agent **RA 2** at 45 °C. In this particular case, **RA 2** showed excellent diastereoselectivity (d.r. > 20:1).

**Scheme 3:**
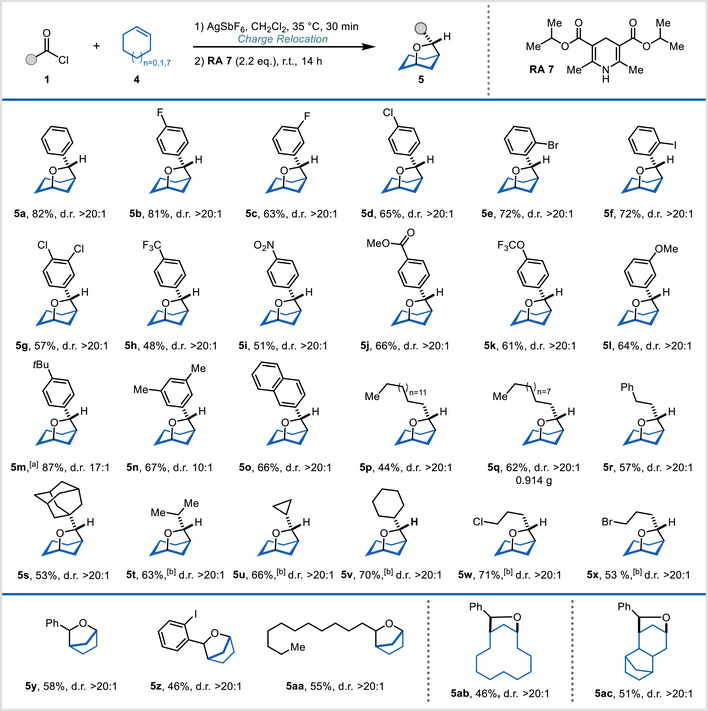
Synthesis of oxa‐bridged bicyclic products. a) Reducing agent **RA 4** was used. b) Due to the low boiling point of products **5t, 5u, 5v, 5w**, and **5x**, yields were determined by NMR analysis of the crude reaction mixture, using mesitylene as an internal standard. c) Conditions: alkene **4** (0.20 mmol, 1.00 equiv), acid chloride **1** (0.24 mmol, 1.20 equiv), AgSbF_6_ (0.24 mmol, 1.20 equiv), CH_2_Cl_2_, 35 °C, 30 min, then filtration and addition of the selected reducing agent (0.44 mmol, 2.20 equiv), r.t., 14 h; see Supporting Information (Section 6.2) for the detailed experimental procedure.

Following slight modification of reaction parameters (see Table S5 for details), we were able to increase the yield of **5a** to 82%, using a small excess of the acylium ion (1.2 equiv) and performing the reduction with reducing agent **RA 7** at ambient temperature, while maintaining an excellent d.r. value of >20:1 (Scheme 3, **5a**).

Our investigation into the utilisation of benzoyl chlorides for the synthesis of oxa‐bridged products demonstrated broad functional group tolerance. Halogen‐substituted benzoyl chlorides consistently afforded excellent selectivity alongside good yields (57% to 81%, **5b**–**5g**) and electron‐deficient congeners, such as benzoyl chlorides substituted with trifluoromethyl, nitro, ester and trifluoromethoxy groups, were well tolerated, allowing the formation of oxa‐bridged bicyclic products in moderate to high yields (**5h**–**5k**, 48%–66%) and >20:1 stereoselectivity throughout.

Similarly, benzoyl chloride derivatives bearing methoxy, *tert*‐butyl, 3,5‐dimethyl substituents delivered the desired products (**5l**–**5n**) in satisfying yields and with good selectivity, as did naphthoyl chloride (**5o**). Aliphatic acyl chlorides were also successfully incorporated, generating the target scaffolds in yields between 44% and 71% (**5p**–**5x**), including a gram‐scale reaction (**5q**, 0.914 g, 62%, d.r. > 20:1).^[^ ^41^ ^]^ Moreover, reactions with cyclopentene afforded the target products in good yields with consistently high stereoselectivity (>20:1). Additionally, the suitability of macrocyclic and fused alkenes was explored, yielding the oxa‐bridged products **5ab** and **5ac** in 46% and 51% yield, respectively, maintaining the high diastereoselectivity in both cases (d.r. > 20:1).

We continued our investigations using vinyl cyclohexane **6a** which would generate a spirocyclic ether framework. This was borne in practice, with ethers **7a**–**7h** being accessible following reduction with Hantzsch ester **RA 7** (Scheme 4a). Investigation of the scope demonstrated that both benzoyl chlorides and aliphatic acyl chlorides allowed the formation of the anticipated spirocyclic products, with yields ranging from 42% to 81% (**7a**–**7h**). Lauroyl chloride, in particular, showed a good result when scaled up to 1 mmol (**7f**, 81%).

**Scheme 4:**
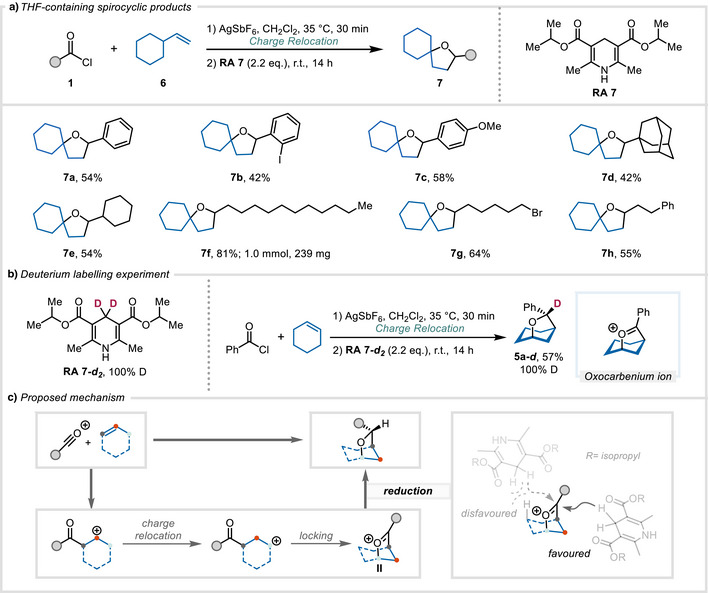
a) Synthesis of spirocyclic THF products. Conditions: alkene **6** (0.20 mmol, 1.00 equiv), acid chloride **1** (0.24 mmol, 1.20 equiv), AgSbF_6_ (0.24 mmol, 1.20 equiv), CH_2_Cl_2_, 35 °C, 30 min, then filtration and addition of **RA 7** (0.44 mmol, 2.20 equiv), r.t., 14 h; see Supporting Information (Section 6.3) for the detailed experimental procedure. b) Deuterium labelling experiment. c) Mechanistic proposal involving charge relocation and a rationale for diastereoselectivity.

When a deuterated version of the reducing agent (**RA 7‐d_2_**) was used, the formation of a deuterated oxa‐bridged bicyclic product was observed (**5a‐*d***, 57% yield, Scheme 4b). This result, in combination with knowledge gained during previous investigations,^[^ ^32^ ^]^ allows us to propose a mechanism for the transformation shown (Scheme 4c). After halide abstraction and formation of an acylium ion, Friedel–Crafts‐type addition of the alkene leads to the formation of a cation β to the carbonyl (likely destabilised as a result of inductive effects). Following the principles of charge relocation,^[^ ^32^ ^]^ the equilibration to the corresponding γ‐cation—through 1,2‐hydride shift^[^ ^42^ ^]^ and capture of the resulting cation by the carbonyl—allows a locking event that forms the pivotal oxocarbenium species **II**. As shown by the result presented in Scheme 4b, delivery of a hydride (or deuteride) from the employed Hantzsch ester ultimately furnishes the desired tetrahydrofuran. The inset in Scheme 4c provides a rationale for the stereoselectivity observed in this step, in which the increased steric demand of the employed reductants, bearing isopropyl (or 2,6‐di‐*tert*‐butylphenyl) substitution, impedes hydride delivery from the more encumbered side of the bridged bicyclic system. Notably, this process places the substituent (e.g., the phenyl ring of **5a‐*d***) in the more disfavoured orientation.

In summary, we have developed a modular approach for the selective construction of tetrahydrofuran‐containing scaffolds showing a variety of architectures. In this procedure, new Hantzsch esters were synthesised to enable the preparation of THF skeletons with excellent diastereoselectivity. The wide range of alkene substrates tolerated facilitated the synthesis of 2,5‐*cis*‐disubstituted tetrahydrofurans, oxa‐bridged bicyclic compounds and spirocyclic ethers by a single common strategy.

## Supporting Information

The authors have cited additional references within the Supporting Information.^[^ ^43^, ^44^, ^45^, ^46^, ^47^ ^]^

## Conflict of Interests

The authors declare no conflict of interest.

## Supporting information



Supporting information

## Data Availability

The data that support the findings of this study are available in the Supporting Information of this article.

## References

[1] A. Lorente , J. Lamariano‐Merketegi , F. Albericio , M. Álvarez , Chem. Rev. 2013, 113, 4567–4610. 23506053 10.1021/cr3004778

[2] L. Fernández‐Peña , C. Díez‐Poza , P. González‐Andrés , A. Barbero , Mar. Drugs. 2022, 20, 120. 36286465 10.3390/md20100642 PMC9605582

[3] N. A. Petasis , M. A. Patane , Tetrahedron 1992, 48, 5757–5821.

[4] W. Zhang , L. Li , C. Li , Chem. Soc. Rev. 2021, 50, 9430–9442. 34286715 10.1039/d0cs01471k

[5] S. L. Walsh , E. C. Strain , M. E. Abreu , G. E. Bigelow , Psychopharmacology 2001, 157, 151–162. 11594439 10.1007/s002130100788

[6] L. D. Maeyer , R. Geerinck , Commun. Agric. Appl. Biol. Sci. 2009, 74, 225–232. 20218531

[7] D. C. Hopp , F. Q. Alali , Z.‐M. Gu , J. L. McLaughlin , Bioorg. Med. Chem. 1998, 6, 569–575. 9629470 10.1016/s0968-0896(98)00018-2

[8] Y. Chen , J. Chen , X. Li , J. Nat. Prod. 2011, 74, 2477–2481. 22011319 10.1021/np200708q

[9] A. Bermejo , B. Figadère , M.‐C. Zafra‐Polo , I. Barrachina , E. Estornell , D. Cortes , Nat. Prod. Rep. 2005, 22, 269–303. 15806200 10.1039/b500186m

[10] K. Asano , S. Matsubara , J. Am. Chem. Soc. 2011, 133, 16711–16713. 21942273 10.1021/ja207322d

[11] H. Qian , X. Han , R. A. Widenhoefer , J. Am. Chem. Soc. 2004, 126, 9536–9537. 15291546 10.1021/ja0477773

[12] J. P. Wolfe , M. A. Rossi , J. Am. Chem. Soc. 2004, 126, 1620–1621. 14871078 10.1021/ja0394838

[13] Z. Zhang , C. Liu , R. E. Kinder , X. Han , H. Qian , R. A. Widenhoefer , J. Am. Chem. Soc. 2006, 128, 9066–9073. 16834380 10.1021/ja062045r

[14] J. P. Wolfe , M. V. Hay , Tetrahedron 2007, 63, 261–290. 18180807 10.1016/j.tet.2006.08.105 PMC1826827

[15] E. J. Tollefson , L. E. Hanna , E. R. Jarvo , Acc. Chem. Res. 2015, 48, 2344–2353. 26197033 10.1021/acs.accounts.5b00223 PMC4956245

[16] T. D. Aicher , K. R. Buszek , F. G. Fang , C. J. Forsyth , S. H. Jung , Y. Kishi , M. C. Matelich , P. M. Scola , D. M. Spero , S. K. Yoon , J. Am. Chem. Soc. 1992, 114, 3162–3164.

[17] K. Namba , H.‐S. Jun , Y. Kishi , J. Am. Chem. Soc. 2004, 126, 7770–7771. 15212512 10.1021/ja047826b

[18] J. H. Lee , Y. Kishi , J. Am. Chem. Soc. 2016, 138, 7178–7186. 27177162 10.1021/jacs.6b03897

[19] K. Namba , Y. Kishi , J. Am. Chem. Soc. 2005, 127, 15382–15383. 16262397 10.1021/ja055966v

[20] Y.‐R. Yang , D.‐S. Kim , Y. Kishi , Org. Lett. 2009, 11, 4516–4519. 19754145 10.1021/ol9016589 PMC2759418

[21] W. Shi , C. Liu , A. Lei , Chem. Soc. Rev. 2011, 40, 2761–2776. 21283847 10.1039/c0cs00125b

[22] E. L. Lucas , N. Y. S. Lam , Z. Zhuang , H. S. S. Chan , D. A. Strassfeld , J.‐Q. Yu , Acc. Chem. Res. 2022, 55, 537–550. 35076221 10.1021/acs.accounts.1c00672 PMC9129890

[23] B. J. Shields , A. G. Doyle , J. Am. Chem. Soc. 2016, 138, 12719–12722. 27653738 10.1021/jacs.6b08397 PMC5215658

[24] Y. Shen , Y. Gu , R. Martin , J. Am. Chem. Soc. 2018, 140, 12200–12209. 30184423 10.1021/jacs.8b07405

[25] S. Xu , Y. Ping , W. Li , H. Guo , Y. Su , Z. Li , M. Wang , W. Kong , J. Am. Chem. Soc. 2023, 145, 5231–5241. 36812098 10.1021/jacs.2c12481

[26] A. Noble , S. J. McCarver , D. W. C. MacMillan , J. Am. Chem. Soc. 2015, 137, 624–627. 25521443 10.1021/ja511913h PMC4308738

[27] Y. Dong , P. Ji , Y. Zhang , C. Wang , X. Meng , W. Wang , Org. Lett. 2020, 22, 9562–9567. 33300807 10.1021/acs.orglett.0c03624 PMC7936573

[28] S. J. Gharpure , R. S. Chavan , A. V. Ardhapure , Adv. Synth. Catal. 2022, 364, 3094–3098.

[29] D. A. Mundal , K. E. Lutz , R. J. Thomson , J. Am. Chem. Soc. 2012, 134, 5782–5785. 22452672 10.1021/ja301489n

[30] S. J. Gharpure , D. S. Vishwakarma , S. K. Nanda , Org. Lett. 2017, 19, 6534–6537. 29166034 10.1021/acs.orglett.7b03241

[31] Y. Landrain , G. Evano , Org. Lett. 2023, 25, 3898–3903. 37220014 10.1021/acs.orglett.3c01265

[32] B. R. Brutiu , G. Iannelli , M. Riomet , D. Kaiser , N. Maulide , Nature 2024, 626, 92–97. 38297174 10.1038/s41586-023-06938-0 PMC10830407

[33] See Table S1 in Supporting Information for details.

[34] A. Hantzsch , Ber. Dtsch. Chem. Ges. 1881, 14, 1637–1638.

[35] See Section 5 (stereochemical analysis) of the Supporting Information for details.

[36] P. Wang , J. Chen , W. Xiao , Org. Biomol. Chem. 2019, 17, 6936–6951. 31268084 10.1039/c9ob01289c

[37] S. Ma , Z. Li , P. Yu , H. Shi , H. Yang , J. Yi , Z. Zhang , X. Duan , X. Xie , X. She , Org. Lett. 2022, 24, 5541–5545. 35894551 10.1021/acs.orglett.2c02023

[38] G. Huang , C. Kouklovsky , A. de la Torre , J. Am. Chem. Soc. 2022, 144, 17803–17807. 36150082 10.1021/jacs.2c08760

[39] Y. Kawamoto , N. Noguchi , T. Kobayashi , H. Ito , Angew. Chem., Int. Ed. 2023, 62, e202304132. 10.1002/anie.202304132 37041112

[40] X.‐Z. Liao , R. Wang , X. Wang , G. Li , Nat. Commun. 2024, 15, 2647. 38531853 10.1038/s41467-024-46896-3 PMC10966040

[41] For failed examples, see Supporting Information, Scheme S4.

[42] L. E. Overman , A. L. Tomasi , J. Am. Chem. Soc. 1998, 120, 4039–4040.

[43] A. F. G. Maier , S. Tussing , T. Schneider , U. Flörke , Z.‐W. Qu , S. Grimme , J. Paradies , Angew. Chem. Int. Ed. 2016, 55, 12219–12223. 10.1002/anie.201606426 27594431

[44] S. Qiu , H. Guo , P. Xu , Org. Lett. 2024, 26, 6730–6735. 39078309 10.1021/acs.orglett.4c02380

[45] D. P. Hari , G. Pisella , M. D. Wodrich , A. V. Tsymbal , F. F. Tirani , R. Scopelliti , J. Waser , Angew. Chem. Int. Ed. 2021, 60, 5475–5481. 10.1002/anie.202012299 33216417

[46] D. P. Hari , J. Waser , J. Am. Chem. Soc. 2017, 139, 8420–8423. 28621137 10.1021/jacs.7b04756

[47] J. Zhang , Y. Li , R. Y. Xu , Y. Y. Chen , Angew. Chem. Int. Ed. 2017, 56, 12619–12623. 10.1002/anie.201707171 28809077

