# Supplementary material for: Charge Relocation Enables a Modular and Diastereoselective Synthesis of cis‐Substituted Tetrahydrofurans
Source: Angew Chem Int Ed Engl. 2025 Jun 4;64(29):e202503750. doi: 10.1002/anie.202503750 (PMC12258665; doi:10.1002/anie.202503750)
Supplement: Supplementary file 1 — Supporting information [file ANIE-64-e202503750-s001.pdf]

Charge Relocation Enables a Modular and Diastereoselective Synthesis of *cis*-Substituted Tetrahydrofurans

Zhi-Jie Niu,[a] Bogdan R. Brutiu,[a] Margaux Riomet,[a] Daniel Kaiser,[a] and Nuno Maulide*[a]

[a] Institute of Organic Chemistry, University of Vienna, Währinger Straße 38, 1090 Vienna (Austria)

E-Mail: nuno.maulide@univie.ac.at, Homepage: http://maulide.univie.ac.at

Content

1. General information 2
2. Overview of scope 3
3. Optimisation 6
4. Preparation of reducing agents 9
   4.1: Di-*tert*-butyl 2,6-dimethyl-1,4-dihydropyridine-3,5-dicarboxylate (RA 2). 9
   4.2: Dimethyl 2,6-dicyclopropyl-1,4-dihydropyridine-3,5-dicarboxylate (RA 3). 9
   4.3: Bis(2,6-di-*tert*-butylphenyl) 2,6-dimethyl-1,4-dihydropyridine-3,5-dicarboxylate (RA 4). 10
   4.4: Di(adamantan-1-yl) 2,6-dimethyl-1,4-dihydropyridine-3,5-dicarboxylate (RA 5). 11
   4.5: Bis(2,4-dimethylpentan-3-yl) 2,6-dimethyl-1,4-dihydropyridine-3,5-dicarboxylate (RA 6). 12
   4.6: Bis(2,6-di-*tert*-butylphenyl) 2,6-dimethyl-1,4-dihydropyridine-3,5-dicarboxylate (RA 7). 13
5. Stereochemical analysis 14
   5.1: NOESY spectra of product 3a (*cis*-2-Benzyl-5-phenyltetrahydrofuran). 14
   5.2: NOESY spectra of 5a (7-Phenyl-6-oxabicyclo[3.2.1]octane). 15
6 Experimental procedure and characterisation 16
   6.1 Synthesis of cis-2,5-disubstituted THF products (3a-3s). 16
   6.2: Synthesis of oxo-bridged bicyclic products (5a–5ac). 36
   6.3: Synthesis of spirocyclic THF products (7a–7h) 66
7. Isotope labelling experiments. 75
   7.1: Synthesis of deuterated reducing agent RA 7-d_2. 75
   7.2: Deuterium labelling experiments 77
8. NMR Spectra 79
9. References 143

1. General information

Unless otherwise stated, all glassware was flame-dried before use and all reactions were performed under an atmosphere of argon. All solvents were distilled from appropriate drying agents prior to use or, if purchased in anhydrous form, used as received. All reagents were used as received from commercial suppliers, unless otherwise stated. Reaction progress was monitored by thin layer chromatography (TLC) performed on aluminium plates coated with silica gel F254 with 0.2 mm thickness. Chromatograms were visualised by fluorescence quenching with UV light at 254 nm, or by staining using either potassium permanganate or phosphomolybdic acid. Flash column chromatography was performed using silica gel 60 (230-400 mesh, Merck and co.) or pre-packed columns. Neat infrared spectra were recorded using a Perkin-Elmer Spectrum 100 FT-IR spectrometer. Wavenumbers (vmax) are reported in cm^{-1}. HR-ESI-MS spectra (*m/z* 50-1900) were obtained in a maXis UHR ESI-Qq-TOF mass spectrometer (Bruker Daltonics, Bremen, Germany) in the positive and/or negative ion mode by direct infusion. The sum formulas of the detected ions were determined using Bruker Compass Data Analysis 4.1 based on the mass accuracy ($\Delta m/z \leq 5$ ppm) and isotopic pattern matching (Smart Formula algorithm). All ^{1}H NMR, ^{13}C DEPTQ-135 NMR, ^{13}C CPD NMR and ^{19}F NMR spectra were recorded using a Bruker AV-400, AV-500, AV-600 or AV-700 spectrometer at 300 K. Chemical shifts are given in parts per million (ppm, δ), referenced to the solvent peak of $CDCl_3$, defined at δ = 7.26 ppm (^{1}H NMR) and δ = 77.16 (^{13}C NMR). Coupling constants are quoted in Hz (*J*). ^{1}H NMR splitting patterns are designated as singlet (s), doublet (d), triplet (t), quartet (q) or heptet (hept) as they appear in the spectrum. If the appearance of a signal differs from the expected splitting pattern, the observed pattern is designated as apparent (app). Splitting patterns that could not be interpreted or easily visualised are designated as multiplet (m) or broad (br).

2. Overview of scope

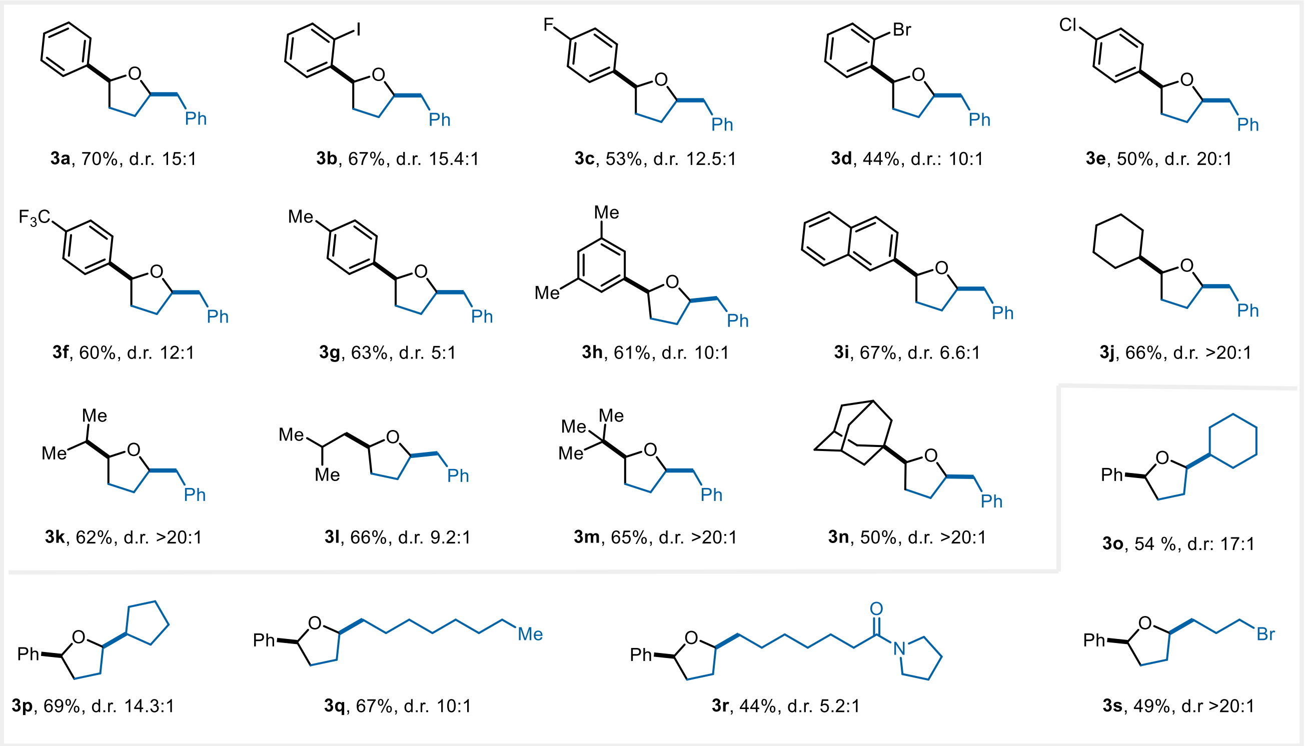

Scheme S1: 2,5-Disubstituted THF products **3a**–**3s**. Conditions: Alkene **2** (0.20 mmol, 1.00 equiv.), acid chloride **1** (0.21 mmol, 1.05 equiv.), $AgSbF_6$ (0.22 mmol, 1.10 equiv.), CH_2Cl_2, 35 °C, 30 min. Then filtration and addition of **RA 4** (0.4 mmol, 2.00 equiv.), 45 °C, 14 h; see section 6.1 for the detailed experimental procedure.

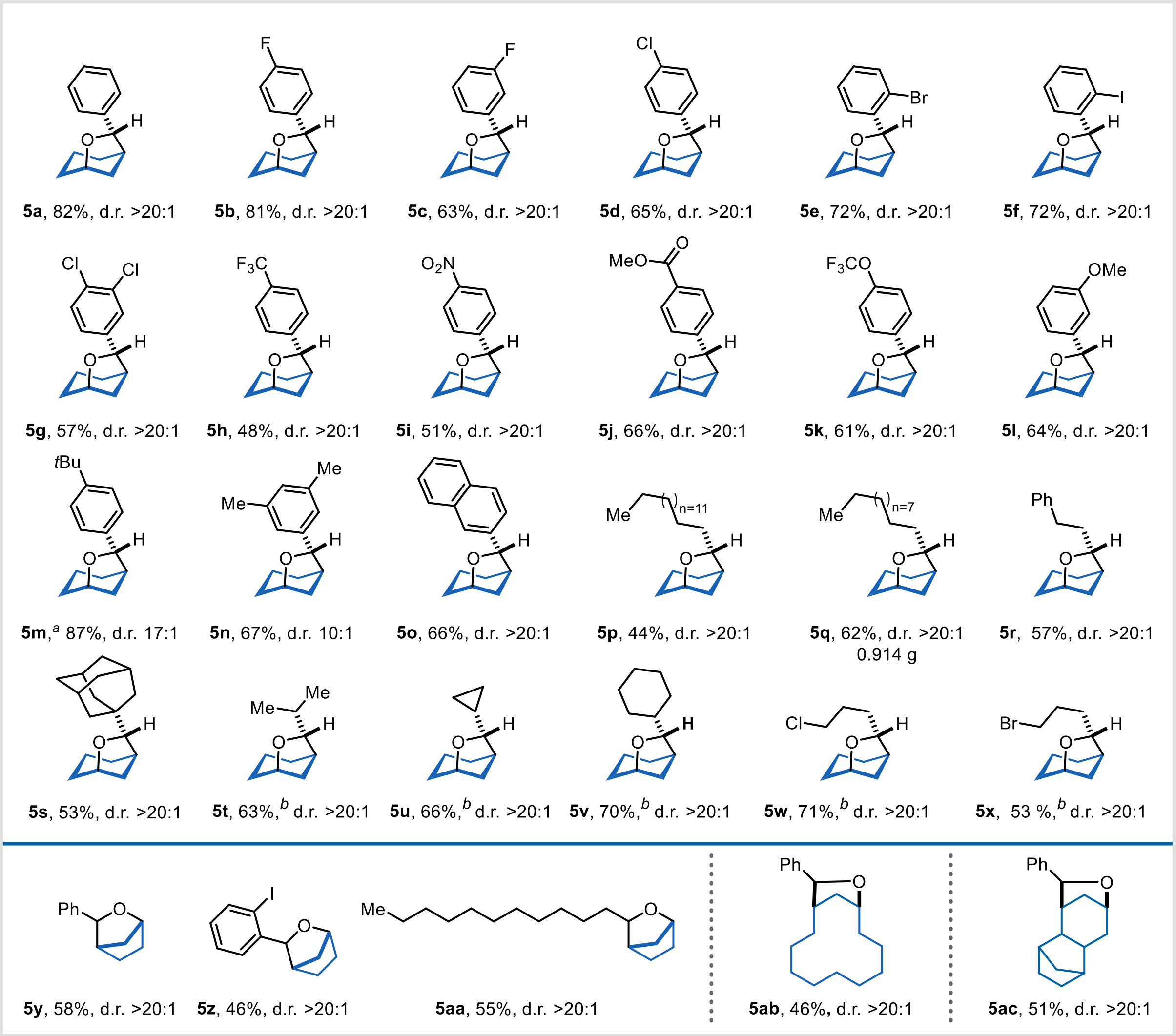

Scheme S2. Synthesis of oxa-bridged bicyclic products (**5a**–**5ac**). a Reducing agent **RA 4** was used; b) Due to the low boiling point of products **5t, 5u, 5v, 5w, and 5x**, yields were determined by NMR analysis of the crude reaction mixture, using mesitylene as an internal standard. c) Conditions: Alkene **4** (0.20 mmol, 1.00 equiv.), acid chloride **1** (0.24 mmol, 1.20 equiv.), $AgSbF_6$ (0.24 mmol, 1.20 equiv.), CH_2Cl_2, 35 °C, 30 min. Then filtration and addition of the selected reducing agent (0.44 mmol, 2.20 equiv.), 22–25 °C, 14 h; see section 6.2 for the detailed experimental procedure.

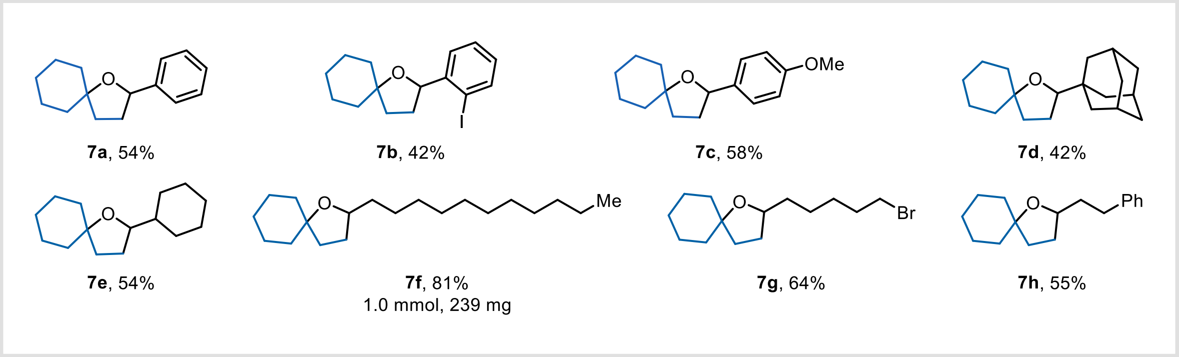

Scheme S3: Spirocyclic THF products (**7a**–**7h**). Conditions: Alkene **6** (0.20 mmol, 1.00 equiv.), acid chloride **1** (0.24 mmol, 1.20 equiv.), $AgSbF_6$ (0.24 mmol, 1.20 equiv.), CH_2Cl_2, 35 °C, 30 min. Then filtration and addition of **RA 7** (0.44 mmol, 2.20 equiv.), 20–25 °C, 14 h; see section 6.3 for the detailed experimental procedure.

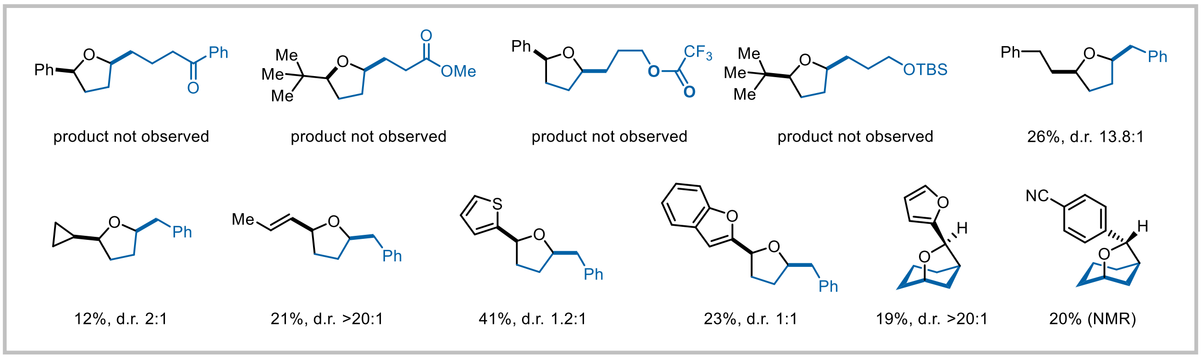

Scheme S4: Failed and low-yielding examples.

3. Optimisation

Table S1: Examination of different reductants.

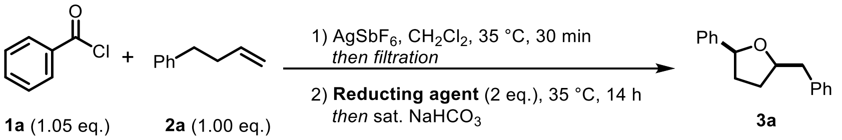

Entry	Reductant	Yield	d.r. (*cis:trans*)[a]
1	$NaBH_3CN$	39 %	1.2:1
2	Et_3SiH	trace	n/a
3	Bu_3SnH	59 %	1.7:1
4	HEH (**RA 1**)	70 %	5:1
5	**RA 2**	70 %	7.2:1

All reactions were carried out on a 0.20 mmol scale. Conditions: Alkene **2a** (0.20 mmol, 1.00 equiv.), acid chloride **1a** (0.21 mmol, 1.05 equiv.), $AgSbF_6$ (0.22 mmol, 1.10 equiv.), CH_2Cl_2, 35 °C, 30 min. Then filtration and addition of reducing agent (0.40 mmol, 2.00 equiv.), 35 °C, 14 h.
a) The *cis*-configured isomer was identified as the major product; stereochemical analysis see section 5.1.

Table S2: Optimisation of solvents, temperature, and stoichiometry of the substrates (with **RA 2**).

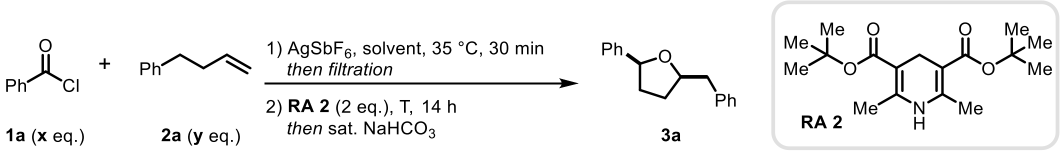

Entry	**1a** (eq.)	**2a** (eq.)	Temperature	Solvent	Yield	d.r. (*cis:trans*)
1	1.00	1.20	35 °C	CH_2Cl_2	60%	7.2:1
2	1.00	1.50	35 °C	CH_2Cl_2	60%	7.7:1
3	1.05	1.00	35 °C	CH_2Cl_2	70%	7.2:1
4	1.05	1.00	40 °C	CH_2Cl_2	78%	7.4:1
5	1.05	1.00	45 °C	CH_2Cl_2	64%	5.4:1
6	1.05	1.00	40 °C	1,2-DCE	58%	3:1
7	1.05	1.00	40 °C	$CHCl_3$	42%	6.6:1

All reactions were carried out on a 0.20 mmol scale. Conditions: Alkene **2a**, acid chloride **1a**, $AgSbF_6$ (0.22 mmol, 1.10 equiv.), CH_2Cl_2, 35 °C, 30 min. Then filtration and addition of **RA 2** (0.40 mmol, 2.00 equiv.), T, 14 h.

Table S3: Optimisation of the stoichiometry of the reducing agent (with **RA 2**).

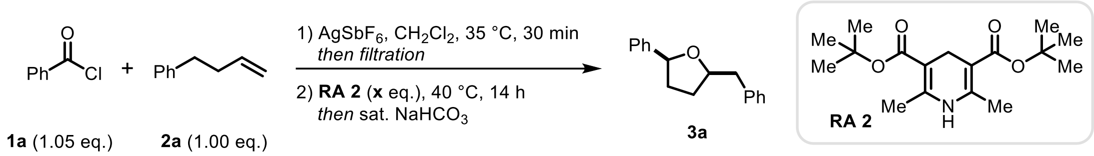

Entry	**RA 2** (eq.)	Yield	d.r. (*cis:trans*)
1	1.50	45%	7.8:1
2	2.50	74%	7.6:1
3	3.00	75%	7.8:1
4	2.00	78%	7.4:1

All reactions were carried out on a 0.20 mmol scale. Conditions: Alkene **2a** (0.20 mmol, 1.00 equiv.), acid chloride **1a** (0.21 mmol, 1.05 equiv.), $AgSbF_6$ (0.22 mmol, 1.10 equiv.), CH_2Cl_2, 35 °C, 30 min. Then filtration and addition of **RA 2** (**x** equiv.), 40 °C, 14 h.

Table S4: Examination of selectivity with Hantzsch ester derivatives.

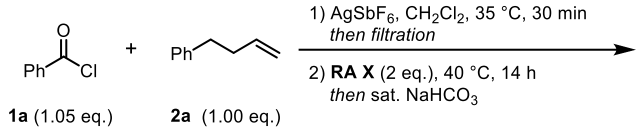

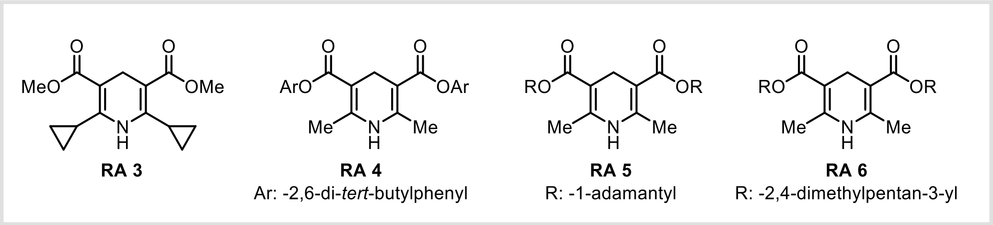

Entry	Reducing agents	Yield	d.r (*cis:trans*)
1	**RA 3**	57%	5.4:1
2	**RA 6**	74%	10:1
3	**RA 5**	64%	8:1
4	**RA 4**	65%	15:1
5[a]	**RA 4**	70%	15:1
6[b]	**RA 4**	70%	14:1

All reactions were carried out on a 0.20 mmol scale. Conditions: Alkene **2a** (0.20 mmol, 1.00 equiv.), acid chloride **1a** (0.21 mmol, 1.05 equiv.), $AgSbF_6$ (0.22 mmol, 1.10 equiv.), CH_2Cl_2, 35 °C, 30 min. Then filtration and addition of **RA X** (0.40 mmol, 2.00 equiv.), 40 °C, 14 h. a) Reduction conducted at 45 °C. b) Reduction conducted at 50 °C.

Table S5: Optimisation of *oxo*-bridged bicyclic product.

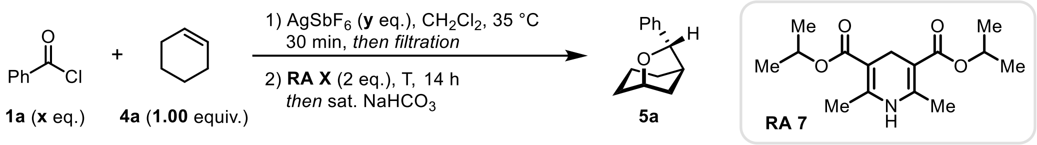

Entry	**1a** (eq.)	$AgSbF_6$ (eq.)	Temp.	Reductant (eq.)	Yield[a]	d.r.
1	1.05	1.1	45 °C	**RA 4**	50%	7.4:1
2	1.05	1.1	45 °C	**RA 2**	51%	>20:1
3	1.05	1.1	45 °C	**RA 2**	45%	4.5:1
4[b]	1.2	1.2	45 °C	**RA 2**	66%	>20:1
5	1.5	1.5	45 °C	**RA 2**	58%	>20:1
6	1.2	1.2	23 °C	**RA 2**	67%	>20:1
7	1.2	1.2	23 °C	**RA 7**	70%	>20:1
8	1.2	1.2	23 °C	**RA 7**[c]	82%	>20:1

All reactions were carried out on a 0.20 mmol scale. Conditions: Alkene **2** (0.20 mmol, 1.00 equiv.), acid chloride **1** (**x** equiv.), $AgSbF_6$ (**y** equiv.), CH_2Cl_2, 35 °C, 30 min. Then filtration and addition of **RA X** (0.40 mmol, 2.00 equiv.), T °C, 14 h. a) Isolated yields. b) Without filtration. c) 2.20 equiv. of **RA 7** were used.

4. Preparation of reducing agents

4.1: Di-*tert*-butyl 2,6-dimethyl-1,4-dihydropyridine-3,5-dicarboxylate (**RA 2**).

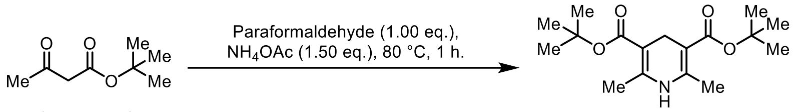

RA 2

A Schlenk flask was charged with paraformaldehyde (20.0 mmol, 606 mg, 1.00 equiv.), ammonium acetate (30.0 mmol, 2.31 g, 1.50 equiv.), *tert*-butyl 3-oxobutanoate (40.0 mmol, 6.32 g, 2.00 equiv.) and subsequently flushed with argon three times. The neat reaction mixture was then heated to 80 °C and stirred at that temperature for 1 h, after which ice-cold water (50mL) was added to the reaction mixture, causing precipitation of the product. The crude product was filtered and the precipitate was recrystallised from methanol yielding a light-yellow solid product (65%, 4.02 g) (**RA 2**). NMR data is consistent with the previous report.[36]

^{1}H NMR (400 MHz, $CDCl_3$): δ 5.05 (s, 1H), 3.17 (s, 2H), 2.14 (s, 6H), 1.47 (s, 18H) ppm.

4.2: Dimethyl 2,6-dicyclopropyl-1,4-dihydropyridine-3,5-dicarboxylate (**RA 3**).

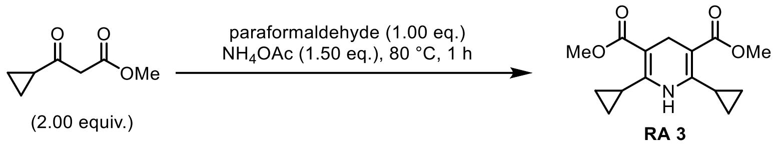

RA 3

A Schlenk flask was charged with paraformaldehyde (5.0 mmol, 151 mg, 1.00 equiv.), ammonium acetate (7.5 mmol, 0.57 g, 1.50 equiv.), methyl 3-cyclopropyl-3-oxopropanoate (10.0 mmol, 1.42 g, 2.00 equiv.) and flushed with argon over three times. The neat reaction mixture was then heated to 80 °C and stirred at that temperature for 1 h, after which ice-cold water (10 mL) was added to the reaction mixture, causing precipitation of the product. The precipitate was recrystallised from methanol yielding a light-yellow solid (**RA 3**, 66%, 0.9 g). NMR data agreed with the previous report.[44]

^{1}H NMR (400 MHz, $CDCl_3$): δ 4.92 (s, 1H), 3.67 (s, 6H), 3.26 (s, 2H), 2.65 (tt, *J* = 8.7, 5.7 Hz, 2H), 0.92 – 0.82 (m, 4H), 0.55 – 0.48 (m, 4H) ppm.

^{13}C NMR (101 MHz, $CDCl_3$): δ 168.1 (2C), 147.9 (2C), 100.3 (2C), 51.0 (2C), 25.6, 12.7 (2C), 5.9 (4C) ppm.

HRMS (ESI$^+$) exact mass calculated for $[C_{15}H_{19}NNaO_4]^+$ ($[M+Na]^+$) required *m/z* 300.1206, found *m/z* 300.1204.

4.3: Bis(2,6-di-*tert*-butylphenyl) 2,6-dimethyl-1,4-dihydropyridine-3,5-dicarboxylate **(RA 4).**

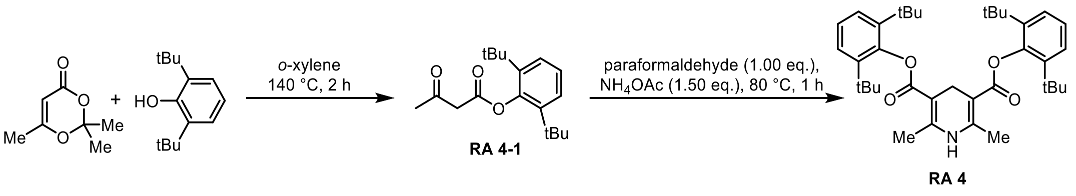

In a round-bottom flask under argon, a mixture of 2,6-di-*tert*-butylphenol (10.32 g, 50.0 mmol, 1.00 equiv.), 2,2,6-trimethyl-4*H*-1,3-dioxin-4-one (7.46 g, 50.0 mmol, 1.00 equiv.) and xylene (15 mL) was stirred at 140 °C for 2 h. After cooling to room temperature, the xylene was evaporated. Purification by flash column chromatography using ethyl acetate:heptane (10:1 to 3:1) as the eluent afforded 2,6-di-*tert*-butylphenyl 3-oxobutanoate (**RA 4-1**) as a colourless oil (84%, 12.1 g). NMR data agreed with the previous report.[44]

^{1}H NMR (400 MHz, CDCl$_3$): δ 12.13 (dt, *J* = 5.7, 2.4 Hz, 0.5H, *OH of enol form*), 7.35 (m, 2H, *ArH of enol and keto form*), 7.17 (m, 1H, *ArH of enol and keto form*), 5.42 – 5.31 (m, 0.5 H, *vinyl H of enol form*), 3.76 (d, *J* = 2.5 Hz, 1H, CH_2 *of keto form*), 2.41 (d, *J* = 1.8 Hz, 1H, CH_3 *of enol form*), 2.11 – 2.08 (m, 1.5H, CH_3 *of keto form*), 1.38 (app dd, *J* = 4.5, 1.6 Hz, 18H, *t*Bu *of enol and keto form*).

A Schlenk flask was charged with paraformaldehyde (10.0 mmol, 303 mg, 1.00 equiv.), ammonium acetate (15.0 mmol, 1.15 g, 1.50 equiv.), 2,6-di-*tert*-butylphenol (20.0 mmol, 5.80 g, 2.00 equiv.) and flushed with argon over three times. The neat reaction mixture was then heated to 80 °C and stirred at that temperature for 1 h, after which ice-cold water (30 mL) was added to the reaction mixture, causing precipitation of the product. The residue was recrystallised from methanol yielding bis(2,6-di-*tert*-butylphenyl) 2,6-dimethyl-1,4-dihydropyridine-3,5-dicarboxylate (**RA 4**) as a yellow solid (73%, 4.18 g).

^{1}H NMR (400 MHz, CDCl$_3$): δ 7.32 (d, *J* = 7.9 Hz, 4H), 7.14 – 7.08 (m, 2H), 5.45 (s, 1H), 4.04 (s, 2H), 2.26 (s, 6H), 1.35 (s, 36H) ppm.

^{13}C NMR (101 MHz, CDCl$_3$): δ 168.0 (2C), 148.4 (4C), 147.6 (2C), 142.8 (2C), 126.1 (2C), 125.3 (4C), 99.6 (2C), 35.4 (4C), 31.7 (14C), 25.5, 19.4 (2C) ppm.

HRMS (ESI$^+$): exact mass calculated for $[C_{37}H_{52}NO]^+$ ($[M+H]^+$) required *m/z* 574.3891, found *m/z* 574.3884.

IR (neat) ν_{max}: 3353, 2930, 1737, 1698, 1493, 1363, 1226, 1178, 991 cm^{-1}.

4.4: Di(adamantan-1-yl) 2,6-dimethyl-1,4-dihydropyridine-3,5-dicarboxylate **(RA 5).**

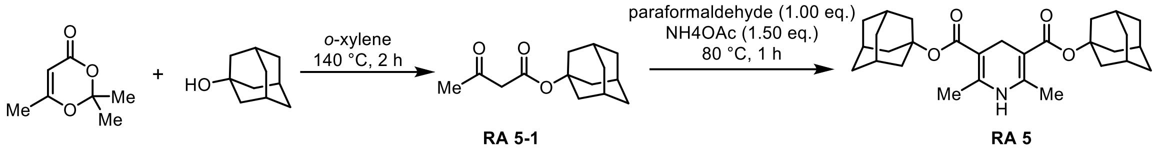

A mixture of 1-adamantanol (3.81 g, 25.0 mmol, 1.00 equiv.), 2,2,6-trimethyl-4*H*-1,3-dioxin-4-one (3.55 g, 25.0 mmol, 1.00 equiv.), and xylene (10 mL) was stirred at 140 °C for 2 h. After cooling to room temperature, the xylene was evaporated and the residue was purified by flash column chromatography with heptane:ethyl acetate (90:10) as eluent afforded adamantan-1-yl 3-oxobutanoate (**RA 5-1**) as a colourless oil (5.15 g, 20.6 mmol, 82%). NMR data agreed with the previous report.[45]

^{1}H NMR (400 MHz, CDCl$_3$): δ 3.38 – 3.29 (m, 2H), 2.30 – 2.21 (m, 3H), 2.20 – 2.05 (m, *J* = 19.3 Hz, 9H), 1.63 (s, 6H) ppm.

^{13}C NMR (101 MHz, CDCl$_3$): δ 201.1, 166.0, 82.0, 51.6, 41.1 (3C), 36.0 (3C), 30.8 (3C), 29.9 ppm.

A Schlenk flask was charged with paraformaldehyde (11.0 mmol, 330 mg, 1.00 equiv.), ammonium acetate (16.5 mmol, 1.27 g, 1.50 equiv.), adamantan-1-yl 3-oxobutanoate (20.6 mmol, 5.15 g, 1.80 equiv.) and flushed with argon over three times. The neat reaction mixture was then heated to 80 °C and stirred at that temperature for 1 h, after which ice-cold water (30 mL) was added to the reaction mixture, causing precipitation of the product. The precipitate was recrystallised from methanol yielding a yellow solid (**RA 5**, 61%, 3.12 g).

^{1}H NMR (400 MHz, CDCl$_3$): δ 4.95 (s, 1H), 3.09 (s, 2H), 2.08 (s, 18H), 2.07 (s, 6H), 1.60 (s, 12H) ppm.

^{13}C NMR (101 MHz, CDCl$_3$): δ 167.4 (2C), 143.8 (2C), 100.9 (2C), 79.5 (2C), 41.6 (6C), 36.3, 30.8 (6C), 25.4 (6C), 19.3 (2C) ppm.

4.5: Bis(2,4-dimethylpentan-3-yl) 2,6-dimethyl-1,4-dihydropyridine-3,5-dicarboxylate **(RA 6).**

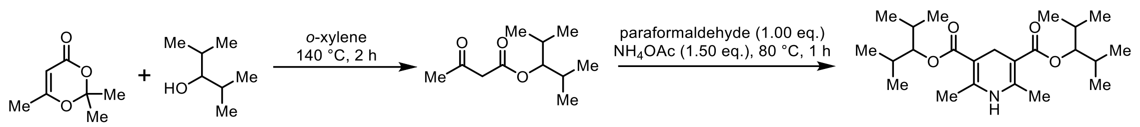

A mixture of 2,4-dimethyl-3-pentanol (3.81 g, 25.0 mmol, 1.00 equiv.), 2,2,6-trimethyl-4*H*-1,3-dioxin-4-one (3.55 g, 25.0 mmol, 1.00 equiv.), and xylene (10 mL) was stirred at 140 °C for 2 h. After cooling to room temperature, the xylene was evaporated and the residue was purified by column chromatography on silica gel (heptane/ethyl acetate 10:1 to 3:1) to afford 2,4-dimethylpentan-3-yl 3-oxobutanoate (**RA 6-1**) as a colourless oil (5.15 g, 20.6 mmol, 82%). NMR data agreed with the previous report.[46]

^{1}H NMR (400 MHz, CDCl$_3$): δ 4.62 – 4.52 (m, 1H), 3.41 (s, 2H), 2.22 (S, 3H), 1.93 – 1.76 (m, 2H), 1.03 – 0.66 (m, 12H) ppm.

^{13}C NMR (101 MHz, CDCl$_3$): δ 200.6, 167.0, 84.1, 50.1, 30.2, 29.3, 19.4 (2C), 17.1 (6C) ppm.

A Schlenk flask was charged with paraformaldehyde (10 mmol, 303 mg, 1.00 equiv.), ammonium acetate (15.0 mmol, 1.15 g, 1.50 equiv.), 2,4-dimethylpentan-3-yl 3-oxobutanoate (20.0 mmol, 4.00 g, 2.00 equiv.) and flushed with argon over three times. The neat reaction mixture was then heated to 80 °C and stirred at that temperature for 1 h, after which ice-cold water (30 mL) was added to the reaction mixture, causing precipitation of the product. The residue was recrystallised from methanol yielding a yellow solid **RA 6** (71%, 2.79 g).

^{1}H NMR (400 MHz, CDCl$_3$): δ 5.22 (s, 1H), 4.63 (t, *J* = 6.0 Hz, 2H), 3.36 (s, 2H), 2.20 (s, 6H), 1.90 (dq, *J* = 13.3, 6.7 Hz, 4H), 0.87 (dd, *J* = 6.7, 5.7 Hz, 24H) ppm.

^{13}C NMR (101 MHz, CDCl$_3$): δ 168.0 (2C), 144.8 (2C), 99.9 (2C), 81.5 (2C), 29.7 (4C), 25.3 (), 19.8 (4C), 19.1 (2C), 17.5 (4C) ppm.

HRMS (ESI$^+$) exact mass calculated for $[C_{23}H_{39}NO_4Na]^+$ ($[M+Na]^+$) required *m/z* 416.2771, found *m/z* 416.2769.

IR (neat) ν_{max}: 3343, 2965, 1737, 1635, 1497, 1296, 940, 904 cm^{-1}.

4.6: Bis(2,6-di-*tert*-butylphenyl) 2,6-dimethyl-1,4-dihydropyridine-3,5-dicarboxylate **(RA 7).**

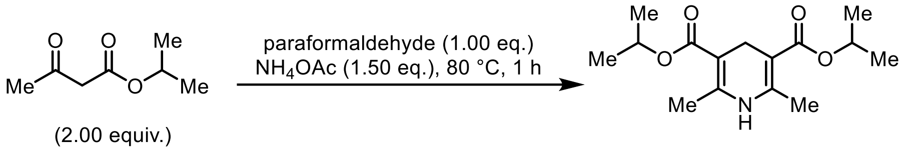

(2.00 equiv.)

RA 7

A Schlenk flask was flushed with argon three times and charged with paraformaldehyde (10.0 mmol, 303 mg, 1.00 equiv.), ammonium acetate (15.0 mmol, 1.15 g, 1.50 equiv.), isopropyl 3-oxobutanoate (20.0 mmol, 2.00 equiv.). The neat reaction mixture was then heated to 80 °C and stirred at that temperature for 1 h, after which ice-cold water (30 mL) was added to the reaction mixture, causing precipitation of the product. The precipitate was recrystallised from methanol yielding a yellow solid **RA 7** (83%, 2.33 g). NMR data agreed with the previous report.[47]

^{1}H NMR (400 MHz, $CDCl_3$): δ 5.22 (s, 1H), 5.03 (hept, *J* = 6.2 Hz, 2H), 3.23 (s, 2H), 2.17 (s, 6H), 1.25 (d, *J* = 6.3 Hz, 12H) ppm.

^{13}C NMR (101 MHz, $CDCl_3$): δ 167.8 (2C), 144.6 (2C), 99.9 (2C), 66.9 (2C), 25.0, 22.20 (2C), 19.3 (4C) ppm.

5. Stereochemical analysis

5.1: NOESY spectra of product **3a** (*cis*-2-Benzyl-5-phenyltetrahydrofuran).

The *cis*-configured product was identified as the major product, which also agreed with the previous report.[29]

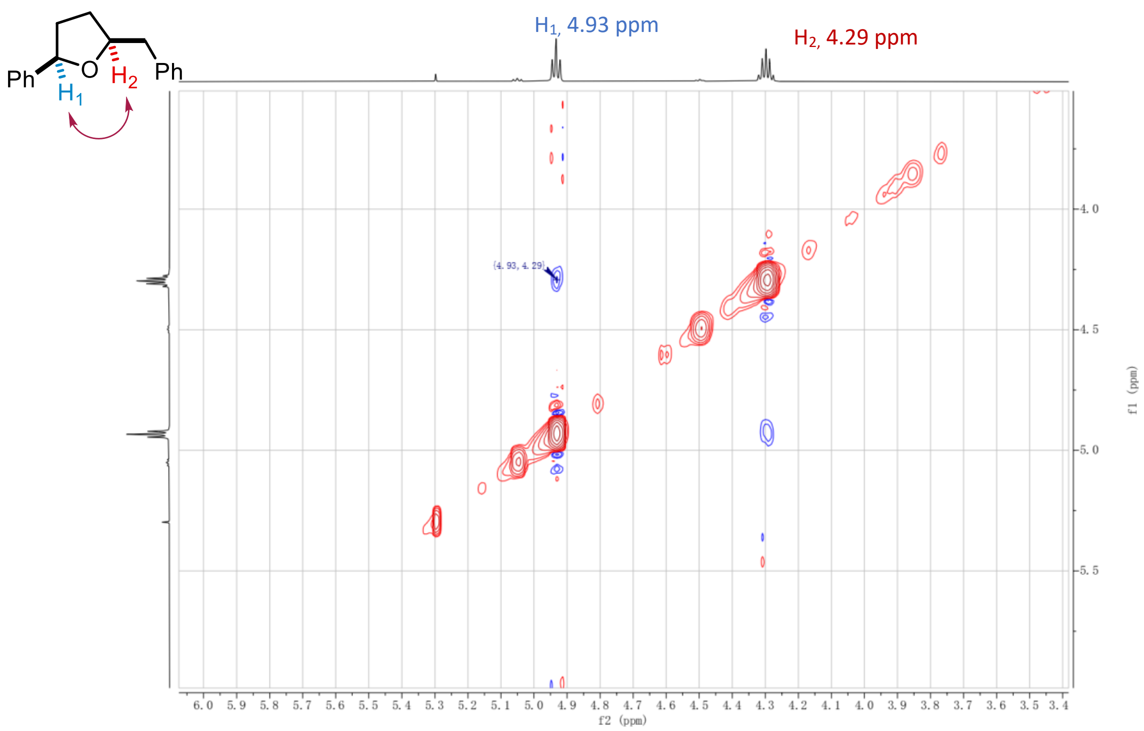

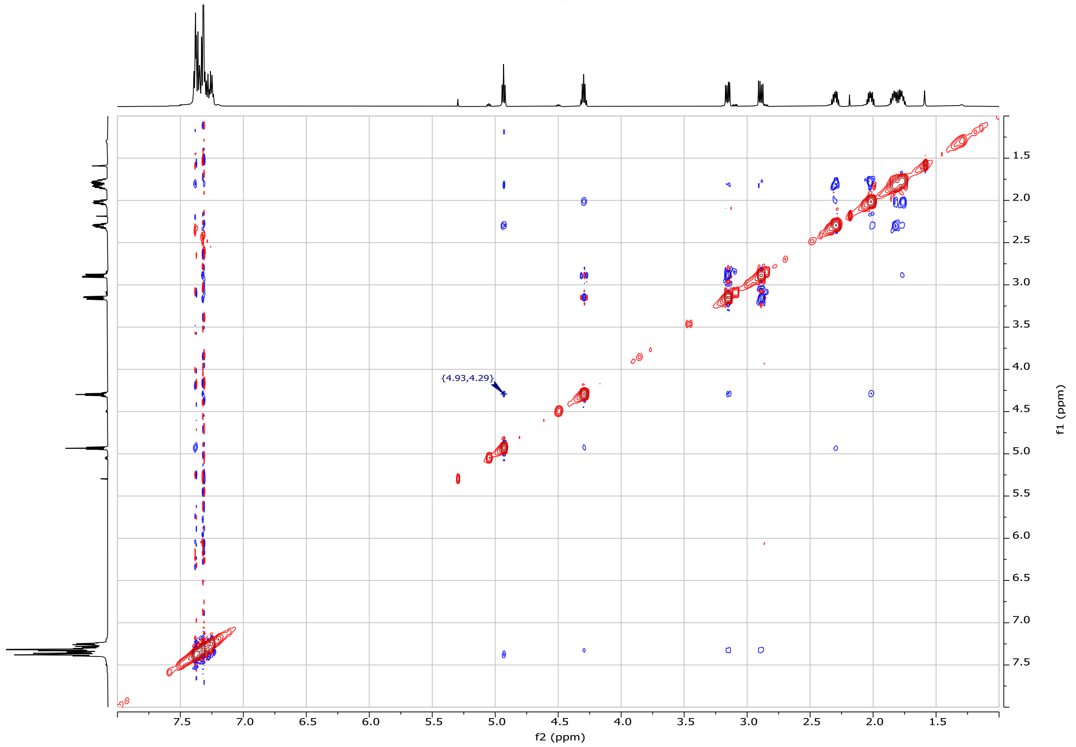

5.2: NOESY spectra of **5a** (7-Phenyl-6-oxabicyclo[3.2.1]octane).

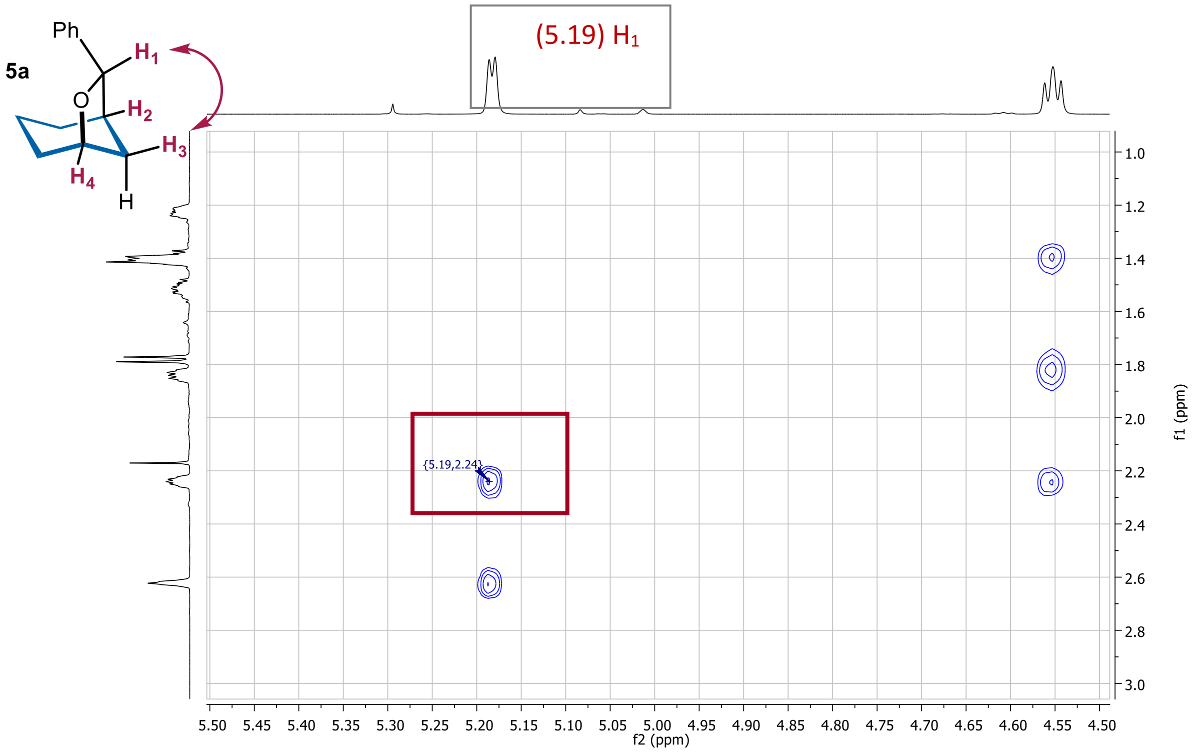

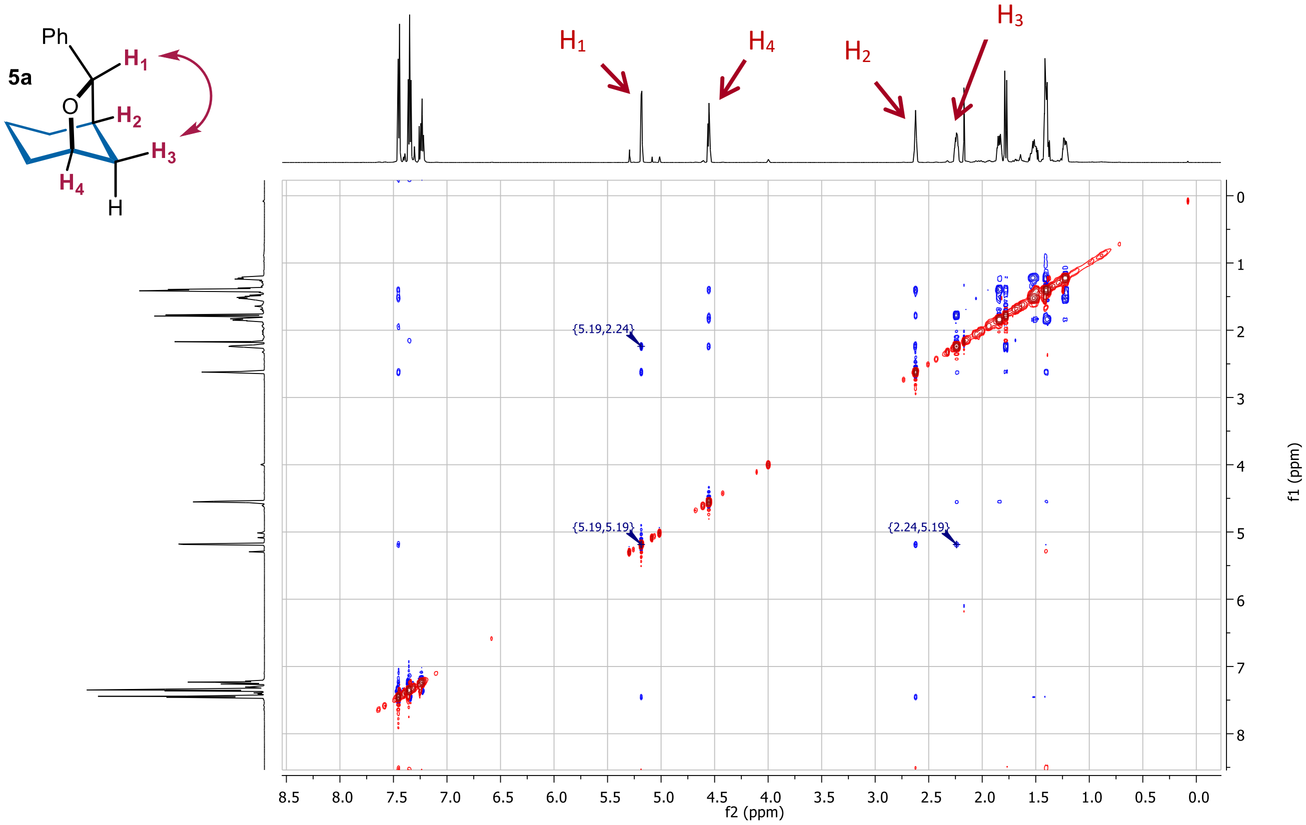

6 Experimental procedure and characterisation

6.1 Synthesis of cis-2,5-disubstituted THF products (3a-3s).

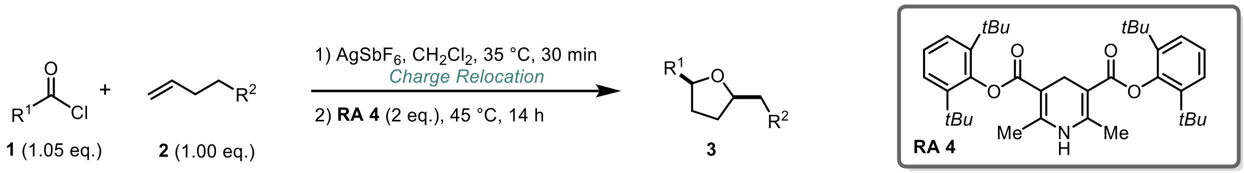

An oven-dried vial was flushed with argon and charged with a magnetic stir bar. After that, silver hexafluoroantimonate (0.22 mmol, 1.10 equiv.) and dichloromethane (2 mL) were charged into the vial, followed by the addition of alkene **2** (0.20 mmol, 1.00 equiv.). To the resulting mixture, acyl chloride **1** (0.21 mmol, 1.05 equiv.) was added and the vial was immediately placed in a heated sand bath at 35 °C. After 30 min, stirring was stopped and the supernatant was transferred by syringe to a separate vial. To this, reducing agent **RA 4** (0.40 mmol, 2.00 equiv.) was added. The vial was placed in a heated sand bath at 45 °C and the solution was stirred at that temperature for 14 h. After this time, the reaction mixture was treated with a saturated aqueous solution of $NaHCO_3$, extracted with DCM (3 × 5 mL), dried over anhydrous magnesium sulfate and filtered. The resulting filtrate was concentrated under reduced pressure and the resulting crude material was purified by flash column chromatography on silica gel (heptane/ethyl acetate) to give the title compounds.

3a: *cis*-2-Benzyl-5-phenyltetrahydrofuran

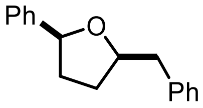

Synthesised following the general procedure, using 4-phenyl-1-butene (30.0 µL, 0.20 mmol, 1.00 equiv.), benzoyl chloride (24.4 µL, 0.21 mmol, 1.05 equiv.), silver hexafluoroantimonate (75.6 mg, 0.22 mmol, 1.10 equiv.), reducing agent **RA 4** (230.0 mg, 0.40 mmol, 2.00 equiv.) and dichloromethane (2 mL). Purification by flash column chromatography (heptane/ethyl acetate 95:15 to 90:10, Rf = 0.44 in heptane/ethyl acetate 10:1) gave the title compound (d.r. 15:1, 33.6 mg, 70%) as a colourless oil.

NMR data was reported only for the major isomer.

^{1}H NMR (400 MHz, CDCl$_3$): δ 7.41 – 7.32 (m, 4H), 7.32 (d, J = 2.6 Hz, 1H), 7.31 – 7.26 (m, 4H), 7.26 – 7.20 (m, 1H), 4.91 (t, J = 7.1 Hz, 1H), 4.32 – 4.24 (m, 1H), 3.14 (dd, J = 13.5, 6.0 Hz, 1H), 2.87 (dd, J = 13.6, 6.9 Hz, 1H), 2.34 – 2.25 (m, 1H), 2.05 – 1.96 (m, 1H), 1.85 – 1.72 (m, 2H) ppm.

^{13}C NMR (101 MHz, CDCl$_3$): δ 143.6, 138.9, 129.5 (2C), 128.5 (2C), 128.4 (2C), 127.3, 126.3, 125.9 (2C), 81.1, 80.8, 42.4, 34.4, 30.9 ppm.

HRMS (EI$^+$): exact mass calculated for $[M]^+$, $(C_{17}H_{18}O)^+$ required *m/z* 238.1352, found *m/z* 238.1351.

IR (neat) ν_{max}: 3027, 2935, 1495, 1360, 1272, 1080, 989, 697 cm^{-1}.

3b: *cis*-2-Benzyl-5-(2-iodophenyl)tetrahydrofuran

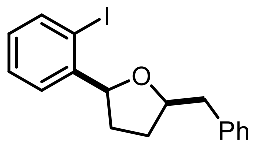

Synthesised following the general procedure, using 4-phenyl-1-butene (30.0 µL, 0.20 mmol, 1.00 equiv.), 2-iodobenzoyl chloride (29.0 µL, 0.21 mmol, 1.05 equiv.), silver hexafluoroantimonate (75.6 mg, 0.22 mmol, 1.10 equiv.), reducing agent **RA 4** (230 mg, 0.40 mmol, 2.00 equiv.) and dichloromethane (2 mL). Purification by flash column chromatography (heptane/ethyl acetate 99:1 to 90:10, Rf = 0.35 in heptane/ethyl acetate 10:1) gave the title compound (d.r. 15.4:1, 48.5 mg, 67%) as a colourless oil.

NMR data was reported only for the major isomer.

^{1}H NMR (400 MHz, CDCl$_3$): δ 7.79 (dd, *J* = 7.9, 1.1 Hz, 1H), 7.53 (dd, *J* = 7.8, 1.6 Hz, 1H), 7.39 – 7.34 (m, 1H), 7.33 – 7.29 (m, 4H), 7.23 (dd, *J* = 6.0, 2.7 Hz, 1H), 6.95 (td, *J* = 7.6, 1.7 Hz, 1H), 5.07 – 5.01 (m, 1H), 4.27 (dt, *J* = 10.1, 6.5 Hz, 1H), 3.18 (dd, *J* = 13.6, 6.2 Hz, 1H), 2.93 (dd, *J* = 13.6, 6.8 Hz, 1H), 2.59 – 2.47 (m, 1H), 2.01 (ddd, *J* = 9.0, 6.6, 4.1 Hz, 1H), 1.74 – 1.60 (m, 2H) ppm.

^{13}C NMR (101 MHz, CDCl$_3$): δ 146.0, 139.2, 138.8, 129.5 (2C), 128.9, 128.4, 128.2 (2C), 126.79, 126.5, 96.7, 84.2, 81.3, 42.1, 33.3, 30.7 ppm.

HRMS (EI^{+}) exact mass calculated for [M]$^{+}$, $(C_{17}H_{17}IO)^{+}$ required *m/z* 364.0319, found *m/z* 364.0319.

IR (neat) ν_{max}: 3060, 2968, 2866, 1496, 1435, 1267, 1097, 910, 750 cm^{-1}.

3c: *cis*-2-Benzyl-5-(4-fluorophenyl)tetrahydrofuran

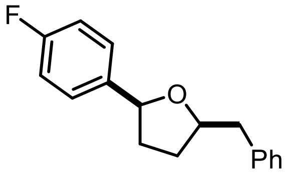

Synthesised following the general procedure, using 4-phenyl-1-butene (30.0 µL, 0.20 mmol, 1.00 equiv.), 4-fluorobenzoyl chloride (29.0 µL, 0.21 mmol, 1.05 equiv.), silver hexafluoroantimonate (75.6 mg, 0.22 mmol, 1.10 equiv.), reducing agent **RA 4** (230 mg, 0.40 mmol, 2.00 equiv.) and dichloromethane (2 mL). Purification by flash column chromatography (heptane/ethyl acetate 99:1 to 90:10, Rf = 0.41 in heptane/ethyl acetate 10:1) gave the title compound (d.r. 12.5:1, 27.0 mg, 53%) as a colourless oil.

NMR data was reported only for the major isomer.

^{1}H NMR (400 MHz, $CDCl_3$): δ 7.31 (ddd, *J* = 11.9, 5.7, 2.0 Hz, 6H), 7.25 – 7.20 (m, 1H), 7.05 – 6.97 (m, 2H), 4.87 (t, *J* = 7.0 Hz, 1H), 4.31 – 4.22 (m, 1H), 3.10 (dd, *J* = 13.5, 6.0 Hz, 1H), 2.86 (dd, *J* = 13.5, 6.9 Hz, 1H), 2.32 – 2.21 (m, 1H), 2.00 (dtd, *J* = 8.6, 5.1, 2.4 Hz, 1H), 1.81 – 1.68 (m, 2H) ppm.

^{13}C NMR (101 MHz, $CDCl_3$): δ 162.1 (d, *J* = 244.7 Hz), 139.1 (2C, d, *J* = 3.3 Hz), 138.7 (2C), 129.5, 128.4, 127.5 (2C, d, *J* = 8.0 Hz), 126.4, 115.1 (2C, d, *J* = 21.4 Hz), 80.7, 80.5, 42.3, 34.4, 30.8 ppm.

^{19}F NMR (377 MHz, $CDCl_3$): δ -115.87 ppm.

HRMS (EI$^+$) exact mass calculated for $[M]^+$, $(C_{17}H_{17}FO)^+$ required *m/z* 256.1258, found *m/z* 256.1252.

IR (neat) ν_{max}: 2969, 2944, 2933, 1738, 1727, 1509, 1365, 1226, 1217, 1058, 739, 699 cm^{-1}.

3d: *cis*-2-Benzyl-5-(2-bromophenyl)tetrahydrofuran

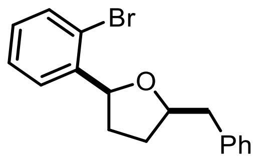

Synthesised following the general procedure, using 4-phenyl-1-butene (30.0 µL, 0.20 mmol, 1.00 equiv.), 2-bromobenzoyl chloride (29.0 µL, 0.21 mmol, 1.05 equiv.), silver hexafluoroantimonate (75.6 mg, 0.22 mmol, 1.10 equiv.), reducing agent **RA 4** (230 mg, 0.40 mmol, 2.00 equiv.) and dichloromethane (2 mL). Purification by flash column chromatography (heptane/ethyl acetate 99:1 to 90:10, Rf = 0.35 in heptane/ethyl acetate 10:1) gave the title compound (d.r. 10:1, 27.8 mg, 44%) as a colourless oil.

NMR data was reported only for the major isomer.

^{1}H NMR (400 MHz, CDCl$_3$): δ 7.61 (dd, *J* = 7.8, 2.3 Hz, 1H), 7.51 (dd, *J* = 8.0, 1.3 Hz, 1H), 7.37 – 7.29 (m, 5H), 7.24 (dt, *J* = 7.0, 2.6 Hz, 1H), 7.12 (td, *J* = 7.6, 1.8 Hz, 1H), 5.24 – 5.15 (m, 1H), 4.33 – 4.23 (m, 1H), 3.19 (dd, *J* = 13.5, 6.1 Hz, 1H), 2.94 (dd, *J* = 13.6, 6.8 Hz, 1H), 2.57 – 2.47 (m, 1H), 2.06 – 1.96 (m, 1H), 1.76 – 1.64 (m, 2H) ppm.

^{13}C NMR (101 MHz, CDCl$_3$): δ 143.1, 138.7, 132.5, 129.4 (2C), 128.5 (2C), 128.5, 127.5, 127.0, 126.4, 121.5, 81.1, 80.0, 42.1, 33.1, 30.7 ppm.

HRMS (EI$^+$) exact mass calculated for [M]$^+$, ($C_{17}H_{17}{}^{79}BrO$)$^+$ required *m/z* 316.0458, found *m/z* 316.0416.

IR (neat) ν_{max}: 3002, 2961, 2870, 1740, 1585, 1479, 1226, 1178, 1058, 998, 739 cm^{-1}.

3e: *cis*-2-Benzyl-5-(4-chlorophenyl)tetrahydrofuran

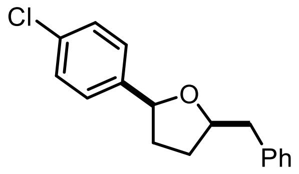

Synthesised following the general procedure, using 4-phenyl-1-butene (30.0 µL, 0.20 mmol, 1.00 equiv.), 4-chlorobenzoyl chloride (27.2 µL, 0.21 mmol, 1.05 equiv.), silver hexafluoroantimonate (75.6 mg, 0.22 mmol, 1.10 equiv.), reducing agent **RA 4** (230 mg, 0.40 mmol, 2.00 equiv.) and dichloromethane (2 mL). Purification by flash column chromatography (heptane/ethyl acetate 99:1 to 90:10, Rf = 0.45 in heptane/ethyl acetate 10:1) gave the title compound (d.r.>20:1, 27.1 mg, 50%) as a colourless oil.

NMR data was reported only for the major isomer.

^{1}H NMR (400 MHz, $CDCl_3$): δ 7.31 – 7.26 (m, 2H), 7.25 (d, *J* = 4.1 Hz, 5H), 7.22 (d, *J* = 0.7 Hz, 1H), 7.19 (ddd, *J* = 8.5, 4.1, 1.9 Hz, 1H), 4.83 (t, *J* = 7.0 Hz, 1H), 4.28 – 4.19 (m, 1H), 3.06 (dd, *J* = 13.5, 6.1 Hz, 1H), 2.83 (dd, *J* = 13.6, 6.8 Hz, 1H), 2.30 – 2.19 (m, 1H), 2.02 – 1.92 (m, 1H), 1.76 – 1.65 (m, 2H) ppm.

^{13}C NMR (101 MHz, $CDCl_3$): δ 142.1, 138.7, 132.8, 129.5 (2C), 128.4 (4C), 127.2 (2C), 126.4, 80.8, 80.3, 42.2, 34.4, 30.8 ppm.

HRMS (EI$^+$) exact mass calculated for $[M]^+$, $(C_{17}H_{17}ClO)^+$ required *m/z* 272.0962, found *m/z* 272.0961.

IR (neat) ν_{max}: 3027, 2940, 1738, 1686, 1491, 1426, 1363, 1228, 1184, 1075, 991, 744, 699 cm^{-1}.

3f: *cis*-2-Benzyl-5-(4-(trifluoromethyl)phenyl)tetrahydrofuran

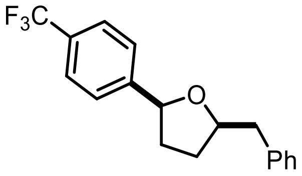

Synthesised following the general procedure, using 4-phenyl-1-butene (30.0 µL, 0.20 mmol, 1.00 equiv.), 4-(trifluoromethyl)benzoyl chloride (32.2 µL, 0.22 mmol, 1.05 equiv.), silver hexafluoroantimonate (75.6 mg, 0.22 mmol, 1.10 equiv.), reducing agent **RA 4** (230 mg, 0.40 mmol, 2.00 equiv.) and dichloromethane (2 mL). Purification by flash column chromatography (heptane/ethyl acetate 99:1 to 90:10, Rf = 0.41 in heptane/ethyl acetate 10:1) gave the title compound (d.r. 12:1, 36.8 mg, 60%) as a colourless oil.

NMR data was reported only for the major isomer.

^{1}H NMR (400 MHz, CDCl$_3$): δ 7.59 (d, *J* = 8.1 Hz, 2H), 7.45 (d, *J* = 8.6 Hz, 2H), 7.36 – 7.26 (m, 4H), 7.23 (ddd, *J* = 8.6, 4.1, 2.0 Hz, 1H), 4.96 (t, *J* = 6.9 Hz, 1H), 4.30 (p, *J* = 6.6 Hz, 1H), 3.11 (dd, *J* = 13.6, 6.2 Hz, 1H), 2.89 (dd, *J* = 13.6, 6.7 Hz, 1H), 2.38 – 2.28 (m, 1H), 2.06 – 1.96 (m, 1H), 1.82 – 1.67 (m, 2H) ppm.

^{13}C NMR (101 MHz, CDCl$_3$): δ 147.8, 138.7, 129.5 (2C), 129.3 (q, *J* = 32.7 Hz) 128.5 (2C), 126.5, 126.1(2C), 125.4 (q, *J* = 3.7 Hz, 2C), 124.27 (q, *J* = 271.8 Hz), 81.0, 80.4, 42.2, 34.4, 30.8 ppm.

^{19}F NMR (376 MHz, CDCl$_3$): δ -62.37 ppm.

HRMS (EI$^+$) exact mass calculated for [M]$^+$, ($C_{18}H_{17}F_3O$)$^+$ required *m/z* 306.1226, found *m/z* 306.1220.

IR (neat) ν_{max}: 3027, 2940, 1620, 1453, 1363, 1220, 1123, 1030, 991, 739, 699 cm^{-1}.

3g: *cis*-2-Benzyl-5-(*p*-tolyl)tetrahydrofuran

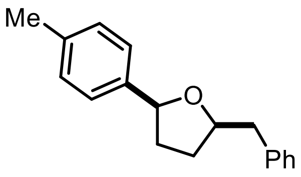

Synthesised following the general procedure, using 4-phenyl-1-butene (30.0 µL, 0.20 mmol, 1.00 equiv.), *p*-toluoyl chloride (28.3 µL, 0.21 mmol, 1.05 equiv.), silver hexafluoroantimonate (75.6 mg, 0.22 mmol, 1.10 equiv.), reducing agent **RA 4** (230 mg, 0.40 mmol, 2.00 equiv.) and dichloromethane (2 mL). Purification by flash column chromatography (heptane/ethyl acetate 99:1 to 90:10, Rf = 0.27 in heptane/ethyl acetate 10:1) gave the title compound (d.r. 5:1, 31.7 mg, 63%) as a colourless oil.

NMR data was reported only for the major isomer.

^{1}H NMR (400 MHz, $CDCl_3$): δ 7.32 – 7.26 (m, 4H), 7.25 (s, 1H), 7.24 – 7.19 (m, 2H), 7.14 (d, *J* = 7.9 Hz, 2H), 4.87 (t, *J* = 7.0 Hz, 1H), 4.30 – 4.21 (m, 1H), 3.11 (dd, *J* = 13.5, 6.0 Hz, 1H), 2.86 (d, *J* = 7.0 Hz, 1H), 2.34 (s, 3H), 2.28 – 2.20 (m, 1H), 2.02 – 1.93 (m, 1H), 1.82 – 1.70 (m, 2H) ppm.

^{13}C NMR (101 MHz, $CDCl_3$): δ 140.5, 138.9, 136.8, 129.5 (2C), 129.0 (2C), 128.4 (2C), 126.3, 125.9 (2C), 81.0, 80.7, 42.4, 34.3, 30.9, 21.2 ppm.

HRMS (EI$^+$) exact mass calculated for $[M]^+$, $(C_{18}H_{20}O)^+$ required *m/z* 252.1509, found *m/z* 252.1504.

IR (neat) ν_{max}: 3026, 2969, 2922, 2863, 1514, 1496, 1453, 1358, 1075, 1061, 1021, 812, 742, 699 cm^{-1}.

3h: *cis*-2-Benzyl-5-(3,5-dimethylphenyl)tetrahydrofuran

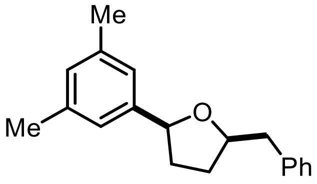

Synthesised following the general procedure, using 4-phenyl-1-butene (30.0 µL, 0.20 mmol, 1.00 equiv.), 3,5-dimethylbenzoyl chloride (32.1 µL, 0.21 mmol, 1.05 equiv.), silver hexafluoroantimonate (75.6 mg, 0.22 mmol, 1.10 equiv.), reducing agent **RA 4** (230 mg, 0.40 mmol, 2.00 equiv.) and dichloromethane (2 mL). Purification by flash column chromatography (heptane/ethyl acetate 99:1 to 90:10, Rf = 0.35 in heptane/ethyl acetate 10:1) gave the title compound (d.r. 10:1, 32.5 mg, 61%) as a colourless oil.

NMR data was reported only for the major isomer.

^{1}H NMR (400 MHz, $CDCl_3$): δ 7.35 – 7.29 (m, 4H), 7.24 – 7.19 (m, 1H), 6.98 (s, 2H), 6.91 (s, 1H), 4.85 (t, *J* = 7.1 Hz, 1H), 4.32 – 4.23 (m, 1H), 3.15 (dd, *J* = 13.5, 5.7 Hz, 1H), 2.88 (dd, *J* = 13.5, 7.2 Hz, 1H), 2.34 (s, 6H), 2.25 (tdd, *J* = 8.0, 5.3, 2.6 Hz, 1H), 2.03 – 1.95 (m, 1H), 1.85 – 1.73 (m, 2H) ppm.

^{13}C NMR (101 MHz, $CDCl_3$): δ 143.2, 138.8, 137.7, 129.5 (2C), 128.8, 128.3 (2C), 126.2, 123.6 (2C), 81.0, 80.5, 42.2, 34.1, 30.7, 21.4 (2C) ppm.

HRMS (EI$^+$) exact mass calculated for $[M]^+$, $(C_{19}H_{22}O)^+$ required *m/z* 266.1665, found *m/z* 266.1662.

IR (neat) ν_{max}: 3026, 2918, 2862, 1737, 1683, 1496, 1376, 1184, 1075, 892, 767 cm^{-1}.

3i: *cis*-2-Benzyl-5-(naphthalen-2-yl)tetrahydrofuran

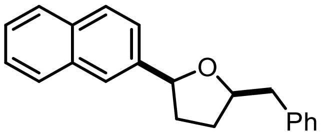

Synthesised following the general procedure, using 4-phenyl-1-buteneb(30.0 µL, 0.20 mmol, 1.00 equiv.), 2 naphthoyl chloride (40.8 mg, 0.21 mmol, 1.05 equiv.), silver hexafluoroantimonate (75.6 mg, 0.22 mmol, 1.10 equiv.), reducing agent **RA 4** (230 mg, 0.40 mmol, 2.00 equiv.) and dichloromethane (2 mL). Purification by flash column chromatography (heptane/ethyl acetate 99:1 to 90:10, Rf = 0.35 in heptane/ethyl acetate 10:1) gave the title compound (d.r. 6.6:1, 38.6 mg, 67%) as a colourless oil.

NMR data was reported only for the major isomer.

^{1}H NMR (400 MHz, $CDCl_3$): δ 7.85 – 7.79 (m, 4H), 7.50 – 7.43 (m, 3H), 7.32 (d, *J* = 5.0 Hz, 4H), 7.26 – 7.22 (m, 1H), 5.08 (t, *J* = 7.2 Hz, 1H), 4.34 (p, *J* = 6.9 Hz, 1H), 3.18 (dd, *J* = 13.5, 6.0 Hz, 1H), 2.93 (dd, *J* = 13.5, 7.0 Hz, 1H), 2.43 – 2.29 (m, 1H), 2.05 (ddt, *J* = 12.5, 8.4, 5.9 Hz, 1H), 1.95 – 1.85 (m, 1H), 1.85 – 1.77 (m, 1H) ppm.

^{13}C NMR (101 MHz, $CDCl_3$): δ 141.0, 138.9, 133.4, 133.0, 129.6 (2C), 128.5 (2C), 128.2, 128.1, 127.8, 126.4, 126.1, 125.7, 124.4, 124.3, 81.2, 81.0, 42.4, 34.4, 30.9 ppm.

HRMS (EI$^+$) exact mass calculated for $[M]^+$, $(C_{21}H_{20}O)^+$ required *m/z* 288.1509, found *m/z* 288.1508.

IR (neat) ν_{max}: 3058, 2933, 1737, 1601, 1374, 1210, 1125, 1109, 1029, 779 cm^{-1}.

3j: *cis*-2-Benzyl-5-cyclohexyltetrahydrofuran

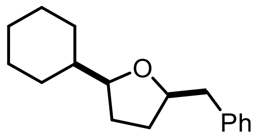

Synthesised following the general procedure, using 4-phenyl-1-buteneb(30.0 µL, 0.20 mmol, 1.00 equiv.), cyclohexane carbonyl chloride (28.8 µL, 0.21 mmol, 1.05 equiv.), silver hexafluoroantimonate (75.6 mg, 0.22 mmol, 1.10 equiv.), reducing agent **RA 4** (230 mg, 0.40 mmol, 2.00 equiv.) and dichloromethane (2 mL). Purification by flash column chromatography (heptane/ethyl acetate 99:1 to 90:10, Rf = 0.45 in heptane/ethyl acetate 10:1) gave the title compound (d.r.>20:1, 32 mg, 66%) as a colourless oil.

NMR data was reported only for the major isomer.

^{1}H NMR (400 MHz, $CDCl_3$): δ 7.30 – 7.25 (m, 2H), 7.24 – 7.16 (m, 3H), 4.09 – 3.99 (m, 1H), 3.60 – 3.51 (m, 1H), 2.98 (dd, *J* = 13.4, 5.6 Hz, 1H), 2.67 (dd, *J* = 13.4, 7.3 Hz, 1H), 2.00 – 1.90 (m, 1H), 1.88 – 1.78 (m, 2H), 1.78 – 1.70 (m, 2H), 1.69 – 1.60 (m, 2H), 1.57 – 1.49 (m, 2H), 1.36 (dddd, *J* = 15.0, 10.9, 6.9, 3.4 Hz, 1H), 1.28 – 1.13 (m, 3H), 1.05 – 0.90 (m, 2H) ppm.

^{13}C NMR (101 MHz, $CDCl_3$): δ 139.2, 129.5 (2C), 128.3 (2C), 126.2, 84.4, 79.9, 43.4, 42.5, 30.7, 30.0, 29.2, 28.6, 26.8, 26.3, 26.2 ppm.

HRMS (EI$^+$) exact mass calculated for $[M]^+$, $(C_{17}H_{24}O)^+$ required *m/z* 244.1822, found *m/z* 244.1815.

IR (neat) ν_{max}: 2932, 2841, 1738, 1435, 1216, 1165, 1093, 895, 697 cm^{-1}

3k: *cis*-2-Benzyl-5-isopropyltetrahydrofuran

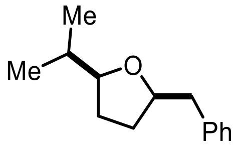

Synthesised following the general procedure, using 4-phenyl-1-butene (30.0 µL, 0.20 mmol, 1.00 equiv.), isobutyryl chloride (22.5 µL, 0.21 mmol, 1.05 equiv.), silver hexafluoroantimonate (75.6 mg, 0.22 mmol, 1.10 equiv.), reducing agent **RA 4** (230 mg, 0.40 mmol, 2.00 equiv.) and dichloromethane (2 mL). Purification by flash column chromatography (heptane/ethyl acetate 99:1 to 90:10, Rf = 0.41 in heptane/ethyl acetate 10:1) gave the title compound (d.r. >20:1, 25.3 mg, 62%) as a colourless oil.

NMR data was reported only for the major isomer.

^{1}H NMR (400 MHz, $CDCl_3$): δ 7.30 – 7.25 (m, 2H), 7.24 – 7.16 (m, 3H), 4.09 – 4.01 (m, 1H), 3.54 (q, *J* = 6.9 Hz, 1H), 2.97 (dd, *J* = 13.4, 5.8 Hz, 1H), 2.68 (dd, *J* = 13.4, 7.2 Hz, 1H), 1.89 – 1.78 (m, 2H), 1.68 (dq, *J* = 13.6, 6.9 Hz, 1H), 1.58 – 1.47 (m, 2H), 0.97 (d, *J* = 6.6 Hz, 3H), 0.87 (d, *J* = 6.8 Hz, 3H) ppm.

^{13}C NMR (101 MHz, $CDCl_3$): δ 139.2, 129.5 (2C), 128.3 (2C), 126.2, 85.1, 80.0, 42.5, 33.4, 30.8, 28.4, 19.5, 18.6 ppm.

HRMS (EI$^+$) exact mass calculated for $[M]^+$, $(C_{14}H_{20}O)^+$ required *m/z* 204.1509, found *m/z* 204.1505.

IR (neat) ν_{max}: 2957, 2936, 2871, 1737, 1727, 1365, 1229, 1216, 1205, 1076, 699 cm^{-1}.

3l: *cis*-2-Benzyl-5-isobutyltetrahydrofuran

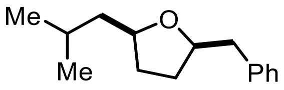

Synthesised following the general procedure, using 4-phenyl-1-butene (30.0 µL, 0.20 mmol, 1.00 equiv.), isovaleryl chloride (25.6 µL, 0.21 mmol, 1.05 equiv.), silver hexafluoroantimonate (75.6 mg, 0.22 mmol, 1.10 equiv.), reducing agent **RA 4** (230 mg, 0.40 mmol, 2.00 equiv.) and dichloromethane (2 mL). Purification by flash column chromatography (heptane/ethyl acetate 99:1 to 90:10, Rf = 0.41 in heptane/ethyl acetate 10:1) gave the title compound (d.r. 9.2:1, 29.0 mg, 66%) as a colourless oil.

NMR data was reported only for the major isomer.

^{1}H NMR (400 MHz, $CDCl_3$): δ 7.31 – 7.26 (m, 2H), 7.25 – 7.18 (m, 3H), 4.10 – 4.00 (m, 1H), 3.95 – 3.87 (m, 1H), 2.98 (dd, *J* = 13.4, 5.7 Hz, 1H), 2.74 – 2.66 (m, 1H), 1.98 – 1.81 (m, 2H), 1.79 – 1.68 (m, 1H), 1.64 – 1.50 (m, 2H), 1.46 – 1.38 (m, 1H), 1.35 – 1.25 (m, 1H), 0.94 (d, *J* = 1.4 Hz, 3H), 0.92 (d, *J* = 1.4 Hz, 3H) ppm.

^{13}C NMR (101 MHz, $CDCl_3$): δ 139.1, 129.5 (2C), 128.3(2C), 126.2, 79.9, 78.1, 45.6, 42.6, 31.5, 30.6, 25.7, 23.3, 22.9 ppm.

HRMS (EI$^+$) exact mass calculated for $[M]^+$, $(C_{15}H_{22}O)^+$ required *m/z* 218.1665, found *m/z* 218.1658.

IR (neat) ν_{max}: 2955, 2927, 2869, 1735, 1465, 1366, 1216, 1089, 743, 699 cm^{-1}.

3m: *cis*-2-Benzyl-5-(*tert*-butyl)tetrahydrofuran

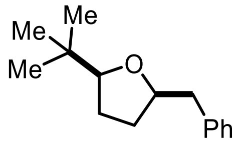

Synthesised following the general procedure, using 4-phenyl-1-butene (30.0 µL, 0.20 mmol, 1.00 equiv.), pivaloyl chloride (25.9 µL, 0.21 mmol, 1.05 equiv.), silver hexafluoroantimonate (75.6 mg, 0.22 mmol, 1.10 equiv.), reducing agent **RA 4** (230 mg, 0.40 mmol, 2.00 equiv.) and dichloromethane (2 mL). Purification by flash column chromatography (heptane/ethyl acetate 99:1 to 90:10, Rf = 0.54 in heptane/ethyl acetate 10:1) gave the title compound (d.r. >20:1; 28.0 mg, 64%) as a colourless oil.

NMR data was reported only for the major isomer.

^{1}H NMR (400 MHz, $CDCl_3$): δ 7.24 – 7.18 (m, 2H), 7.17 – 7.08 (m, 3H), 4.01 – 3.91 (m, 1H), 3.49 (t, *J* = 7.4 Hz, 1H), 2.91 (dd, *J* = 13.4, 5.7 Hz, 1H), 2.60 (dd, *J* = 13.4, 7.3 Hz, 1H), 1.75 (ddt, *J* = 14.3, 8.2, 6.0 Hz, 1H), 1.69 – 1.60 (m, 1H), 1.60 – 1.52 (m, 1H), 1.47 – 1.37 (m, 1H), 0.82 (s, 9H) ppm.

^{13}C NMR (101 MHz, $CDCl_3$): δ 139.2, 129.6 (2C), 128.3 (2C), 126.2, 87.6, 80.0, 42.2, 33.7, 31.0, 26.1, 26.0 (3C) ppm.

HRMS (EI$^+$) exact mass calculated for $[M]^+$, $(C_{15}H_{22}O)^+$ required *m/z* 218.1665, found *m/z* 218.1658.

IR (neat) ν_{max}: 2955, 2907, 2867, 1478, 1362, 1047, 699 cm^{-1}.

3n: *cis*-2-(Adamantan-1-yl)-5-benzyltetrahydrofuran

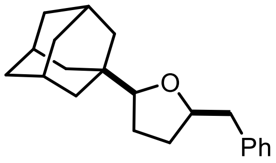

Synthesised following the general procedure, using 4-phenyl-1-butene (30.0 µL, 0.20 mmol, 1.00 equiv.), 1-adamantanecarbonyl chloride (40.6 mg, 0.21 mmol, 1.05 equiv.), silver hexafluoroantimonate (75.6 mg, 0.22 mmol, 1.10 equiv.), reducing agent **RA 4** (230 mg, 0.40 mmol, 2.00 equiv.) and dichloromethane (2 mL). Purification by flash column chromatography (heptane/ethyl acetate 99:1 to 90:10, Rf = 0.41 in heptane/ethyl acetate 10:1) gave the title compound (d.r. >20:1, 29.2 mg, 50%) as a colourless oil.

NMR data was reported only for the major isomer.

^{1}H NMR (400 MHz, CDCl$_3$): δ 7.31 – 7.26 (m, 2H), 7.25 – 7.18 (m, 3H), 4.07 – 3.94 (m, 1H), 3.42 (t, J = 7.3 Hz, 1H), 2.99 (dd, J = 13.3, 5.5 Hz, 1H), 2.66 (dd, J = 13.4, 7.4 Hz, 1H), 1.98 (s, 3H), 1.84 – 1.77 (m, 1H), 1.73 (ddd, J = 8.7, 6.1, 4.4 Hz, 4H), 1.69 – 1.63 (m, 4H), 1.61 (dd, J = 4.8, 2.6 Hz, 1H), 1.60 – 1.57 (m, 2H), 1.52 – 1.44 (m, 4H) ppm.

^{13}C NMR (101 MHz, CDCl$_3$): δ 139.2, 129.6 (2C), 128.3 (2C), 126.1, 87.8, 79.7, 42.1, 38.5 (3C), 37.5 (3C), 35.6, 30.8, 28.4 (3C), 24.4 ppm.

HRMS (EI$^+$) exact mass calculated for [M]$^+$, $(C_{21}H_{28}O)^+$ required *m/z* 297.2213, found *m/z* 297.2209.

IR (neat) ν_{max}: 2899, 2845, 1723, 1496, 1371, 1344, 1256, 1066, 748, 699 cm^{-1}.

3o: *cis*-2-Cyclohexyl-5-phenyltetrahydrofuran

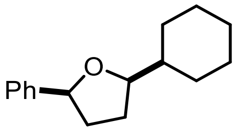

Synthesised following the general procedure, using allyl cyclohexane (31.20µL, 0.20 mmol, 1.00 equiv.), benzoyl chloride (24.9 µL, 0.21 mmol, 1.05 equiv.), silver hexafluoroantimonate (75.6 mg, 0.22 mmol, 1.10 equiv.), reducing agent **RA 4** (230 mg, 0.40 mmol, 2.00 equiv.) and dichloromethane (2 mL). Purification by flash column chromatography (heptane/ethyl acetate 99:1 to 90:10, Rf = 0.48 in heptane/ethyl acetate 10:1) gave the title compound (d.r. 17:1, 25 mg, 54%) as a colourless oil.

NMR data was reported only for the major isomer.

^{1}H NMR (600 MHz, $CDCl_3$): δ 7.38 – 7.30 (m, 4H), 7.23 (ddd, *J* = 8.6, 3.9, 1.9 Hz, 1H), 4.85 (t, *J* = 7.0 Hz, 1H), 3.73 (q, *J* = 7.1 Hz, 1H), 2.33 – 2.23 (m, 1H), 2.08 – 2.01 (m, 1H), 1.99 – 1.93 (m, 1H), 1.80 – 1.66 (m, 6H), 1.51 (ddd, *J* = 14.8, 7.1, 3.6 Hz, 1H), 1.33 – 1.17 (m, 3H), 1.13 – 1.01 (m, 2H) ppm.

^{13}C NMR (101 MHz, $CDCl_3$): δ 143.8, 128.3 (2C), 127.2, 126.0 (2C), 84.6, 80.6, 43.3, 34.7, 30.0, 29.3, 29.0, 26.8, 26.3, 26.2 ppm.

HRMS (EI$^+$) exact mass calculated for $[M]^+$, $(C_{16}H_{22}O)^+$ required *m/z* 230.1665, found *m/z* 230.1661.

IR (neat) $ν_{max}$: 2927, 2856, 1738, 1686, 1448, 1367, 1287, 1115, 1081, 992, 701 cm^{-1}.

3p: *cis*-2-Cyclopentyl-5-phenyltetrahydrofuran

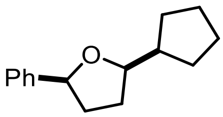

Synthesised following the general procedure, using allyl cyclopentane (27.8 µL, 0.20 mmol, 1.00 equiv.), benzoyl chloride (24.4 µL, 0.21 mmol, 1.05 equiv.), silver hexafluoroantimonate (75.6 mg, 0.22 mmol, 1.10 equiv.), reducing agent **RA 4** (230 mg, 0.40 mmol, 2.00 equiv.) and dichloromethane (2 mL). Purification by flash column chromatography (heptane/ethyl acetate 99:1 to 90:10, Rf = 0.58 in heptane/ethyl acetate 10:1) gave the title compound (d.r. 14.3:1, 30.0 mg, 69%) as a colourless oil.

NMR data was reported only for the major isomer.

^{1}H NMR (600 MHz, $CDCl_3$): δ 7.35 (d, *J* = 7.4 Hz, 2H), 7.32 (t, *J* = 7.5 Hz, 2H), 7.23 (t, *J* = 7.2 Hz, 1H), 4.87 (t, *J* = 7.2 Hz, 1H), 3.83 (q, *J* = 7.3 Hz, 1H), 2.28 (dt, *J* = 13.2, 7.0 Hz, 1H), 2.11 – 2.00 (m, 2H), 1.95 – 1.89 (m, 1H), 1.82 – 1.76 (m, 1H), 1.76 – 1.57 (m, 6H), 1.52 – 1.45 (m, 1H), 1.31 – 1.25 (m, 1H) ppm.

^{13}C NMR (151 MHz, $CDCl_3$): δ 143.9, 128.3 (2C), 127.2, 125.9 (2C), 84.5, 80.9, 45.5, 34.8, 30.4, 30.2, 29.3, 25.8, 25.7 ppm.

HRMS (EI$^+$) exact mass calculated for $[M]^+$, $(C_{15}H_{20}O)^+$ required *m/z* 216.1509, found *m/z* 216.1506.

IR (neat) ν_{max}: 2925, 2852, 1686, 1448, 1365, 1287, 1081, 991, 699 cm^{-1}.

3q: *cis*-2-Octyl-5-phenyltetrahydrofuran

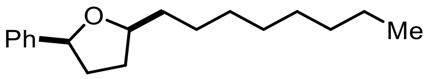

Synthesised following the general procedure, using 1-undecene (40.2 µL, 0.20 mmol, 1.00 equiv.), benzoyl chloride (24.9 µL, 0.21 mmol, 1.05 equiv.), silver hexafluoroantimonate (75.6 mg, 0.22 mmol, 1.10 equiv.), reducing agent **RA 4** (230 mg, 0.40 mmol, 2.00 equiv.) and dichloromethane (2 mL). Purification by flash column chromatography (heptane/ethyl acetate 99:1 to 90:10, Rf = 0.58 in heptane/ethyl acetate 10:1) gave the title compound (d.r. 10:1, 34.7 mg, 67%) as a colourless oil.

NMR data was reported only for the major isomer.

^{1}H NMR (400 MHz, $CDCl_3$): δ 7.38 – 7.30 (m, 4H), 7.26 – 7.21 (m, 1H), 4.87 (t, *J* = 7.3 Hz, 1H), 4.07 – 3.93 (m, 1H), 2.29 (dt, *J* = 19.1, 6.7 Hz, 1H), 2.06 (dt, *J* = 14.8, 6.5 Hz, 1H), 1.86 – 1.71 (m, 2H), 1.69 – 1.60 (m, 1H), 1.53 – 1.43 (m, 2H), 1.29 (s, 11H), 0.88 (m, 3H) ppm.

^{13}C NMR (101 MHz, $CDCl_3$): δ 143.8, 128.3 (2C), 127.2, 125.9 (2C), 80.9, 80.3, 36.2, 34.6, 32.0, 31.5, 29.9, 29.7, 29.4, 26.4, 22.8, 14.3 ppm.

HRMS (EI$^+$) exact mass calculated for $[M]^+$, $(C_{18}H_{28}O)^+$ required *m/z* 260.2135, found *m/z* 260.2125.

IR (neat) ν_{max}: 2955, 2925, 2854, 1740, 1464, 1377, 1092, 1057, 909, 699 cm^{-1}.

3r: *cis*-7-(5-Phenyltetrahydrofuran-2-yl)-1-(pyrrolidin-1-yl)heptan-1-one

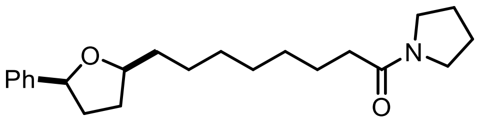

Synthesised following the general procedure, using 1-(pyrrolidin-1-yl)undec-10-en-1-one (48.4 µL, 0.20 mmol, 1.00 equiv.), benzoyl chloride (59.2 µL, 0.50 mmol, 2.5 equiv.), silver hexafluoroantimonate (172.0 mg, 0.50 mmol, 2.5 equiv.), reducing agent **RA 4** (230 mg, 0.40 mmol, 2.00 equiv.) and dichloromethane (2 mL). Purification by flash column chromatography (heptane/ethyl acetate 90:10 to 20:80, Rf = 0.21 in heptane/ethyl acetate 1:1) gave the title compound (d.r., 5.2:1, 49.6 mg, 44%) as a colourless oil.

NMR data was reported only for the major isomer.

^{1}H NMR (400 MHz, $CDCl_3$): δ 7.36 – 7.28 (m, 4H), 7.23 (ddd, *J* = 9.3, 4.1, 1.9 Hz, 1H), 4.86 (t, *J* = 7.3 Hz, 1H), 3.99 (p, *J* = 6.7 Hz, 1H), 3.45 (t, *J* = 6.8 Hz, 2H), 3.40 (t, *J* = 6.8 Hz, 2H), 2.33 – 2.21 (m, 3H), 2.05 (ddt, *J* = 12.3, 8.3, 6.5 Hz, 1H), 1.93 (q, *J* = 6.5 Hz, 3H), 1.88 – 1.80 (m, 3H), 1.71 – 1.60 (m, 3H), 1.40 – 1.31 (m, 9H) ppm.

^{13}C NMR (176 MHz, $CDCl_3$): δ 171.9, 143.7, 128.3 (2C), 127.1, 125.9 (2C), 80.8, 80.1, 46.7, 45.6, 36.1, 34.9, 34.6, 31.4, 29.7, 29.5, 29.5, 26.3, 26.2, 25.0, 24.5 ppm.

HRMS (EI$^+$) exact mass calculated for $[M]^+$, $(C_{22}H_{33}NO)^+$ required *m/z* 343.2506, found *m/z* 343.2500.

IR (neat) ν_{max}: 2927, 2854, 1635, 1445, 1429, 1050, 1027, 753, 699 cm^{-1}.

3s: *cis*-2-(3-Bromopropyl)-5-phenyltetrahydrofuran

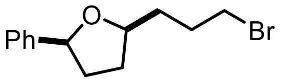

Synthesised following the general procedure, using 6-bromo-1-hexene (134.0 µL, 1.00 mmol, 1.00 equiv.), benzoyl chloride (128.0 µL, 1.05 mmol, 1.05 equiv.), silver hexafluoroantimonate (378.00 mg, 1.10 mmol, 1.10 equiv.), reducing agent **RA 4** (1.15.00 g, 2.00 mmol, 2.00 equiv.) and dichloromethane (2 mL). Purification by flash column chromatography (heptane/ethyl acetate 99:1 to 90:10, Rf = 0.58 in heptane/ethyl acetate 10:1) gave the title compound (d.r. >20:1, 65.3 mg, 49%) as a colourless oil.

NMR data was reported only for the major isomer.

^{1}H NMR (400 MHz, CDCl$_3$): δ 7.36 – 7.31 (m, 4H), 7.25 (qd, *J* = 5.5, 3.1 Hz, 1H), 4.87 (t, *J* = 7.3 Hz, 1H), 4.06 (dd, *J* = 13.0, 6.7 Hz, 1H), 3.56 – 3.45 (m, 2H), 2.34 – 2.25 (m, 1H), 2.11 (tdt, *J* = 9.4, 6.0, 4.7 Hz, 2H), 2.06 – 1.95 (m, 1H), 1.88 – 1.76 (m, 3H), 1.70 – 1.63 (m, 1H) ppm.

^{13}C NMR (101 MHz, CDCl$_3$): δ 143.3, 128.4 (2C), 127.3, 125.9 (2C), 81.1, 79.1, 34.7, 34.5, 34.2, 31.6, 29.8 ppm.

HRMS (EI$^+$) exact mass calculated for [M]$^+$, $(C_{13}H_{17}{}^{79}BrO)^+$ required *m/z* 268.0457, found *m/z* 268.0454.

IR (neat) ν_{max}: 3028, 2969, 2868, 1738, 1493, 1365, 1216, 1057, 944, 752, 699 cm^{-1}.

6.2: Synthesis of oxo-bridged bicyclic products **(5a–5ac).**

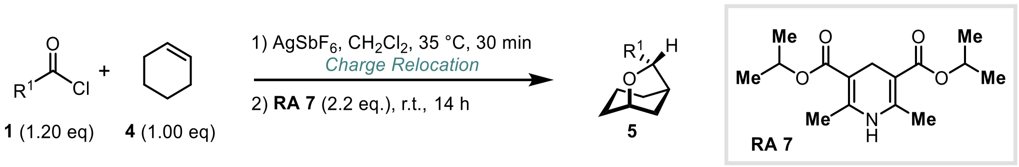

The oven-dried vial was flushed with argon and charged with a magnetic stir bar. After that, a solution of cyclohexene (0.20 mmol, 1.00 equiv.) and acyl chloride (0.24 mmol, 1.20 equiv.) in dichloromethane (2 mL) was treated with silver hexafluoroantimonate (0.24 mmol, 1.20 equiv.) under argon atmosphere, and the vial was immediately heated in a sand bath at 35 °C. After 30 min, stirring was stopped and the supernatant was transferred by syringe to a separate vial. To this, reducing agent **RA 7** (2.20 equiv.) was added. The vial was brought to ambient temperature (22–25 °C) and the solution was stirred at that temperature for 14 h. Then the reaction mixture was quenched with the saturated $NaHCO_3$ aqueous solution, extracted with dichloromethane (3×5 mL), dried over anhydrous magnesium sulfate, filtered, and concentrated under reduced pressure. The resulting crude product was purified by flash column chromatography on silica gel (heptane/ethyl acetate) to give the title compounds.

5a: 7-Phenyl-6-oxabicyclo[3.2.1]octane

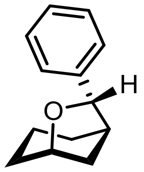

Synthesised following the general procedure, using cyclohexene (20.3 µL, 0.20 mmol, 1.00 equiv.), benzoyl chloride (27.9 µL, 0.24 mmol, 1.20 equiv.), silver hexafluoroantimonate (82.5 mg, 0.24 mmol, 1.20 equiv.), reducing agent **RA 7** (126 mg, 0.44 mmol, 2.20 equiv.) and dichloromethane (2 mL). Purification by flash column chromatography (heptane/ethyl acetate 99:1 to 90:10, Rf = 0.58 in heptane/ethyl acetate 10:1) gave the title compound (d.r. >20:1, 32.6 mg, 82%) as a colourless oil.

^{1}H NMR (400 MHz, $CDCl_3$): δ 7.48 – 7.43 (m, 2H), 7.35 (dd, *J* = 10.5, 4.9 Hz, 2H), 7.24 (dd, *J* = 10.7, 3.9 Hz, 1H), 5.18 (d, *J* = 4.0 Hz, 1H), 4.59 – 4.52 (m, 1H), 2.67 – 2.59 (m, 1H), 2.29 – 2.21 (m, 1H), 1.88 – 1.81 (m, 1H), 1.78 (d, *J* = 11.0 Hz, 1H), 1.58 – 1.48 (m, 1H), 1.41 (dt, *J* = 13.1, 6.4 Hz, 3H), 1.29 – 1.18 (m, 1H) ppm.

^{13}C NMR (101 MHz, $CDCl_3$): δ 140.9, 128.0 (2C), 126.2, 125.7 (2C), 83.2, 76.4, 39.4, 39.0, 32.2, 26.1, 18.0 ppm.

HRMS (EI$^+$) exact mass calculated for $[M]^+$, $(C_{13}H_{16}O)^+$ required *m/z* 188.1196, found *m/z* 188.1190.

IR (neat) ν_{max}: 2940, 1679, 1465, 1358, 1298, 1118, 892, 764, 701 cm^{-1}.

5b: 7-(4-Fluorophenyl)-6-oxabicyclo[3.2.1]octane

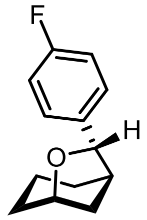

Synthesised following the general procedure, using cyclohexene (20.3 µL, 0.20 mmol, 1.00 equiv.), 4-fluorobenzoyl chloride (29.8 µL, 0.24 mmol, 1.20 equiv.), silver hexafluoroantimonate (82.5 mg, 0.24 mmol, 1.20 equiv.), reducing agent **RA 7** (126 mg, 0.44 mmol, 2.20 equiv.) and dichloromethane (2 mL). Purification by flash column chromatography (heptane/ethyl acetate 99:1 to 90:10, Rf = 0.32 in heptane/ethyl acetate 10:1) gave the title compound (d.r. >20:1, 33.4 mg, 81%) as a colourless oil.

^{1}H NMR (400 MHz, CDCl$_3$): δ 7.43 – 7.38 (m, 2H), 7.03 (t, *J* = 8.8 Hz, 2H), 5.12 (d, *J* = 3.9 Hz, 1H), 4.56 – 4.51 (m, 1H), 2.58 (d, *J* = 3.8 Hz, 1H), 2.22 (dtd, *J* = 8.2, 4.5, 2.1 Hz, 1H), 1.86 – 1.80 (m, 1H), 1.77 (d, *J* = 11.1 Hz, 1H), 1.47 – 1.35 (m, 4H), 1.27 – 1.20 (m, 1H) ppm.

^{19}F NMR (376 MHz, CDCl$_3$): δ -117.21 ppm.

^{13}C NMR (101 MHz, CDCl$_3$): δ 161.5 (d, *J* = 243.5 Hz), 136.4 (d, *J* = 3.0 Hz), 127.2 (d, *J* = 7.6 Hz), 114.8 (d, *J* = 21.3 Hz, 2C), 114.7, 82.7, 76.4, 39.4, 39.0, 32.1, 26.0, 18.0 ppm.

HRMS (EI$^+$) exact mass calculated for [M]$^+$, $(C_{13}H_{15}FO)^+$ required *m/z* 206.1101, found *m/z* 206.1098.

IR (neat) ν_{max}: 3332, 3320, 2944, 1738, 1680, 1465, 1219, 1054, 892, 669 cm^{-1}.

5c: 7-(3-Fluorophenyl)-6-oxabicyclo[3.2.1]octane

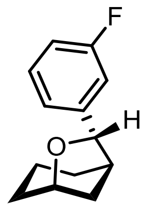

Synthesised following the general procedure, using cyclohexene (20.3 µL, 0.20 mmol, 1.00 equiv.), 3-fluorobenzoyl chloride (29.8 µL, 0.24 mmol, 1.20 equiv.), silver hexafluoroantimonate (82.5 mg, 0.24 mmol, 1.20 equiv.), reducing agent **RA 7** (126 mg, 0.44 mmol, 2.20 equiv.) and dichloromethane (2 mL). Purification by flash column chromatography (heptane/ethyl acetate 99:1 to 90:10, Rf = 0.36 in heptane/ethyl acetate 10:1) gave the title compound (d.r. >20:1, 25.7 mg, 63%) as a colourless oil.

^{1}H NMR (400 MHz, $CDCl_3$): δ 7.30 (td, *J* = 7.9, 6.0 Hz, 1H), 7.23 – 7.15 (m, 2H), 6.96 – 6.88 (m, 1H), 5.14 (d, *J* = 4.0 Hz, 1H), 4.57 – 4.50 (m, 1H), 2.61 (d, *J* = 3.3 Hz, 1H), 2.28 – 2.19 (m, 1H), 1.88 – 1.76 (m, 2H), 1.51 – 1.37 (m, 4H), 1.25 (dd, *J* = 9.9, 4.8 Hz, 1H) ppm.

^{19}F NMR (376 MHz, $CDCl_3$): δ -113.69 ppm.

^{13}C NMR (101 MHz, $CDCl_3$): δ 163.0 (d, *J* = 244.8 Hz).143.7 (d, *J* = 7.1 Hz), 129.5 (d, *J* = 8.2 Hz), 121.3 (d, *J* = 2.7 Hz), 113.0 (d, *J* = 21.8 Hz), 112 (d, *J* = 15.6 Hz), 82.8 (d, *J* = 1.9 Hz), 76.5, 39.4, 39.1, 32.1, 26.1, 18.1 ppm.

HRMS (EI^{+}) exact mass calculated for $[M]^+$, $(C_{13}H_{15}FO)^+$ required *m/z* 206.1101, found *m/z* 206.1100.

IR (neat) ν_{max}: 3342, 2940, 2858, 1723, 1488, 1286, 1054, 892, 761, 660 cm^{-1}.

5d: 7-(4-Chlorophenyl)-6-oxabicyclo[3.2.1]octane

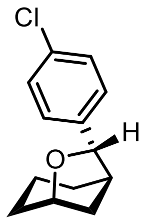

Synthesised following the general procedure, using cyclohexene (20.3 µL, 0.20 mmol, 1.00 equiv.), 4-chlorobenzoyl chloride (31.1 µL, 0.24 mmol, 1.20 equiv.), silver hexafluoroantimonate (82.5 mg, 0.24 mmol, 1.20 equiv.), reducing agent **RA 7** (126 mg, 0.44 mmol, 2.20 equiv.) and dichloromethane (2 mL). Purification by flash column chromatography (heptane/ethyl acetate 99:1 to 90:10, Rf = 0.33 in heptane/ethyl acetate 10:1) gave the title compound (d.r. >20:1, 28.7 mg, 65%) as a colourless oil.

^{1}H NMR (400 MHz, $CDCl_3$): δ 7.38 (d, *J* = 8.6 Hz, 2H), 7.31 (d, *J* = 8.5 Hz, 2H), 5.11 (d, *J* = 4.1 Hz, 1H), 4.56 – 4.51 (m, 1H), 2.59 (d, *J* = 3.2 Hz, 1H), 2.27 – 2.19 (m, 1H), 1.87 – 1.75 (m, 2H), 1.44 – 1.36 (m, 4H), 1.27 – 1.20 (m, 1H) ppm.

^{13}C NMR (101 MHz, $CDCl_3$): δ 139.4, 131.9, 128.2 (2C), 127.2 (2C), 82.7, 76.5, 39.4, 39.0, 32.0, 26.0, 18.0 ppm.

HRMS (EI$^+$) exact mass calculated for $[M]^+$, $(C_{13}H_{15}ClO)^+$ required *m/z* 222.0806, found *m/z* 222.0803.

IR (neat) ν_{max}: 3342, 2940, 1738, 1680, 1465, 1364, 1232, 1118, 1054, 988, 781 cm^{-1}.

5e: 7-(2-Bromophenyl)-6-oxabicyclo[3.2.1]octane

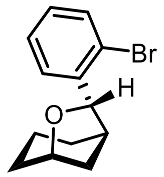

Synthesised following the general procedure, using cyclohexene (20.3 µL, 0.20 mmol, 1.00 equiv.), 2-bromobenzoyl chloride (31.4 µL, 0.24 mmol, 1.20 equiv.), silver hexafluoroantimonate (82.5 mg, 0.24 mmol, 1.20 equiv.), reducing agent **RA 7** (126 mg, 0.44 mmol, 2.20 equiv.) and dichloromethane (2 mL). Purification by flash column chromatography (heptane/ethyl acetate 99:1 to 90:10, Rf = 0.38 in heptane/ethyl acetate 10:1) gave the title compound (d.r. >20:1, 38.4 mg, 72%) as a colourless oil.

^{1}H NMR (400 MHz, CDCl$_3$): δ 7.94 (d, J = 7.6 Hz, 1H), 7.53 (dd, J = 7.9, 1.4 Hz, 1H), 7.32 (td, J = 7.6, 1.5 Hz, 1H), 7.12 (td, J = 7.9, 2.5 Hz, 1H), 5.10 (d, J = 4.1 Hz, 1H), 4.58 – 4.52 (m, 1H), 3.19 (p, J = 3.7 Hz, 1H), 2.27 (tdd, J = 6.8, 5.7, 3.4 Hz, 1H), 1.97 – 1.89 (m, 1H), 1.76 (d, J = 11.0 Hz, 1H), 1.73 – 1.61 (m, 1H), 1.53 – 1.42 (m, 2H), 1.35 – 1.27 (m, 1H), 1.20 (ddd, J = 10.3, 7.4, 3.5 Hz, 1H) ppm.

^{13}C NMR (101 MHz, CDCl$_3$): δ 139.7, 132.9, 128.9, 128.3, 126.8, 121.3, 84.0, 76.2, 39.2, 37.3, 31.9, 25.9, 17.8 ppm.

HRMS (EI$^+$) exact mass calculated for [M]$^+$, $(C_{13}H_{15}BrO)^+$ required *m/z* 266.0301, found *m/z* 266.0284.

IR (neat) ν_{max}: 2936, 1737, 1463,1364, 1215,1096, 1020, 988, 892, 675 cm^{-1}.

5f: 7-(2-Iodophenyl)-6-oxabicyclo[3.2.1]octane

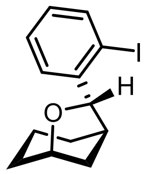

Synthesised following the general procedure, using cyclohexene (20.3 µL, 0.20 mmol, 1.00 equiv.), 2-iodobenzoyl chloride (33.8 µL, 0.24 mmol, 1.20 equiv.), silver hexafluoroantimonate (82.5 mg, 0.24 mmol, 1.20 equiv.), reducing agent **RA 7** (126 mg, 0.44 mmol, 2.20 equiv.) and dichloromethane (2 mL). Purification by flash column chromatography (heptane/ethyl acetate 99:1 to 90:10, Rf = 0.63 in heptane/ethyl acetate 10:1) gave the title compound (d.r. >20:1, 45.3 mg, 72%) as a colourless oil.

^{1}H NMR (400 MHz, CDCl$_3$): δ 7.90 (d, *J* = 7.8 Hz, 1H), 7.83 (dd, *J* = 7.8, 1.3 Hz, 1H), 7.35 (td, *J* = 7.6, 1.5 Hz, 1H), 6.99 – 6.93 (m, 1H), 4.98 (d, *J* = 4.0 Hz, 1H), 4.60 – 4.55 (m, 1H), 3.31 (p, *J* = 3.8 Hz, 1H), 2.27 (dtt, *J* = 11.0, 4.5, 2.3 Hz, 1H), 1.98 – 1.90 (m, 1H), 1.71 (dd, *J* = 25.5, 11.4 Hz, 2H), 1.52 – 1.40 (m, 2H), 1.32 (dt, *J* = 15.3, 7.6 Hz, 1H), 1.21 – 1.11 (m, 1H) ppm.

^{13}C NMR (101 MHz, CDCl$_3$): δ 142.5, 139.8, 128.6, 128.6, 127.5, 95.9, 87.1, 76.5, 39.0, 37.0, 31.8, 25.6, 17.6 ppm.

HRMS (EI$^+$) exact mass calculated for [M]$^+$, $(C_{13}H_{15}IO)^+$ required *m/z* 314.0162, found *m/z* 314.0165.

IR (neat) ν_{max}: 2933, 2851, 1720, 1433, 1206, 1034, 986, 757, 660 cm^{-1}.

5g: 7-(3,4-Dichlorophenyl)-6-oxabicyclo[3.2.1]octane

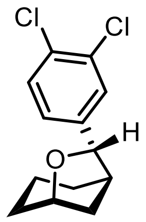

Synthesised following the general procedure, using cyclohexene (20.3 µL, 0.20 mmol, 1.00 equiv.), 3,4-dichlorobenzoyl chloride (51.3 mg, 0.24 mmol, 1.20 equiv.), silver hexafluoroantimonate (82.5 mg, 0.24 mmol, 1.20 equiv.), reducing agent **RA 7** (126 mg, 0.44 mmol, 2.20 equiv.) and dichloromethane (2 mL). Purification by flash column chromatography (heptane/ethyl acetate 99:1 to 90:10, Rf = 0.33 in heptane/ethyl acetate 10:1) gave the title compound (d.r. >20:1, 28.9 mg, 57%) as a colourless oil.

^{1}H NMR (400 MHz, $CDCl_3$): δ 7.56 (dd, *J* = 1.9, 1.1 Hz, 1H), 7.41 (d, *J* = 8.3 Hz, 1H), 7.27 – 7.23 (m, 1H), 5.07 (d, *J* = 4.1 Hz, 1H), 4.54 (dd, *J* = 6.1, 5.0 Hz, 1H), 2.59 (d, *J* = 2.9 Hz, 1H), 2.26 – 2.18 (m, 1H), 1.87 – 1.75 (m, 2H), 1.46 – 1.34 (m, 4H), 1.27 (d, *J* = 10.7 Hz, 1H) ppm.

^{13}C NMR (101 MHz, $CDCl_3$): δ 141.3, 132.20 (2C), 130.1, 127.9, 125.3, 82.20 , 76.7, 39.3, 38.9, 31.9, 26.0, 18.0 ppm.

HRMS (EI$^+$): exact mass calculated for $[M]^+$, $(C_{13}H_{14}Cl_2O)^+$ required *m/z* 256.0416, found *m/z* 256.0420.

IR (neat) ν_{max}: 2934, 1681, 1465, 1385, 1265, 1060, 986, 823, 674 cm^{-1}.

5h: 7-(4-(Trifluoromethyl)phenyl)-6-oxabicyclo[3.2.1]octane

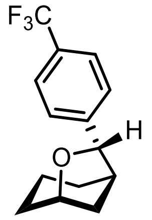

Synthesised following the general procedure, using cyclohexene (20.3 µL, 0.20 mmol, 1.00 equiv.), 4-(trifluoromethyl)benzoyl chloride (36.8 µL, 0.24 mmol, 1.20 equiv.), silver hexafluoroantimonate (82.5 mg, 0.24 mmol, 1.20 equiv.), reducing agent **RA 7** (126 mg, 0.44 mmol, 2.20 equiv.) and dichloromethane (2 mL). Purification by flash column chromatography (heptane/ethyl acetate 99:1 to 90:10, Rf = 0.28 in heptane/ethyl acetate 10:1) gave the title compound (d.r. >20:1, 24.2 mg, 48%) as a colourless oil.

^{1}H NMR (400 MHz, CDCl$_3$): δ 7.61 (d, *J* = 8.5 Hz, 2H), 7.56 (d, *J* = 8.4 Hz, 2H), 5.18 (d, *J* = 3.6 Hz, 1H), 4.57 (dd, *J* = 6.0, 5.1 Hz, 1H), 2.66 (s, 1H), 2.29 – 2.21 (m, 1H), 1.83 (ddd, *J* = 18.3, 10.7, 6.8 Hz, 2H), 1.49 – 1.32 (m, 4H), 1.28 – 1.21 (m, 1H) ppm.

^{13}C NMR (101 MHz, CDCl$_3$): δ 145.1 (d, *J* = 1.1 Hz), 128.6 (q, *J* = 32.20 Hz), 126.1 (2C), 125.0 (q, *J* = 3.8 Hz, 2C),124.5 (q, *J* = 271.6 Hz), 82.9, 76.6, 39.4, 39.1, 32.0, 26.0, 18.4 ppm.

^{19}F NMR (377 MHz, CDCl$_3$): δ 63.13 (3F) ppm.

HRMS (EI$^+$): exact mass calculated for $[M]^+$, $(C_{13}H_{15}F_3O)^+$ required *m/z* 256.1070, found *m/z* 256.1064.

IR (neat) ν_{max}: 2968, 1738, 1615, 1321, 1193, 1094, 990, 899, 712 cm^{-1}.

5i: 7-(4-Nitrophenyl)-6-oxabicyclo[3.2.1]octane

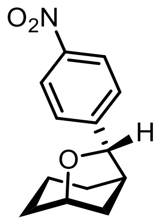

Synthesised following the general procedure, using cyclohexene (20.3 µL, 0.20 mmol, 1.00 equiv.), 4-nitrobenzoyl chloride (46.7 mg, 0.24 mmol, 1.20 equiv.), silver hexafluoroantimonate (82.5 mg, 0.24 mmol, 1.20 equiv.), reducing agent **RA 7** (126 mg, 0.44 mmol, 2.20 equiv.) and dichloromethane (2 mL). Purification by flash column chromatography (heptane/ethyl acetate 99:1 to 90:10, Rf = 0.33 in heptane/ethyl acetate 10:1) gave the title compound (d.r. >20:1, 23.7 mg, 51%) as a colourless oil.

^{1}H NMR (400 MHz, CDCl$_3$): δ 8.22 (d, *J* = 8.8 Hz, 2H), 7.64 – 7.59 (m, 2H), 5.19 (d, *J* = 4.2 Hz, 1H), 4.62 – 4.55 (m, 1H), 2.70 (d, *J* = 3.3 Hz, 1H), 2.31 – 2.22 (m, 1H), 1.85 (dt, *J* = 17.6, 8.2 Hz, 2H), 1.43 (ddd, *J* = 10.7, 7.1, 1.9 Hz, 2H), 1.37 – 1.24 (m, 3H) ppm.

^{13}C NMR (101 MHz, CDCl$_3$): δ 148.8, δ 146.8, 126.7 (2C), 123.5 (2C), 82.8 (2C), 39.4, 39.2, 31.9, 26.0, 18.0 ppm.

HRMS (EI$^+$): exact mass calculated for $[M]^+$, $(C_{13}H_{15}NO_3)^+$ required *m/z* 233.1046, found *m/z* 233.1053.

IR (neat) ν_{max}: 2938, 1738, 1603, 1493, 1339, 1269, 1193, 1097, 990, 891, 699 cm^{-1}.

5j: Methyl 4-(6-oxabicyclo[3.2.1]octan-7-yl)benzoate

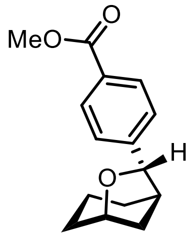

Synthesised following the general procedure, using cyclohexene (20.3 µL, 0.20 mmol, 1.00 equiv.), methyl 4-chlorocarbonylbenzoate (50.2 mg, 0.24 mmol, 1.20 equiv.), silver hexafluoroantimonate (82.5 mg, 0.24 mmol, 1.20 equiv.), reducing agent **RA 7** (126 mg, 0.44 mmol, 2.20 equiv.) and dichloromethane (2 mL). Purification by flash column chromatography (heptane/ethyl acetate 99:1 to 90:10, Rf = 0.12 in heptane/ethyl acetate 10:1) gave the title compound (d.r. >20:1, 32.20 mg, 66%) as a colourless oil.

^{1}H NMR (400 MHz, $CDCl_3$): δ 8.04 – 7.99 (m, 2H), 7.55 – 7.49 (m, 2H), 5.18 (d, *J* = 4.1 Hz, 1H), 4.55 (dd, *J* = 6.0, 5.0 Hz, 1H), 3.91 (s, 3H), 2.66 (d, *J* = 2.9 Hz, 1H), 2.29 – 2.21 (m, 1H), 1.89 – 1.76 (m, 2H), 1.46 – 1.37 (m, 3H), 1.35 (dd, *J* = 6.0, 3.0 Hz, 1H), 1.23 (dt, *J* = 7.9, 4.6 Hz, 1H) ppm.

^{13}C NMR (101 MHz, $CDCl_3$): δ 167.3, 146.4, 129.4 (2C), 128.3, 125.8 (2C), 83.1, 76.6, 52.1, 39.4, 39.1, 32.0, 26.0, 18.0 ppm.

HRMS (EI$^+$): exact mass calculated for $[M]^+$, $(C_{15}H_{18}O_3)^+$ required *m/z* 246.1250, found *m/z* 246.1248.

IR (neat) ν_{max}: 2941, 1720, 1610, 1435, 1308, 1233, 1204, 1107, 1074, 749 cm^{-1}.

5k: 7-(4-(Trifluoromethoxy)phenyl)-6-oxabicyclo[3.2.1]octane

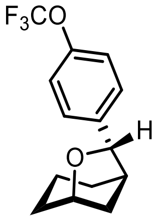

Synthesised following the general procedure, using cyclohexene (20.3 µL, 0.20 mmol, 1.00 equiv.), 4-(trifluoromethoxy)benzoyl chloride (38.7 µL, 0.24 mmol, 1.20 equiv.), silver hexafluoroantimonate (82.5 mg, 0.24 mmol, 1.20 equiv.), reducing agent **RA 7** (126 mg, 0.44 mmol, 2.20 equiv.) and dichloromethane (2 mL). Purification by flash column chromatography (heptane/ethyl acetate 99:1 to 90:10, Rf = 0.34 in heptane/ethyl acetate 10:1) gave the title compound (d.r. >20:1, 33.2 mg, 61%) as a colourless oil.

^{1}H NMR (400 MHz, CDCl$_3$): δ 7.46 (dd, *J* = 7.4, 1.4 Hz, 2H), 7.19 (d, *J* = 8.1 Hz, 2H), 5.14 (d, *J* = 4.1 Hz, 1H), 4.58 – 4.52 (m, 1H), 2.61 (d, *J* = 2.6 Hz, 1H), 2.28 – 2.20 (m, 1H), 1.86 – 1.75 (m, 2H), 1.41 (qd, *J* = 11.3, 4.5 Hz, 4H), 1.25 (dd, *J* = 10.2, 5.4 Hz, 1H) ppm.

^{13}C NMR (101 MHz, CDCl$_3$): δ 147.7 (q, *J* = 2.0 Hz), 139.6, 127.1 (2C), 120.6 (q, *J* = 256.5 Hz), 120.6 (2C), 82.7, 76.5, 39.4, 39.0, 32.0, 26.0, 18.0 ppm.

^{19}F NMR (376 MHz, CDCl$_3$): δ 57.61 (3F) ppm.

HRMS (EI$^+$): exact mass calculated for [M]$^+$, $(C_{14}H_{15}F_3O_2)^+$ required *m/z* 272.1019, found *m/z* 272.1019.

IR (neat) ν_{max}: 3333, 2862, 1602, 1467, 1300, 1206, 1120, 998, 892, 702 cm^{-1}.

5l: 7-(3-Methoxyphenyl)-6-oxabicyclo[3.2.1]octane

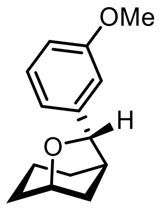

Synthesised following the general procedure, using cyclohexene (20.3 µL, 0.20 mmol, 1.00 equiv.), 3-methoxybenzoyl chloride (34.4 µL, 0.24 mmol, 1.20 equiv.), silver hexafluoroantimonate (82.5 mg, 0.24 mmol, 1.20 equiv.), reducing agent **RA 7** (126 mg, 0.44 mmol, 2.20 equiv.) and dichloromethane (2 mL). Purification by flash column chromatography (heptane/ethyl acetate 99:1 to 90:10, Rf = 0.54 in heptane/ethyl acetate 10:1) gave the title compound (d.r. >20:1, 28.0 mg, 64%) as a colourless oil.

^{1}H NMR (400 MHz, CDCl$_3$): δ 7.26 (q, *J* = 7.8 Hz, 1H), 7.08 – 7.05 (m, 1H), 6.99 (dd, *J* = 7.6, 1.0 Hz, 1H), 6.77 (dd, *J* = 8.2, 2.6 Hz, 1H), 5.15 (d, *J* = 4.1 Hz, 1H), 4.57 – 4.51 (m, 1H), 3.83 (s, 3H), 2.63 – 2.57 (m, 1H), 2.23 (dtd, *J* = 13.2, 4.5, 2.1 Hz, 1H), 1.89 – 1.80 (m, 1H), 1.77 (d, *J* = 11.0 Hz, 1H), 1.57 – 1.50 (m, 1H), 1.45 – 1.35 (m, 3H), 1.24 (dt, *J* = 12.1, 5.9 Hz, 1H) ppm.

^{13}C NMR (101 MHz, CDCl$_3$): δ 159.5, 142.6, 129.0, 118.2, 111.6, 111.5, 83.1, 76.4, 55.3, 39.4, 39.1, 32.2, 26.2, 18.2 ppm.

HRMS (EI$^+$) exact mass calculated for [M]$^+$, $(C_{14}H_{18}O_2)^+$ required *m/z* 218.1301, found *m/z* 218.1299.

IR (neat) ν_{max}: 2940, 1680, 1596, 1451, 1357, 1218, 1118, 1084, 986, 683 cm^{-1}.

5m: 7-(4-(*tert*-Butyl)phenyl)-6-oxabicyclo[3.2.1]octane

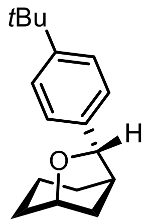

Synthesised following the general procedure, using cyclohexene (20.3 µL, 0.20 mmol, 1.00 equiv.), 4-*tert*-butylbenzoyl chloride (29.8 µL, 0.24 mmol, 1.20 equiv.), silver hexafluoroantimonate (82.5 mg, 0.24 mmol, 1.20 equiv.), reducing agent **RA 4** (252 mg, 0.44 mmol, 2.20 equiv.) and dichloromethane (2 mL). Purification by flash column chromatography (heptane/ethyl acetate 99:1 to 90:10, Rf = 0.43 in heptane/ethyl acetate 10:1) gave the title compound (d.r. >17:1, 42.4 mg, 87%) as a colourless oil.

NMR data was reported only for the major isomer.

^{1}H NMR (400 MHz, $CDCl_3$): δ 7.36 (s, 4H), 5.16 (d, *J* = 4.1 Hz, 1H), 4.57 – 4.51 (m, 1H), 2.61 (d, *J* = 3.1 Hz, 1H), 2.29 – 2.19 (m, 1H), 1.83 (dd, *J* = 12.7, 6.2 Hz, 1H), 1.77 (d, *J* = 11.0 Hz, 1H), 1.53 (ddd, *J* = 18.3, 8.8, 3.7 Hz, 1H), 1.41 (dd, *J* = 12.7, 6.1 Hz, 3H), 1.36 – 1.32 (m, 9H), 1.23 (dd, *J* = 12.5, 6.0 Hz, 1H) ppm.

^{13}C NMR (151 MHz, $CDCl_3$): δ 148.9, 137.7, 125.4 (2C), 124.9 (2C), 83.2, 76.3, 39.4, 39.0, 34.5, 32.3, 31.6 (3C), 26.2, 18.2 ppm.

HRMS (EI^{+}) exact mass calculated for $[M]^{+}$, $(C_{17}H_{24}O)^{+}$ required *m/z* 244.1822, found *m/z* 244.1808.

IR (neat) ν_{max}: 3332, 2944, 2859, 1738, 1680, 1364, 1118, 957, 775 cm^{-1}.

5n: 7-(3,5-Dimethylphenyl)-6-oxabicyclo[3.2.1]octane

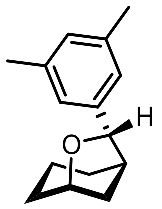

Synthesised following the general procedure, using cyclohexene (20.3 µL, 0.20 mmol, 1.00 equiv.), 3,5-dimethylbenzoyl chloride (36.7 µL, 0.24 mmol, 1.20 equiv.), silver hexafluoroantimonate (82.5 mg, 0.24 mmol, 1.20 equiv.), reducing agent **RA 7** (126 mg, 0.44 mmol, 2.20 equiv.) and dichloromethane (2 mL). Purification by flash column chromatography (heptane/ethyl acetate 99:1 to 90:10) gave the title compound (d.r. 10:1, 29.0 mg, 67%) as a colourless oil.

NMR data was reported only for the major isomer.

^{1}H NMR (400 MHz, CDCl$_3$): δ 7.06 (s, 2H), 6.88 (s, 1H), 5.13 (d, *J* = 3.8 Hz, 1H), 4.56 – 4.51 (m, 1H), 2.60 (s, 1H), 2.33 (s, 6H), 2.22 (ddd, *J* = 8.8, 6.3, 4.2 Hz, 1H), 1.85 (dt, *J* = 11.3, 4.8 Hz, 1H), 1.77 (d, *J* = 11.0 Hz, 1H), 1.57 (ddt, *J* = 18.7, 12.9, 6.2 Hz, 1H), 1.46 – 1.35 (m, 3H), 1.24 (dt, *J* = 12.0, 5.8 Hz, 1H) ppm.

^{13}C NMR (101 MHz, CDCl$_3$): δ 140.7, 137.5 (2C), 127.8, 123.5 (2C), 83.3, 76.3, 39.4, 39.0, 32.2, 26.2, 21.5, 18.2 ppm.

HRMS (EI$^+$) exact mass calculated for [M]$^+$, $(C_{15}H_{20}O)^+$ required *m/z* 216.1509, found *m/z* 216.1506.

IR (neat) ν_{max}: 2936, 1738, 1674, 1448, 1364, 1216, 1118, 1050, 990, 895 cm^{-1}.

5o: 7-(Naphthalen-2-yl)-6-oxabicyclo[3.2.1]octane

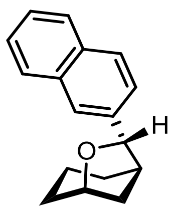

Synthesised following the general procedure, using cyclohexene (20.3 µL, 0.20 mmol, 1.00 equiv.), 2-naphthoyl chloride (46.7 mg, 0.24 mmol, 1.20 equiv.), silver hexafluoroantimonate (82.5 mg, 0.24 mmol, 1.20 equiv.), reducing agent **RA 7** (126 mg, 0.44 mmol, 2.20 equiv.) and dichloromethane (2 mL). Purification by flash column chromatography (heptane/ethyl acetate 99:1 to 90:10, Rf = 0.33 in heptane/ethyl acetate 10:1) gave the title compound (d.r. >20:1, 31.3 mg, 66%) as a colourless oil.

^{1}H NMR (400 MHz, CDCl$_3$): δ 8.03 (s, 1H), 7.89 – 7.80 (m, 3H), 7.51 – 7.42 (m, 3H), 5.34 (d, *J* = 4.0 Hz, 1H), 4.65 – 4.60 (m, 1H), 2.77 – 2.69 (m, 1H), 2.36 – 2.27 (m, 1H), 1.90 (dd, *J* = 12.5, 6.0 Hz, 1H), 1.82 (d, *J* = 10.9 Hz, 1H), 1.66 – 1.52 (m, 1H), 1.43 (ddd, *J* = 9.6, 9.0, 4.9 Hz, 3H), 1.21 (dd, *J* = 12.4, 6.5 Hz, 1H) ppm.

^{13}C NMR (101 MHz, CDCl$_3$): δ 138.3, 133.5, 132.4, 128.0, 127.73, 127.69, 126.0, 125.4, 124.4, 124.1, 83.5, 76.5, 39.5, 39.1, 32.2, 26.2, 18.1 ppm.

HRMS (EI$^+$): exact mass calculated for [M]$^+$, ($C_{17}H_{18}O$)$^+$ required *m/z* 238.1352, found *m/z* 238.1348.

IR (neat) ν_{max}: 3053, 2853, 1738, 1601, 1462, 1364, 1231, 1117, 993, 897, 712 cm^{-1}.

5p: 7-Pentadecyl-6-oxabicyclo[3.2.1]octane

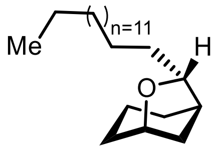

Synthesised following the general procedure, using cyclohexene (20.3 µL, 0.20 mmol, 1.00 equiv.), palmitoyl chloride (74.8 µL, 0.24 mmol, 1.20 equiv.), silver hexafluoroantimonate (82.5 mg, 0.24 mmol, 1.20 equiv.), reducing agent **RA 7** (126 mg, 0.44 mmol, 2.20 equiv.) and dichloromethane (2 mL). Purification by flash column chromatography (heptane/ethyl acetate 99:1 to 90:10, Rf = 0.40 in heptane/ethyl acetate 10:1) gave the title compound (d.r. >20:1, 28.1 mg, 44%) as a colourless oil.

^{1}H NMR (400 MHz, $CDCl_3$): δ 4.36 – 4.28 (m, 1H), 3.91 (td, *J* = 7.0, 3.3 Hz, 1H), 2.08 – 1.97 (m, 2H), 1.90 – 1.67 (m, 4H), 1.64 – 1.54 (m, 2H), 1.46 (dddd, *J* = 18.7, 10.8, 9.3, 4.3 Hz, 3H), 1.38 – 1.18 (m, 26H), 0.87 (t, *J* = 6.9 Hz, 3H) ppm.

^{13}C NMR (101 MHz, $CDCl_3$): δ 83.3, 75.3, 39.5, 37.1, 32.0, 32.0, 30.0, 29.9, 29.8 (6C), 29.78, 29.76, 29.5, 27.2, 25.8, 22.8, 19.0, 14.2 ppm.

HRMS (EI$^+$) exact mass calculated for $[M]^+$, $(C_{22}H_{42}O)^+$ required *m/z* 322.3230, found *m/z* 322.3221.

IR (neat) $ν_{max}$: 2920, 2851, 1465, 1274, 1260, 1096, 949, 850, 763, 747 cm^{-1}.

5q: 7-Undecyl-6-oxabicyclo[3.2.1]octane

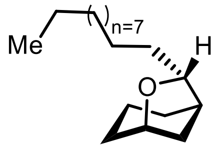

Synthesised following the general procedure, using cyclohexene (0.568 mL, 5.50 mmol, 1.00 equiv.), lauroyl chloride (1.363 mL, 6.60 mmol, 1.20 equiv.), silver hexafluoroantimonate (2.26 g, 6.60 mmol, 1.20 equiv.), reducing agent **RA 7** (3.40 g, 12.10 mmol, 2.20 equiv.) and dichloromethane (10 mL). Purification by flash column chromatography (heptane/ethyl acetate 99:1 to 90:10, Rf = 0.40 in heptane/ethyl acetate 10:1) gave the title compound (d.r. >20:1, 0.914 g, 63%) as a colourless oil.

^{1}H NMR (400 MHz, CDCl$_3$): δ 4.33 – 4.28 (m, 1H), 3.90 (td, *J* = 7.0, 3.3 Hz, 1H), 2.06 – 1.96 (m, 2H), 1.90 – 1.66 (m, 4H), 1.63 – 1.53 (m, 2H), 1.52 – 1.38 (m, 3H), 1.36 – 1.19 (m, 18H), 0.86 (t, *J* = 6.9 Hz, 3H) ppm.

^{13}C NMR (101 MHz, CDCl$_3$): δ 83.3, 75.3, 39.5, 37.1, 32.1, 32.0, 30.1, 29.9, 29.8 (2C), 29.8, 29.7, 29.5, 27.2, 25.8, 22.8, 19.0, 14.2 ppm.

HRMS (EI$^+$): exact mass calculated for [M]$^+$, ($C_{18}H_{34}O$)$^+$ required *m/z* 266.2604, found *m/z* 266.2605.

IR (neat) ν_{max}: 2924, 2853, 1738, 1457, 1365, 1229, 1097, 949, 895, 527 cm^{-1}.

5r: 7-Phenethyl-6-oxabicyclo[3.2.1]octane

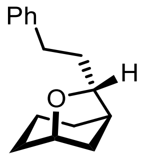

Synthesised following the general procedure, using cyclohexene (20.3 µL, 0.20 mmol, 1.00 equiv.), hydrocinnamoyl chloride (36.3 µL, 0.24 mmol, 1.20 equiv.), silver hexafluoroantimonate (82.5 mg, 0.24 mmol, 1.20 equiv.), reducing agent **RA 7** (126 mg, 0.44 mmol, 2.20 equiv.) and dichloromethane (2 mL). Purification by flash column chromatography (heptane/ethyl acetate 99:1 to 90:10, Rf = 0.28 in heptane/ethyl acetate 10:1) gave the title compound (d.r. >20:1, 57%) as a colourless oil.

^{1}H NMR (400 MHz, $CDCl_3$): δ 7.32 – 7.26 (m, 2H), 7.22 (dd, J = 10.5, 4.1 Hz, 2H), 7.20 – 7.13 (m, 1H), 4.39 – 4.33 (m, 1H), 4.02 – 3.94 (m, 1H), 2.87 – 2.78 (m, 1H), 2.65 (ddd, J = 13.8, 10.4, 6.0 Hz, 1H), 2.19 – 2.00 (m, 3H), 1.93 – 1.80 (m, 2H), 1.80 – 1.69 (m, 2H), 1.60 (d, J = 10.9 Hz, 1H), 1.57 – 1.47 (m, 2H), 1.43 – 1.30 (m, 1H) ppm.

^{13}C NMR (101 MHz, $CDCl_3$): δ 142.8, 128.9 (2C), 128.8 (2C), 126.2, 82.9, 75.8, 39.9, 37.6, 33.9, 32.3, 32.2, 26.2, 19.3 ppm.

HRMS (EI$^+$) exact mass calculated for $[M]^+$, $(C_{15}H_{20}O)^+$ required m/z 216.1509, found m/z 216.1515.

IR (neat) ν_{max}: 2931, 2853, 1495, 1260, 1096, 954, 894, 764, 698 cm^{-1}.

5s: 7-(Adamantan-1-yl)-6-oxabicyclo[3.2.1]octane

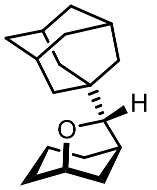

Synthesised following the general procedure, using cyclohexene (20.3 µL, 0.20 mmol, 1.00 equiv.), 1-adamantanecarbonyl chloride (49.2 mg, 0.24 mmol, 1.20 equiv.), silver hexafluoroantimonate (82.5 mg, 0.24 mmol, 1.20 equiv.), reducing agent **RA 7** (126 mg, 0.44 mmol, 2.20 equiv.) and dichloromethane (2 mL). Purification by flash column chromatography (heptane/ethyl acetate 99:1 to 90:10, Rf = 0.46 in heptane/ethyl acetate 10:1) gave the title compound (d.r. >20:1, 27.8 mg, 53%) as a colourless oil.

^{1}H NMR (400 MHz, CDCl$_3$): δ 4.34 (dd, *J* = 6.5, 4.7 Hz, 1H), 3.36 (d, *J* = 2.7 Hz, 1H), 2.13 (dd, *J* = 6.3, 3.0 Hz, 1H), 2.10 – 2.00 (m, 2H), 1.98 (dd, *J* = 7.3, 4.7 Hz, 3H), 1.95 – 1.88 (m, 1H), 1.86 – 1.75 (m, 4H), 1.71 (d, *J* = 8.7 Hz, 9H), 1.57 (ddd, *J* = 12.1, 6.6, 2.7 Hz, 1H), 1.50 (d, *J* = 10.9 Hz, 1H), 1.47 – 1.34 (m, 2H) ppm.

^{13}C NMR (101 MHz, CDCl$_3$): δ 93.1, 74.7, 40.7, 40.1 (3C), 37.3 (3C), 36.4, 36.3, 32.6, 28.6 (3C), 27.1, 18.2 ppm.

HRMS (EI$^+$) exact mass calculated for [M]$^+$, $(C_{17}H_{26}O)^+$ required *m/z* 246.1978, found *m/z* 246.1976.

IR (neat) ν_{max}: 2899, 2846, 1449, 1104, 1096, 992, 894, 744 cm^{-1}.

5t: 7-Isopropyl-6-oxabicyclo[3.2.1]octane

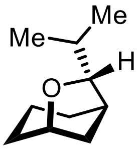

Synthesised following the general procedure, using cyclohexene (20.3 µL, 0.20 mmol, 1.00 equiv.), isobutyryl chloride (25.7 µL, 0.24 mmol, 1.20 equiv.), silver hexafluoroantimonate (82.5 mg, 0.24 mmol, 1.20 equiv.), reducing agent **RA 7** (126 mg, 0.44 mmol, 2.20 equiv.) and dichloromethane (2 mL). Purification by flash column chromatography (heptane/ethyl acetate 99:1 to 90:10, Rf = 0.33 in heptane/ethyl acetate 10:1) gave the title compound (d.r. >20:1, NMR 63%, determined using mesitylene as an internal standard) as a colourless oil.

^{1}H NMR (400 MHz, $CDCl_3$): δ 4.38 – 4.30 (m, 1H), 3.46 (dd, *J* = 10.3, 3.3 Hz, 1H), 2.16 – 2.10 (m, 1H), 2.07 – 1.95 (m, 2H), 1.89 – 1.78 (m, 1H), 1.74 (ddd, *J* = 8.5, 7.4, 2.8 Hz, 2H), 1.59 (d, *J* = 7.2 Hz, 1H), 1.55 – 1.42 (m, 2H), 1.39 – 1.30 (m, 1H), 1.07 (d, *J* = 6.5 Hz, 3H), 0.86 (d, *J* = 6.6 Hz, 3H) ppm.

^{13}C NMR (101 MHz, $CDCl_3$): δ 89.7, 75.4, 39.7, 36.2, 32.2, 28.1, 25.8, 20.9, 19.7, 19.4 ppm.

HRMS (EI$^+$): exact mass calculated for $[M]^+$, $(C_{10}H_{18}O)^+$ required *m/z* 154.1352, found *m/z* 154.1353.

IR (neat) ν_{max}: 2956, 2931, 2924, 1736, 742 cm^{-1}.

5u: 7-Cyclopropyl-6-oxabicyclo[3.2.1]octane

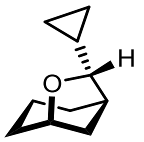

Synthesised following the general procedure, using cyclohexene (20.3 µL, 0.20 mmol, 1.00 equiv.), cyclopropanecarbonyl chloride (22.3 µL, 0.24 mmol, 1.20 equiv.), silver hexafluoroantimonate (82.5 mg, 0.24 mmol, 1.20 equiv.), reducing agent **RA 7** (126 mg, 0.44 mmol, 2.20 equiv.) and dichloromethane (2 mL). Purification by flash column chromatography (heptane/ethyl acetate 99:1 to 90:10, Rf = 0.21 in heptane/ethyl acetate 10:1) gave the title compound (d.r. >20:1, NMR 66%, determined using mesitylene as an internal standard) as a colourless oil.

^{1}H NMR (400 MHz, $CDCl_3$): δ 4.36 – 4.32 (m, 1H), 3.04 (dd, *J* = 9.8, 3.5 Hz, 1H), 2.14 (dd, *J* = 6.4, 3.3 Hz, 1H), 2.01 – 1.88 (m, 3H), 1.86 – 1.76 (m, 1H), 1.62 – 1.55 (m, 3H), 1.40 (dd, *J* = 11.8, 5.4 Hz, 1H), 1.23 – 1.16 (m, 1H), 0.71 – 0.61 (m, 1H), 0.57 – 0.48 (m, 1H), 0.43 – 0.34 (m, 1H), 0.09 (dq, *J* = 9.3, 4.9 Hz, 1H) ppm.

^{13}C NMR (101 MHz, $CDCl_3$): δ 89.5, 75.7, 39.3, 37.8, 32.0, 26.5, 19.0, 9.6, 4.1, 1.7 ppm.

HRMS (EI$^+$): exact mass calculated for $[M]^+$, $(C_{10}H_{16}O)^+$ required *m/z* 152.1196, found *m/z* 152.1187.

IR (neat) ν_{max}: 2970, 2945, 1738, 1365, 1228, 1206, 895, 738 cm^{-1}.

5v: 7-Cyclohexyl-6-oxabicyclo[3.2.1]octane

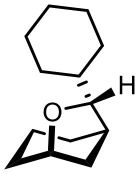

Synthesised following the general procedure, using cyclohexene (20.3 µL, 0.20 mmol, 1.00 equiv.), cyclohexanecarbonyl chloride (32.9 µL, 0.24 mmol, 1.20 equiv.), silver hexafluoroantimonate (82.5 mg, 0.24 mmol, 1.20 equiv.), reducing agent **RA 7** (126 mg, 0.44 mmol, 2.20 equiv.) and dichloromethane (2 mL). Purification by flash column chromatography (heptane/ethyl acetate 99:1 to 90:10, Rf = 0.33 in heptane/ethyl acetate 10:1) gave the title compound (d.r. >20:1, NMR 70%, determined using mesitylene as an internal standard) as a colourless oil.

^{1}H NMR (400 MHz, $CDCl_3$): δ 4.35 – 4.30 (m, 1H), 3.53 (dd, *J* = 10.2, 3.3 Hz, 1H), 2.16 – 2.09 (m, 2H), 2.00 (dtt, *J* = 13.1, 4.5, 2.1 Hz, 1H), 1.93 – 1.80 (m, 1H), 1.78 – 1.64 (m, 6H), 1.61 – 1.55 (m, 2H), 1.54 – 1.41 (m, 2H), 1.31 (dddd, *J* = 12.6, 9.0, 6.8, 4.2 Hz, 3H), 1.18 (ddd, *J* = 12.5, 7.6, 3.1 Hz, 1H), 1.05 – 0.94 (m, 1H), 0.94 – 0.84 (m, 1H) ppm.

^{13}C NMR (151 MHz, $CDCl_3$): δ 88.1, 75.1, 39.5, 37.8, 35.7, 32.3, 31.3, 29.9, 26.7, 26.1, 25.9, 25.9, 19.4 ppm.

HRMS (EI$^+$) exact mass calculated for $[M]^+$, $(C_{13}H_{22}O)^+$ required *m/z* 194.1665, found *m/z* 194.1663.

IR (neat) ν_{max}: 2927, 2854, 1724, 1449, 1365, 1216, 1120, 895, 733 cm^{-1}.

5w: 7-(3-Chloropropyl)-6-oxabicyclo[3.2.1]octane

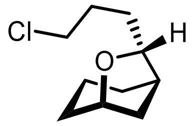

Synthesised following the general procedure, using cyclohexene (20.3 µL, 0.20 mmol, 1.00 equiv.), 4-chlorobutyryl chloride (27.6 µL, 0.24 mmol, 1.20 equiv.), silver hexafluoroantimonate (82.5 mg, 0.24 mmol, 1.20 equiv.), reducing agent **RA 7** (126 mg, 0.44 mmol, 2.20 equiv.) and dichloromethane (2 mL). Purification by flash column chromatography (heptane/ethyl acetate 99:1 to 90:10, Rf = 0.23 in heptane/ethyl acetate 10:1) gave the title compound (d.r. >20:1, NMR 71%, determined using mesitylene as an internal standard) as a colourless oil.

^{1}H NMR (400 MHz, CDCl$_3$): δ 4.36 – 4.30 (m, 1H), 3.97 – 3.90 (m, 1H), 3.61 (qt, *J* = 10.7, 6.2 Hz, 2H), 2.07 (d, *J* = 3.0 Hz, 1H), 2.01 (dddd, *J* = 10.1, 8.3, 5.4, 2.3 Hz, 2H), 1.91 – 1.69 (m, 6H), 1.63 (d, *J* = 14.4 Hz, 1H), 1.52 (ddd, *J* = 14.6, 9.2, 5.0 Hz, 2H), 1.40 – 1.30 (m, 1H) ppm.

^{13}C NMR (101 MHz, CDCl$_3$): δ 82.4, 75.6, 45.4, 39.5, 37.4, 31.9, 30.5, 27.3, 25.8, 18.9 ppm.

HRMS (EI$^+$) exact mass calculated for [M]$^+$, $(C_{10}H_{17}ClO)^+$ required *m/z* 188.0962, found *m/z* 188.0960.

IR (neat) ν_{max}: 2931, 2855, 1738, 1457, 1365, 1204, 1042, 763 cm^{-1}.

5x: 7-(3-Bromopropyl)-6-oxabicyclo[3.2.1]octane

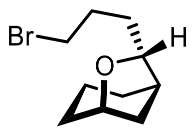

Synthesised following the general procedure, using cyclohexene (20.3 µL, 0.20 mmol, 1.00 equiv.), 4-bromobutyryl chloride (27.8 µL, 0.24 mmol, 1.20 equiv.), silver hexafluoroantimonate (82.5 mg, 0.24 mmol, 1.20 equiv.), reducing agent **RA 7** (126 mg, 0.44 mmol, 2.20 equiv.) and dichloromethane (2 mL). Purification by flash column chromatography (heptane/ethyl acetate 99:1 to 90:10, Rf = 0.27 in heptane/ethyl acetate 10:1) gave the title compound (d.r. >20:1, NMR 53%, determined by mesitylene as internal standard) as a colourless oil.

^{1}H NMR (400 MHz, $CDCl_3$): δ 4.36 – 4.31 (m, 1H), 3.97 – 3.90 (m, 1H), 3.56 – 3.43 (m, 2H), 2.10 – 2.05 (m, 2H), 2.04 – 1.98 (m, 1H), 1.97 – 1.85 (m, 2H), 1.85 – 1.69 (m, 4H), 1.60 (d, *J* = 10.9 Hz, 1H), 1.57 – 1.47 (m, 2H), 1.39 – 1.30 (m, 1H) ppm.

^{13}C NMR (101 MHz, $CDCl_3$): δ 82.3, 75.6, 39.5, 37.4, 34.3, 31.9, 30.7, 28.6, 25.8, 18.9 ppm.

HRMS (EI$^+$): exact mass calculated for $[M]^+$, $(C_{10}H_{17}{}^{79}BrO)^+$ required *m/z* 232.0457, found *m/z* 232.0457.

IR (neat) ν_{max}: 2935, 2856, 1738, 1455, 1318, 1229, 1097, 953, 894, 855 cm^{-1}.

5y: 3-Phenyl-2-oxabicyclo[2.2.1]heptane

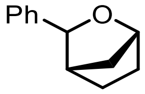

Synthesised following the general procedure, using cyclopentene (18.0 µL, 0.20 mmol, 1.00 equiv.), benzoyl chloride (28.4 µL, 0.24 mmol, 1.20 equiv.), silver hexafluoroantimonate (82.5 mg, 0.24 mmol, 1.20 equiv.), reducing agent **RA 7** (114 mg, 0.44 mmol, 2.20 equiv.) and dichloromethane (2 mL). Purification by flash column chromatography (heptane/ethyl acetate 99:1 to 90:10, Rf = 0.60 in heptane/ethyl acetate 10:1) gave the title compound (d.r. >20:1, 20.2 mg, 58%) as a colourless oil.

^{1}H NMR (400 MHz, CDCl$_3$): δ 7.36 – 7.31 (m, 4H), 7.27 – 7.21 (m, 1H), 5.09 (s, 1H), 4.51 (s, 1H), 2.63 (s, 1H), 2.12 – 2.07 (m, 1H), 1.75 – 1.63 (m, 2H), 1.54 (tdd, *J* = 12.9, 4.3, 2.1 Hz, 1H), 1.33 (dddd, *J* = 8.6, 4.6, 3.4, 1.8 Hz, 1H), 1.24 – 1.15 (m, 1H) ppm.

^{13}C NMR (101 MHz, CDCl$_3$): δ 141.6, 128.1 (2C), 126.7, 125.6 (2C), 83.1, 78.5, 43.0, 39.5, 31.3, 21.3 ppm.

HRMS (EI$^+$): exact mass calculated for $[M]^+$, $(C_{12}H_{14}O)^+$ required *m/z* 174.1039, found *m/z* 174.1035.

IR (neat) ν_{max}: 2969, 2871, 1738, 1596, 1467, 1305, 1275, 1049, 901, 737 cm^{-1}.

5z: 3-(2-Iodophenyl)-2-oxabicyclo[2.2.1]heptane

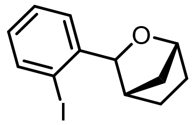

Synthesised following the general procedure, using cyclopentene (45.1 µL, 0.50 mmol, 1.00 equiv.), 2 iodobenzoyl chloride (84.4 µL, 0.60 mmol, 1.20 equiv.), silver hexafluoroantimonate (210.0 mg, 0.60 mmol, 1.20 equiv.), reducing agent **RA 7** (309 mg, 1.10 mmol, 2.20 equiv.) and dichloromethane (4 mL). Purification by flash column chromatography (heptane/ethyl acetate 99:1 to 90:10, Rf = 0.65 in heptane/ethyl acetate 10:1) gave the title compound (d.r. >20:1, 68.4 mg, 46%) as a colourless oil.

^{1}H NMR (400 MHz, CDCl$_3$): δ 7.81 (dd, *J* = 7.8, 1.1 Hz, 1H), 7.56 (dd, *J* = 7.7, 1.20Hz, 1H), 7.36 (td, *J* = 7.7, 0.8 Hz, 1H), 6.97 (ddd, *J* = 7.4, 1.7, 0.8 Hz, 1H), 5.03 (s, 1H), 4.58 (s, 1H), 3.13 (d, *J* = 1.5 Hz, 1H), 2.17 – 2.09 (m, 1H), 1.85 – 1.76 (m, 1H), 1.68 – 1.64 (m, 1H), 1.62 – 1.52 (m, 1H), 1.34 (ttd, *J* = 12.2, 4.7, 1.7 Hz, 1H), 1.14 – 1.05 (m, 1H) ppm.

^{13}C NMR (101 MHz, CDCl$_3$): δ 142.7, 139.4, 128.9, 127.9, 127.5, 96.8, 87.2, 79.0, 40.1, 39.6, 31.3, 21.20ppm.

HRMS (ESI$^+$): exact mass calculated for [M]$^+$ ($C_{12}H_{13}IO$) requires *m/z* 300.0006, found *m/z* 300.0002.

IR (neat) ν_{max}: 2971, 2870, 1738, 1434, 1365, 1228, 1047, 963, 848, 662 cm^{-1}.

5aa: 3-Undecyl-2-oxabicyclo[2.2.1]heptane

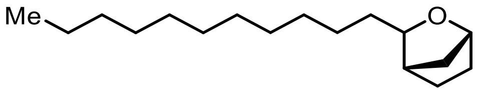

Synthesised following the general procedure, using cyclopentene (45.1 µL, 0.50 mmol, 1.00 equiv.), lauroyl chloride (142 µL, 0.60 mmol, 1.20 equiv.), silver hexafluoroantimonate (210.0 mg, 0.6 mmol, 1.20 equiv.), reducing agent **RA 7** (309 mg, 1.1 mmol, 2.20 equiv.) and dichloromethane (4 mL). Purification by flash column chromatography (heptane/ethyl acetate 99:1 to 90:10, Rf = 0.65 in heptane/ethyl acetate 10:1) gave the title compound (d.r. >20:1, 69.8 mg, 55%) as a colourless oil.

^{1}H NMR (400 MHz, $CDCl_3$): δ 4.25 (s, 1H), 3.79 (dd, *J* = 8.7, 4.1 Hz, 1H), 2.30 (s, 1H), 1.85 – 1.79 (m, 1H), 1.72 – 1.64 (m, 1H), 1.63 – 1.45 (m, 4H), 1.44 – 1.34 (m, 2H), 1.24 (s, 18H), 0.86 (t, *J* = 6.8 Hz, 3H) ppm.

^{13}C NMR (101 MHz, $CDCl_3$): δ 81.5, 77.4, 40.1, 39.2, 32.4, 32.0, 31.5, 30.0, 29.8, 29.8 (2C), 29.7, 29.5, 26.7, 22.8, 21.1, 14.2 ppm.

HRMS (EI$^+$) exact mass calculated for $[M]^+$ ($C_{17}H_{32}O^+$) requires *m/z* 252.2448, found *m/z* 252.2438.

IR (neat) ν_{max}: 2969, 2924, 2853, 1738, 1455, 1365, 1228, 1141, 932 cm^{-1}.

5ab: 13-Phenyl-12-oxabicyclo[9.2.1]tetradecane

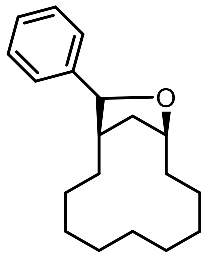

Synthesised following the general procedure, using cyclododecene (20.3 µL, 0.20 mmol, 1.00 equiv.), silver hexafluoroantimonate (75.6 mg, 0.24 mmol, 1.20 equiv.), benzoyl chloride (42.1 mg, 0.24 mmol, 1.20 equiv.), reducing agent **RA 7** (126 mg, 0.44 mmol, 2.20 equiv.) and dichloromethane (2 mL). Purification by flash column chromatography (heptane/ethyl acetate 99:1 to 90:10, Rf = 0.32 in heptane/ethyl acetate 10:1) gave the title compound (d.r. >20:1, 24.7 mg, 46%) as a colourless oil.

^{1}H NMR (400 MHz, $CDCl_3$): δ 7.30 (t, *J* = 7.3 Hz, 2H), 7.24 – 7.16 (m, 3H), 5.17 (d, *J* = 7.6 Hz, 1H), 4.57 – 4.49 (m, 1H), 2.73 (d, *J* = 4.4 Hz, 1H), 2.05 – 1.94 (m, 1H), 1.72 (d, *J* = 15.7 Hz, 3H), 1.55 – 1.27 (m, 13H), 1.19 (s, 2H), 0.78 (dt, *J* = 15.7, 7.9 Hz, 1H) ppm.

^{13}C NMR (101 MHz, $CDCl_3$): δ 142.0, 127.8 (2C), 126.8, 126.4 (2C), 82.7, 78.4, 40.5, 32.4, 26.9, 26.2, 26.0, 25.1, 23.9, 23.7 (2C), 21.6 (2C) ppm.

HRMS (EI$^+$): exact mass calculated for $[M]^+$ $(C_{19}H_{28}O)^+$ requires *m/z* 272.2135, found *m/z* 272.2129.

IR (neat) ν_{max}: 3025, 2969, 2868, 1738, 1470, 1365, 1228, 1041, 700 cm^{-1}.

5ac: 2-phenyldecahydro-1,4:6,9-dimethanobenzo[d]oxepine

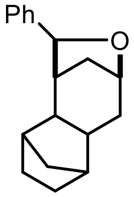

Synthesised following the general procedure, using tricyclo[6.2.1.02,7]undeca-4-ene (48.3 µL, 0.30 mmol, 1.00 equiv.), silver hexafluoroantimonate (113 mg, 0.36 mmol, 1.20 equiv.), benzoyl chloride (41.8 ul, 0.36 mmol, 1.20 equiv.), reducing agent **RA 7** (126 mg, 0.44 mmol, 2.20 equiv.) and dichloromethane (2 mL). Purification by flash column chromatography (heptane/ethyl acetate 99:1 to 90:10, Rf = 0.46 in heptane/ethyl acetate 10:1) gave the title compound (d.r. >20:1, 39.0 mg, 51%) as a colourless oil.

^{1}H NMR (400 MHz, $CDCl_3$): δ 7.36 – 7.30 (m, 4H), 7.22 (ddd, *J* = 11.0, 5.6, 2.9 Hz, 1H), 4.75 (s, 1H), 3.66 (d, *J* = 6.6 Hz, 1H), 2.25 – 2.02 (m, 4H), 1.88 (s, 1H), 1.83 (dd, *J* = 12.2, 3.4 Hz, 1H), 1.76 – 1.70 (m, 1H), 1.51 (dt, *J* = 11.8, 5.6 Hz, 3H), 1.42 – 1.34 (m, 1H), 1.28 (td, *J* = 11.9, 3.3 Hz, 2H), 1.22 – 1.14 (m, 1H), 1.07 (ddd, *J* = 12.2, 9.1, 5.1 Hz, 1H) ppm.

^{13}C NMR (101 MHz, $CDCl_3$): δ 143.1, 128.1 (2C), 126.7, 125.7 (2C), 81.4, 81.3, 49.1, 43.5, 39.8, 39.2, 36.4, 35.0, 31.0, 29.8, 24.6, 23.0 ppm.

HRMS (EI$^+$) exact mass calculated for $[M]^+$ $(C_{18}H_{22}O)^+$ requires *m/z* 254.1665, found *m/z* 254.1660.

IR (neat) ν_{max}: 3035, 2859, 1736, 1463, 1357, 1215, 1037, 698 cm^{-1}.

6.3: Synthesis of spirocyclic THF products (7a–7h)

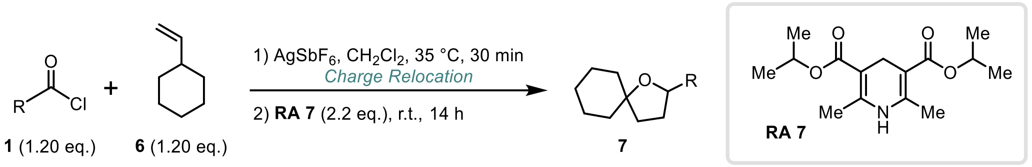

An oven-dried vial was flushed with argon and charged with a magnetic stir bar. After that, vinyl cyclohexane (0.20 mmol, 1.00 equiv.) and acyl chloride (0.24 mmol, 1.20 equiv.) were added, followed by dichloromethane (2 mL). The resulting solution was treated with silver hexafluoroantimonate (0.24 mmol, 1.20 equiv.), and the vial was immediately heated in a sand bath at 35 °C. After 30 min, stirring was stopped and the supernatant was transferred by syringe to a separate vial. To this, reducing agent **RA 7** (2.20 equiv.) was added. The vial was placed at ambient temperature (22–25 °C) and the solution was stirred at that temperature for 14 h. The reaction mixture was then treated with a saturated aqueous solution of $NaHCO_3$, extracted with DCM (3 × 5 mL), dried over anhydrous magnesium sulfate, filtered, and the filtrate was concentrated under reduced pressure. The resulting crude material was purified by flash column chromatography on silica gel (heptane/ethyl acetate) to give the title compounds.

7a: 2-Phenyl-1-oxaspiro[4.5]decane

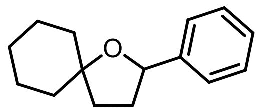

Synthesised following the general procedure, using vinyl cyclohexane (27.4 µL, 0.20 mmol, 1.00 equiv.), benzoyl chloride (27.9 µL, 0.24 mmol, 1.20 equiv.), silver hexafluoroantimonate (82.5 mg, 0.24 mmol, 1.20 equiv.), reducing agent **RA 7** (114 mg, 0.44 mmol, 2.20 equiv.) and dichloromethane (2 mL). Purification by flash column chromatography (heptane/ethyl acetate 99:1 to 90:10, Rf = 0.40 in heptane/ethyl acetate 10:1) gave the title compound (23 mg, 54%) as a colourless oil.

^{1}H NMR (400 MHz, CDCl$_3$): δ 7.40 – 7.35 (m, 2H), 7.32 (dd, *J* = 10.2, 4.8 Hz, 3H), 7.26 – 7.21 (m, 1H), 4.97 (dd, *J* = 7.9, 6.1 Hz, 1H), 2.33 – 2.27 (m, 1H), 1.96 – 1.86 (m, 2H), 1.85 – 1.70 (m, 2H), 1.69 – 1.57 (m, 2H), 1.60 – 1.50 (m, 3H), 1.42 – 1.28 (m, 3H) ppm.

^{13}C NMR (101 MHz, CDCl$_3$): δ 144.0, 128.3 (2C), 127.1, 125.9 (2C), 83.4, 80.0, 38.4, 38.0, 36.8, 35.3, 25.9, 24.2, 23.9 ppm.

HRMS (EI$^+$): exact mass calculated for [M]$^+$, $(C_{15}H_{20}O)^+$ required *m/z* 216.1509, found *m/z* 216.1507.

IR (neat) ν_{max}: 2931, 1707, 1271, 1173, 1025, 961, 848, 708, 686 cm^{-1}.

7b: 2-(2-Iodophenyl)-1-oxaspiro[4.5]decane

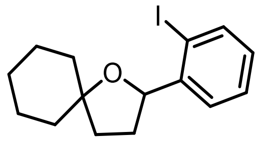

Synthesised following the general procedure, using vinyl cyclohexane (69.8 µL, 0.50 mmol, 1.00 equiv.), 2-iodobenzoyl chloride (84.4 µL, 0.60 mmol, 1.20 equiv.), silver hexafluoroantimonate (210 mg, 0.60 mmol, 1.20 equiv.), reducing agent **RA 7** (309 mg, 1.10 mmol, 2.20 equiv.) and dichloromethane (4 mL). Purification by flash column chromatography (heptane/ethyl acetate 99:1 to 90:10, Rf = 0.78 in heptane/ethyl acetate 10:1) gave the title compound (71.6 mg, 42%) as a colourless oil.

^{1}H NMR (400 MHz, $CDCl_3$): δ 7.77 (dd, *J* = 7.9, 1.4 Hz, 1H), 7.57 (dd, *J* = 7.8, 1.9 Hz, 1H), 7.34 (td, *J* = 7.5, 1.4 Hz, 1H), 6.93 (td, *J* = 7.6, 1.9 Hz, 1H), 5.07 (dd, *J* = 8.3, 6.4 Hz, 1H), 2.59 (dq, *J* = 12.6, 6.5 Hz, 1H), 1.88 – 1.72 (m, 5H), 1.70 – 1.57 (m, 4H), 1.51 – 1.39 (m, 4H) ppm.

^{13}C NMR (101 MHz, $CDCl_3$): δ 146.2, 139.1, 128.8, 128.5, 126.8, 96.8, 83.9, 83.3, 38.4, 37.6, 36.5, 33.7, 25.9, 24.2, 23.8 ppm.

HRMS (EI$^+$): exact mass calculated for $[M]^+$, $(C_{15}H_{19}IO)^+$ required *m/z* 342.0475, found *m/z* 342.0467.

IR (neat) ν_{max}: 2969, 2854, 1459, 1365, 1228, 1112, 1053, 941, 752 cm^{-1}.

7c: 2-(4-Methoxyphenyl)-1-oxaspiro[4.5]decane

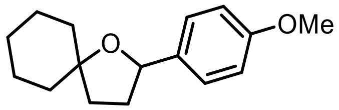

Synthesised following the general procedure, using vinyl cyclohexane (69.8 µL, 0.50 mmol, 1.00 equiv.), 4-methoxy benzoyl chloride (82.9 µL, 0.60 mmol, 1.20 equiv.), silver hexafluoroantimonate (210 mg, 0.60 mmol, 1.20 equiv.), reducing agent **RA 7** (309 mg, 1.10 mmol, 2.20 equiv.) and dichloromethane (2 mL). Purification by flash column chromatography (heptane/ethyl acetate 99:1 to 90:10, Rf = 0.38 in heptane/ethyl acetate 10:1) gave the title compound (71.3 mg, 58%) as a colourless oil.

^{1}H NMR (400 MHz, CDCl$_3$): δ 7.29 (d, *J* = 8.5 Hz, 2H), 6.87 (d, *J* = 8.7 Hz, 2H), 4.92 (t, *J* = 6.8 Hz, 1H), 3.79 (s, 3H), 2.29 – 2.18 (m, 1H), 1.94 – 1.79 (m, 3H), 1.78 – 1.72 (m, 2H), 1.69 (dd, *J* = 8.4, 3.5 Hz, 1H), 1.62 (dt, *J* = 12.8, 7.3 Hz, 3H), 1.43 (dd, *J* = 14.5, 8.2 Hz, 4H) ppm.

^{13}C NMR (101 MHz, CDCl$_3$): δ 158.9, 136.0, 127.2 (2C), 113.7, (2C), 83.1, 79.7, 55.4, 38.4, 38.0, 36.8, 35.3, 25.8, 24.2, 23.9 ppm.

HRMS (EI$^+$) exact mass calculated for [M]$^+$, $(C_{16}H_{22}O_2)^+$ required *m/z* 246.1614, found *m/z* 246.1616.

IR (neat) ν_{max}: 2928, 1738, 1511, 1365, 1243, 1170, 1067, 951, 828 cm^{-1}.

7d: 2-(Adamantan-1-yl)-1-oxaspiro[4.5]decane

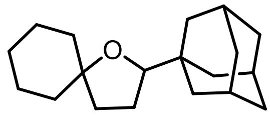

Synthesised following the general procedure, using vinyl cyclohexane (41.9 µL, 0.30 mmol, 1.00 equiv.), 1-adamantanecarbonyl chloride (69.6 µL, 0.36 mmol, 1.20 equiv.), silver hexafluoroantimonate (126 mg, 0.36 mmol, 1.20 equiv.), reducing agent **RA 7** (186 mg, 0.66 mmol, 2.20 equiv.) and dichloromethane (3 mL). Purification by flash column chromatography (heptane/ethyl acetate 99:1 to 90:10, Rf = 0.82 in heptane/ethyl acetate 10:1) gave the title compound (34.6 mg, 42%) as a colourless oil.

^{1}H NMR (400 MHz, CDCl$_3$): δ 3.44 (dd, *J* = 8.4, 6.3 Hz, 1H), 1.94 (d, *J* = 2.6 Hz, 3H), 1.74 – 1.64 (m, 9H), 1.62 (t, *J* = 3.7 Hz, 2H), 1.60 – 1.55 (m, 3H), 1.52 – 1.44 (m, 6H), 1.43 – 1.24 (m, 6H) ppm.

^{13}C NMR (101 MHz, CDCl$_3$): δ 86.5, 81.6, 38.7 (3C), 38.0, 37.8 (3C), 37.6, 36.1, 35.4, 28.5, 26.0 (3C), 24.8, 24.2, 24.1 ppm.

HRMS (EI$^+$): exact mass calculated for [M]$^+$, ($C_{19}H_{30}O$)$^+$ required *m/z* 274.2291, found *m/z* 274.2291.

IR (neat) ν_{max}: 2925, 2847, 1447, 1253, 1216, 1077, 969 573 cm^{-1}.

7e: 2-Cyclohexyl-1-oxaspiro[4.5]decane

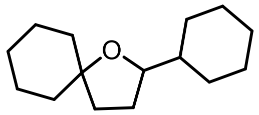

Synthesised following the general procedure, using vinyl cyclohexane (41.9 µL, 0.30 mmol, 1.00 equiv.), cyclohexanecarbonyl chloride (49.4 µL, 0.36 mmol, 1.20 equiv.), silver hexafluoroantimonate (126 mg, 0.36 mmol, 1.20 equiv.), reducing agent **RA 7** (186 mg, 0.66 mmol, 2.20 equiv.) and dichloromethane (3 mL). Purification by flash column chromatography (heptane/ethyl acetate 99:1 to 90:10, Rf = 0.82 in heptane/ethyl acetate 10:1) gave the title compound (34.6 mg, 54%) as a colourless oil.

^{1}H NMR (400 MHz, $CDCl_3$): δ 3.62 (dd, *J* = 13.6, 6.8 Hz, 1H), 1.95 – 1.82 (m, 2H), 1.74 – 1.57 (m, 10H), 1.49 (dd, *J* = 9.7, 6.2 Hz, 3H), 1.38 – 1.29 (m, 4H), 1.27 – 1.08 (m, 4H), 1.00 – 0.86 (m, 2H) ppm.

^{13}C NMR (101 MHz, $CDCl_3$): δ 82.6, 81.9, 43.5, 38.4, 37.8, 36.0, 30.2, 28.8, 28.7, 26.8, 26.3, 26.2, 25.9, 24.2, 24.0 ppm.

HRMS (EI$^+$): exact mass calculated for $[M]^+$, $(C_{15}H_{26}O)^+$ required *m/z* 222.1978, found *m/z* 222.1978.

IR (neat) ν_{max}: 2924, 2852, 1738, 1448, 1365, 1228, 1070, 949 cm^{-1}.

7f: 2-Undecyl-1-oxaspiro[4.5]decane

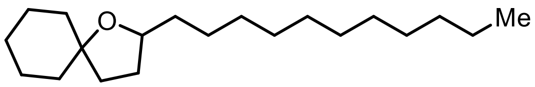

Synthesised following the general procedure, using vinyl cyclohexane (140.0 µL, 1.0 mmol, 1.00 equiv.), lauroyl chloride (283.0 µL, 1.20 mmol, 1.20 equiv.), silver hexafluoroantimonate (412 mg, 1.20 mmol, 1.20 equiv.), reducing agent **RA 7** (619 mg, 2.20 mmol, 2.20 equiv.) and dichloromethane (10 mL). Purification by flash column chromatography (heptane/ethyl acetate 99:1 to 90:10, Rf = 0.82 in heptane/ethyl acetate 10:1) gave the title compound (239.2 mg, 81%) as a colourless oil.

^{1}H NMR (400 MHz, $CDCl_3$): δ 3.82 (dq, *J* = 7.9, 6.1 Hz, 1H), 1.87 (dddd, *J* = 12.1, 8.0, 5.9, 4.4 Hz, 1H), 1.69 – 1.51 (m, 5H), 1.49 – 1.37 (m, 5H), 1.37 – 1.10 (m, 23H), 0.81 (t, *J* = 6.9 Hz, 3H) ppm.

^{13}C NMR (101 MHz, $CDCl_3$): δ 82.2, 78.1, 38.8, 37.7, 36.7, 36.0, 32.0, 31.5, 29.9, 29.8, 29.7, 29.7 (2C), 29.5, 26.3, 25.9, 24.3, 23.9, 22.8, 14.2 ppm.

HRMS (EI$^+$): exact mass calculated for $[M]^+$, $(C_{20}H_{38}O)^+$ required *m/z* 294.2917, found *m/z* 294.2912.

IR (neat) ν_{max}: 2922, 2852, 1738, 1448, 1365, 1228, 1081, 948, 896, 721 cm^{-1}.

7g: 2-(5-Bromopentyl)-1-oxaspiro[4.5]decane

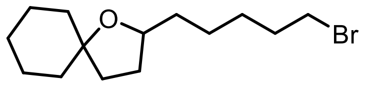

Synthesised following the general procedure, using vinyl cyclohexane (69.8 µL, 0.50 mmol, 1.00 equiv.), 6-bromohexanoyl chloride (93.7 µL, 0.60 mmol, 1.20 equiv.), silver hexafluoroantimonate (210.0 mg, 0.60 mmol, 1.20 equiv.), reducing agent **RA 7** (309 mg, 1.10 mmol, 2.20 equiv.) and dichloromethane (4 mL). Purification by flash column chromatography (heptane/ethyl acetate 99:1 to 90:10, Rf = 0.65 in heptane/ethyl acetate 10:1) gave the title compound (91.6 mg, 64%) as a colourless oil.

^{1}H NMR (400 MHz, $CDCl_3$): δ 3.88 (tt, *J* = 12.2, 6.1 Hz, 1H), 3.38 (t, *J* = 6.9 Hz, 2H), 1.93 (dddd, *J* = 12.1, 7.9, 6.0, 4.5 Hz, 1H), 1.88 – 1.80 (m, 2H), 1.73 – 1.55 (m, 5H), 1.46 (qdd, *J* = 11.2, 8.8, 3.0 Hz, 10H), 1.36 – 1.26 (m, 4H) ppm.

^{13}C NMR (101 MHz, $CDCl_3$): δ 82.3, 77.8, 38.7, 37.7, 36.4, 35.9, 34.0, 32.8, 31.4, 28.4, 25.8, 25.4, 24.2, 23.9 ppm.

HRMS (EI$^+$): exact mass calculated for $[M]^+$, $(C_{14}H_{25}{}^{79}BrO)^+$ required *m/z* 288.1083, found *m/z* 288.1098.

IR (neat) ν_{max}: 2927, 2855, 1738, 1447, 1365, 1268, 1081, 949, 895 cm^{-1}.

7h: 2-Phenethyl-1-oxaspiro[4.5]decane

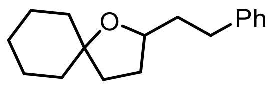

Synthesised following the general procedure, using vinyl cyclohexane (41.9 µL, 0.30 mmol, 1.00 equiv.), hydrocinnamoyl chloride (54.5 µL, 0.36 mmol, 1.20 equiv.), silver hexafluoroantimonate (126 mg, 0.36 mmol, 1.20 equiv.), reducing agent **RA 7** (186 mg, 0.66 mmol, 2.20 equiv.) and dichloromethane (3 mL). Purification by flash column chromatography (heptane/ethyl acetate 99:1 to 90:10, Rf = 0.60 in heptane/ethyl acetate 10:1) gave the title compound (40.0 mg, 55%) as a colourless oil.

^{1}H NMR (400 MHz, $CDCl_3$): δ 7.30 – 7.25 (m, 2H), 7.23 – 7.14 (m, 3H), 3.95 (dq, *J* = 12.7, 6.3 Hz, 1H), 2.70 (dddd, *J* = 33.1, 13.8, 10.1, 6.0 Hz, 2H), 2.04 – 1.95 (m, 1H), 1.95 – 1.86 (m, 1H), 1.81 – 1.71 (m, 3H), 1.67 (dd, *J* = 8.0, 4.2 Hz, 2H), 1.59 – 1.54 (m, 2H), 1.53 – 1.47 (m, 3H), 1.42 – 1.32 (m, 4H) ppm.

^{13}C NMR (101 MHz, $CDCl_3$): δ 142.6, 128.5 (2C), 128.3, 125.7 (2C), 82.5, 77.3, 38.8, 38.4, 37.8, 36.0, 32.6, 31.4, 25.9, 24.3, 23.9 ppm.

HRMS (EI$^+$): exact mass calculated for $[M]^+$, $(C_{17}H_{24}O)^+$ required *m/z* 244.1822, found *m/z* 244.1820.

IR (neat) ν_{max}: 3025, 2927, 2855, 1603, 1495, 1048, 974, 745, 698 cm^{-1}.

7. Isotope labelling experiments.

7.1: Synthesis of deuterated reducing agent **RA 7-*d*₂.**

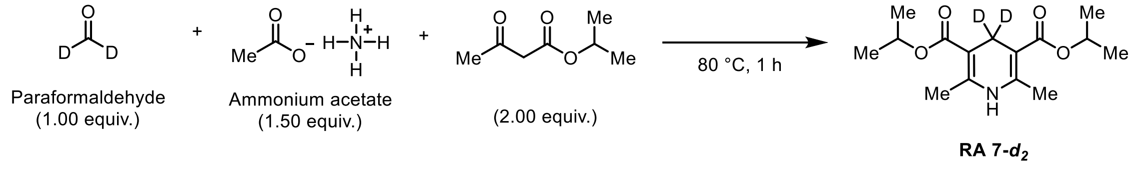

A Schlenk flask was flushed with argon three times and charged with paraformaldehyde (10.0 mmol, 320 mg, 1.00 equiv.), ammonium acetate (15.0 mmol, 1.15 g, 1.50 equiv.) and isopropyl 3-oxobutanoate (20.0 mmol, 3.27 g, 2.00 equiv.). After stirring at 80 °C for 1 h, ice-cold water was added to the reaction mixture and the crude product was filtered. The precipitate was recrystallised from methanol, yielding a yellow solid **RA 7-**d_2 (1.86 g, 66%).

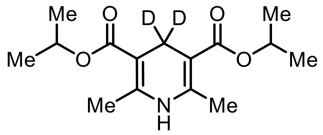

^{1}H NMR (400 MHz, $CDCl_3$): δ 5.17 (m, 1H), 5.04 (hept, *J* = 6.2 Hz, 2H), 2.17 (s, 6H), 1.25 (d, *J* = 6.3 Hz, 12H) ppm.

^{13}C NMR (101 MHz, $CDCl_3$): δ 167.8 (2C), 144.6 (2C), 99.8 (2C), 66.9(2C), 22.2 (4C), 19.4(2C) ppm. The CD_2 peak (around 25.0 ppm) was not found in the ^{13}C spectra.

HRMS (ESI$^+$): exact mass calculated for $[M+Na]^+$ ($C_{15}H_{21}D_2NONa^+$) requires *m/z* 306.1644, found *m/z* 306.1645.

IR (neat) ν_{max}: 3348, 2936, 1720, 1655, 1455, 1272, 1140, 1062, 742 cm $^{-1}$.

^{1}H NMR (400 MHz, $CDCl_3$)

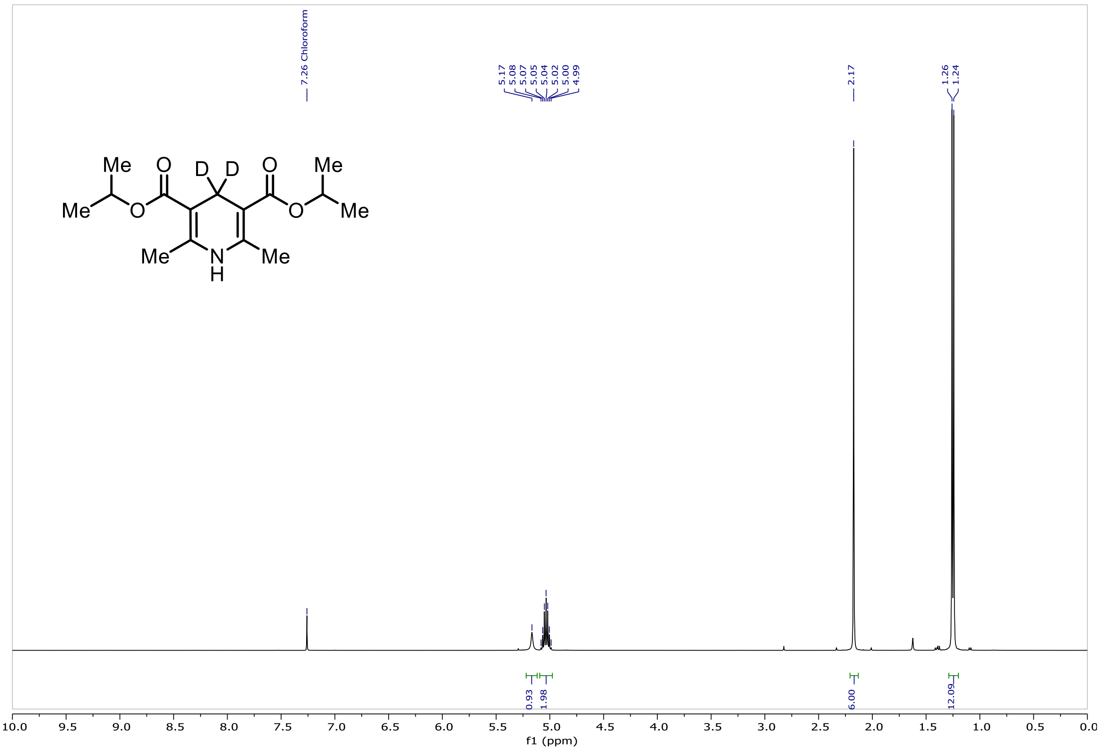

^{13}C NMR (101 MHz, $CDCl_3$)

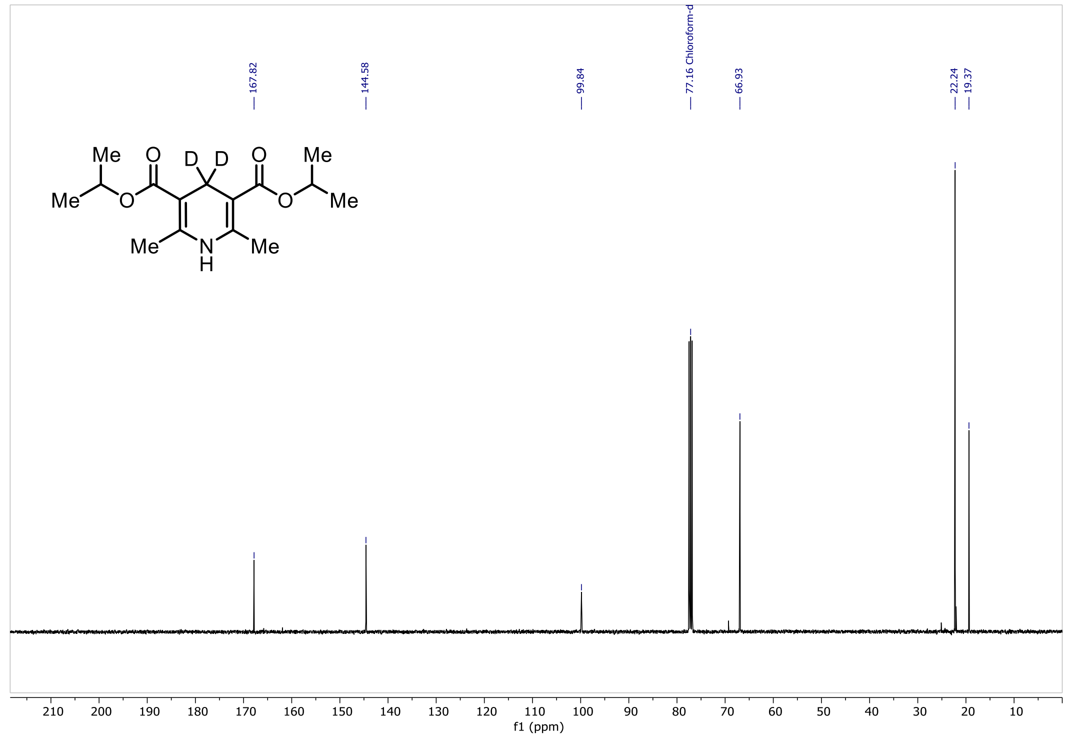

7.2: Deuterium labelling experiments

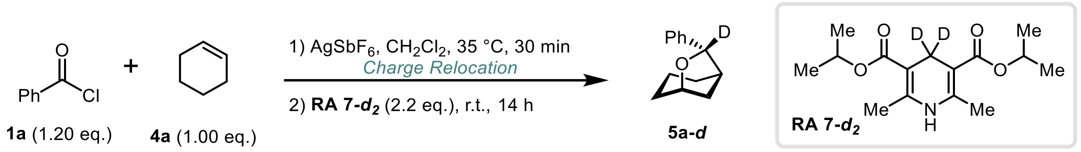

An oven-dried vial was flushed with argon and charged with a magnetic stir bar. After that, cyclohexene (0.20 mmol, 1.00 equiv.) and benzoyl chloride (0.24 mmol, 1.20 equiv.) were added, followed by dichloromethane (2 mL). The resulting solution was treated with silver hexafluoroantimonate (0.24 mmol, 1.20 equiv.) under argon atmosphere, and the vial was immediately heated in a sand bath at 35 °C. After 30 min, stirring was stopped and the supernatant was transferred by syringe to a separate vial. To this, reducing agent **RA 7**-*d*$_2$ (0.44 mmol, 2.20equiv.) was added. The vial was placed at ambient temperature (22–25 °C) and the solution was stirred at that temperature for 14 h. The reaction mixture was then treated with a saturated aqueous solution of $NaHCO_3$, extracted with DCM (3 × 5 mL), dried over anhydrous magnesium sulfate, filtered, and the filtrate was concentrated under reduced pressure. The resulting crude material was purified by flash column chromatography on silica gel (heptane/ethyl acetate) to give the title compounds.

5a-*d*: 7-Phenyl-6-oxabicyclo[3.2.1]octane-7-*d*

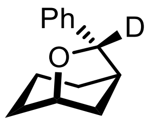

^{1}H NMR (400 MHz, $CDCl_3$): δ 7.44 – 7.34 (m, 2H), 7.31 (dd, *J* = 10.4, 4.8 Hz, 2H), 7.19 (dd, *J* = 10.4, 4.3 Hz, 1H), 4.54 – 4.46 (m, 1H), 2.57 (d, *J* = 3.0 Hz, 1H), 2.19 (dt, *J* = 10.8, 5.3 Hz, 1H), 1.85 – 1.76 (m, 1H), 1.74 (d, *J* = 11.0 Hz, 1H), 1.53 – 1.44 (m, 1H), 1.40 – 1.32 (m, 3H), 1.23 – 1.14 (m, 1H) ppm.

^{13}C NMR (101 MHz, $CDCl_3$): δ 140.8, 128.0 (2C), 126.2, 125.7 (2C), 82.9 (t, *J* = 22.2 Hz), 76.3, 39.4, 38.9, 32.20, 26.1, 18.1 ppm.

HRMS (EI$^+$): exact mass calculated for $[M]^+$ $(C_{13}H_{15}DO)^+$ requires *m/z* 189.1258, found *m/z* 189.1254.

IR (neat) ν_{max}: 3025, 2937, 1738, 1493, 1228, 1111, 1036, 981, 709, 698 cm^{-1}.

^{1}H NMR (400 MHz, $CDCl_3$)

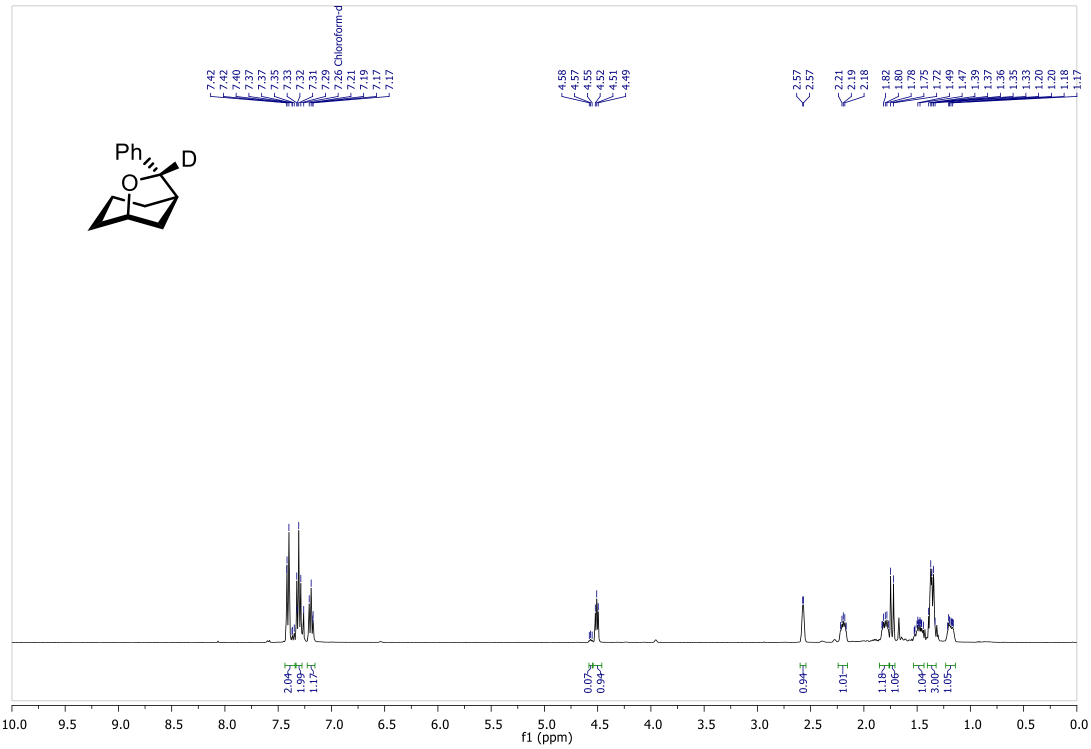

^{13}C NMR (101 MHz, $CDCl_3$)

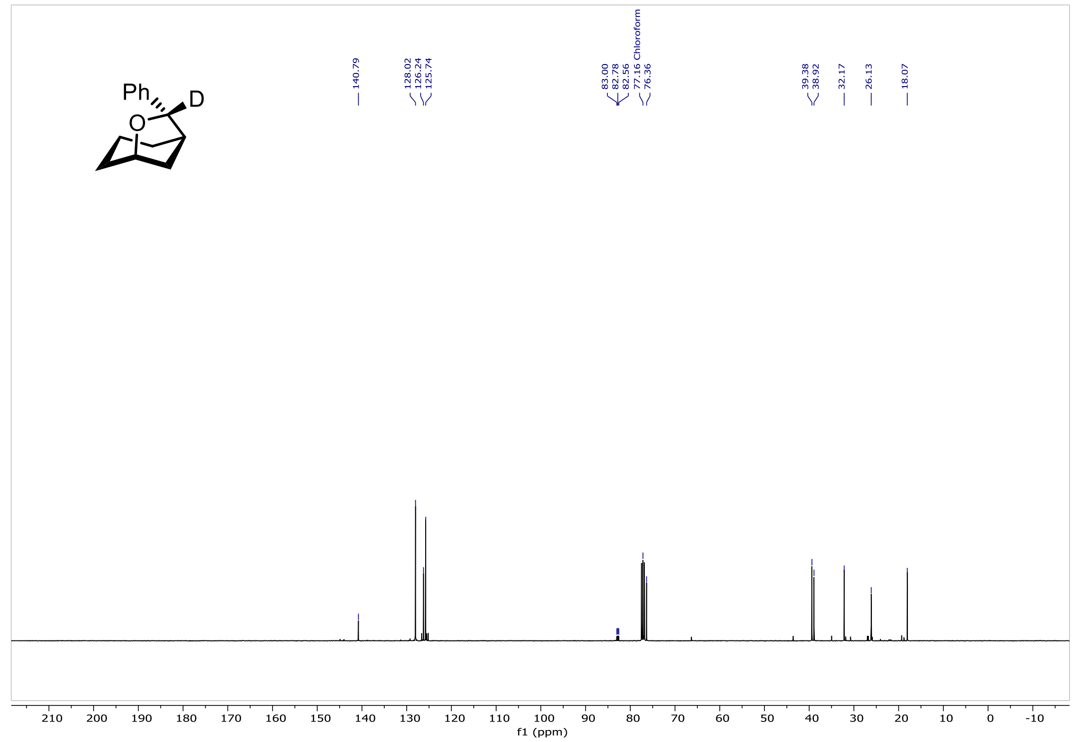

8. NMR Spectra

RA 4: Bis(2,6-di-*tert*-butylphenyl) 2,6-dimethyl-1,4-dihydropyridine-3,5-dicarboxylate

^{1}H NMR (400 MHz, $CDCl_3$)

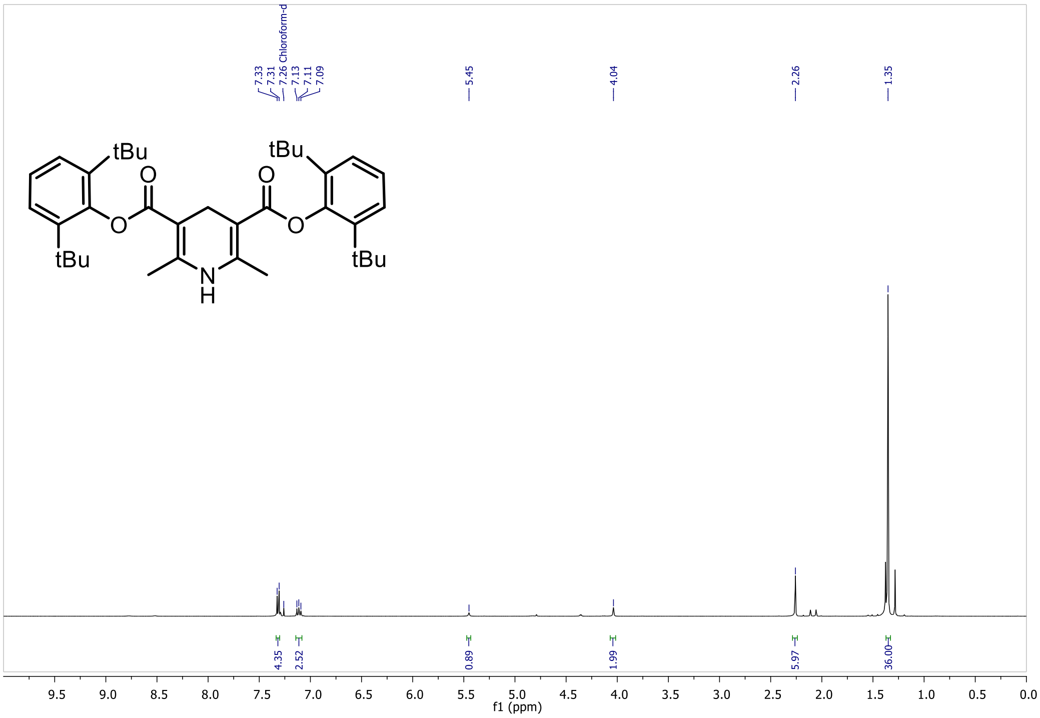

^{13}C NMR (101 MHz, $CDCl_3$)

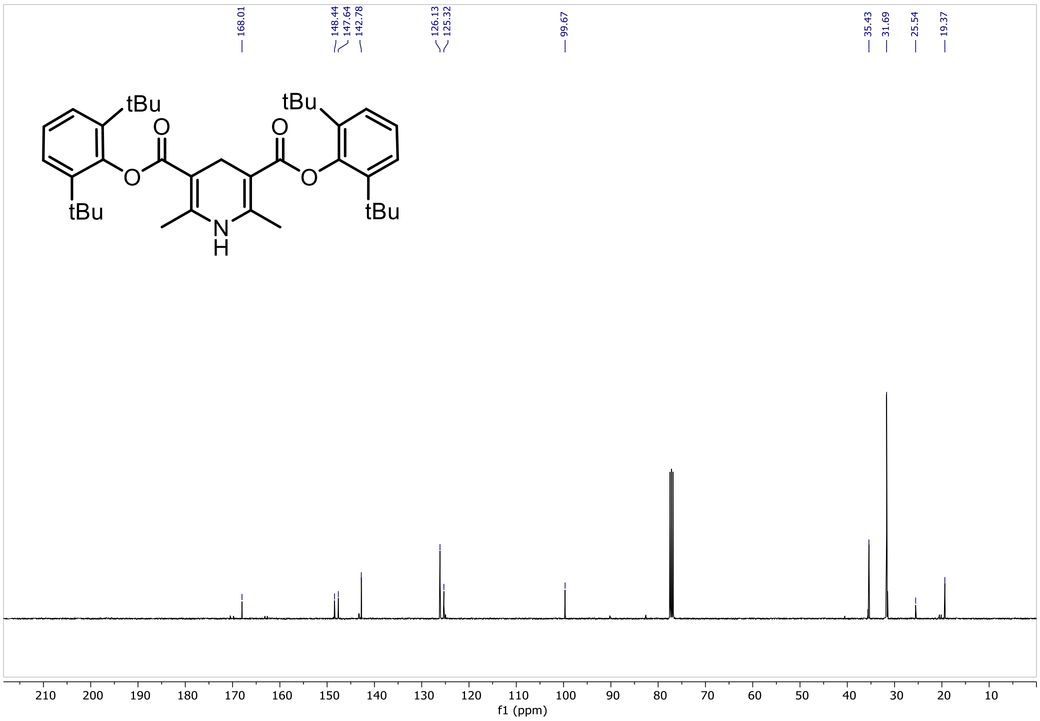

RA 6: Bis(2,4-dimethylpentan-3-yl) 2,6-dimethyl-1,4-dihydropyridine-3,5-dicarboxylate

^{1}H NMR (400 MHz, $CDCl_3$)

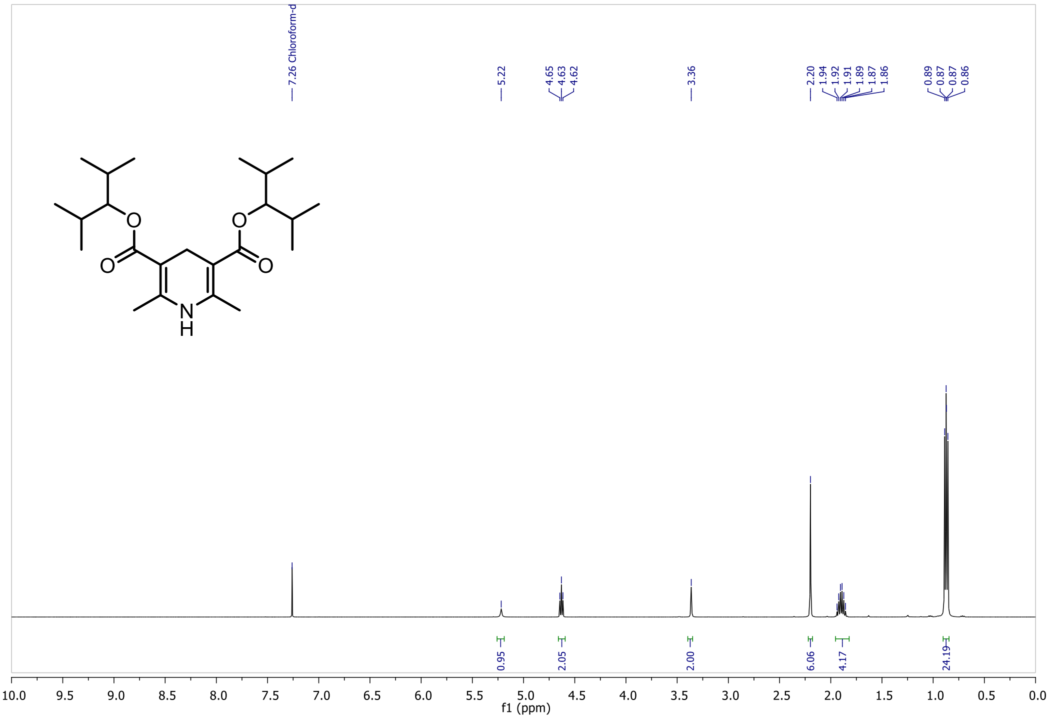

^{13}C NMR (101 MHz, $CDCl_3$)

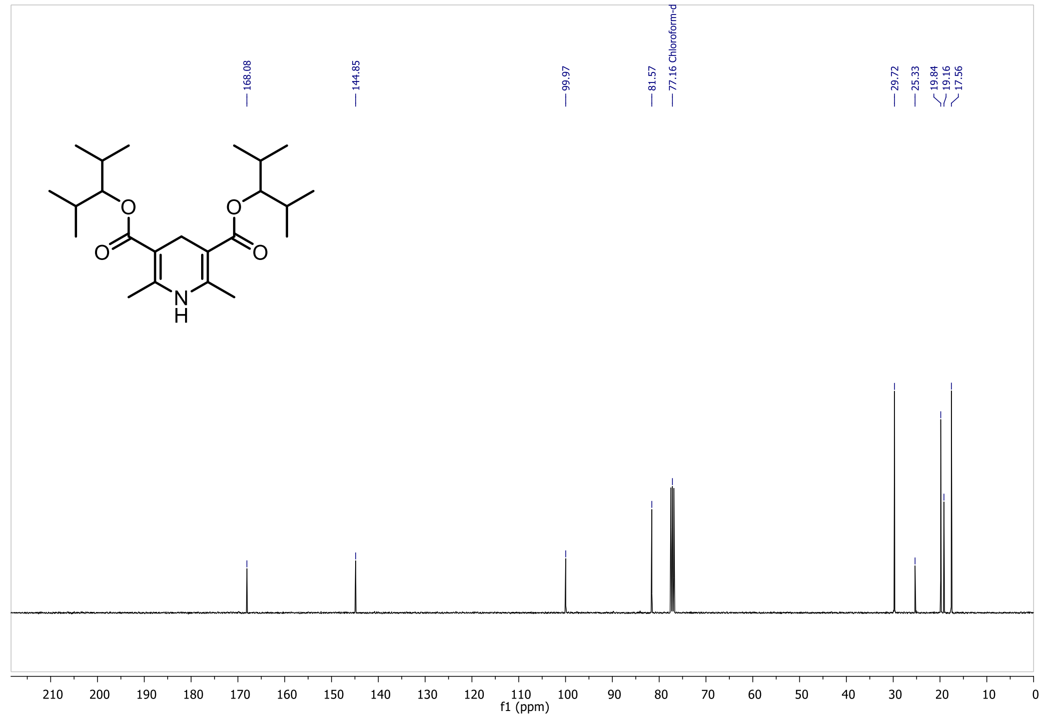

3a: *cis*-2-Benzyl-5-phenyltetrahydrofuran

^{1}H NMR (400 MHz, $CDCl_3$)

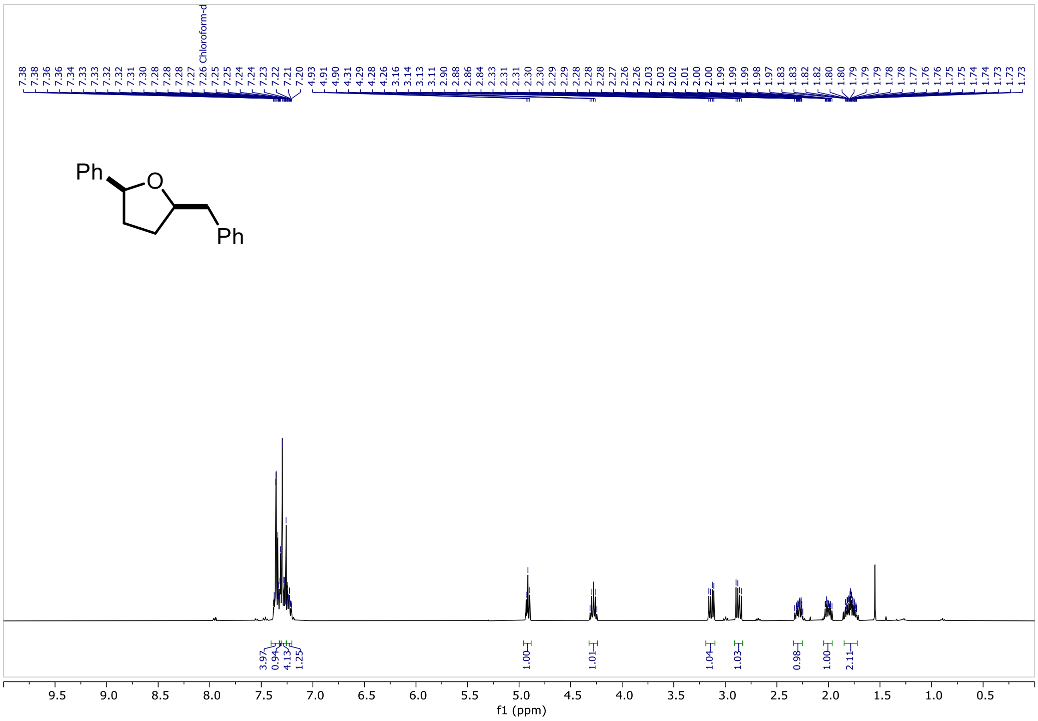

^{13}C NMR (101 MHz, $CDCl_3$)

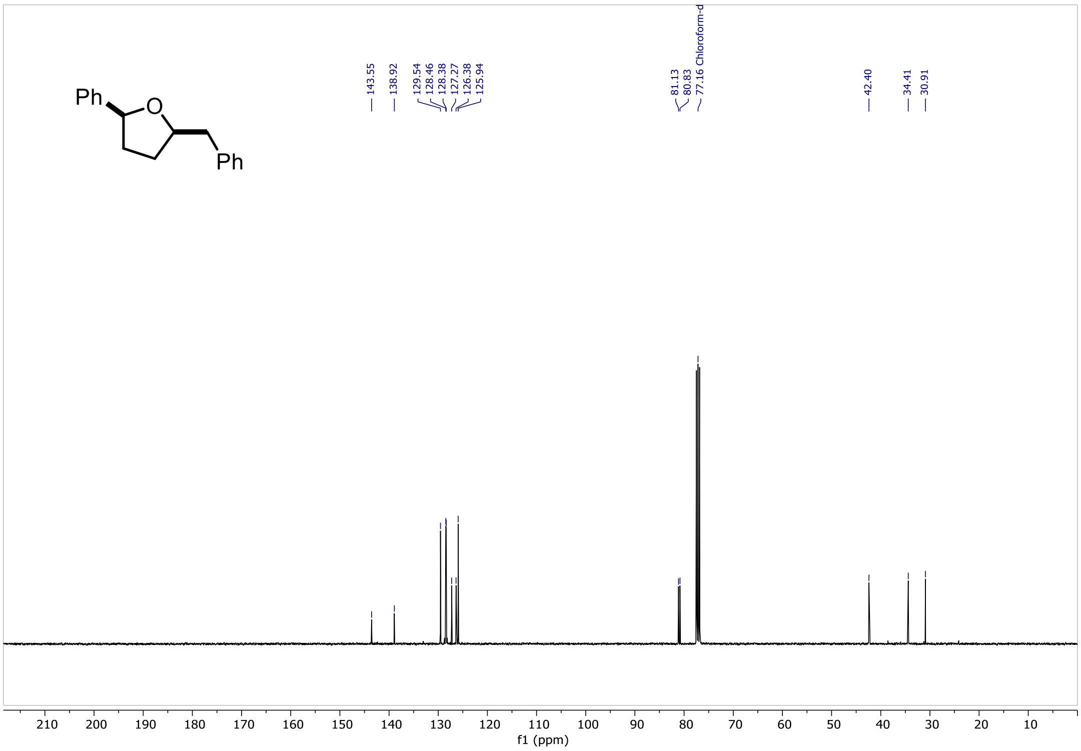

3b: *cis*-2-Benzyl-5-(2-iodophenyl)tetrahydrofuran

^{1}H NMR (400 MHz, $CDCl_3$)

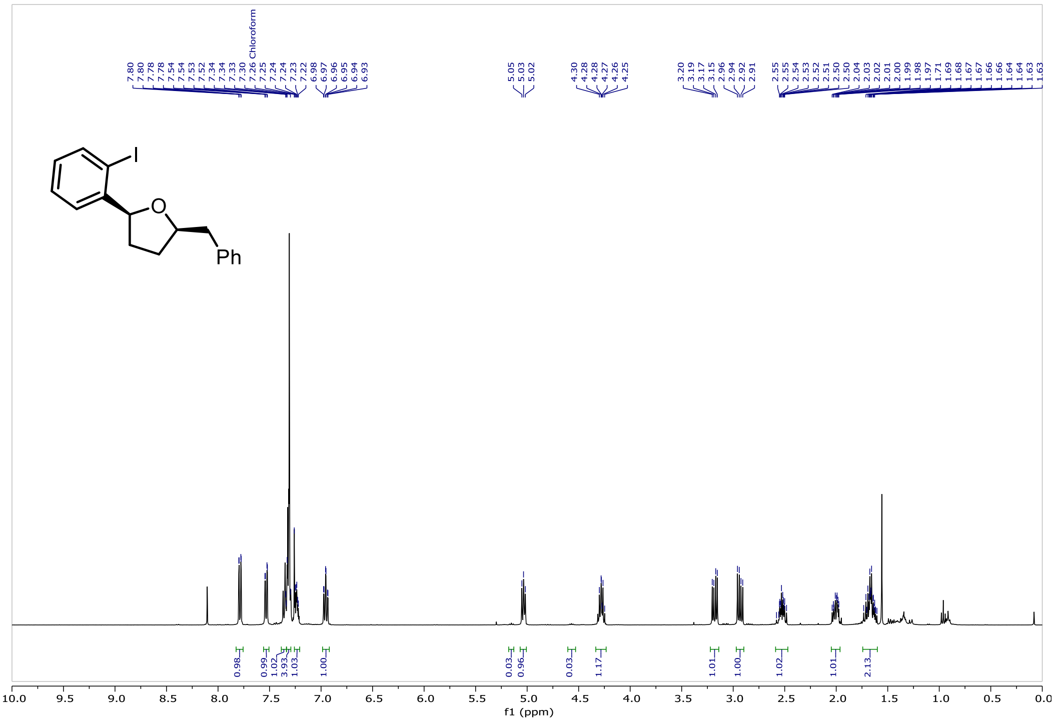

^{13}C NMR (101 MHz, $CDCl_3$)

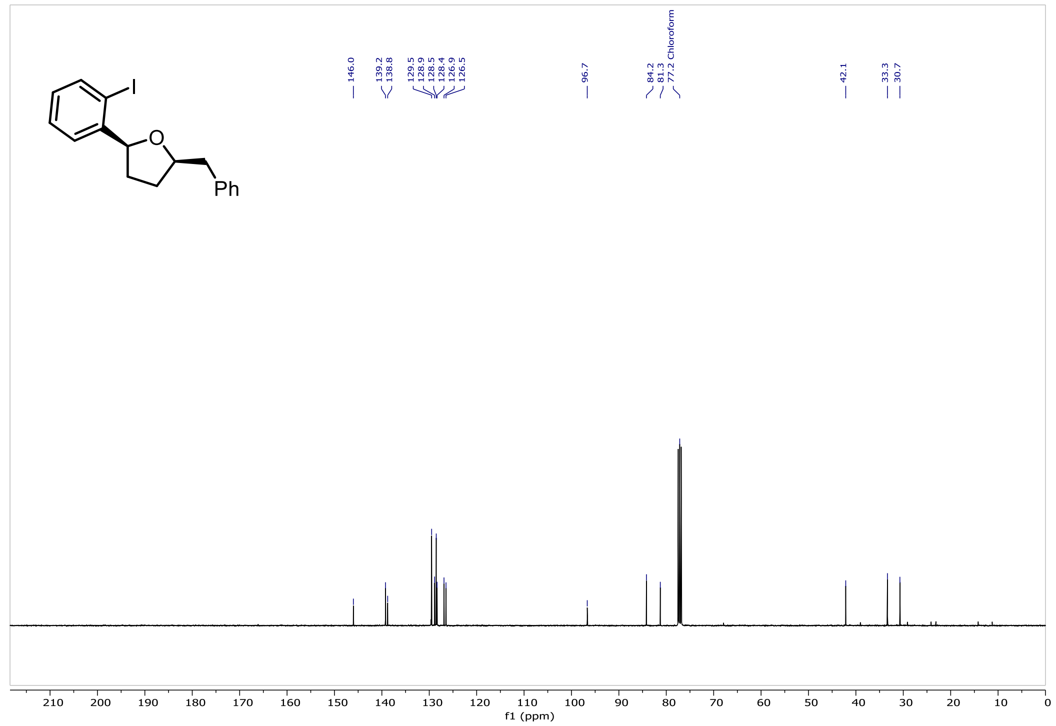

3c: *cis*-2-Benzyl-5-(4-fluorophenyl)tetrahydrofuran

^{1}H NMR (400 MHz, $CDCl_3$)

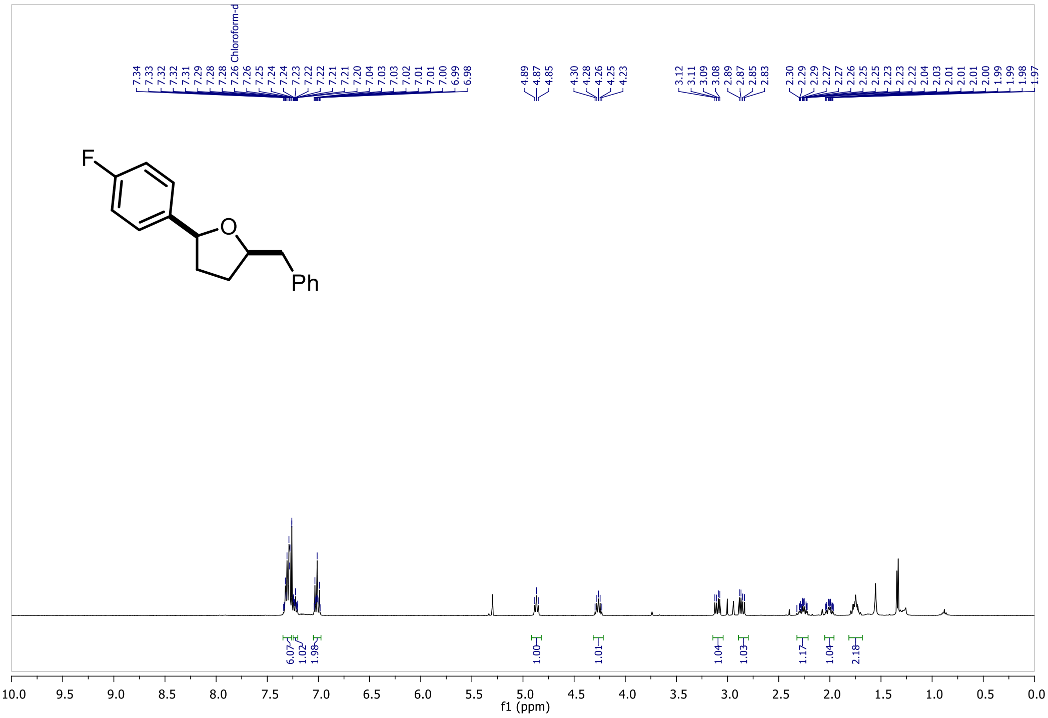

^{13}C NMR (101 MHz, $CDCl_3$)

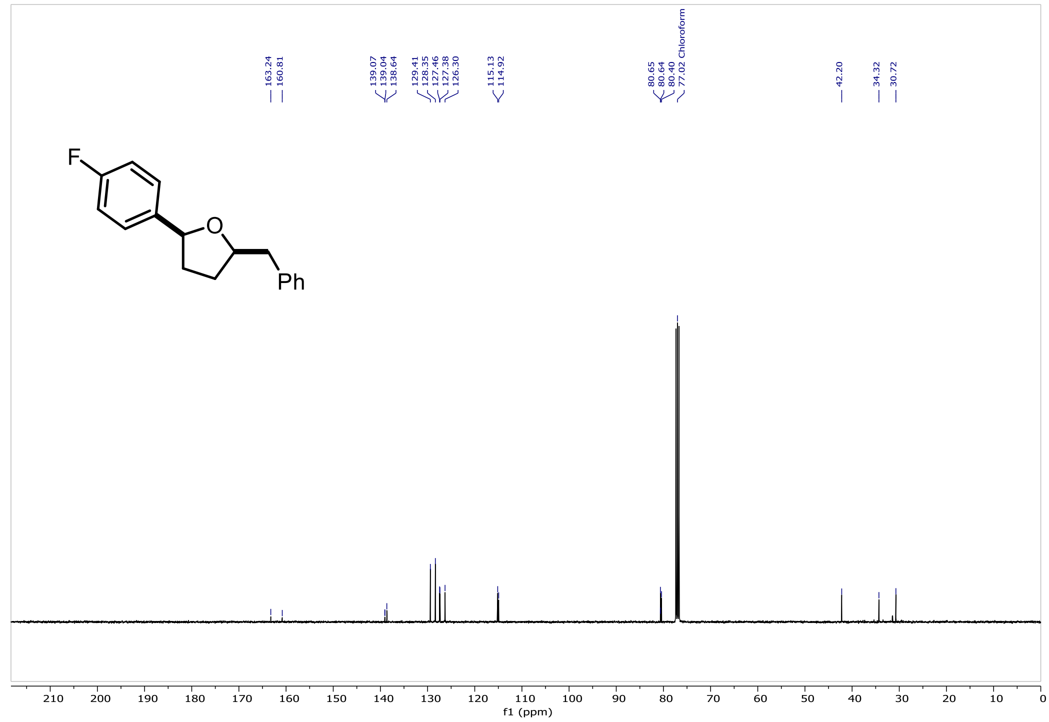

^{19}F NMR (377 MHz, $CDCl_3$)

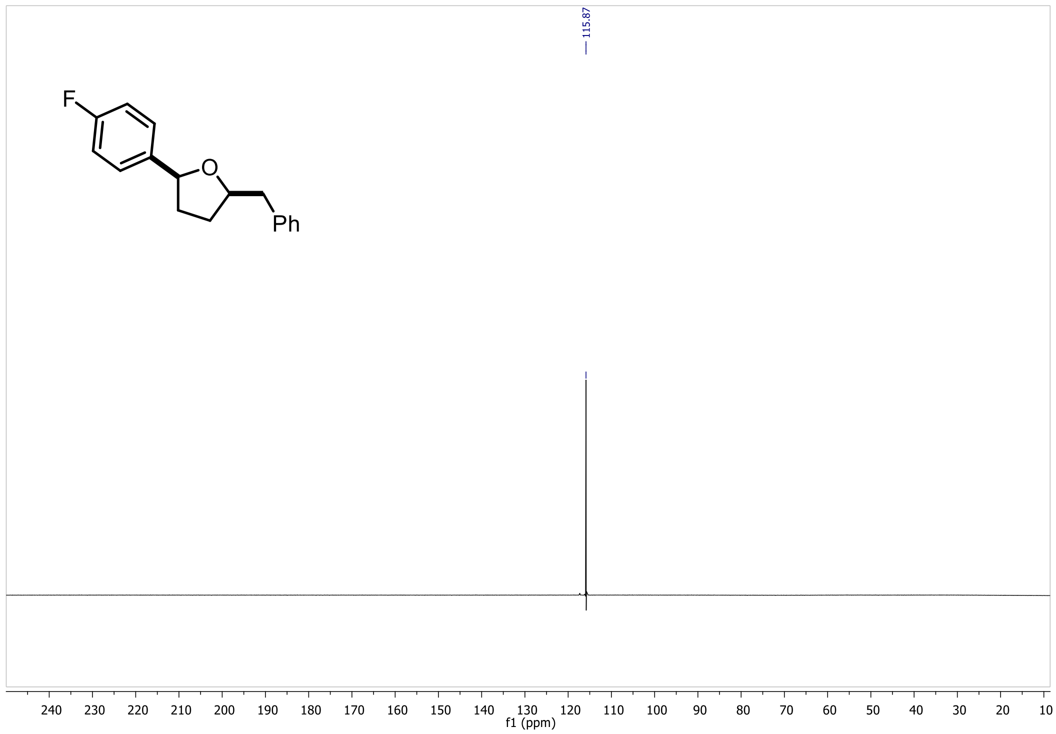

3d: *cis*-2-Benzyl-5-(2-bromophenyl)tetrahydrofuran

^{1}H NMR (400 MHz, $CDCl_3$)

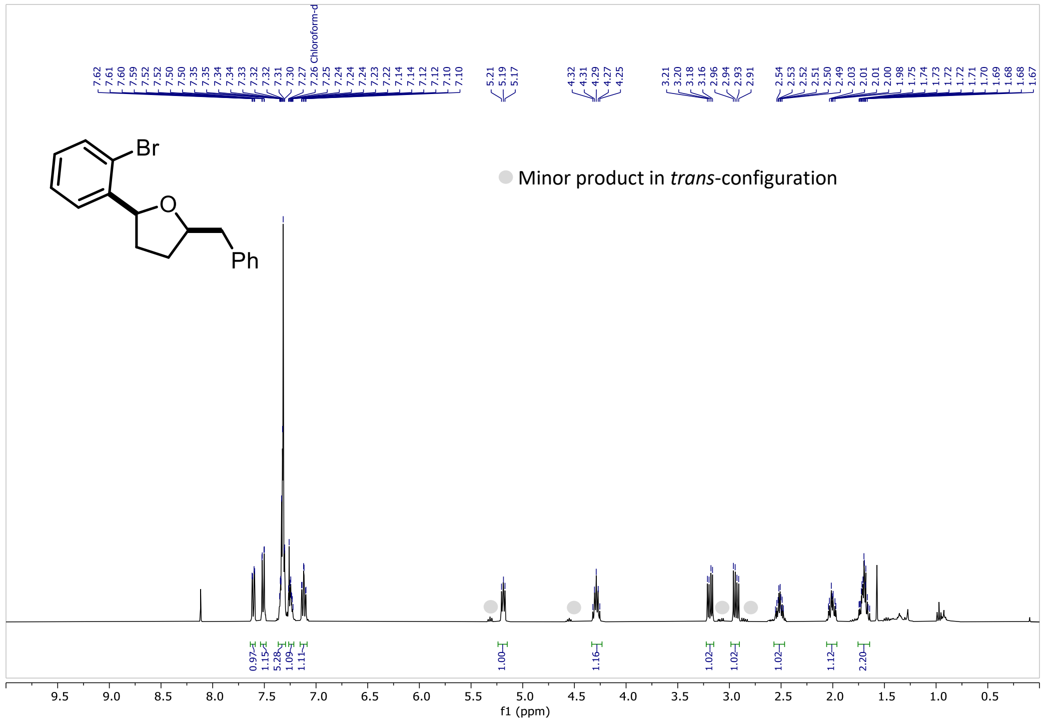

^{13}C NMR (101 MHz, $CDCl_3$)

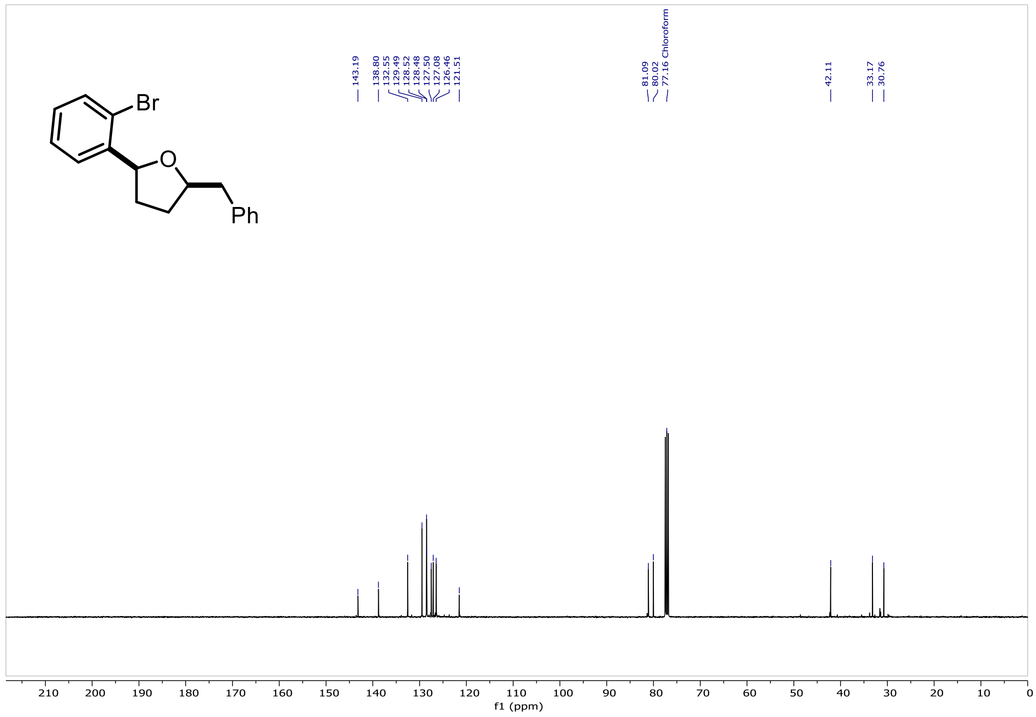

3e: *cis*-2-Benzyl-5-(4-chlorophenyl)tetrahydrofuran

^{1}H NMR (400 MHz, $CDCl_3$)

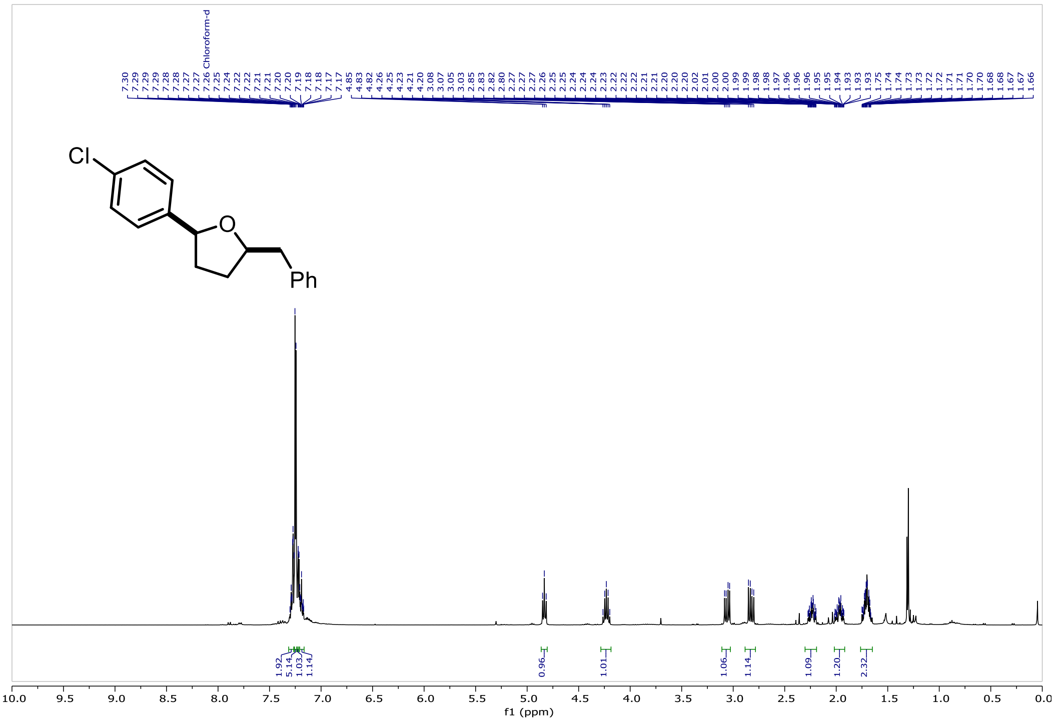

^{13}C NMR (101 MHz, $CDCl_3$)

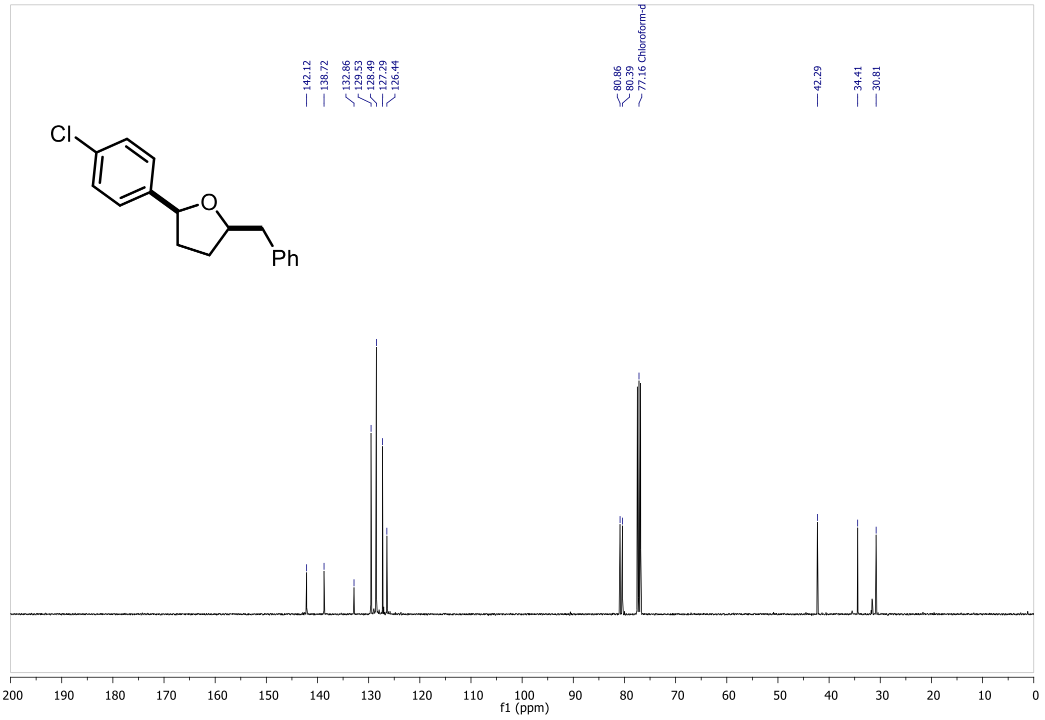

3f: *cis*-2-Benzyl-5-(4-(trifluoromethyl)phenyl)tetrahydrofuran

^{1}H NMR (400 MHz, $CDCl_3$)

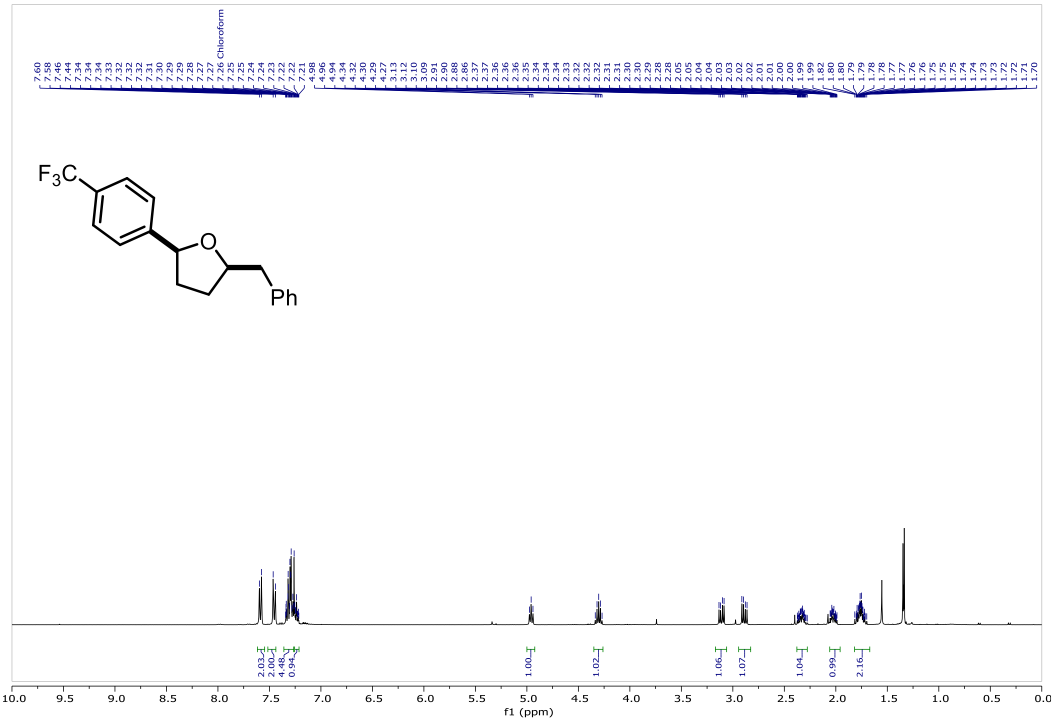

^{13}C NMR (101 MHz, $CDCl_3$)

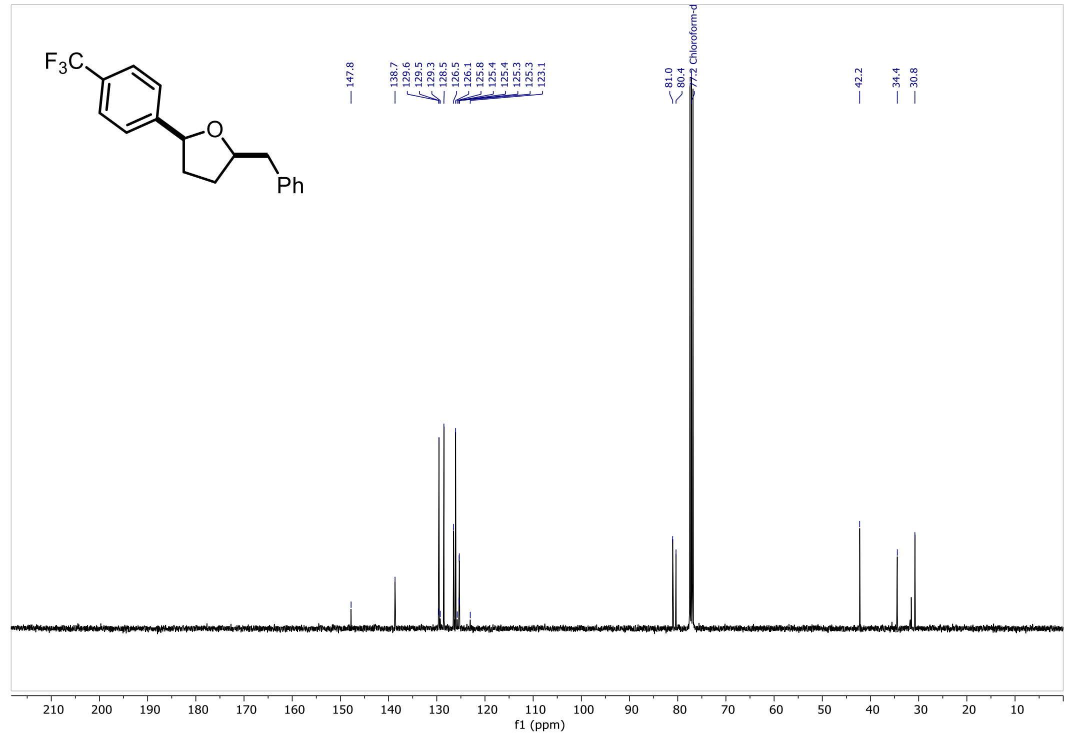

^{19}F NMR (377 MHz, $CDCl_3$)

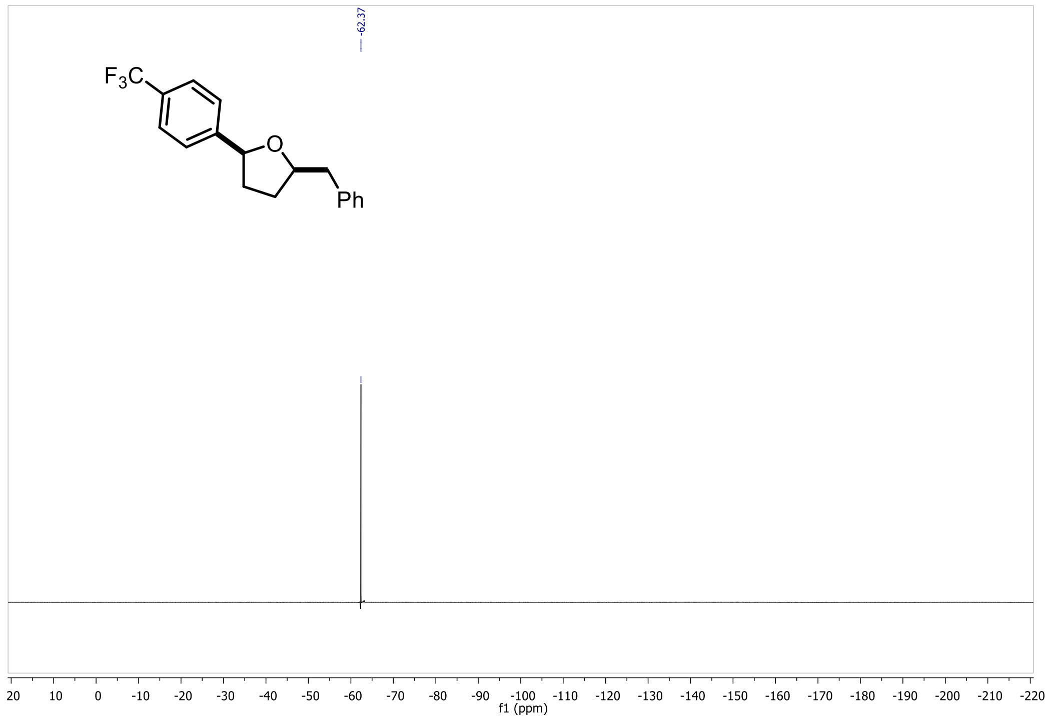

3g: *cis*-2-Benzyl-5-(*p*-tolyl)tetrahydrofuran

^{1}H NMR (400 MHz, $CDCl_3$)

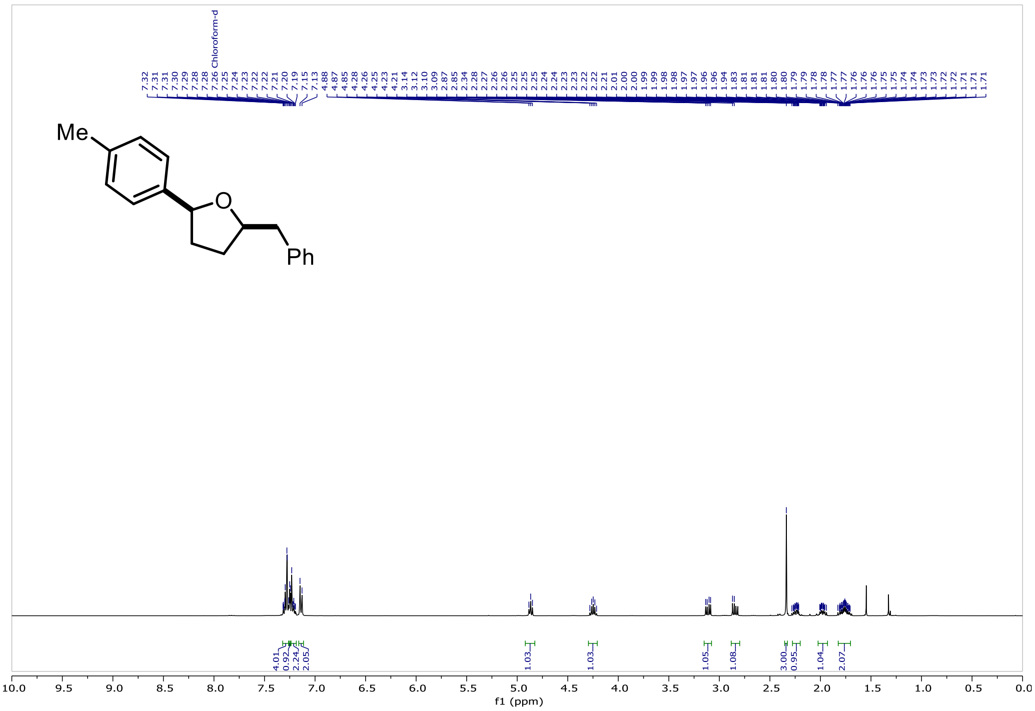

^{13}C NMR (101 MHz, $CDCl_3$)

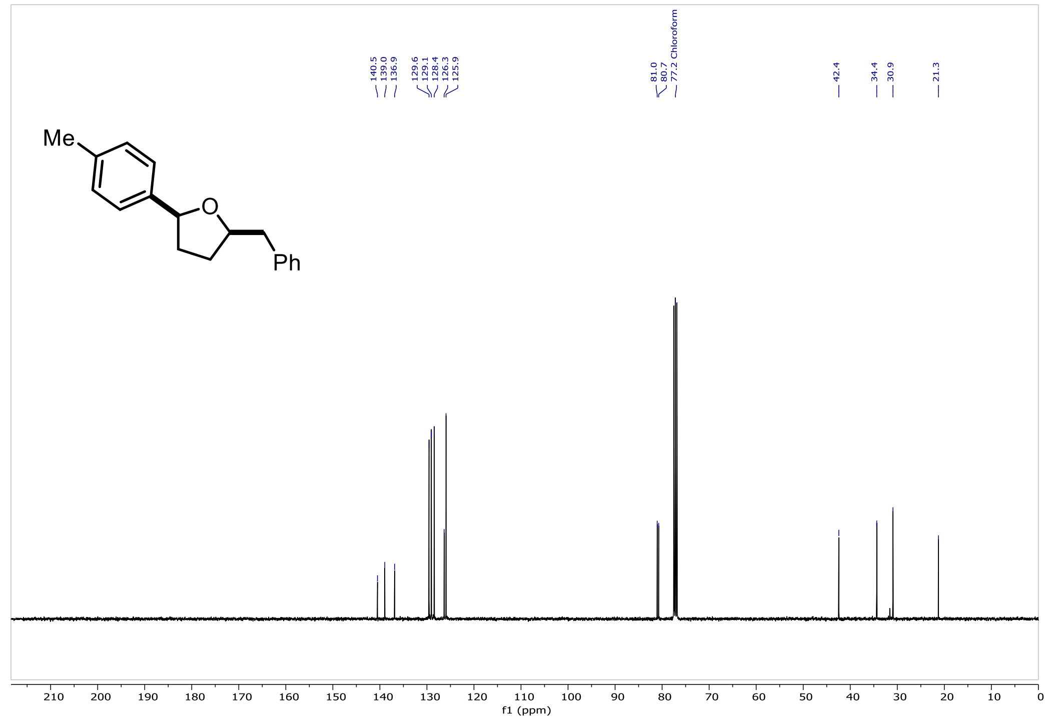

3h: *cis*-2-Benzyl-5-(3,5-dimethylphenyl)tetrahydrofuran

^{1}H NMR (400 MHz, $CDCl_3$)

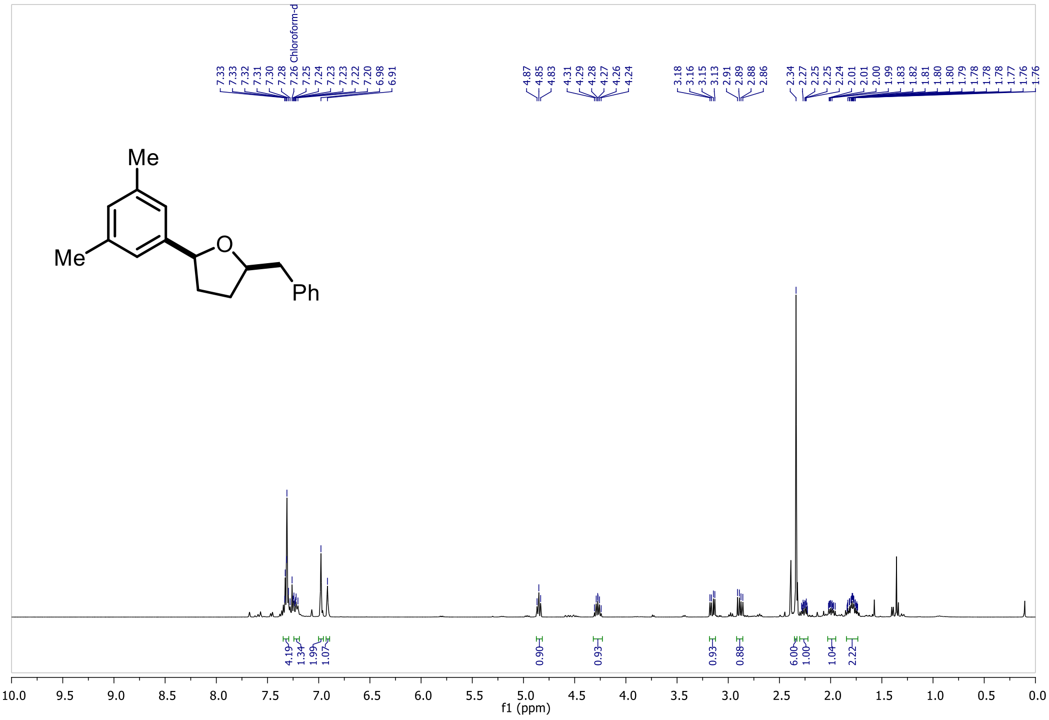

^{13}C NMR (101 MHz, $CDCl_3$)

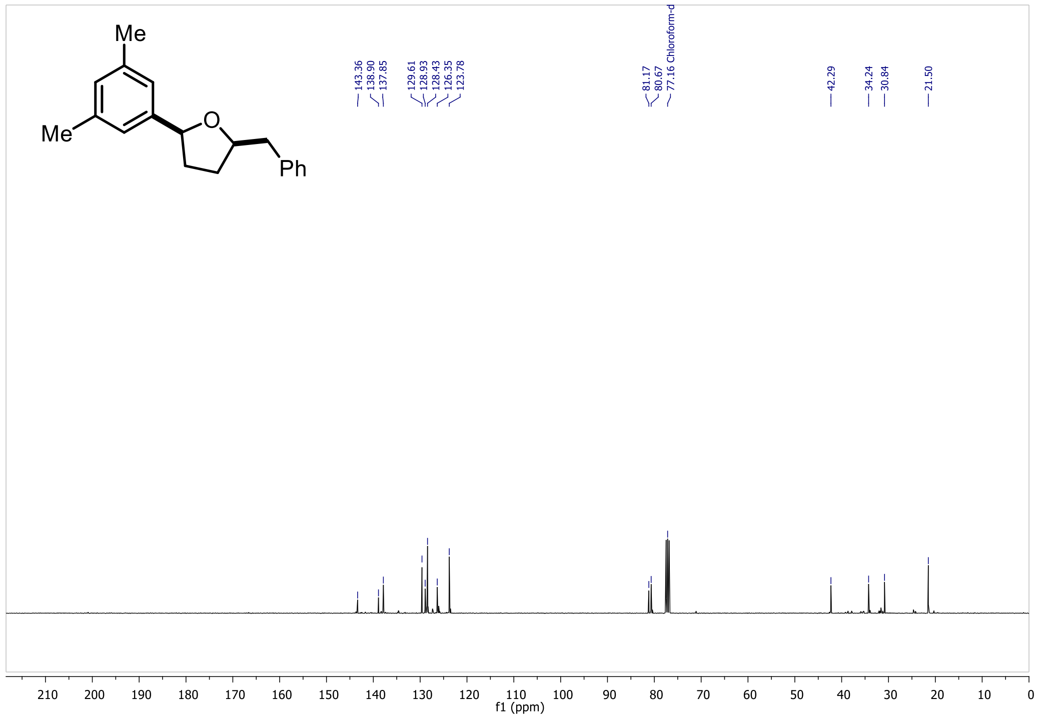

3i: *cis*-2-Benzyl-5-(naphthalen-2-yl)tetrahydrofuran

^{1}H NMR (400 MHz, $CDCl_3$)

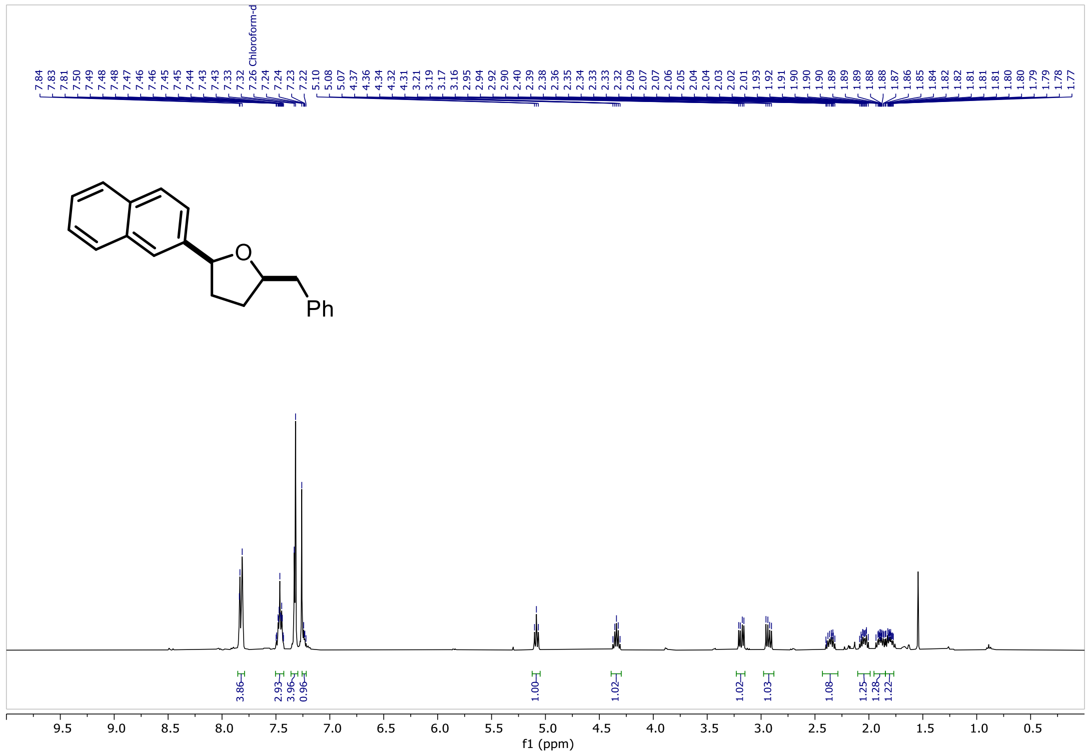

^{13}C NMR (101 MHz, $CDCl_3$)

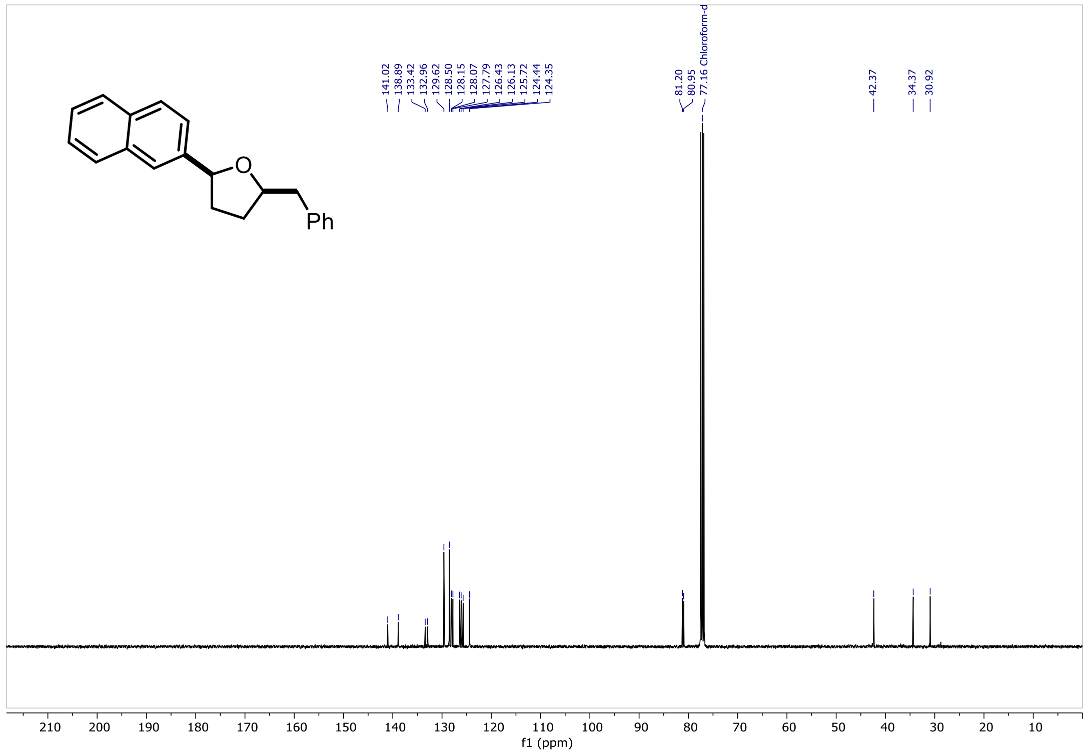

3j: 2-Benzyl-5-cyclohexyltetrahydrofuran

^{1}H NMR (400 MHz, $CDCl_3$)

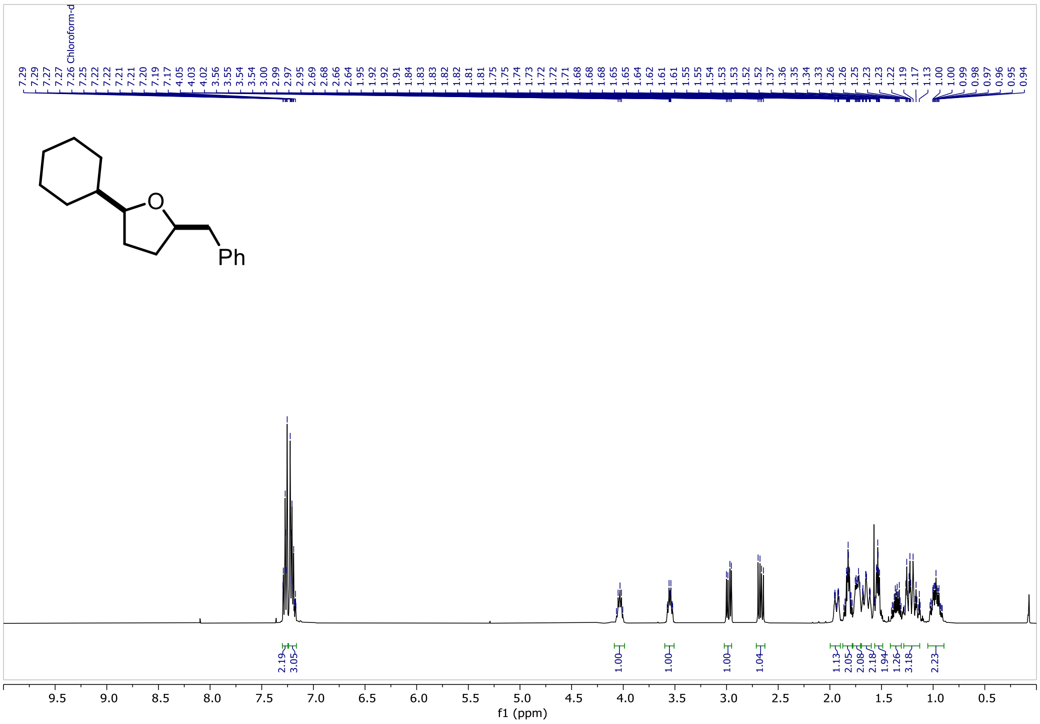

^{13}C NMR (101 MHz, $CDCl_3$)

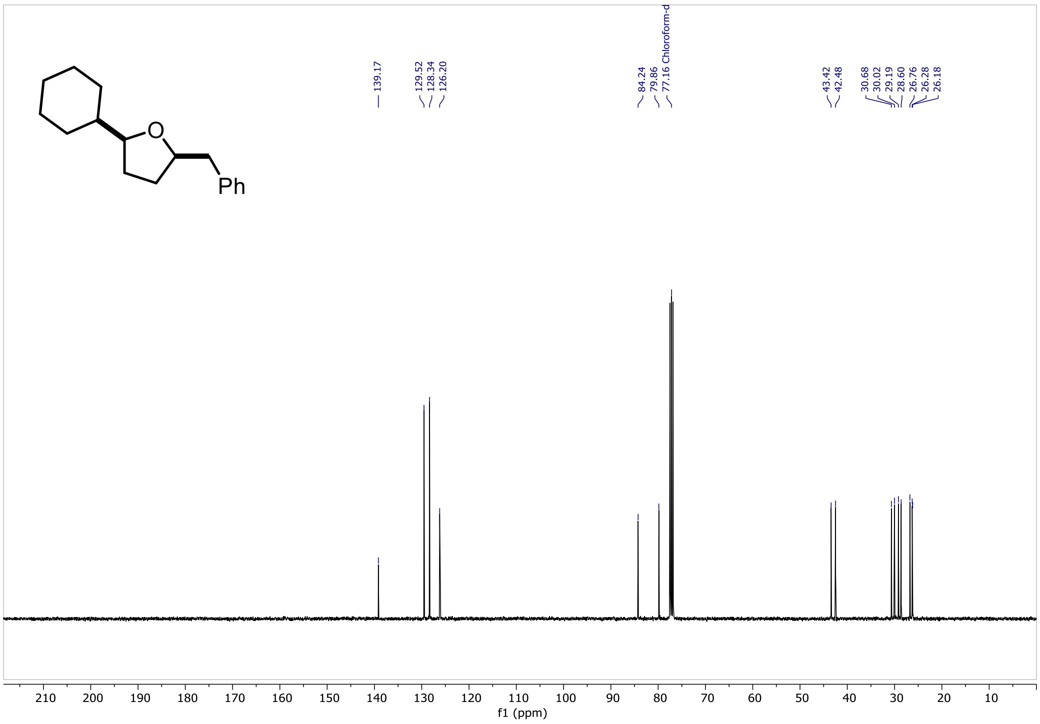

3k: *cis*-2-Benzyl-5-isopropyltetrahydrofuran

^{1}H NMR (400 MHz, $CDCl_3$)

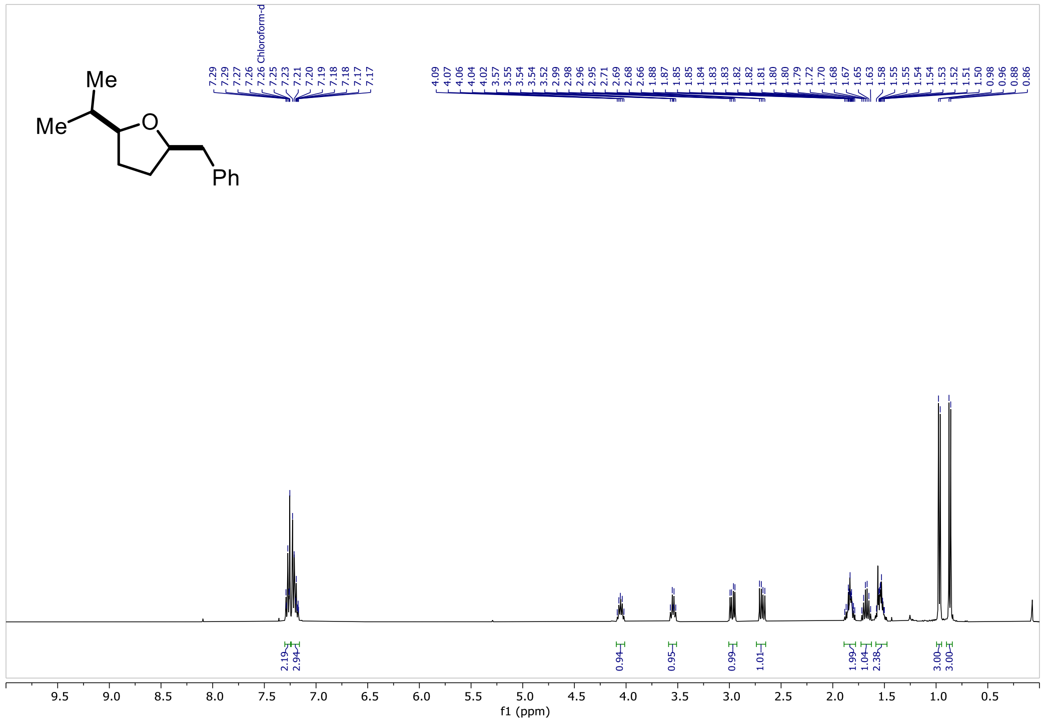

^{13}C NMR (101 MHz, $CDCl_3$)

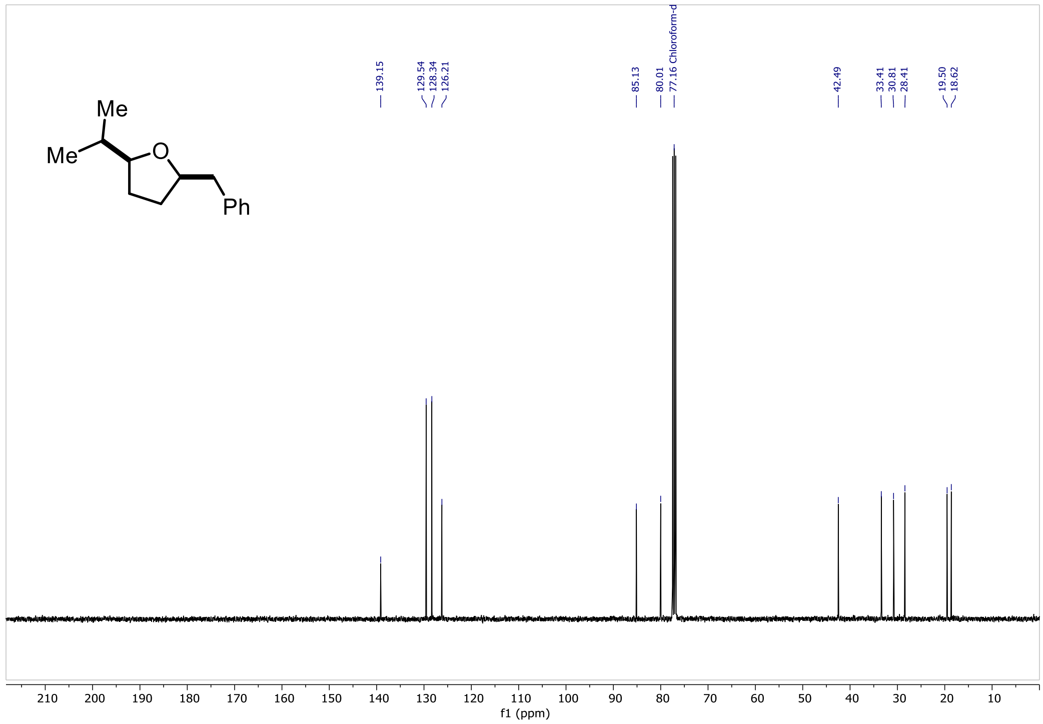

3l: *cis*-2-Benzyl-5-isobutyltetrahydrofuran

^{1}H NMR (400 MHz, $CDCl_3$)

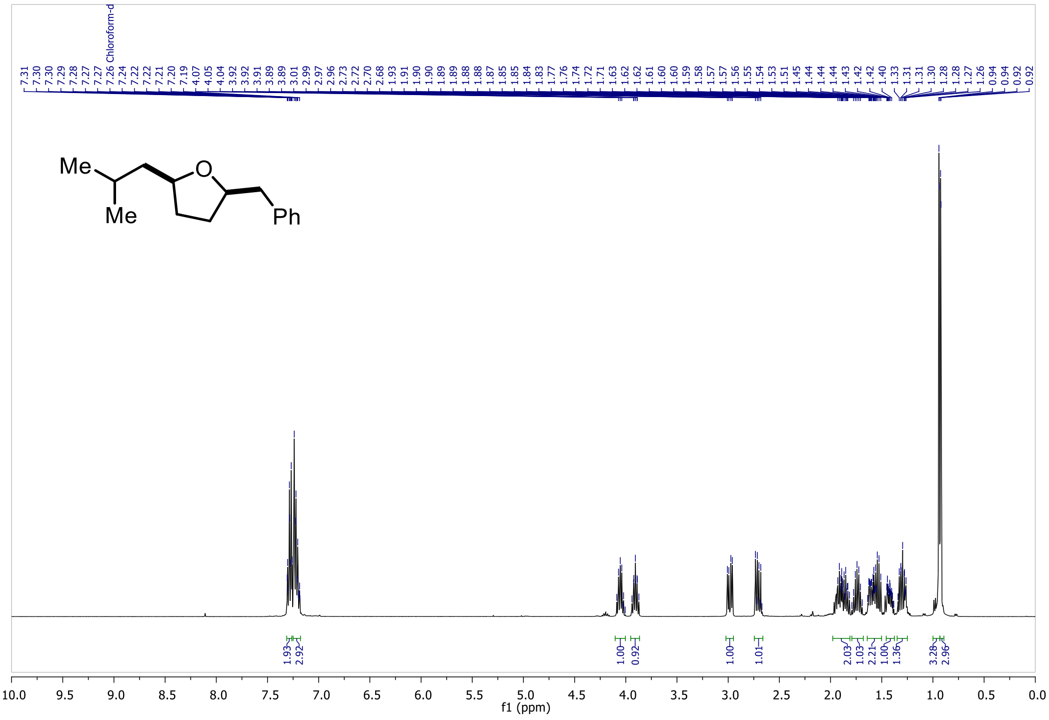

^{13}C NMR (101 MHz, $CDCl_3$)

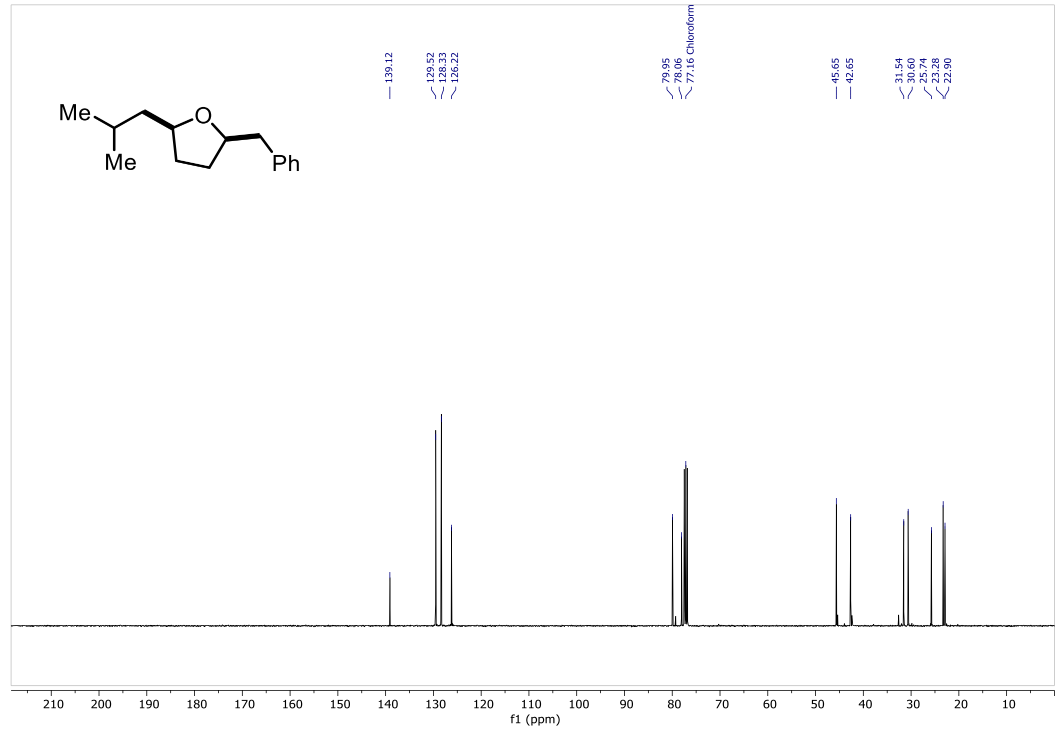

3m: *cis*-2-Benzyl-5-(*tert*-butyl)tetrahydrofuran

^{1}H NMR (400 MHz, $CDCl_3$)

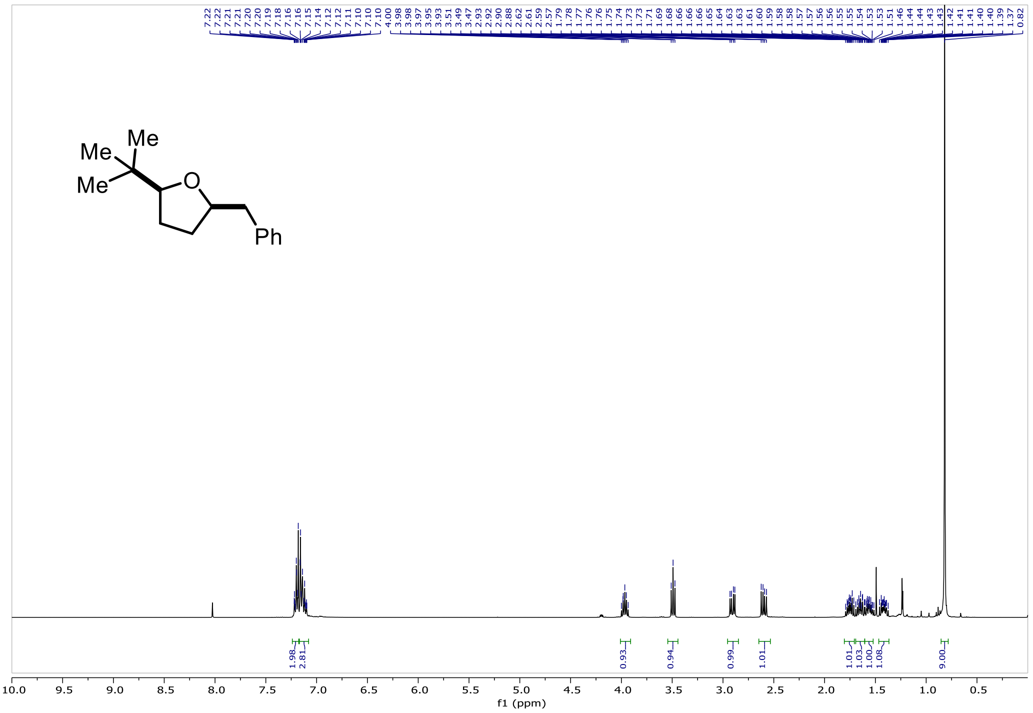

^{13}C NMR (101 MHz, $CDCl_3$)

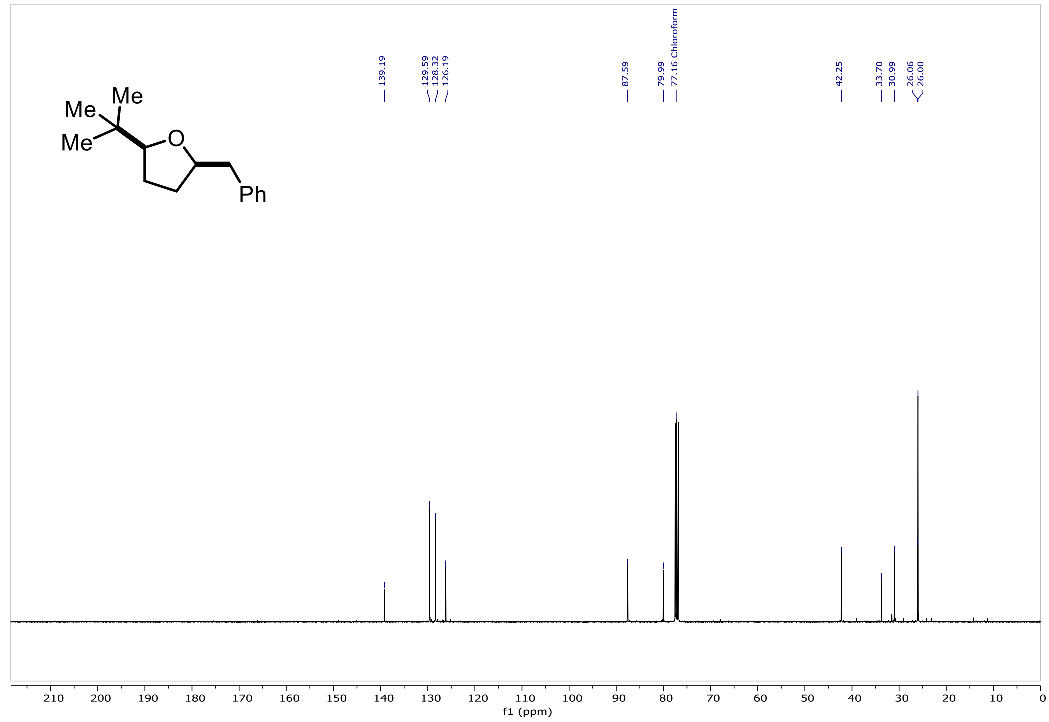

3n: *cis*-2-(Adamantan-1-yl)-5-benzyltetrahydrofuran

^{1}H NMR (400 MHz, $CDCl_3$)

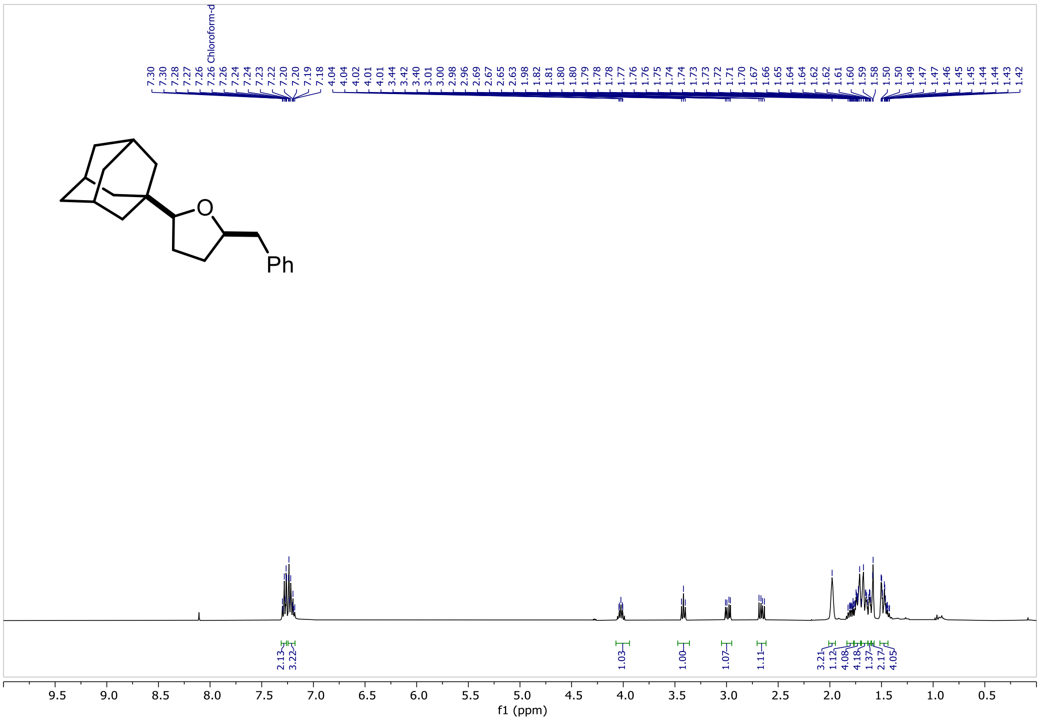

^{13}C NMR (101 MHz, $CDCl_3$)

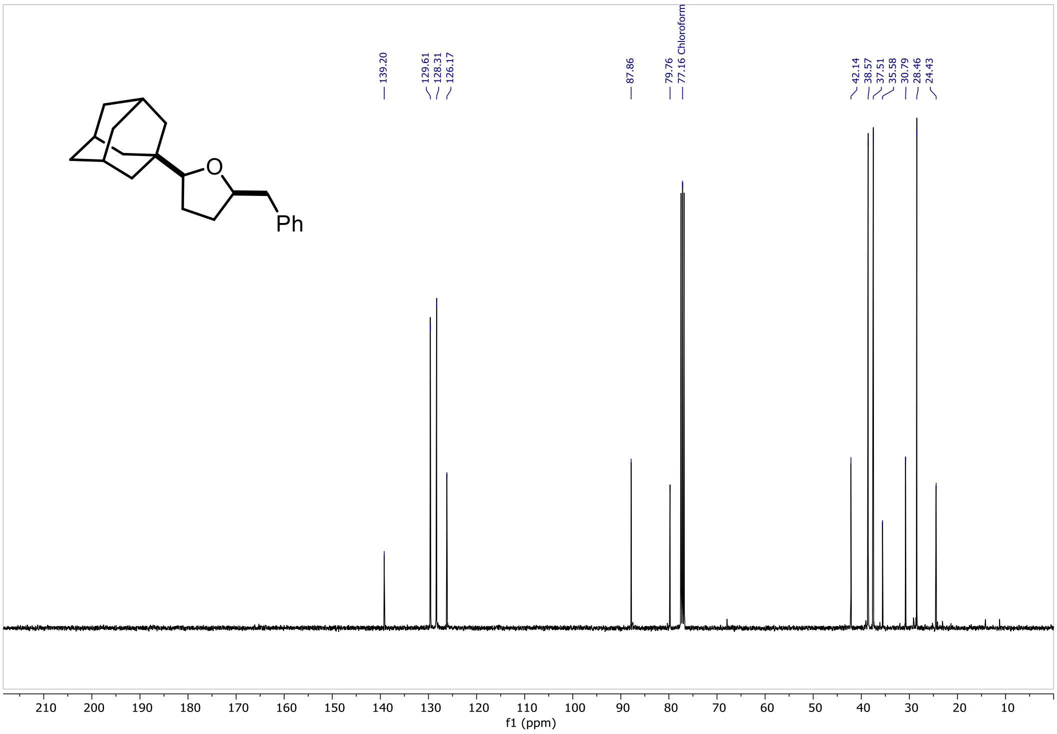

3o: *cis*-2-Cyclohexyl-5-phenyltetrahydrofuran

^{1}H NMR (600 MHz, $CDCl_3$)

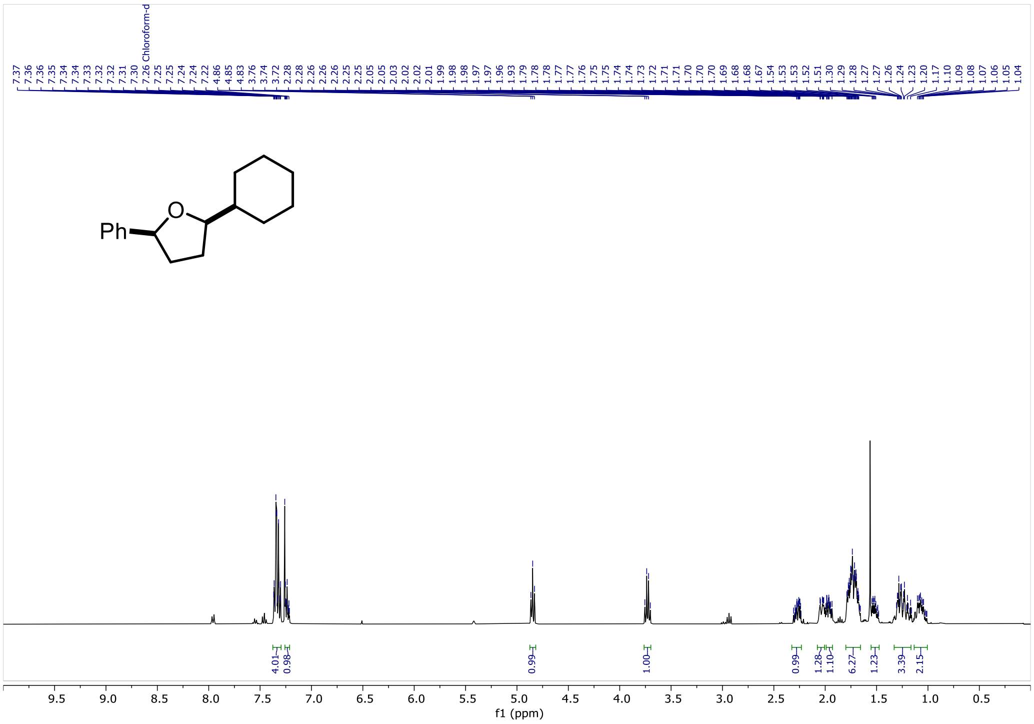

^{13}C NMR (101 MHz, $CDCl_3$)

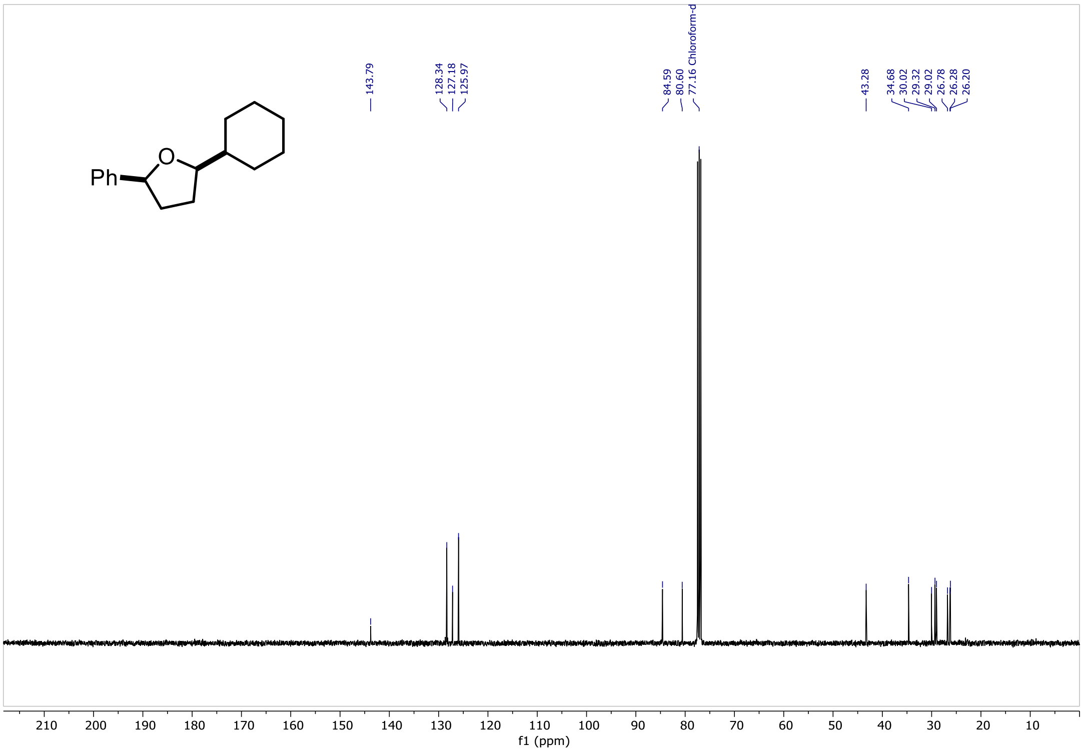

3p: *cis*-2-Cyclopentyl-5-phenyltetrahydrofuran

^{1}H NMR (600 MHz, $CDCl_3$)

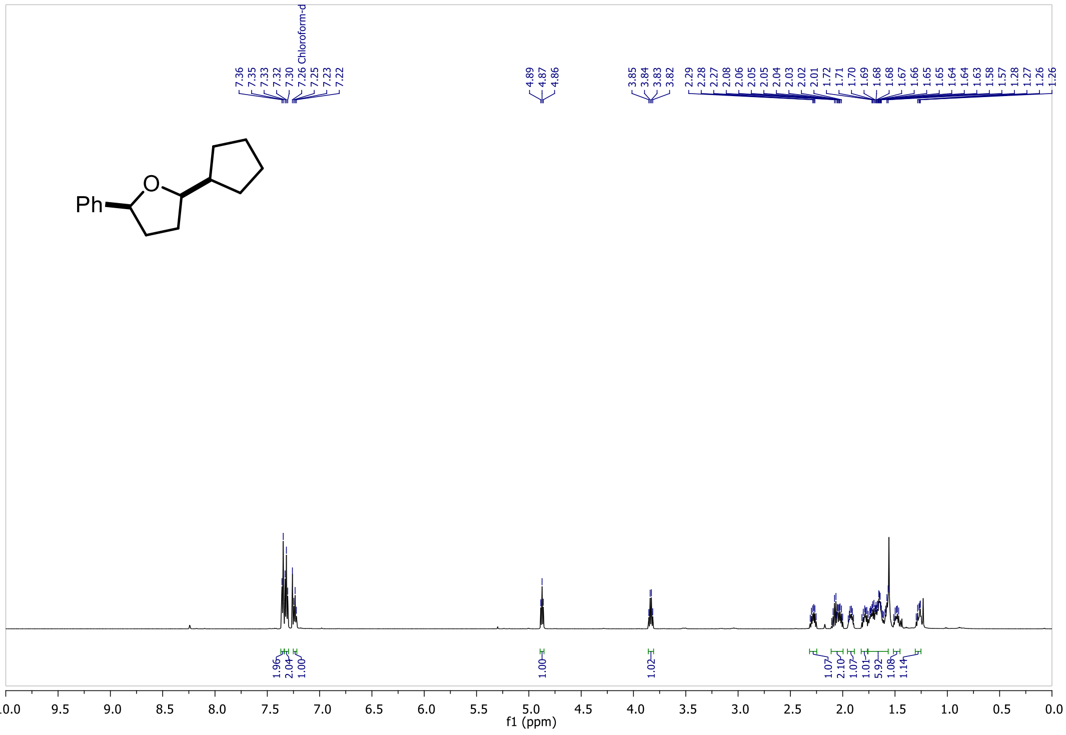

^{13}C NMR (151 MHz, $CDCl_3$)

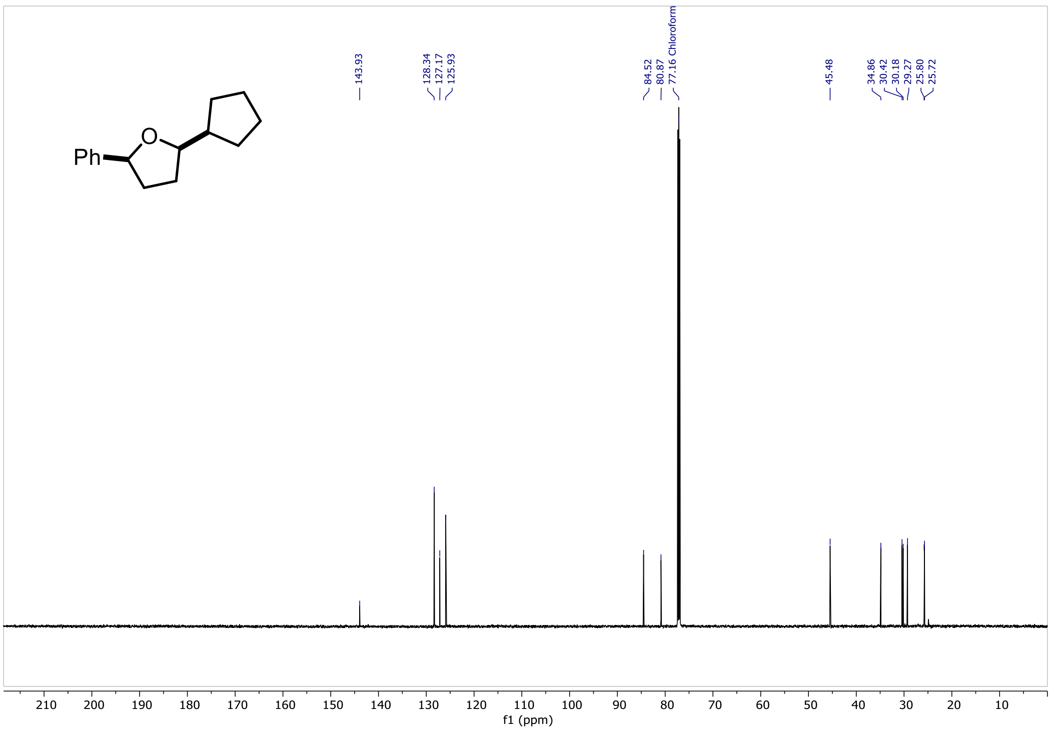

3q: *cis*-2-Octyl-5-phenyltetrahydrofuran

^{1}H NMR (400 MHz, $CDCl_3$)

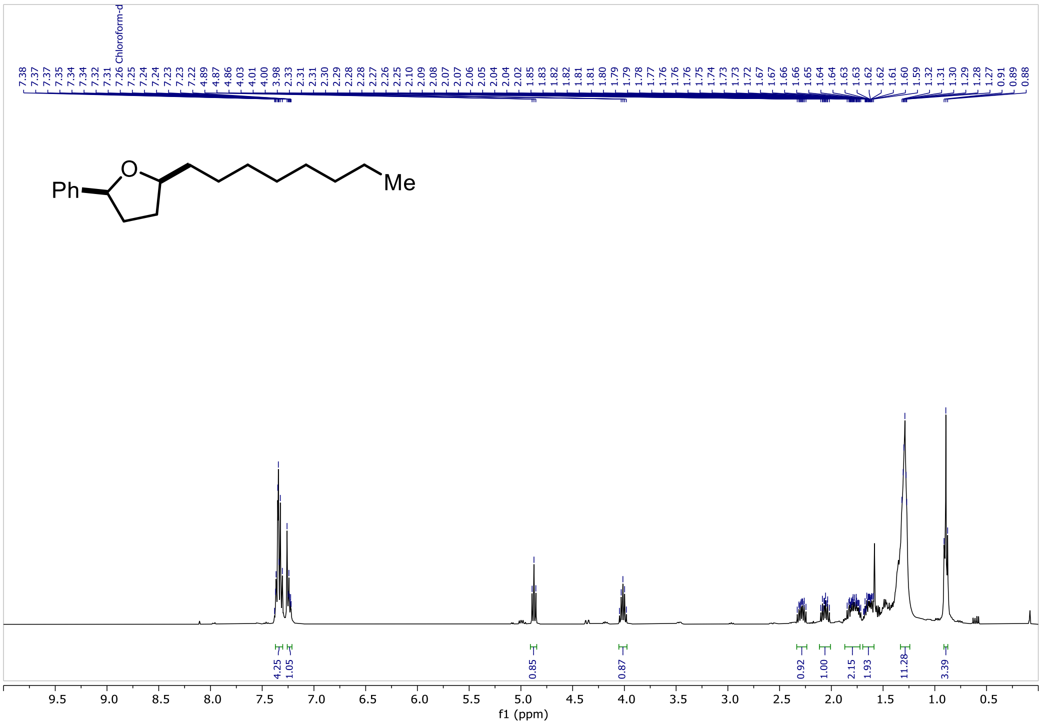

^{13}C NMR (101 MHz, $CDCl_3$)

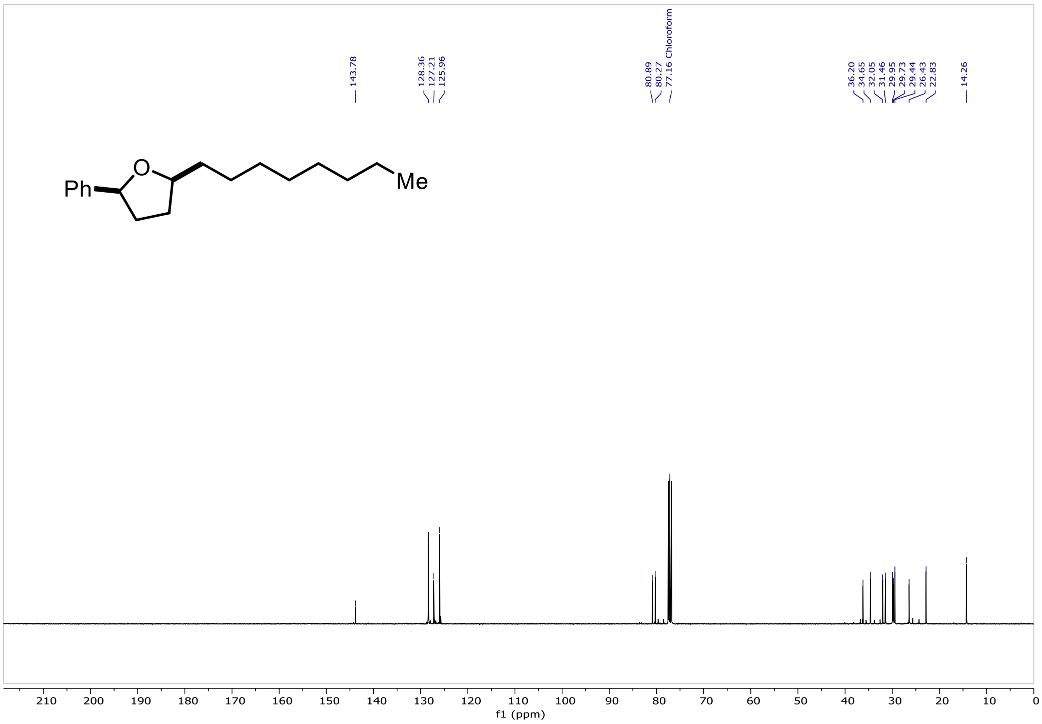

3r: *cis*-7-(5-Phenyltetrahydrofuran-2-yl)-1-(pyrrolidin-1-yl)heptan-1-one

^{1}H NMR (400 MHz, $CDCl_3$)

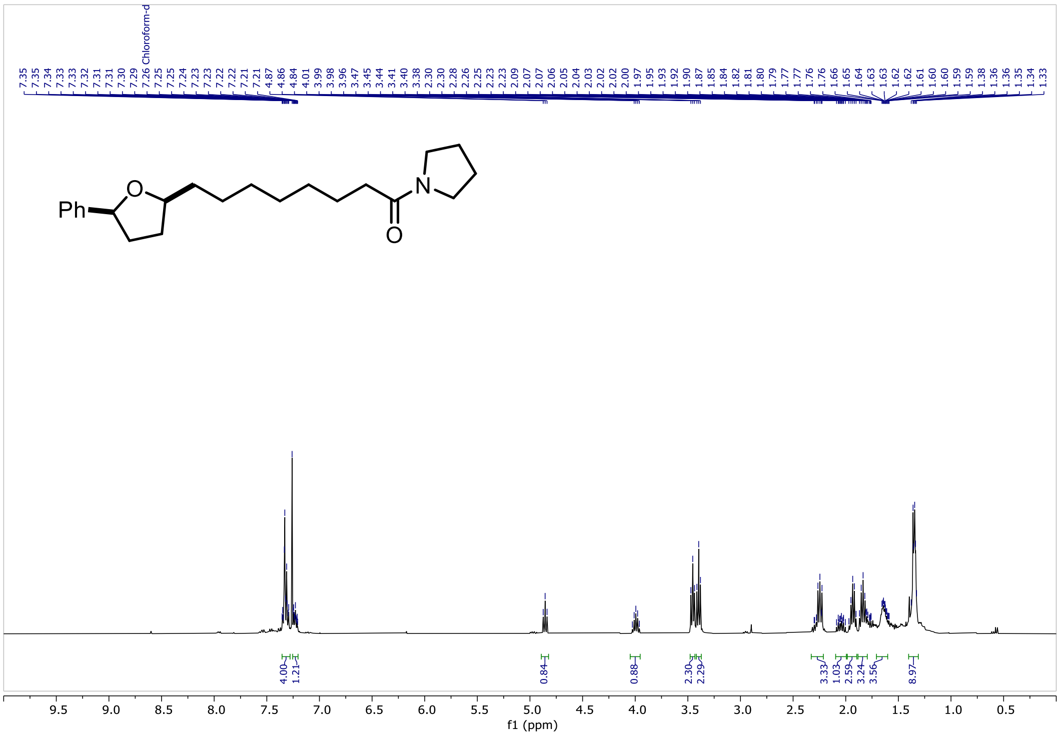

^{13}C NMR (176 MHz, $CDCl_3$)

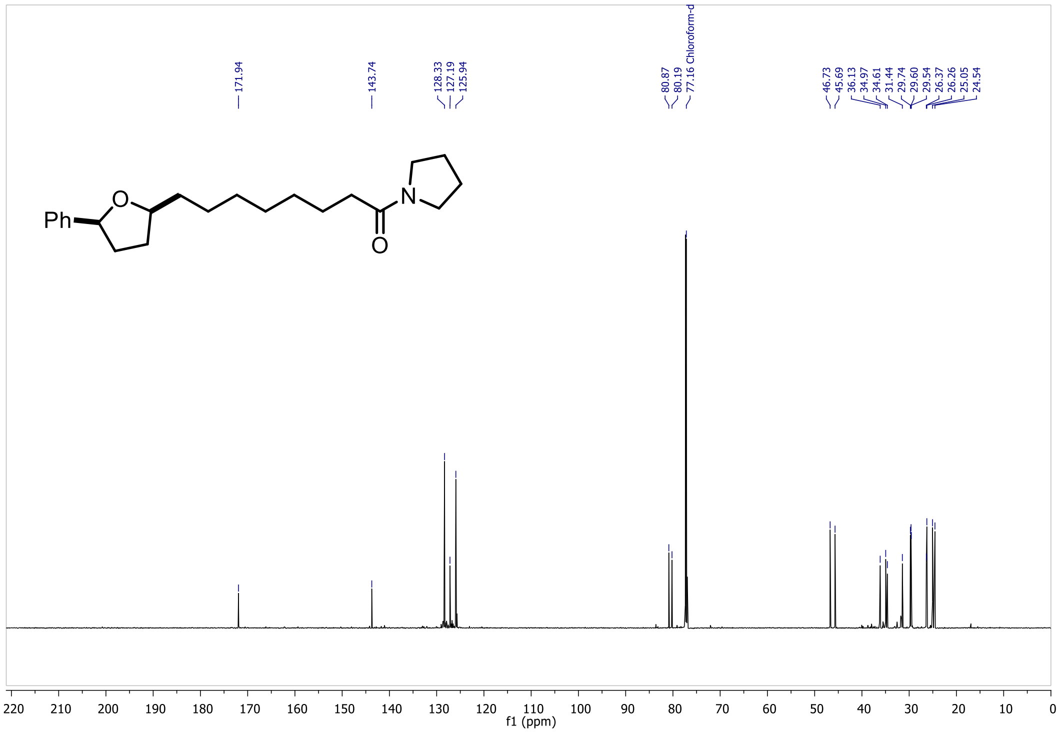

3s: *cis*-2-(3-Bromopropyl)-5-phenyltetrahydrofuran

^{1}H NMR (400 MHz, $CDCl_3$)

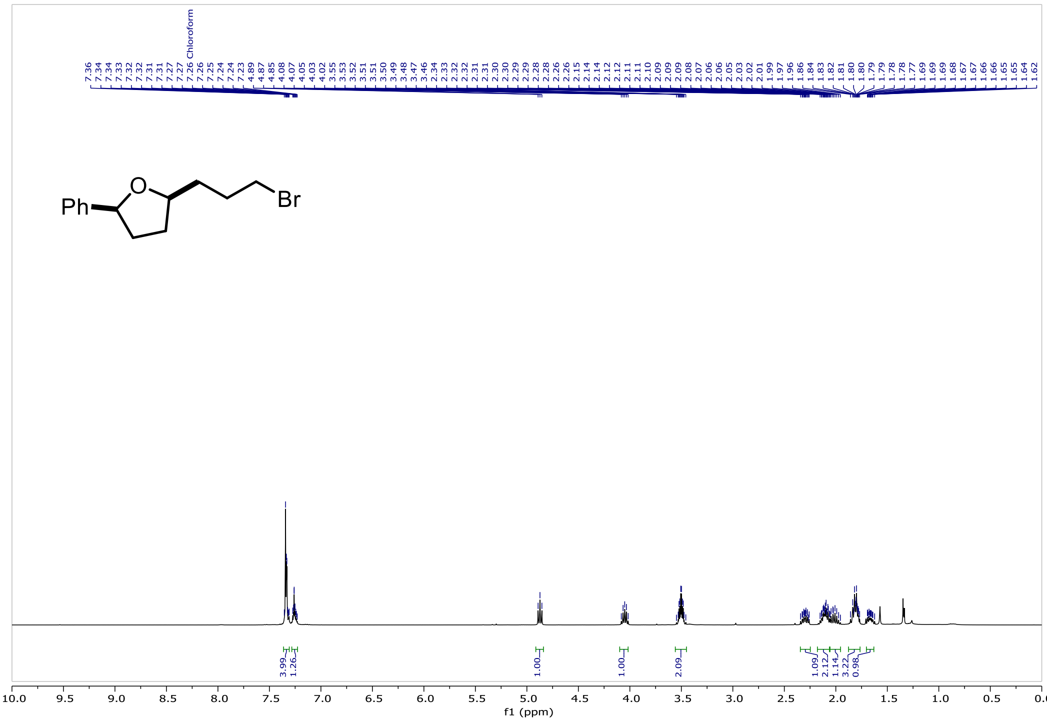

^{13}C NMR (101 MHz, $CDCl_3$)

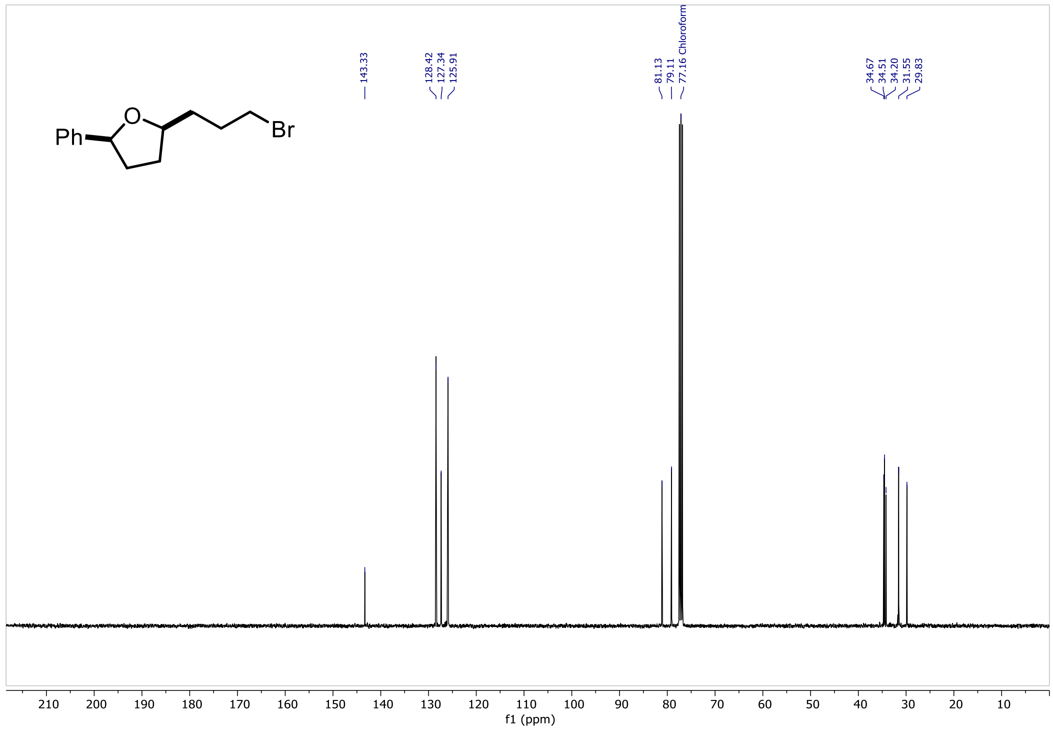

5a: 7-Phenyl-6-oxabicyclo[3.2.1]octane

^{1}H NMR (400 MHz, $CDCl_3$)

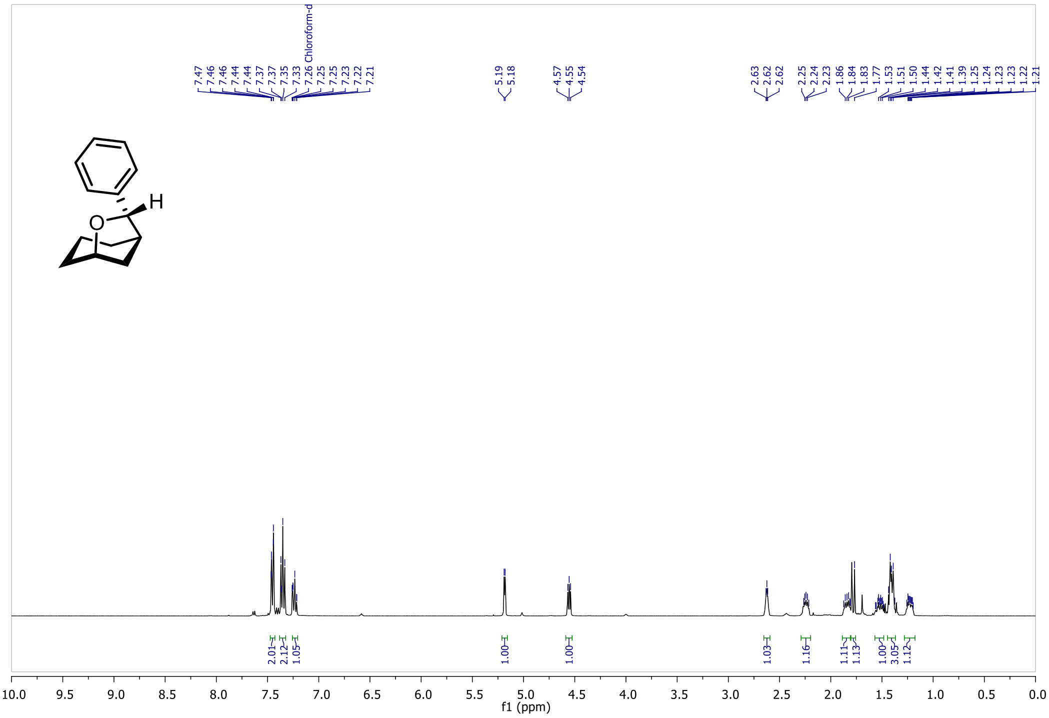

^{13}C NMR (101 MHz, $CDCl_3$)

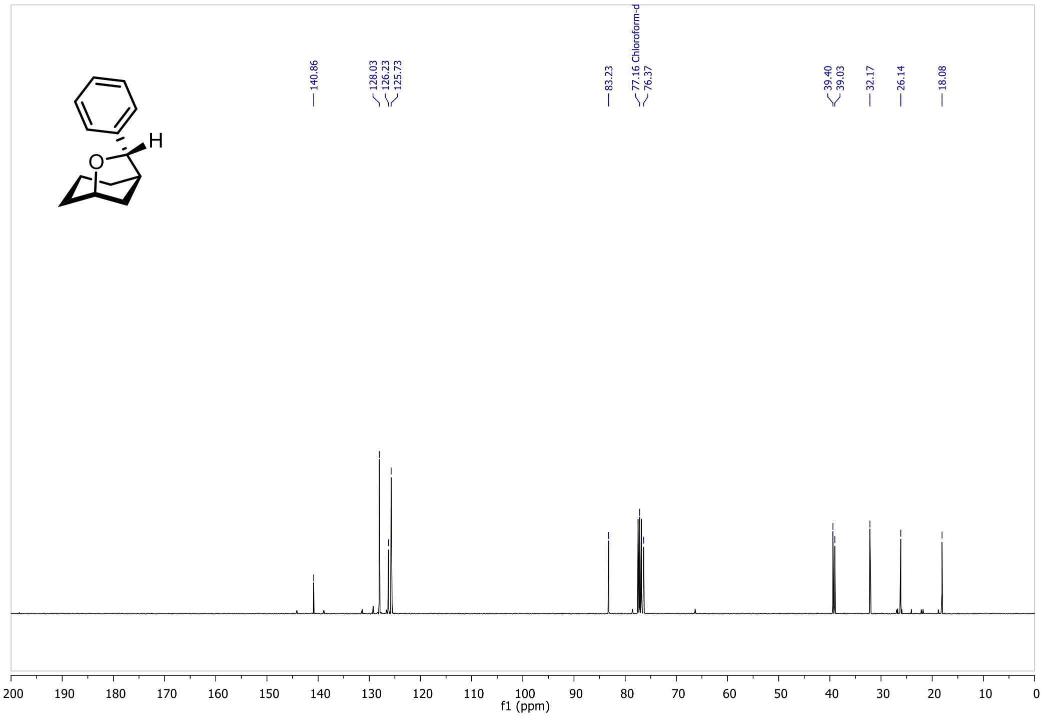

5b: 7-(4-Fluorophenyl)-6-oxabicyclo[3.2.1]octane

^{1}H NMR (400 MHz, CDCl$_3$)

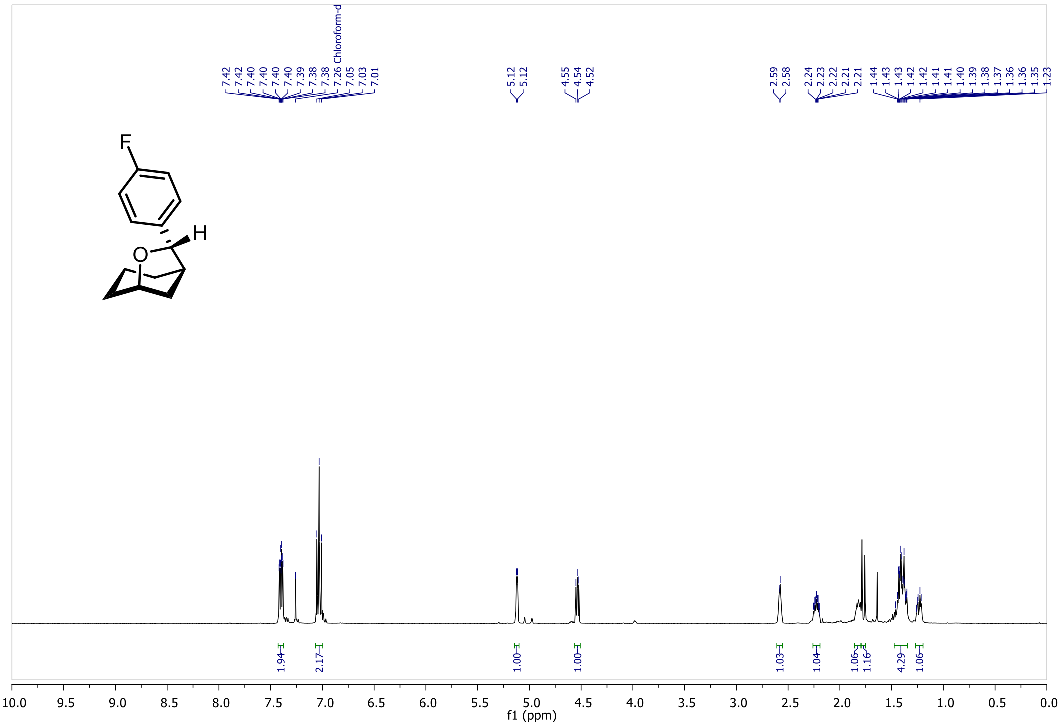

^{13}C NMR (101 MHz, CDCl$_3$)

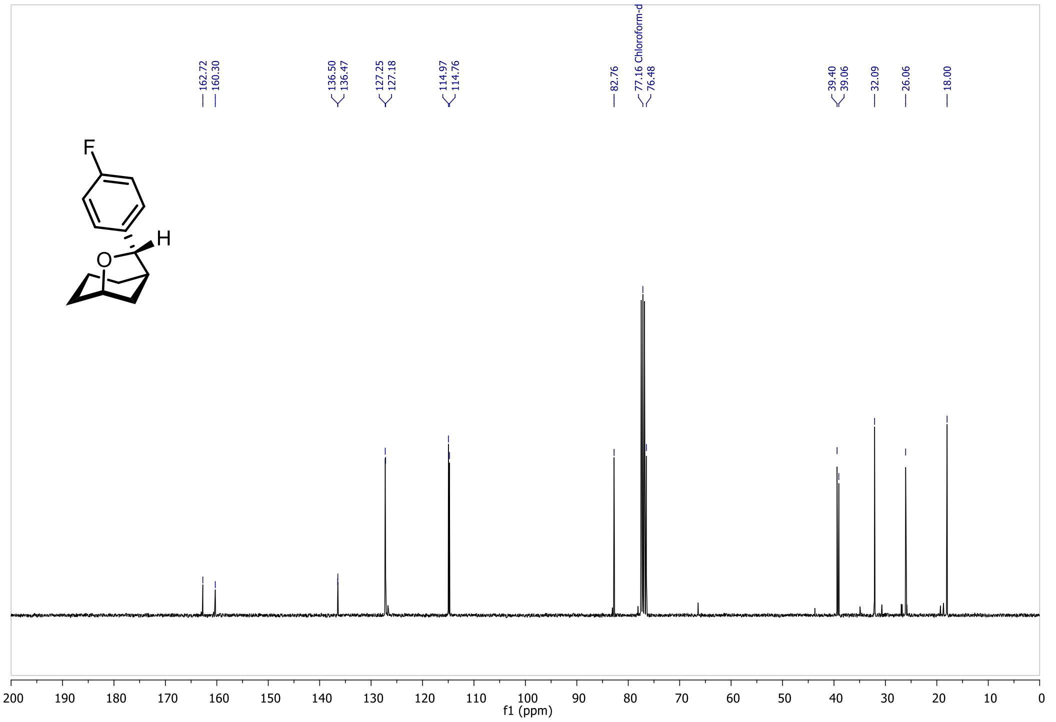

^{19}F NMR (376 MHz, $CDCl_3$)

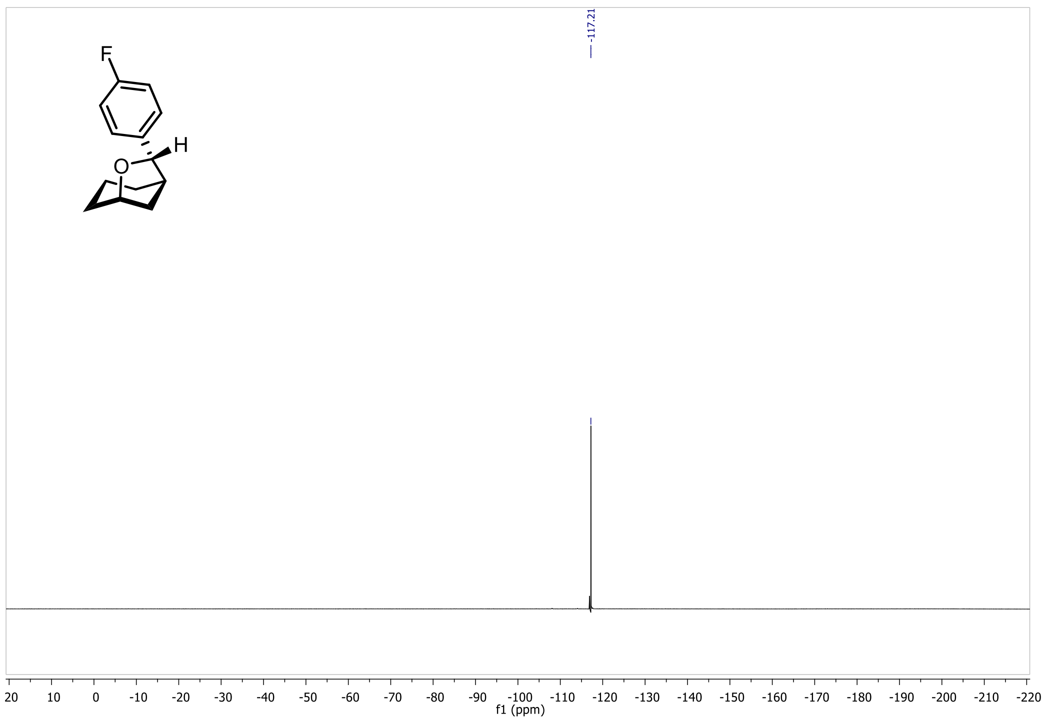

5c: 7-(3-Fluorophenyl)-6-oxabicyclo[3.2.1]octane

^{1}H NMR (400 MHz, $CDCl_3$)

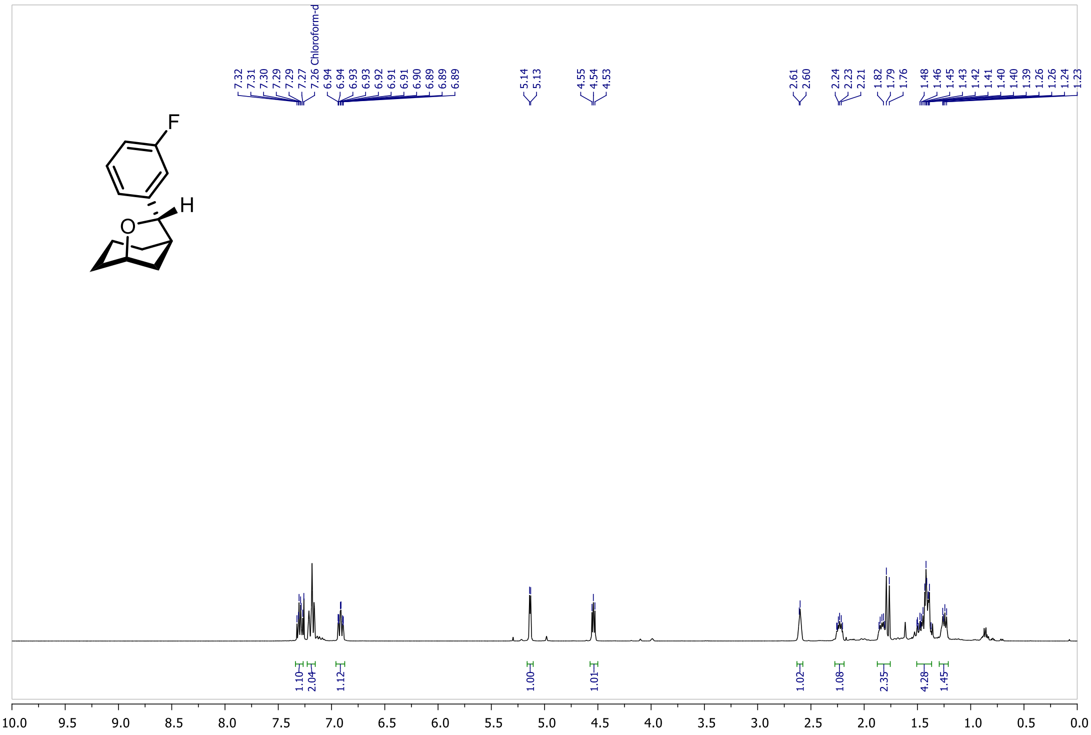

^{13}C NMR (101 MHz, $CDCl_3$)

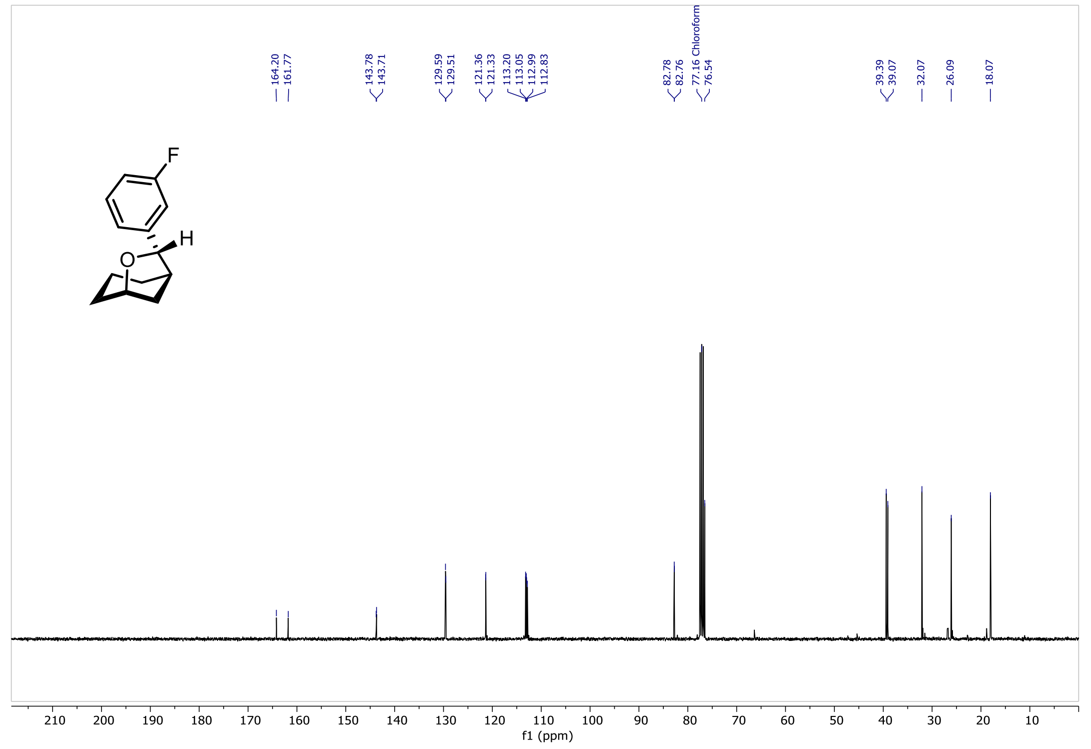

^{19}F NMR (125 MHz, $CDCl_3$)

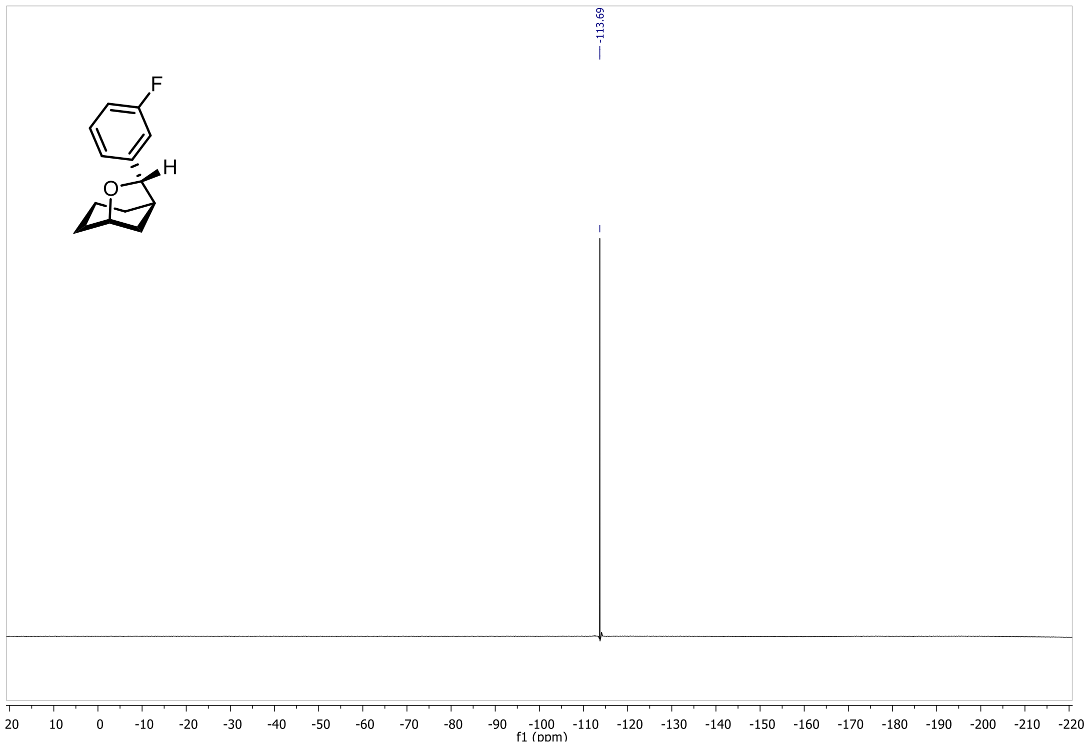

5d: 7-(4-Chlorophenyl)-6-oxabicyclo[3.2.1]octane

^{1}H NMR (400 MHz, $CDCl_3$)

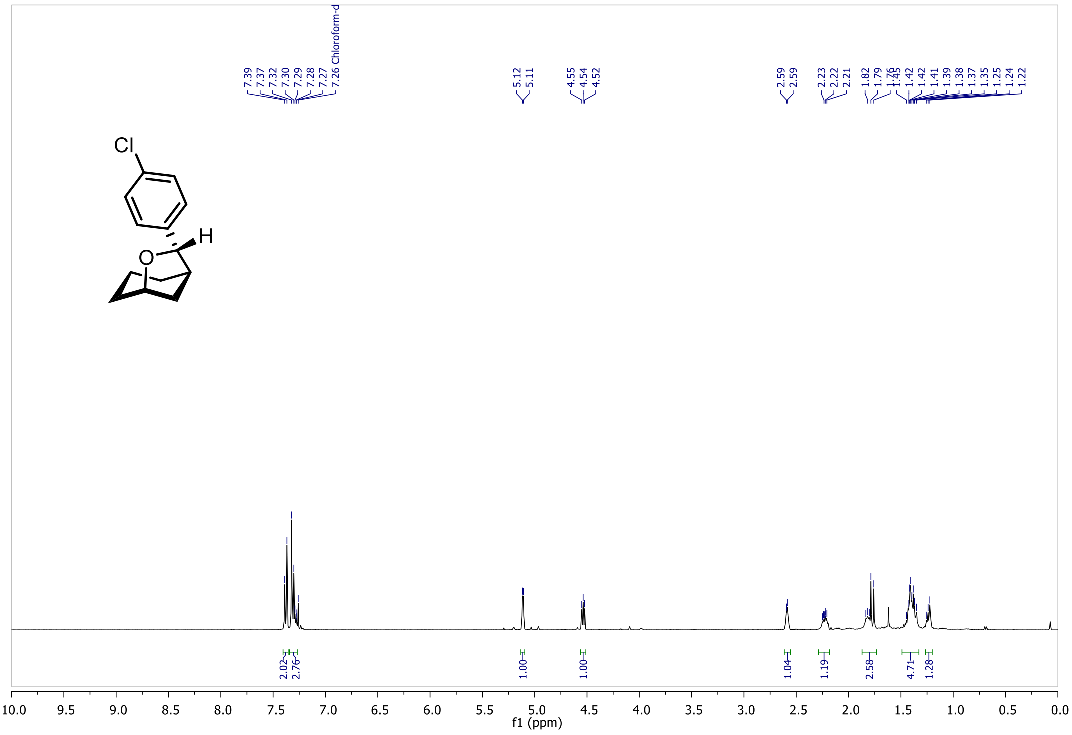

^{1}H NMR (400 MHz, $CDCl_3$)

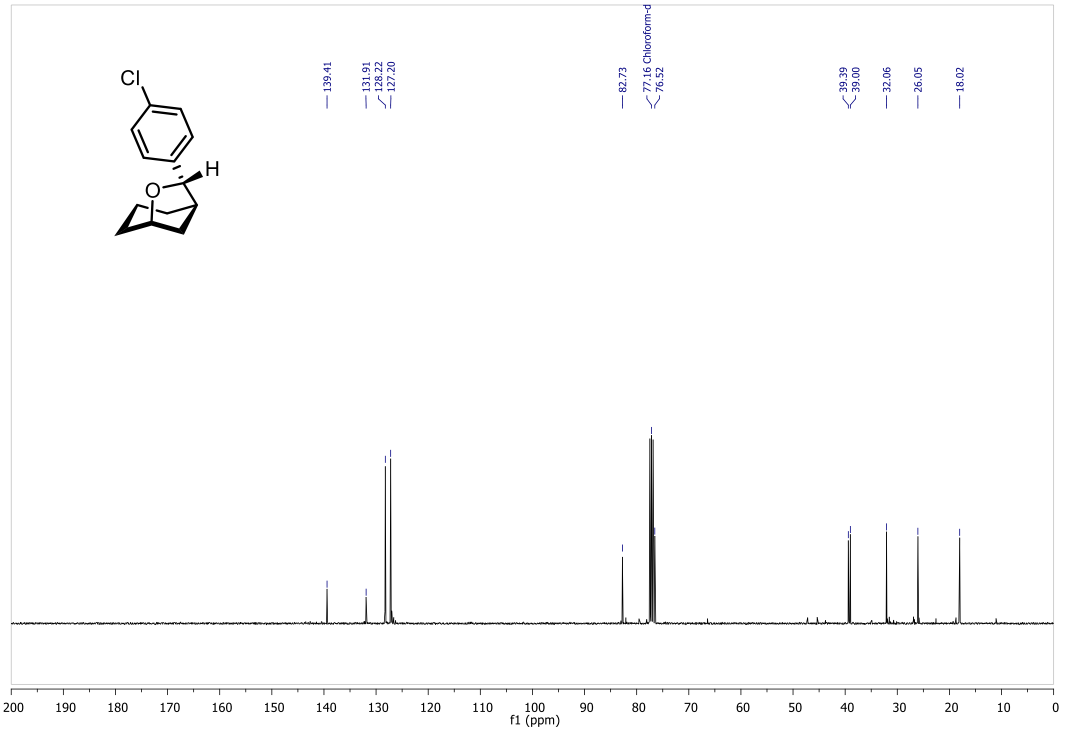

5e: 7-(2-Bromophenyl)-6-oxabicyclo[3.2.1]octane

^{1}H NMR (400 MHz, $CDCl_3$)

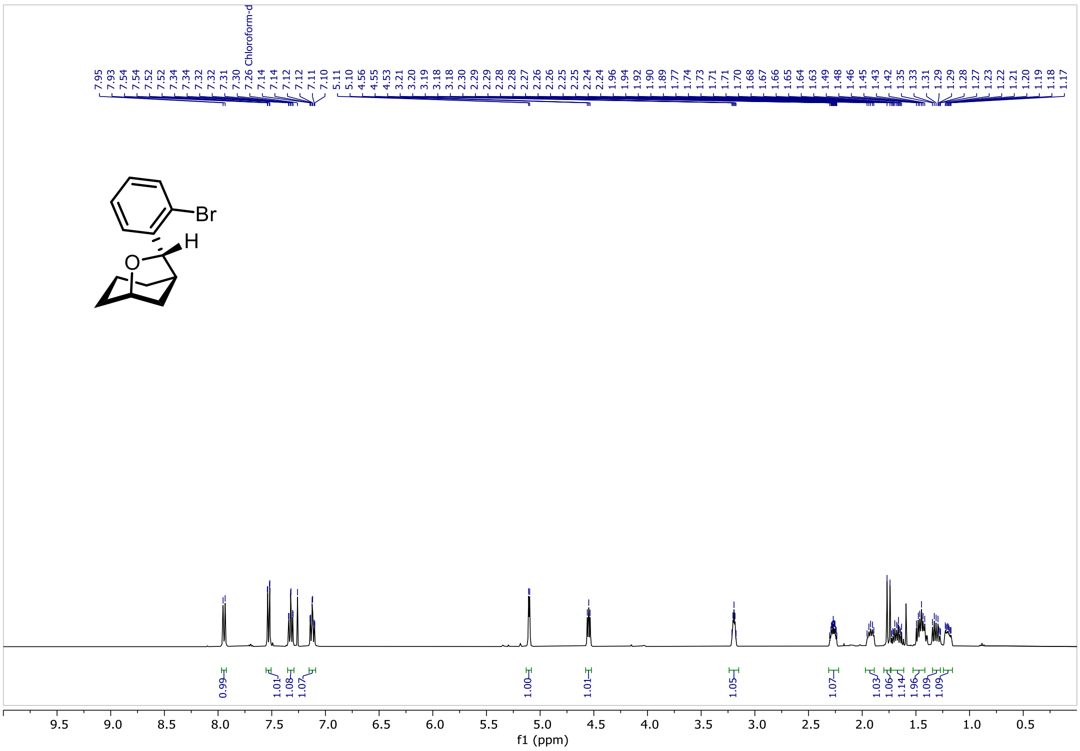

^{13}C NMR (101 MHz, $CDCl_3$)

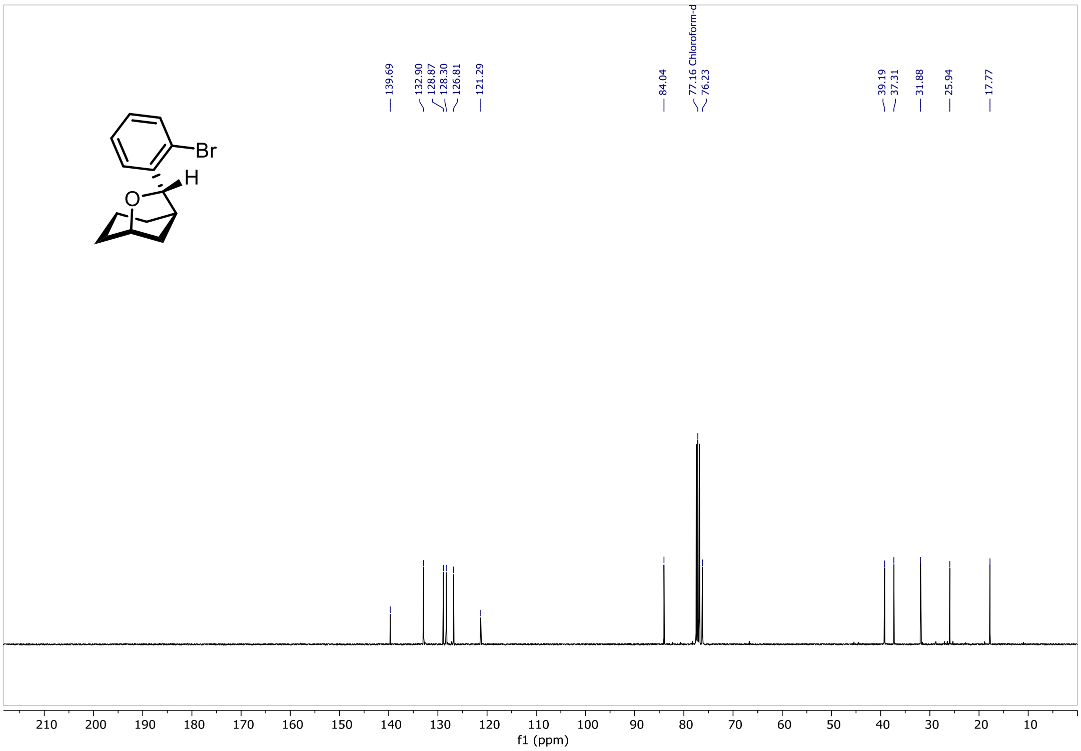

5f: 7-(2-Iodophenyl)-6-oxabicyclo[3.2.1]octane

^{1}H NMR (400 MHz, $CDCl_3$)

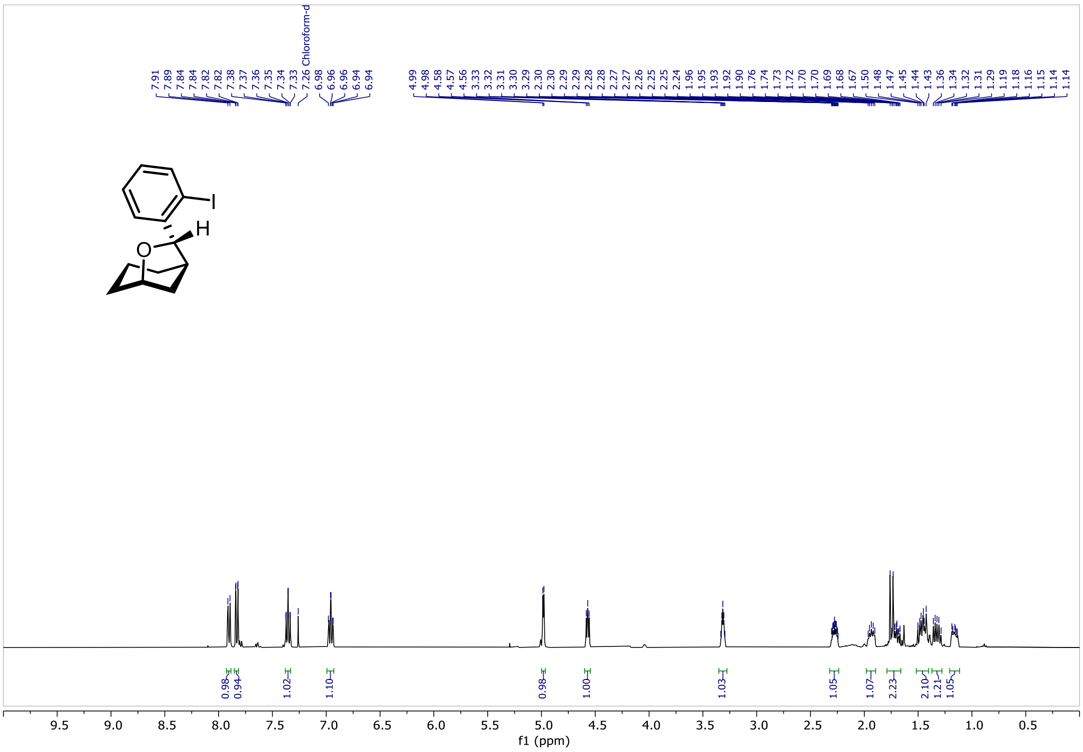

^{13}C NMR (101 MHz, $CDCl_3$)

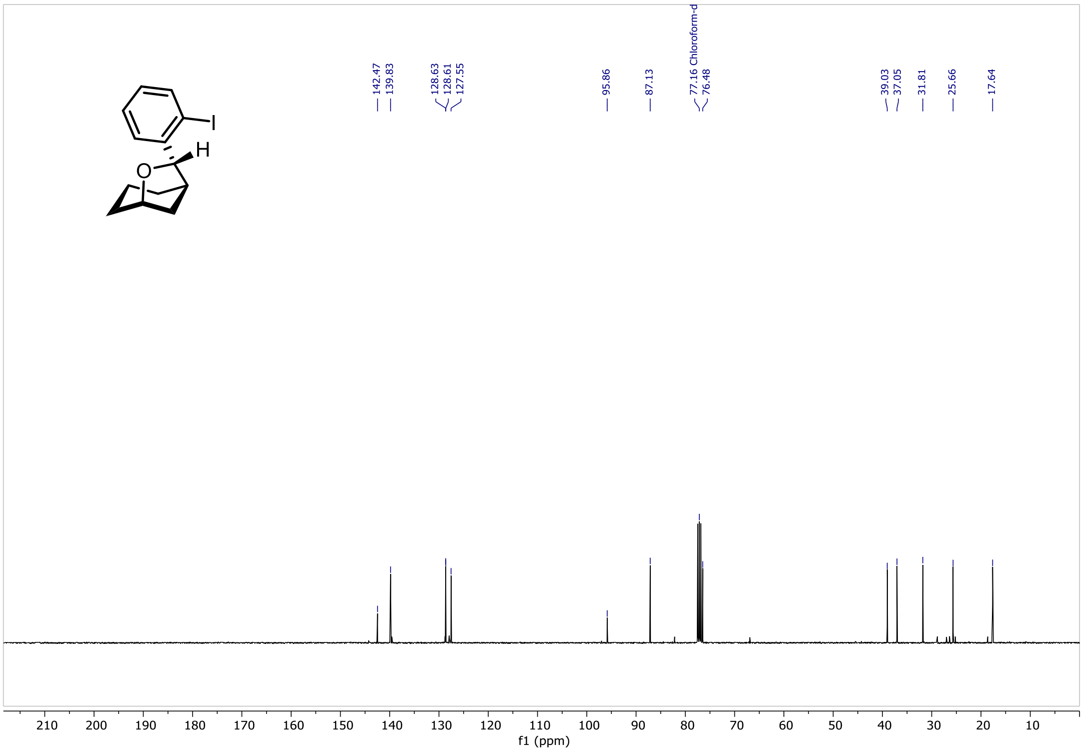

5g: 7-(3,4-Dichlorophenyl)-6-oxabicyclo[3.2.1]octane

^{1}H NMR (400 MHz, $CDCl_3$)

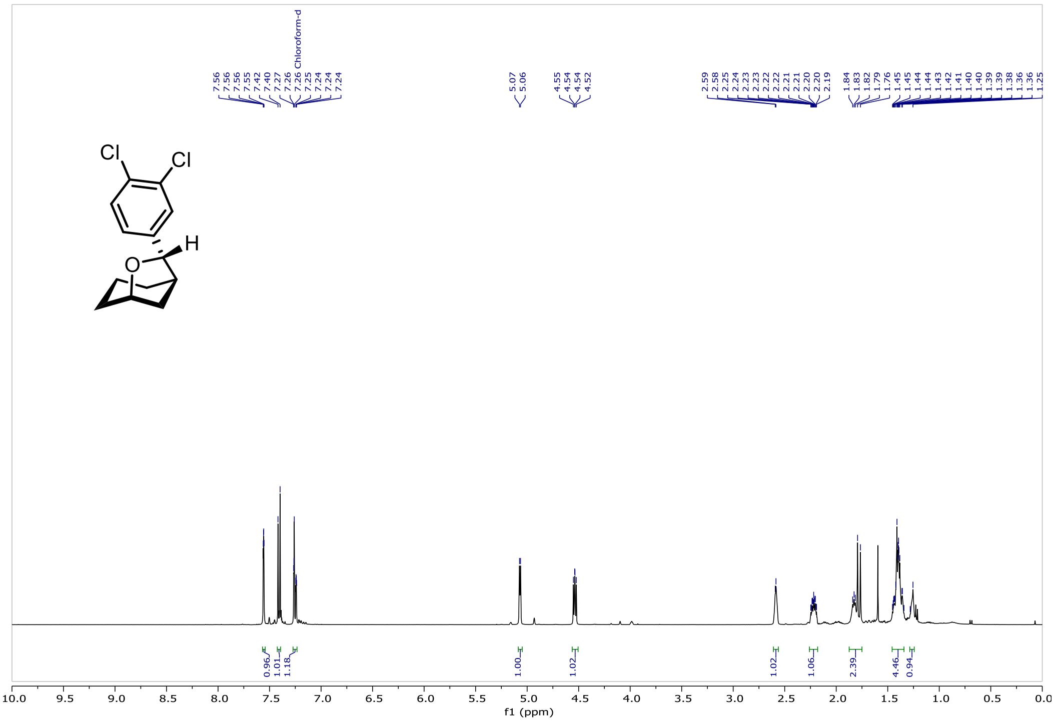

^{13}C NMR (101 MHz, $CDCl_3$)

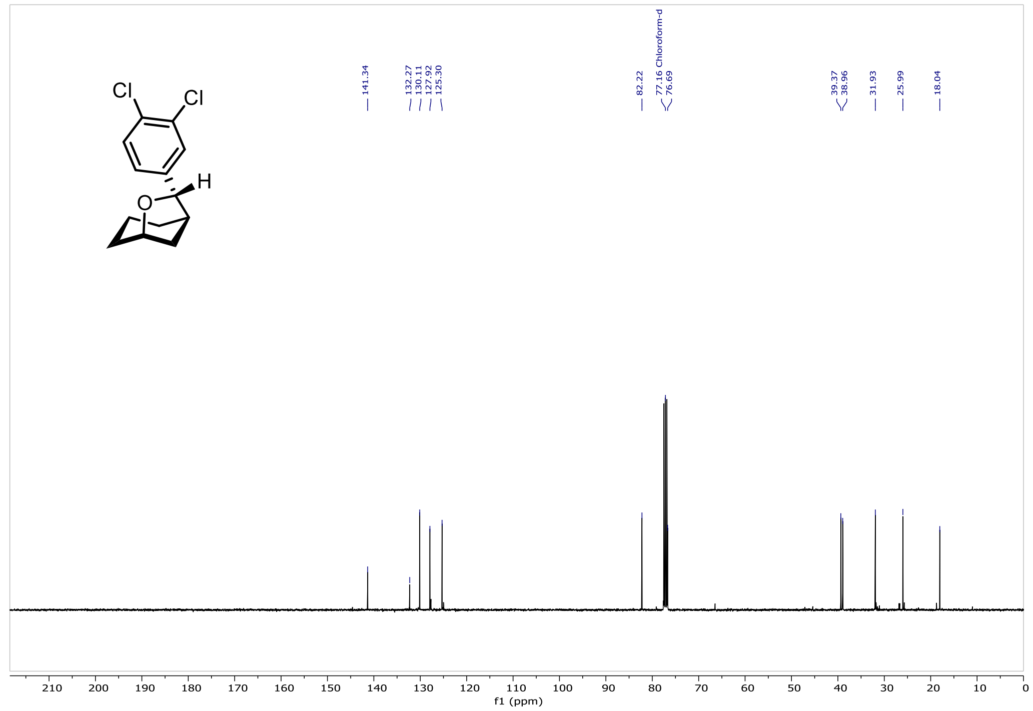

5h: 7-(4-(Trifluoromethyl)phenyl)-6-oxabicyclo[3.2.1]octane

^{1}H NMR (400 MHz, $CDCl_3$)

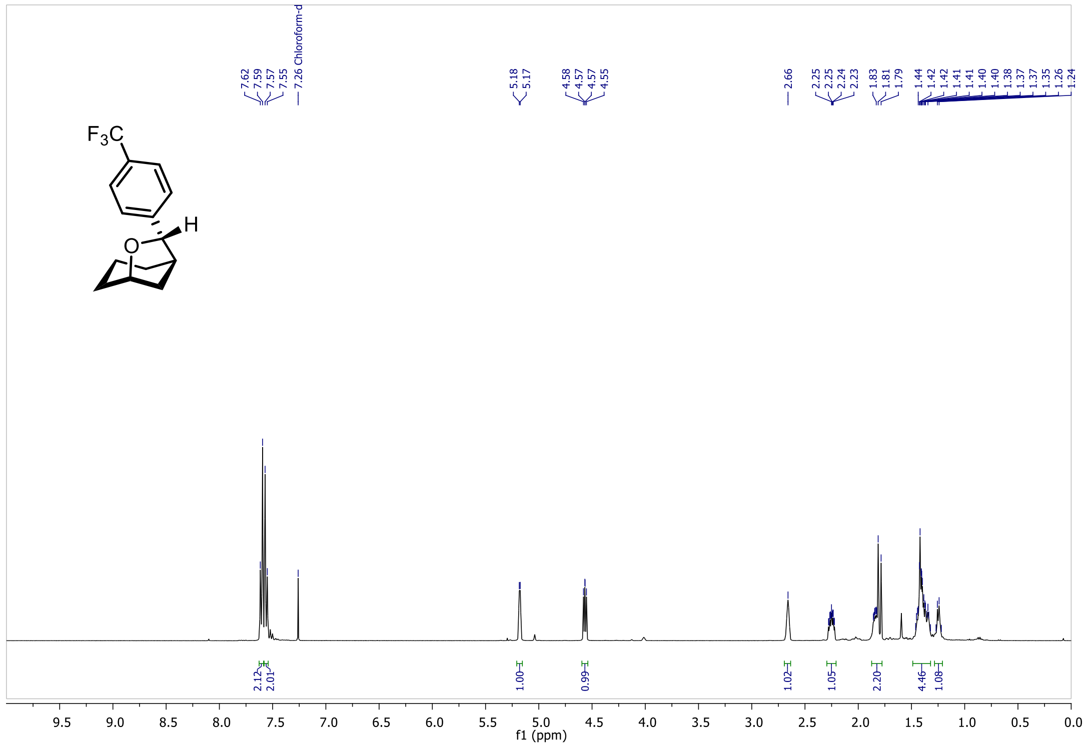

^{13}C NMR (101 MHz, $CDCl_3$)

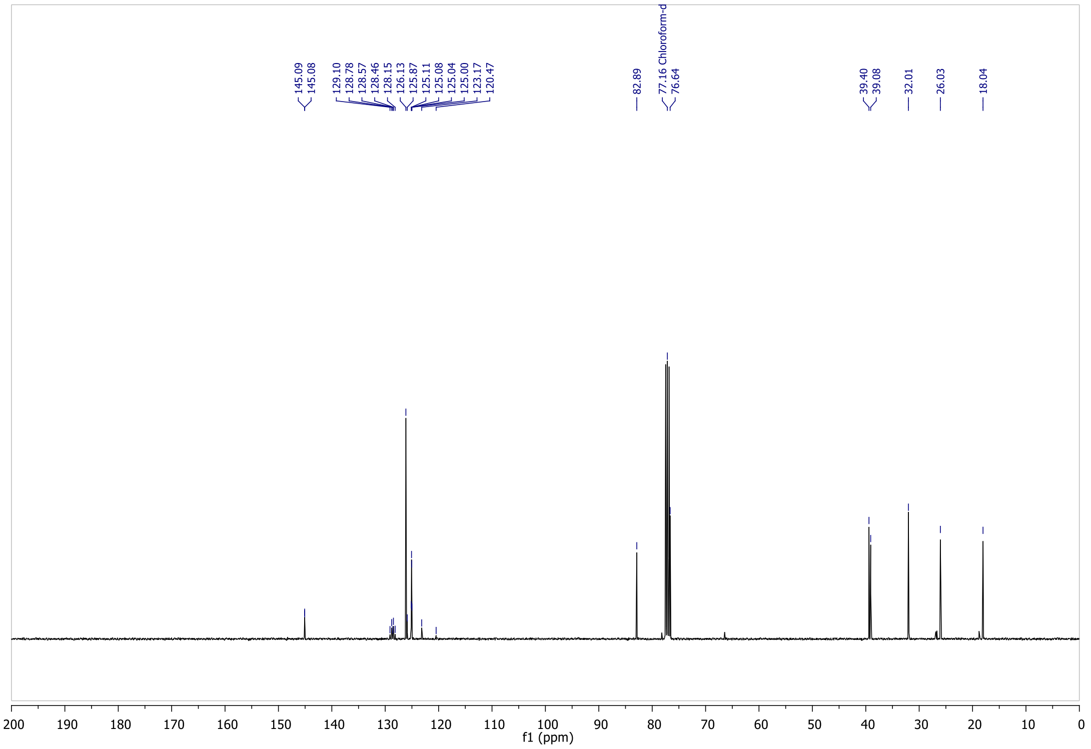

^{19}F NMR (377 MHz, $CDCl_3$)

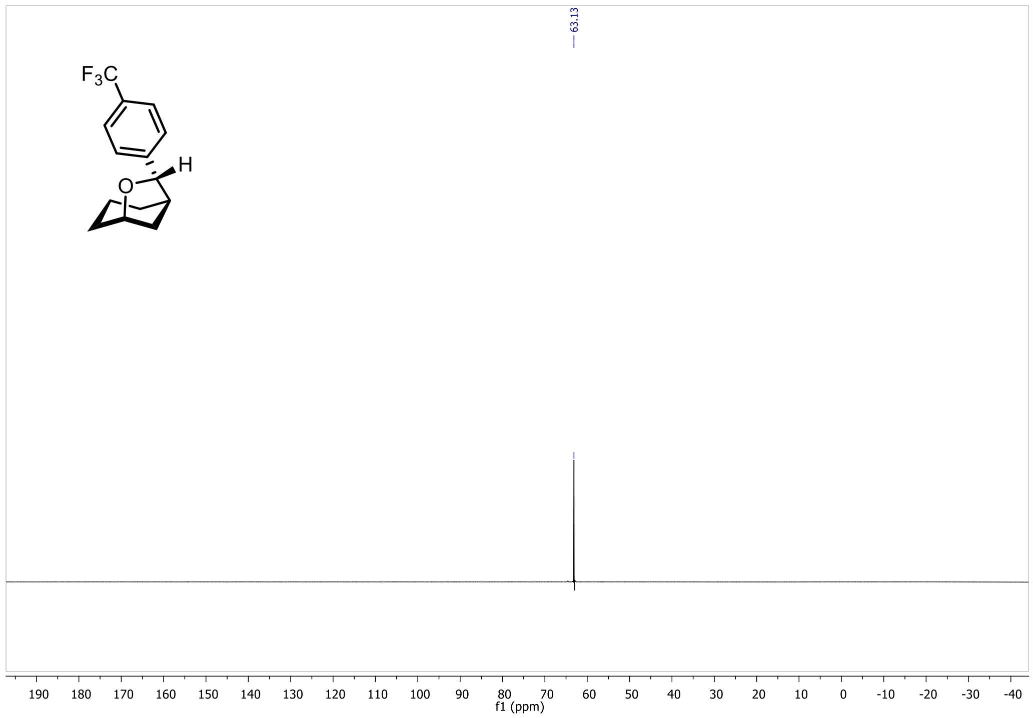

5i: 7-(4-Nitrophenyl)-6-oxabicyclo[3.2.1]octane

^1H NMR (400 MHz, $CDCl_3$)

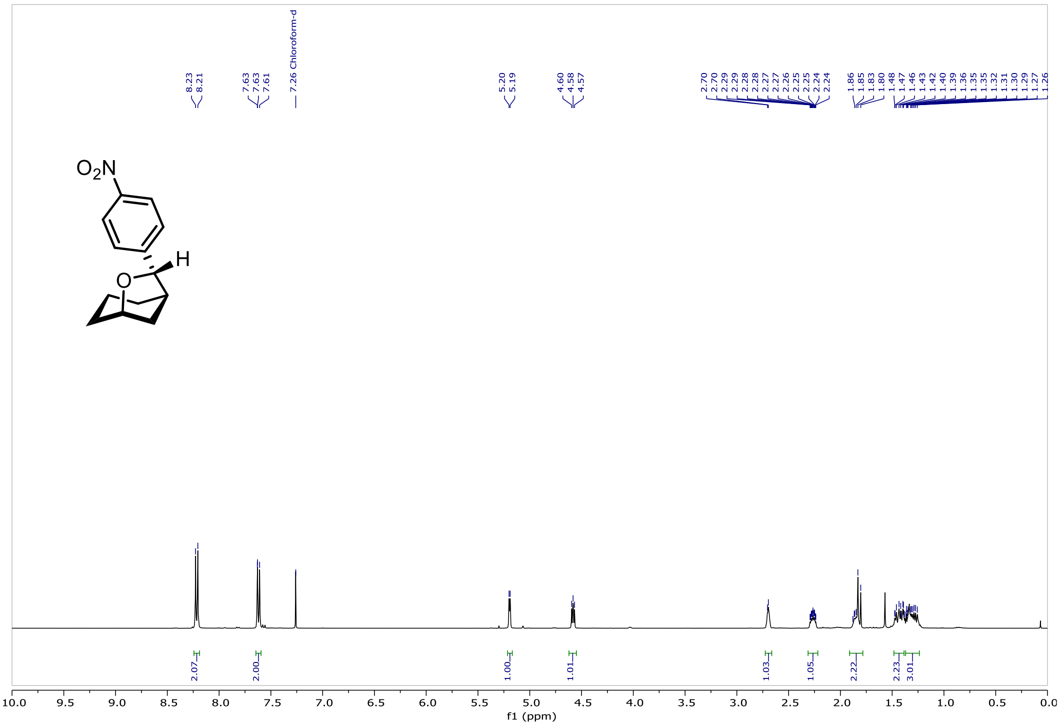

^{13}C NMR (101 MHz, $CDCl_3$)

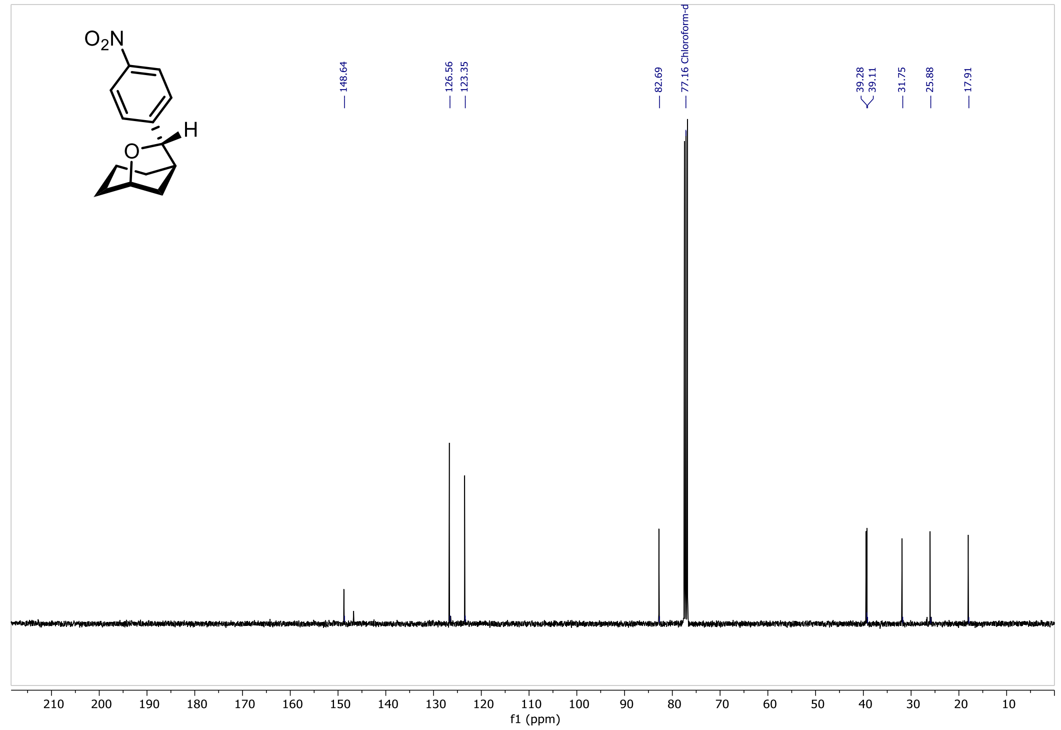

5j: Methyl 4-(6-oxabicyclo[3.2.1]octan-7-yl)benzoate

^{1}H NMR (400 MHz, $CDCl_3$)

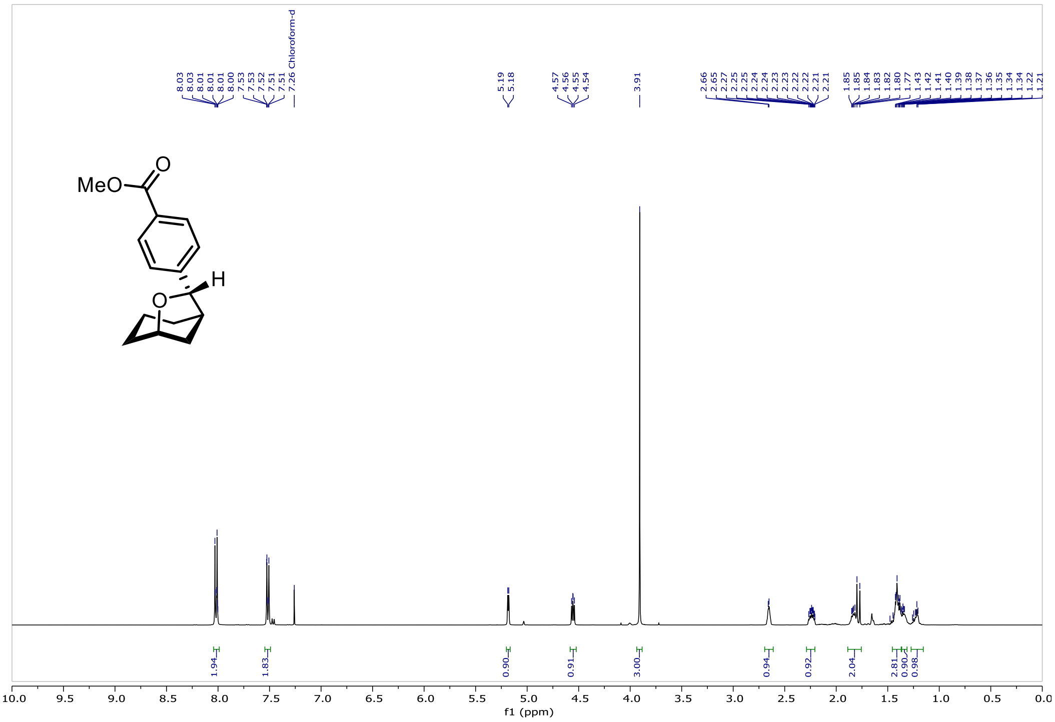

^{13}C NMR (101 MHz, $CDCl_3$)

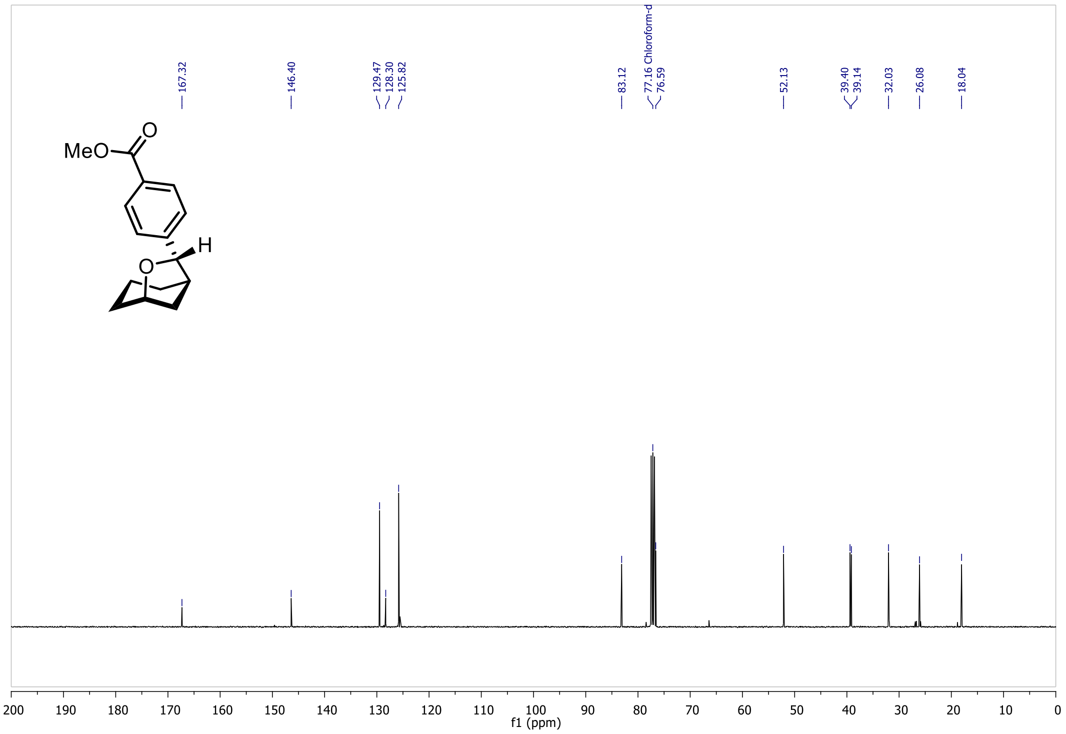

5k: 7-(4-(Trifluoromethoxy)phenyl)-6-oxabicyclo[3.2.1]octane

^{1}H NMR (400 MHz, $CDCl_3$)

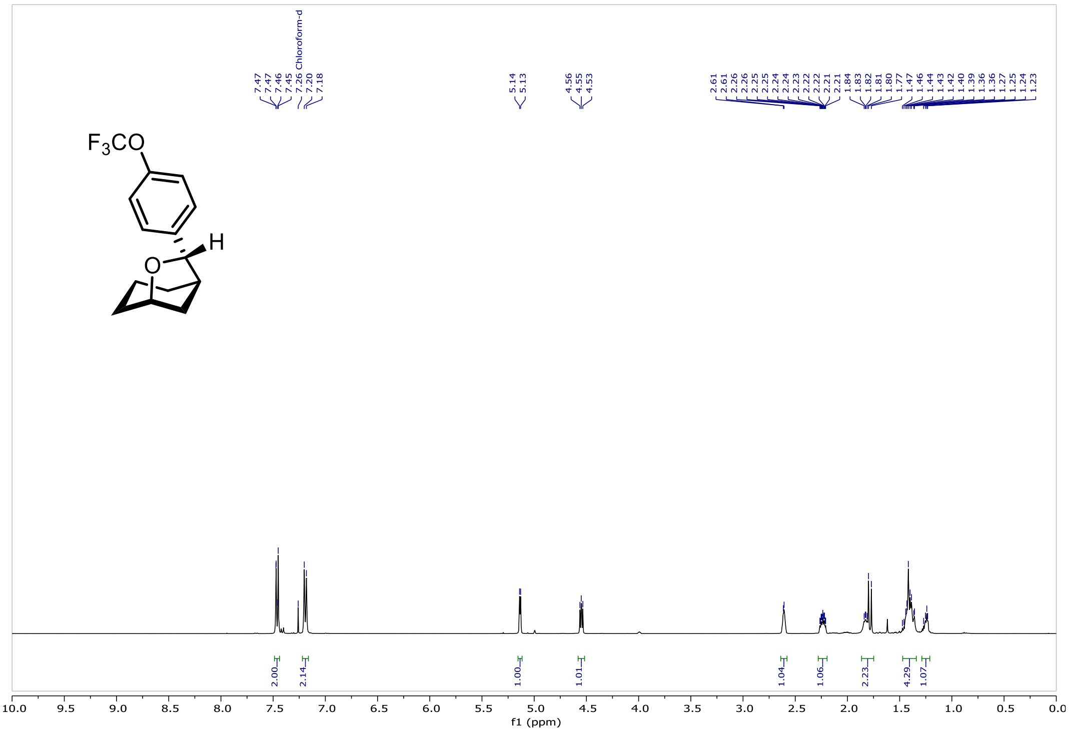

^{13}C NMR (101 MHz, $CDCl_3$)

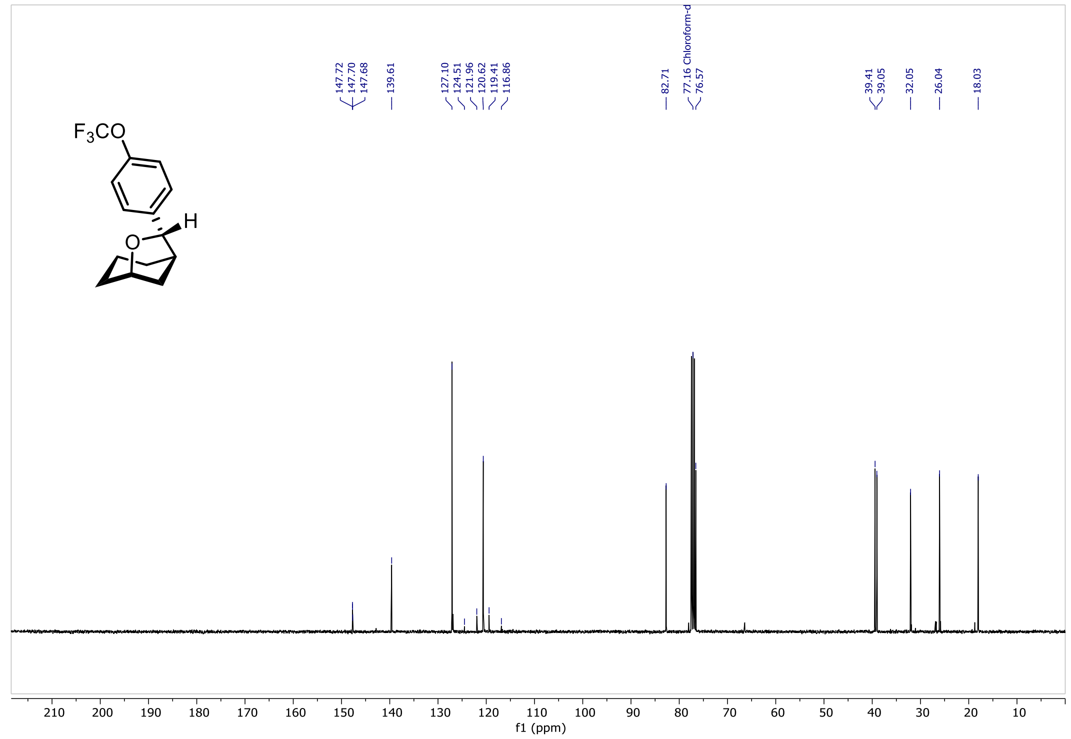

^{19}F NMR (376 MHz, $CDCl_3$)

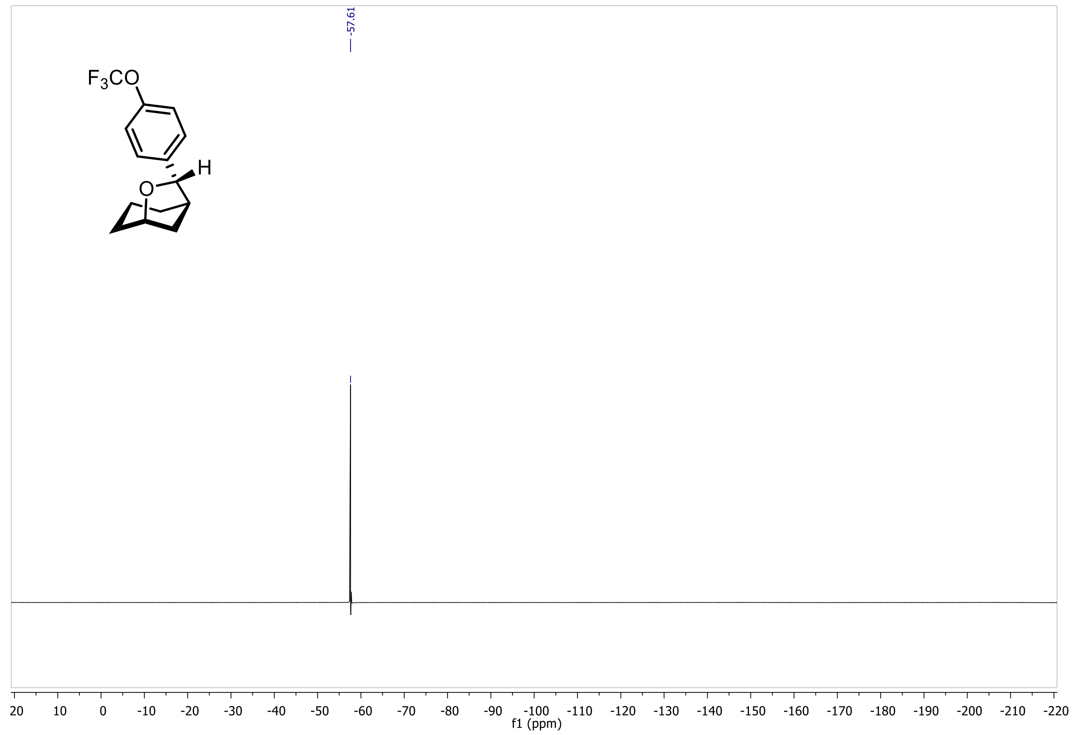

5l: 7-(3-Methoxyphenyl)-6-oxabicyclo[3.2.1]octane

^{1}H NMR (400 MHz, $CDCl_3$)

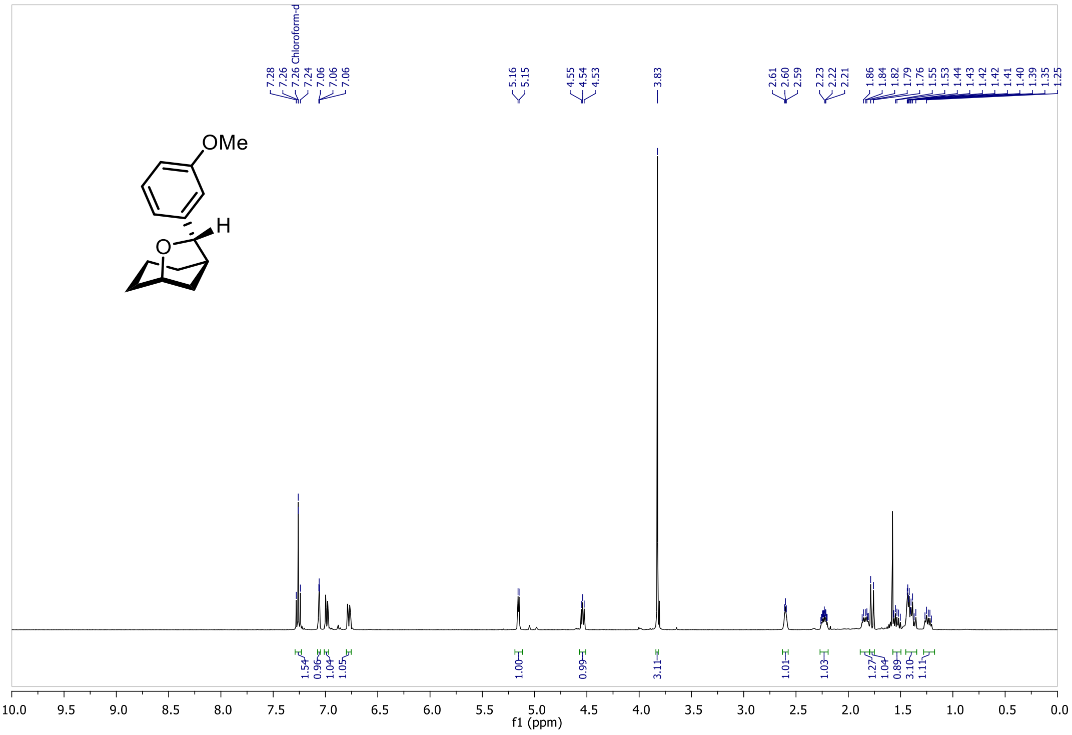

^{13}C NMR (101 MHz, $CDCl_3$)

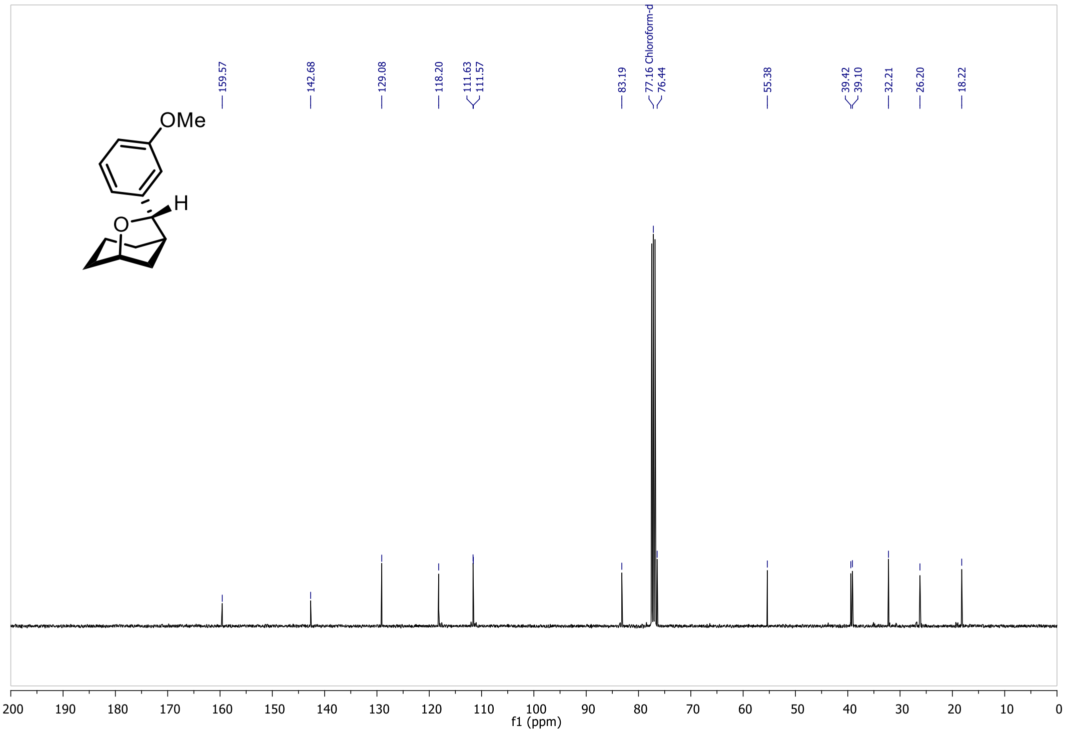

5m: 7-(4-(*tert*-Butyl)phenyl)-6-oxabicyclo[3.2.1]octane

^{1}H NMR (400 MHz, $CDCl_3$)

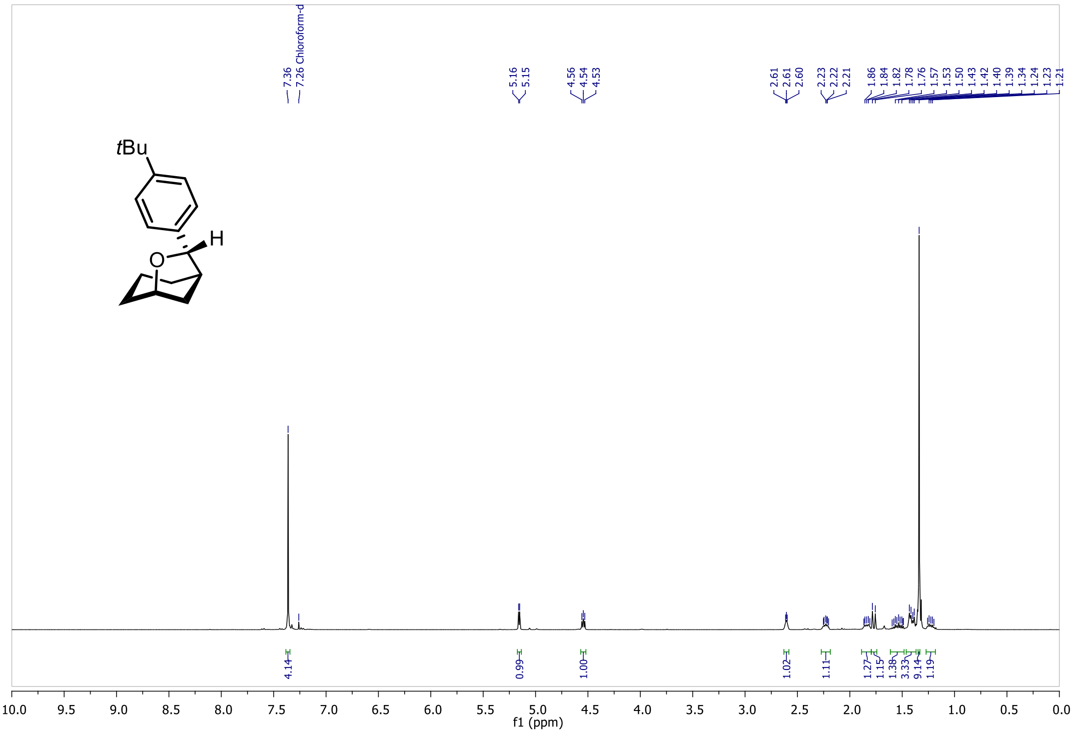

^{13}C NMR (101 MHz, $CDCl_3$)

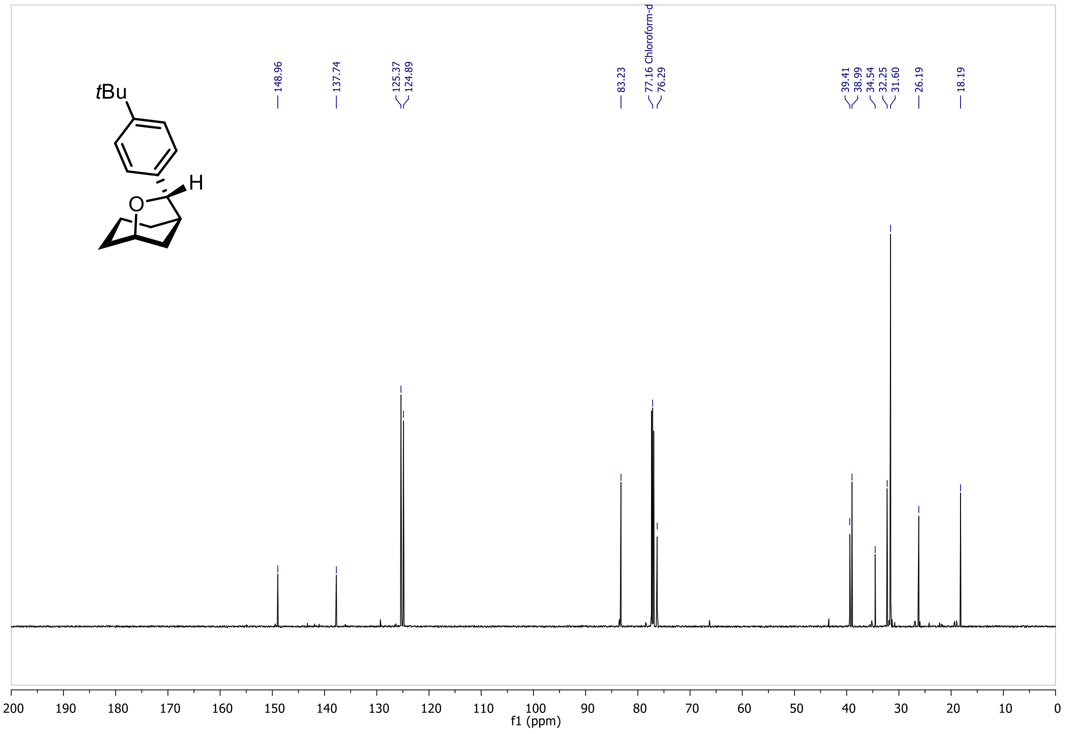

5n: 7-(3,5-Dimethylphenyl)-6-oxabicyclo[3.2.1]octane

^{1}H NMR (400 MHz, $CDCl_3$)

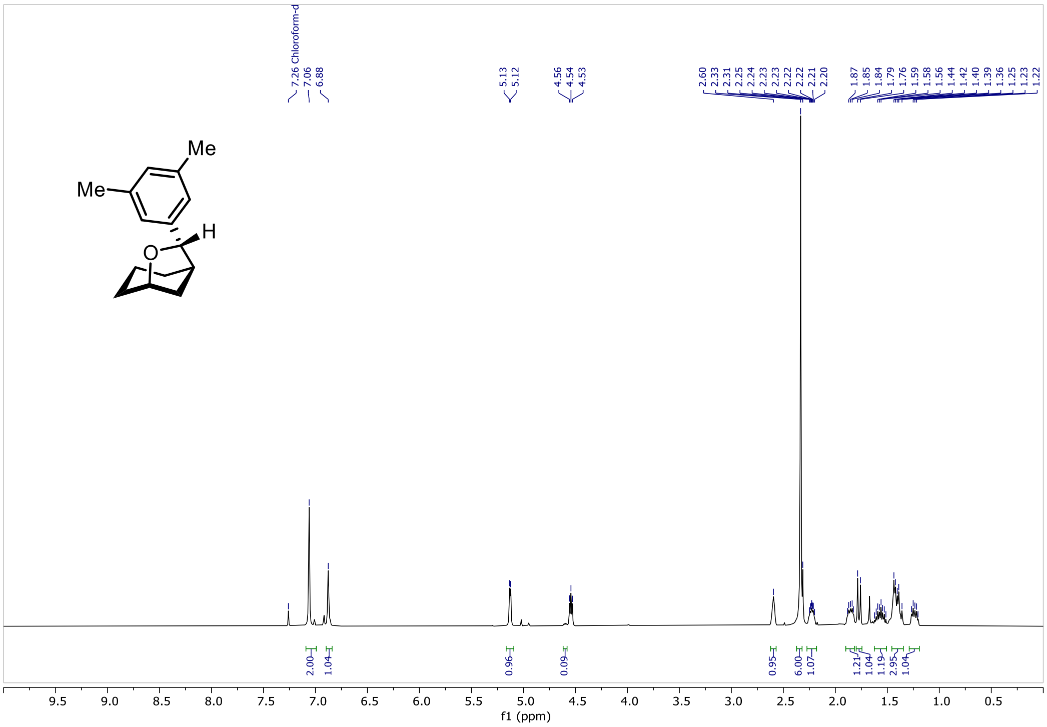

^{13}C NMR (101 MHz, $CDCl_3$)

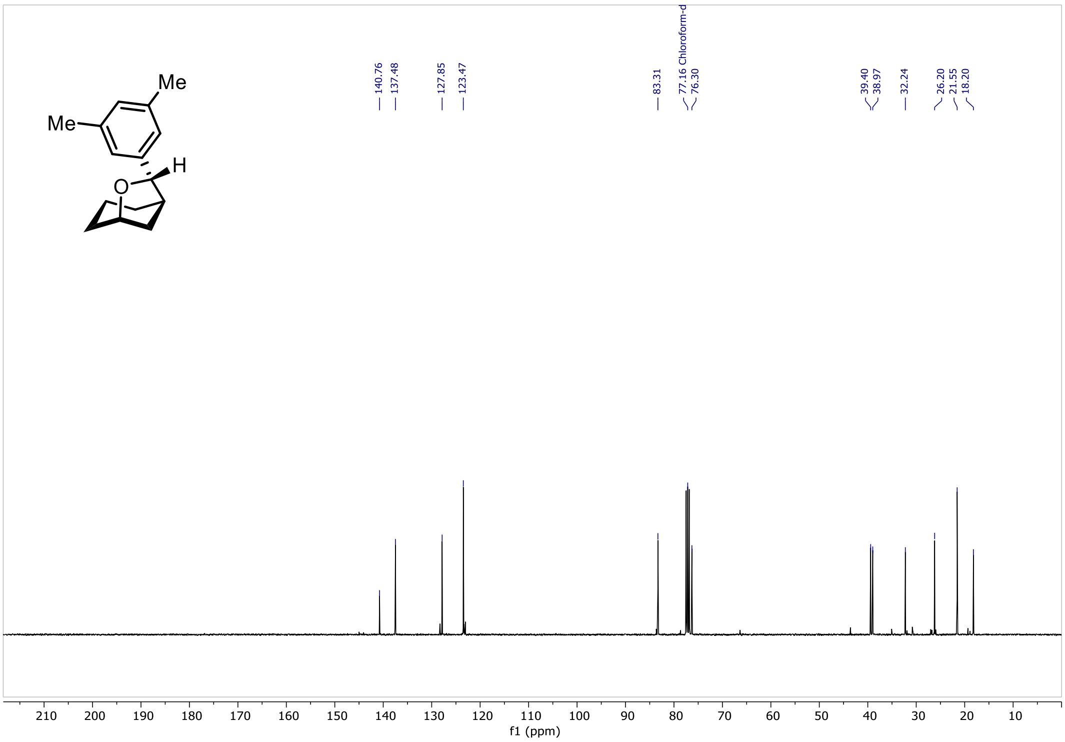

5o: 7-(Naphthalen-2-yl)-6-oxabicyclo[3.2.1]octane

^{1}H NMR (400 MHz, $CDCl_3$)

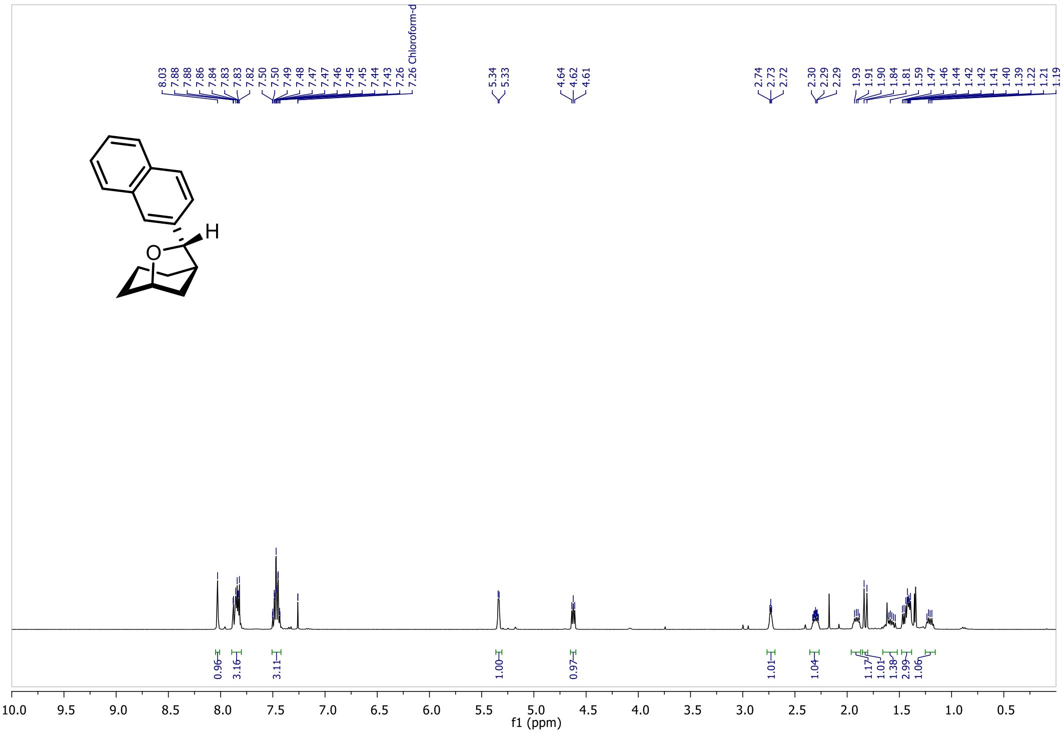

^{13}C NMR (101 MHz, $CDCl_3$)

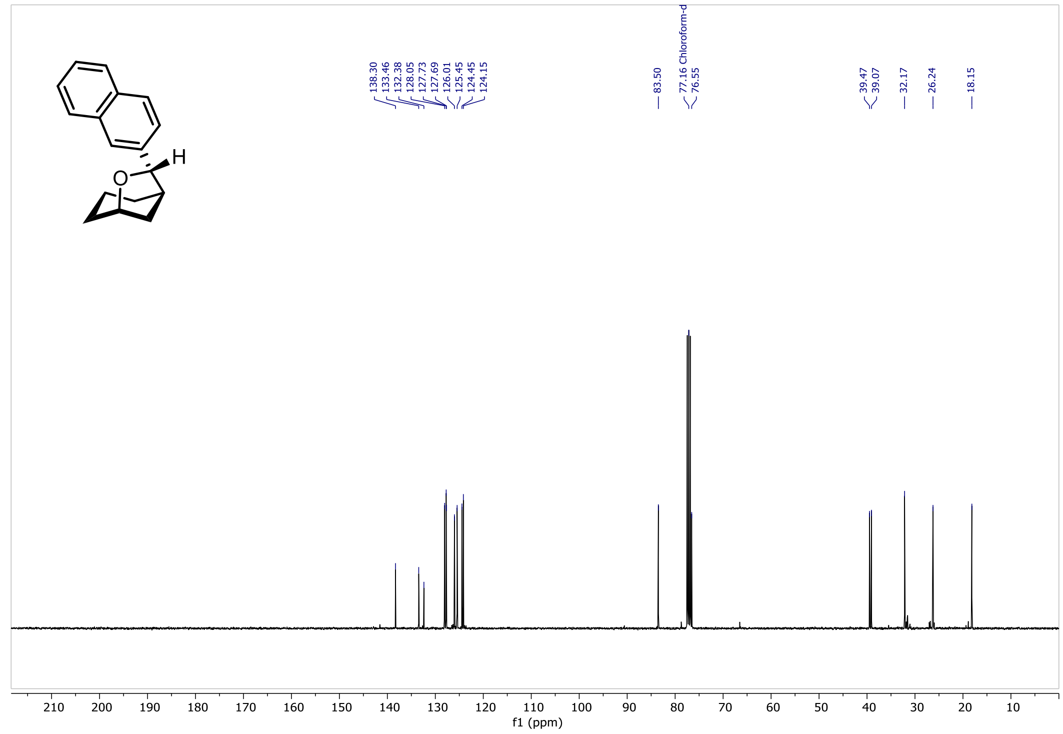

5p: 7-Pentadecyl-6-oxabicyclo[3.2.1]octane

^{1}H NMR (400 MHz, $CDCl_3$)

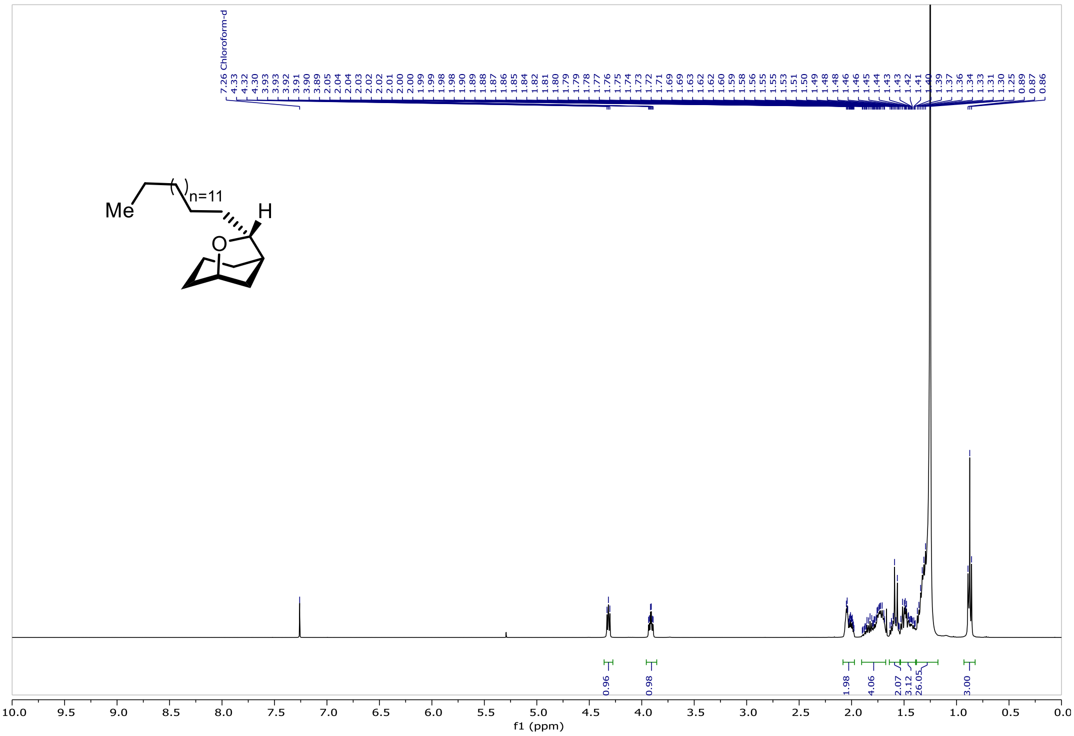

^{13}C NMR (101 MHz, $CDCl_3$)

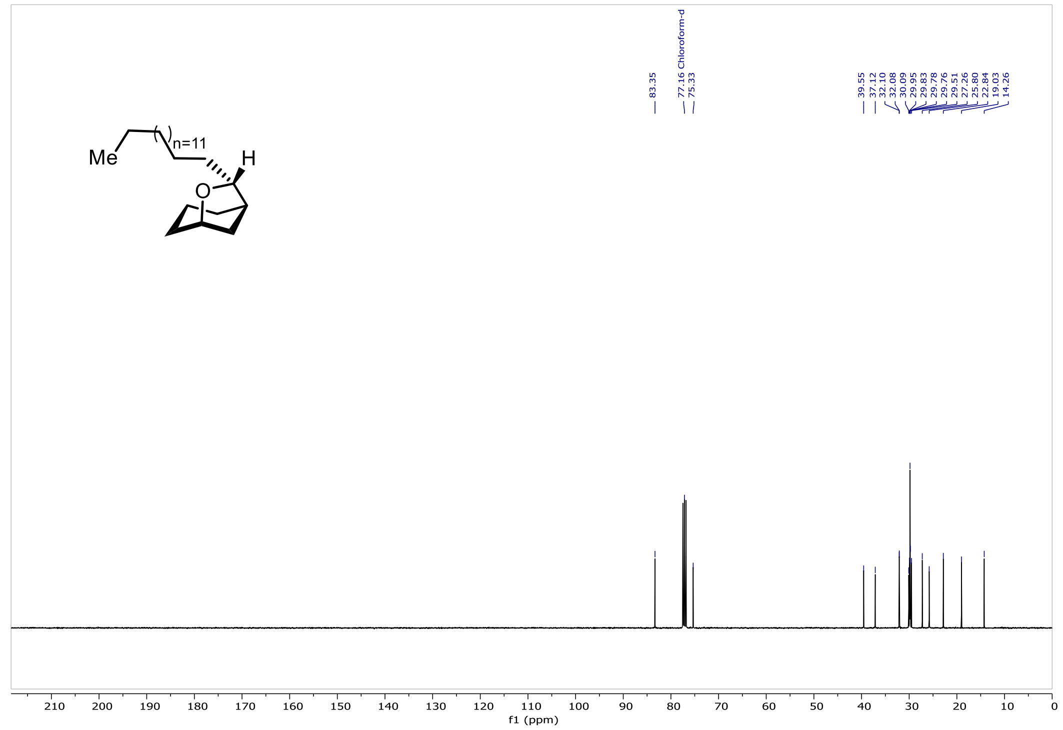

5q: 7-Undecyl-6-oxabicyclo[3.2.1]octane

^{1}H NMR (400 MHz, $CDCl_3$)

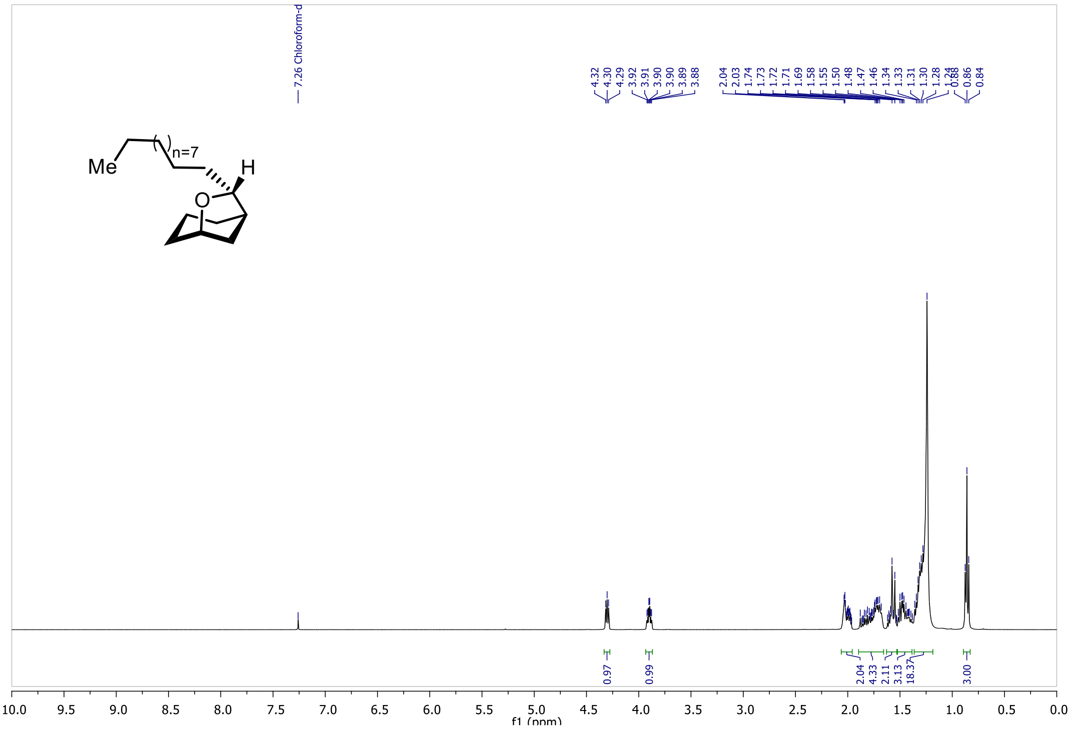

^{13}C NMR (101 MHz, $CDCl_3$)

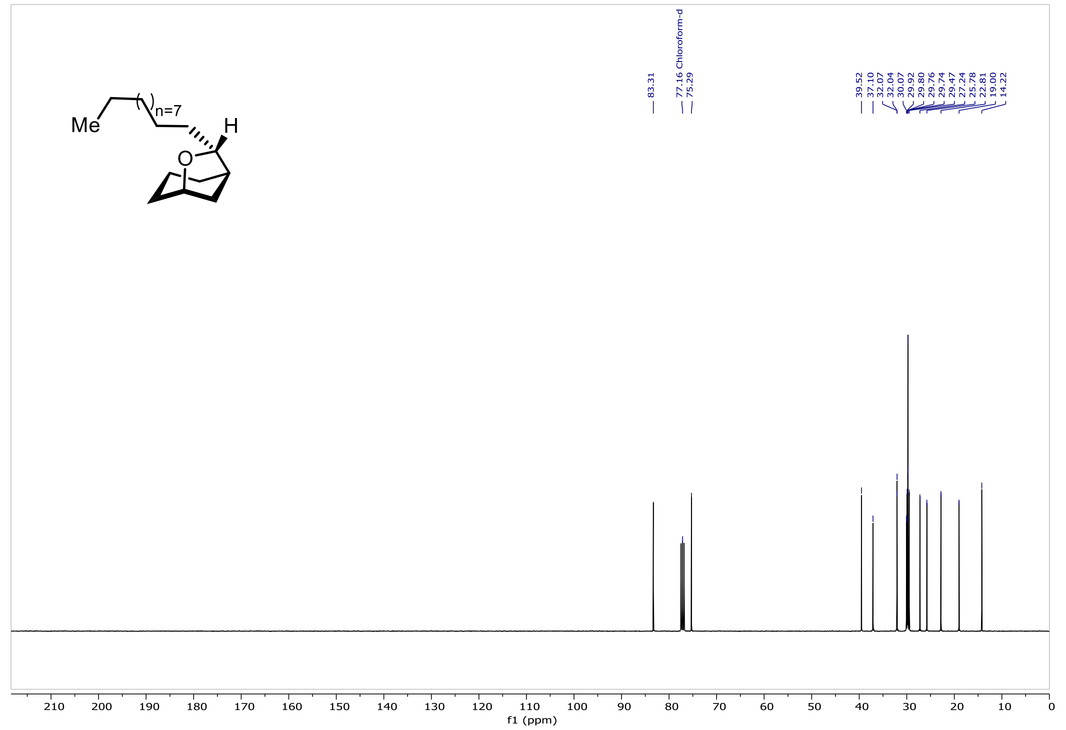

5r: 7-Phenethyl-6-oxabicyclo[3.2.1]octane

^{1}H NMR (400 MHz, $CDCl_3$)

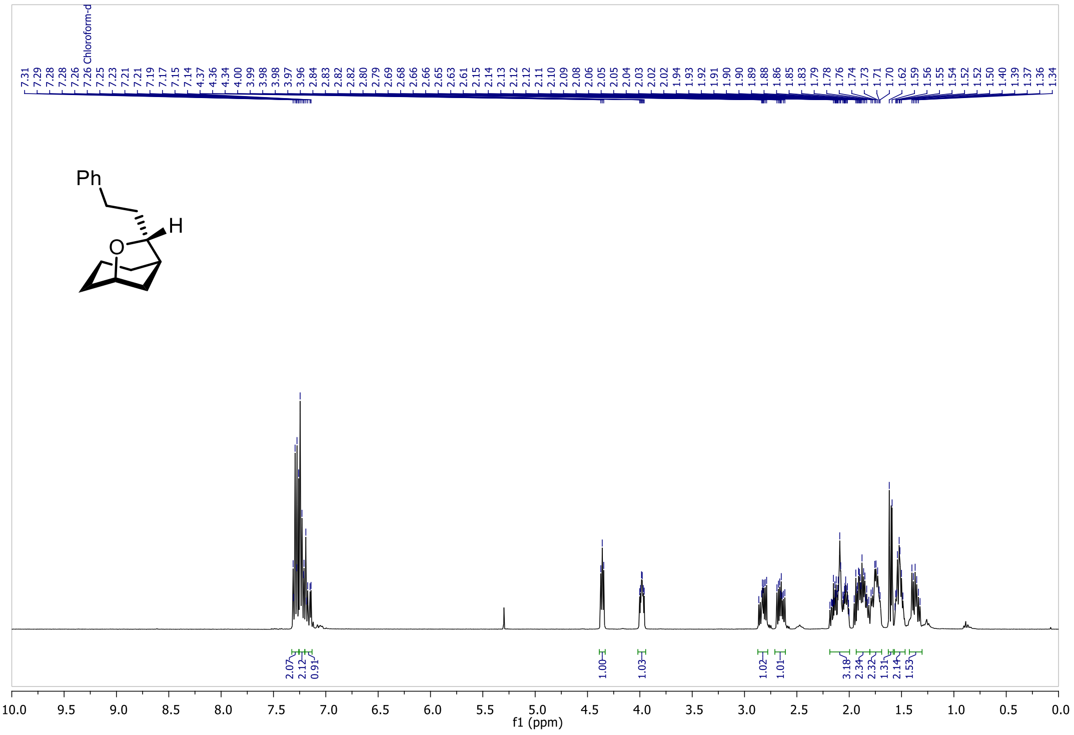

^{13}C NMR (101 MHz, $CDCl_3$)

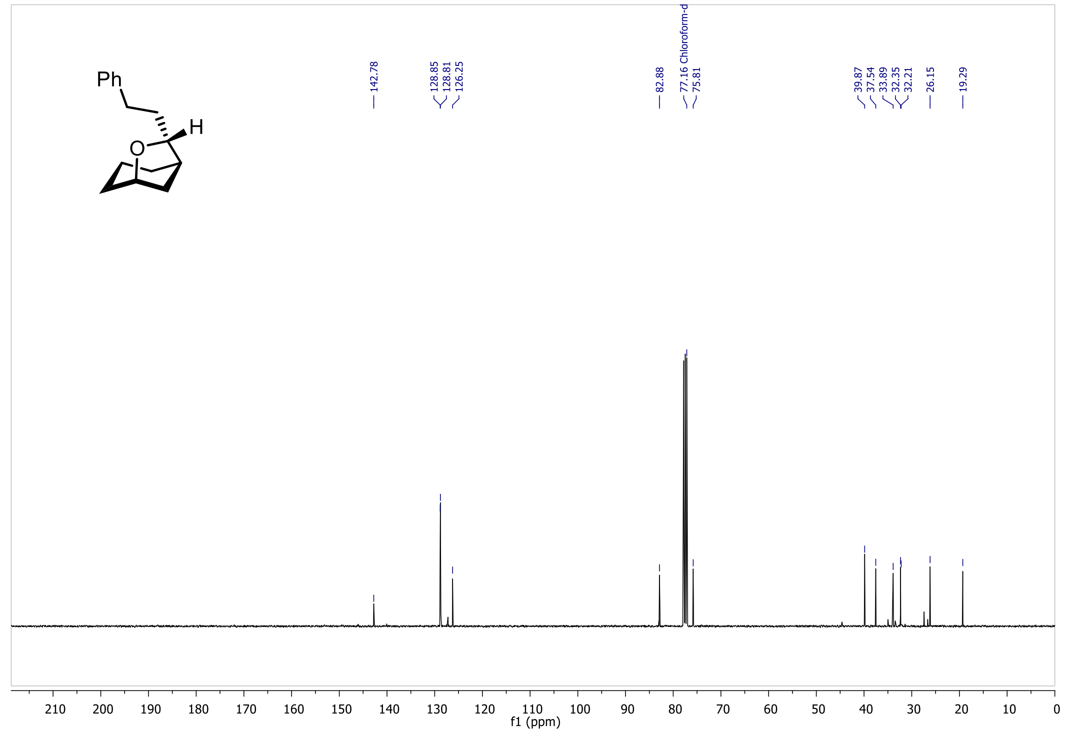

5s: 7-(Adamantan-1-yl)-6-oxabicyclo[3.2.1]octane

^{1}H NMR (400 MHz, CDCl$_3$)

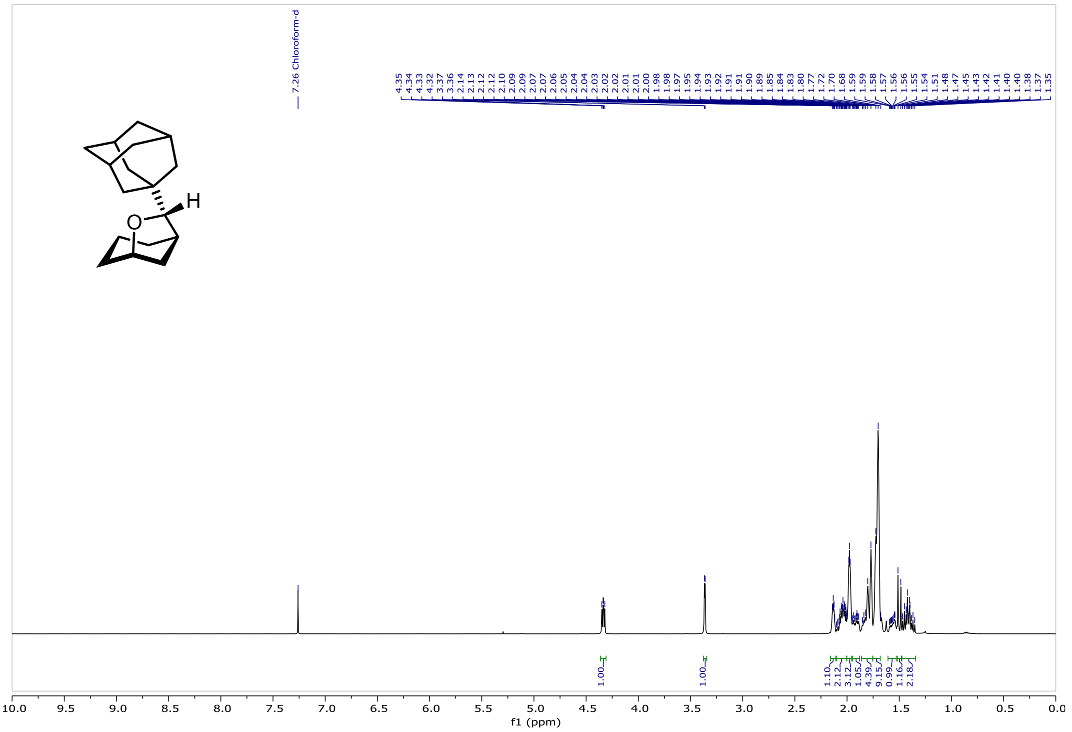

^{13}C NMR (101 MHz, CDCl$_3$)

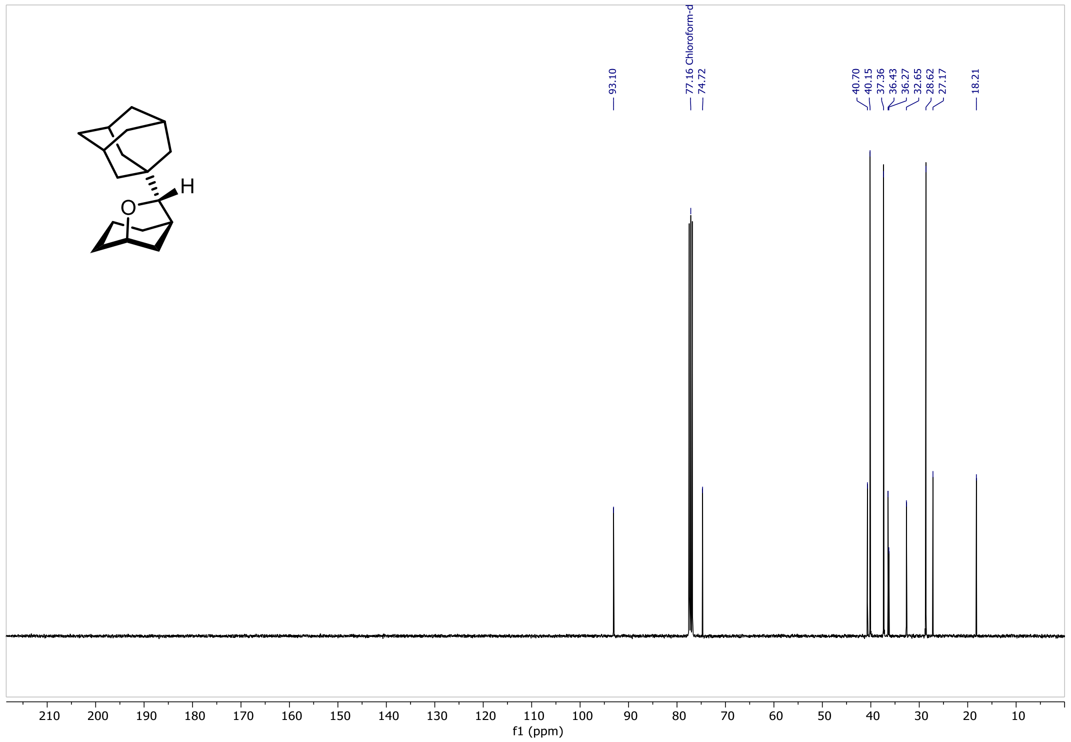

5t: 7-Isopropyl-6-oxabicyclo[3.2.1]octane

^{1}H NMR (400 MHz, $CDCl_3$)

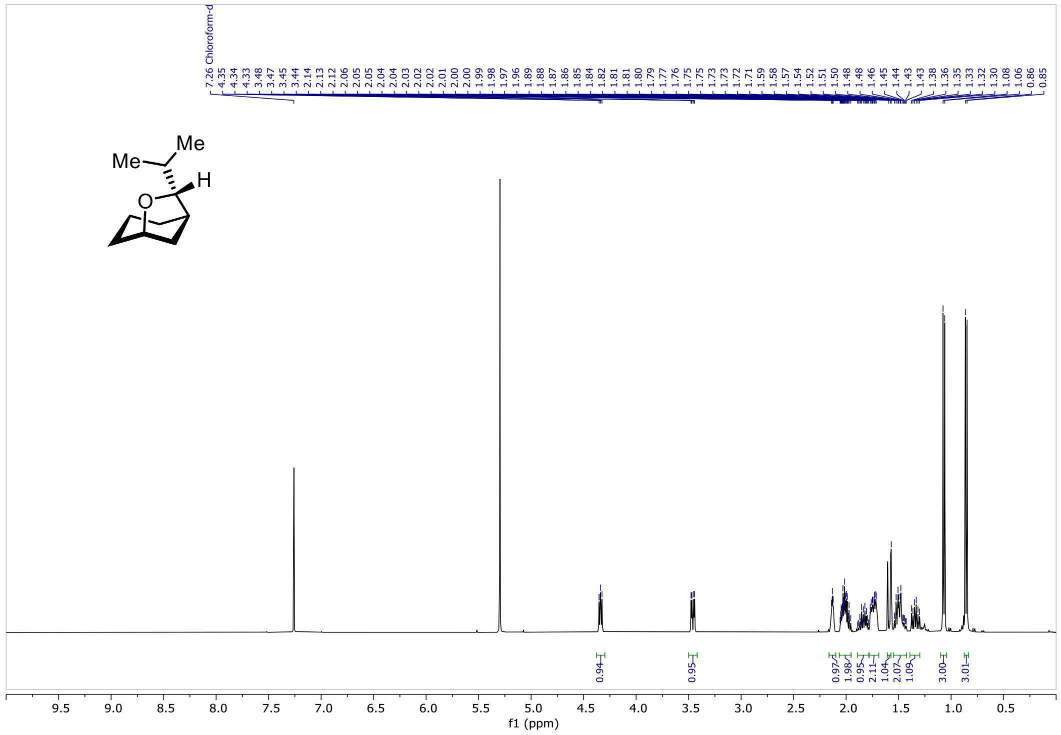

^{13}C NMR (151 MHz, $CDCl_3$)

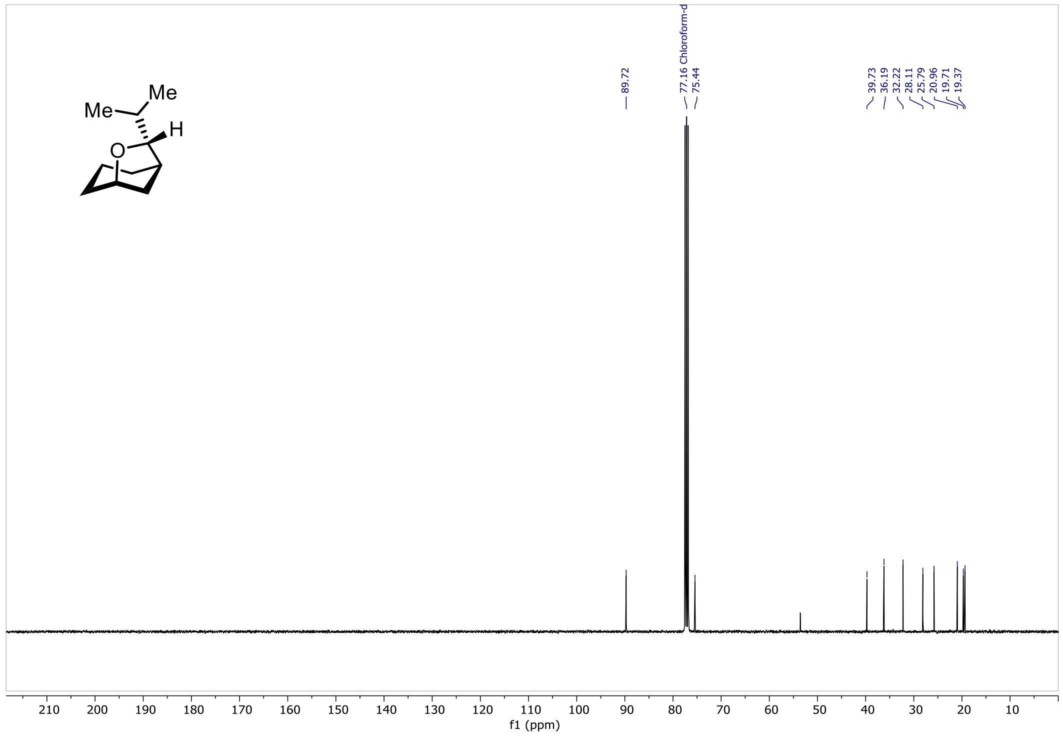

5u: 7-Cyclopropyl-6-oxabicyclo[3.2.1]octane

^{1}H NMR (400 MHz, $CDCl_3$)

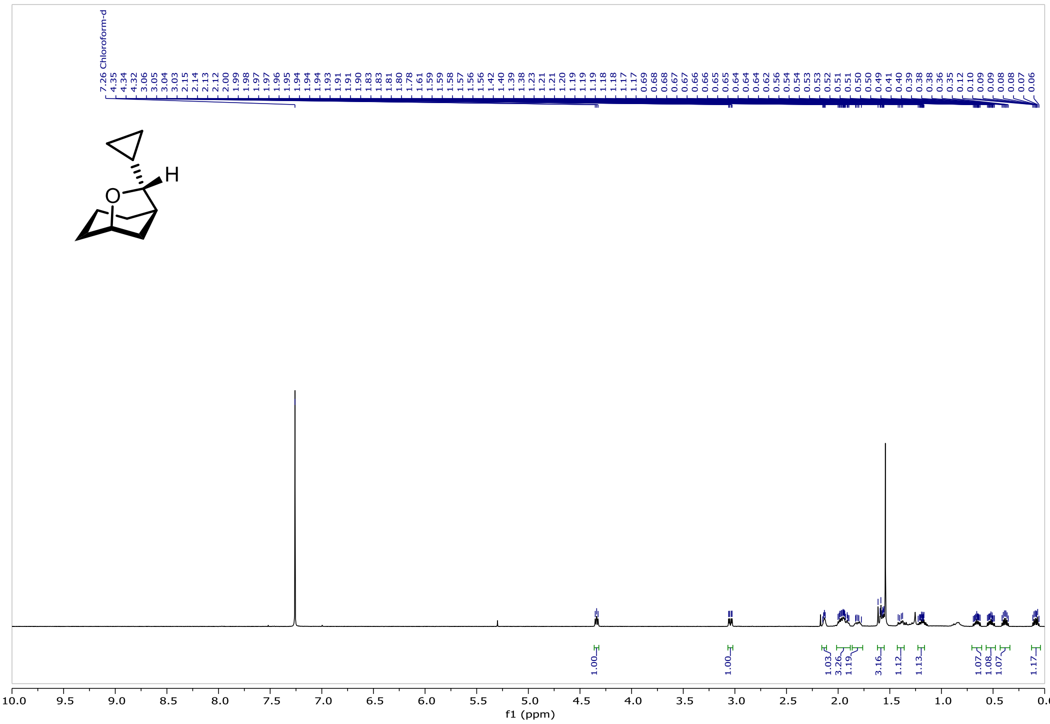

^{13}C NMR (101 MHz, $CDCl_3$)

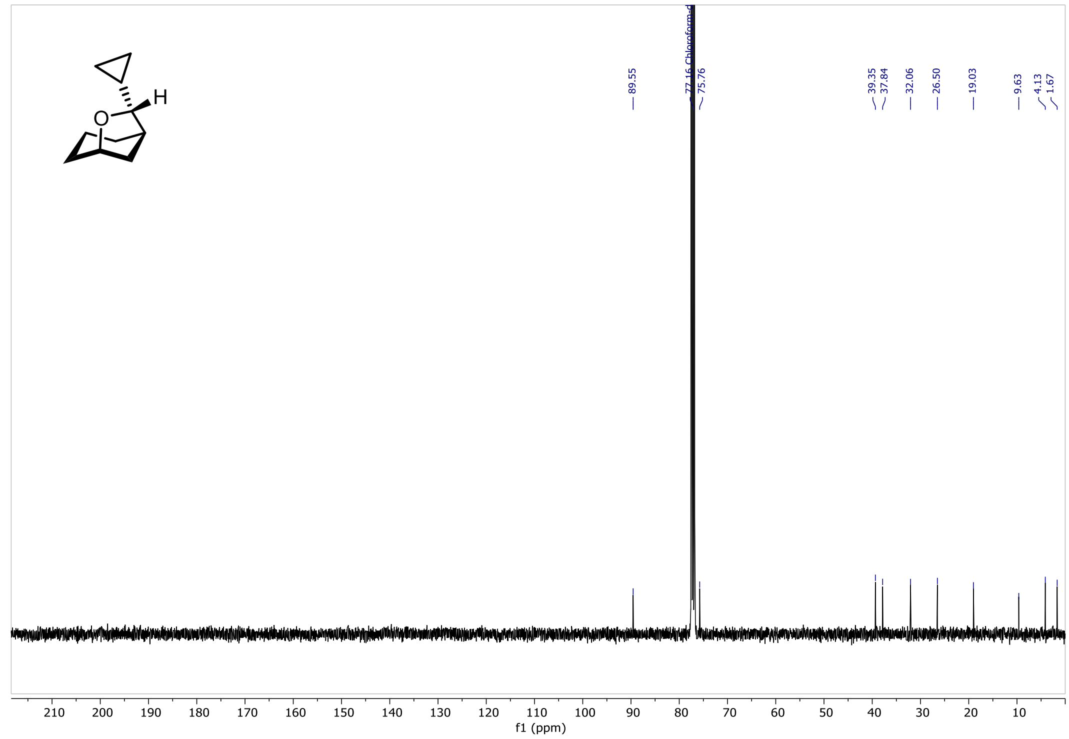

5v: 7-Cyclohexyl-6-oxabicyclo[3.2.1]octane

^{1}H NMR (400 MHz, CDCl$_3$)

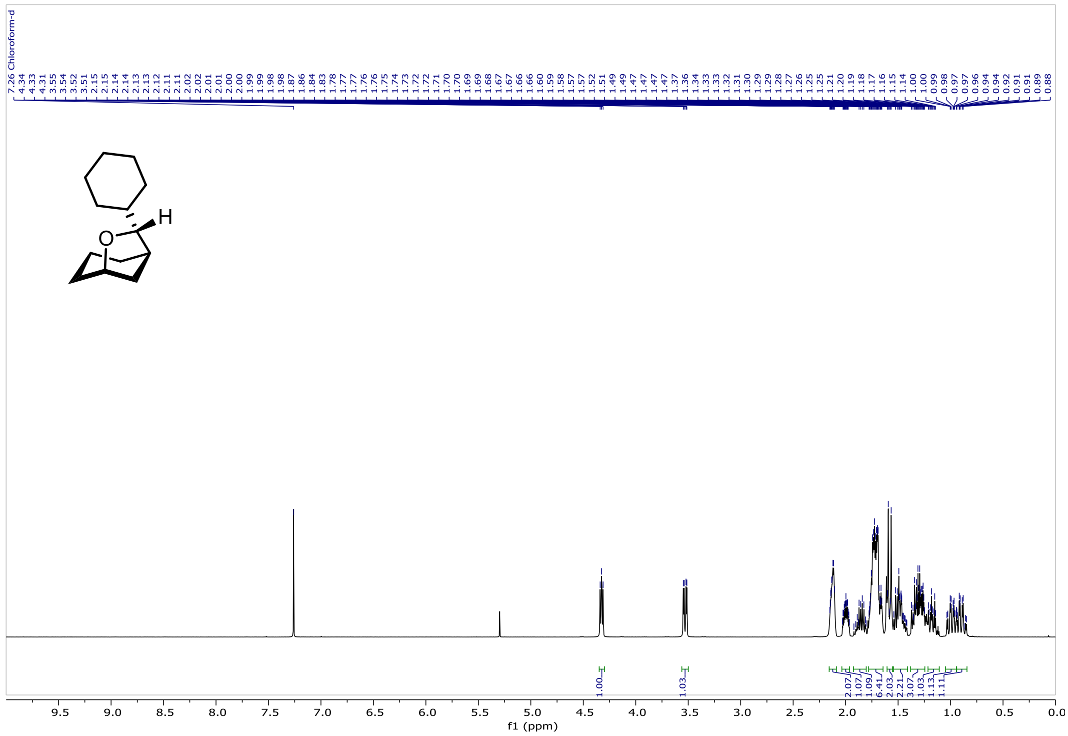

^{13}C NMR (151 MHz, CDCl$_3$)

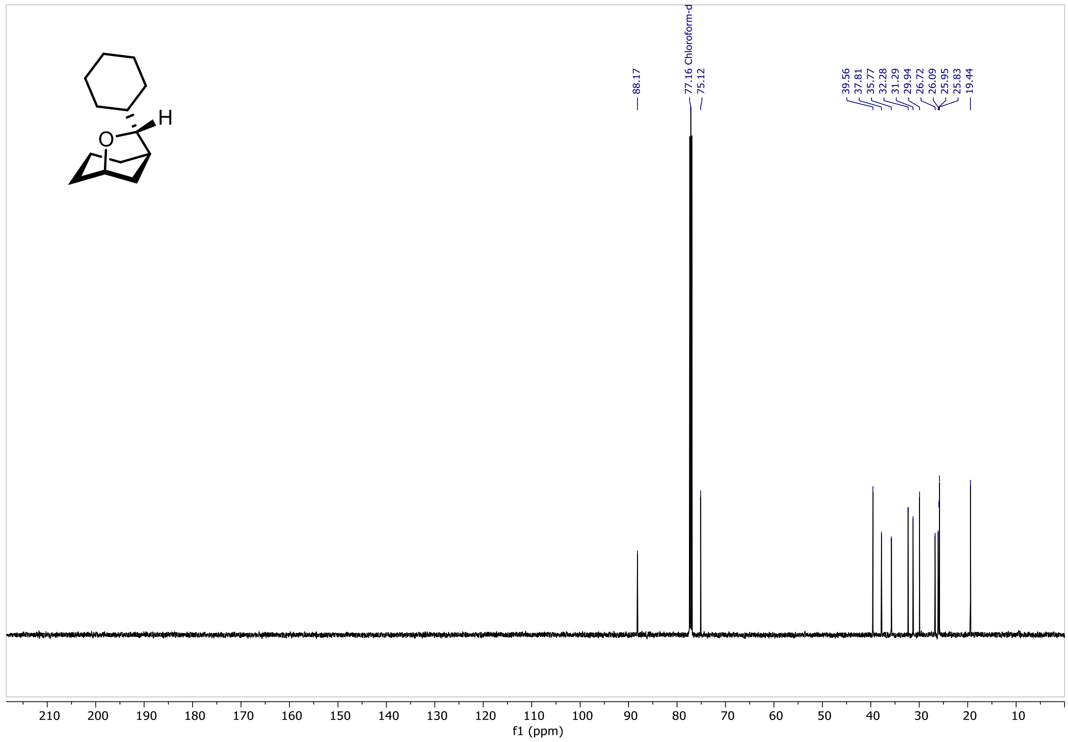

5w: 7-(3-Chloropropyl)-6-oxabicyclo[3.2.1]octane

^{1}H NMR (400 MHz, $CDCl_3$)

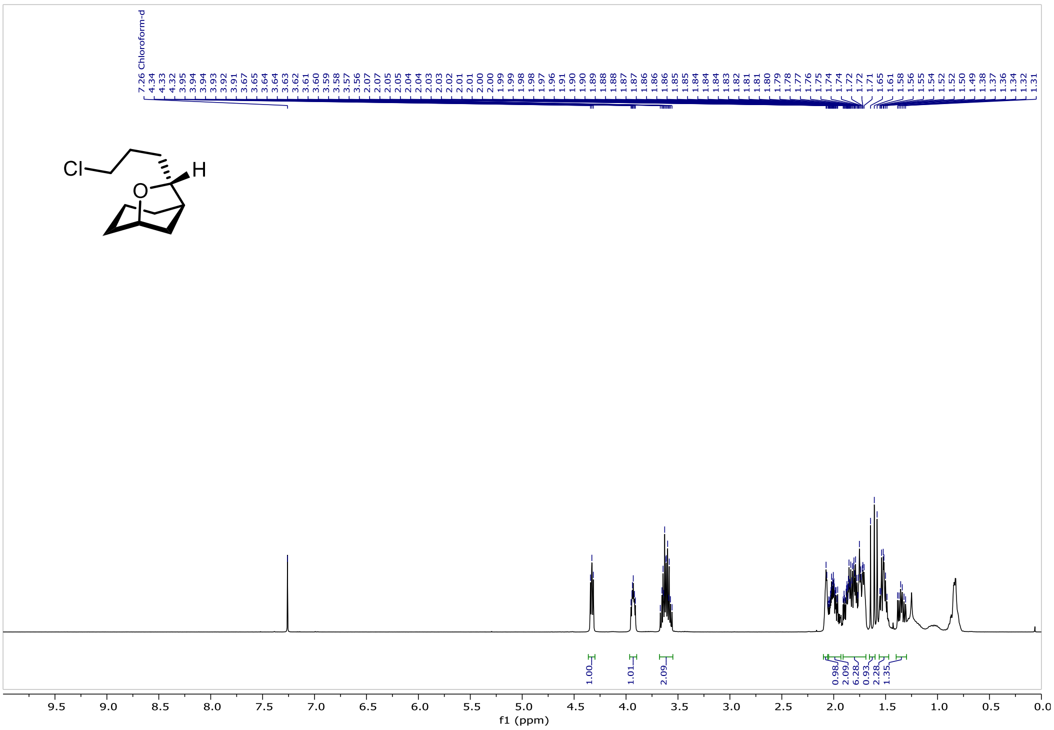

^{13}C NMR (101 MHz, $CDCl_3$)

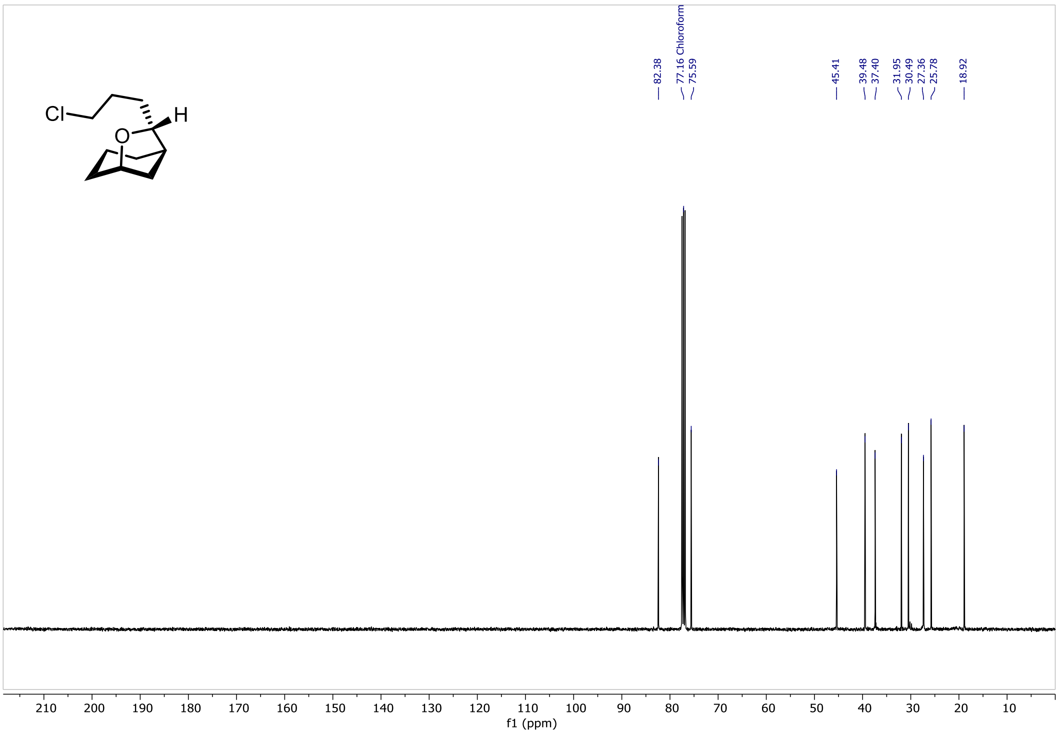

5x: 7-(3-Bromopropyl)-6-oxabicyclo[3.2.1]octane

^{1}H NMR (400 MHz, $CDCl_3$)

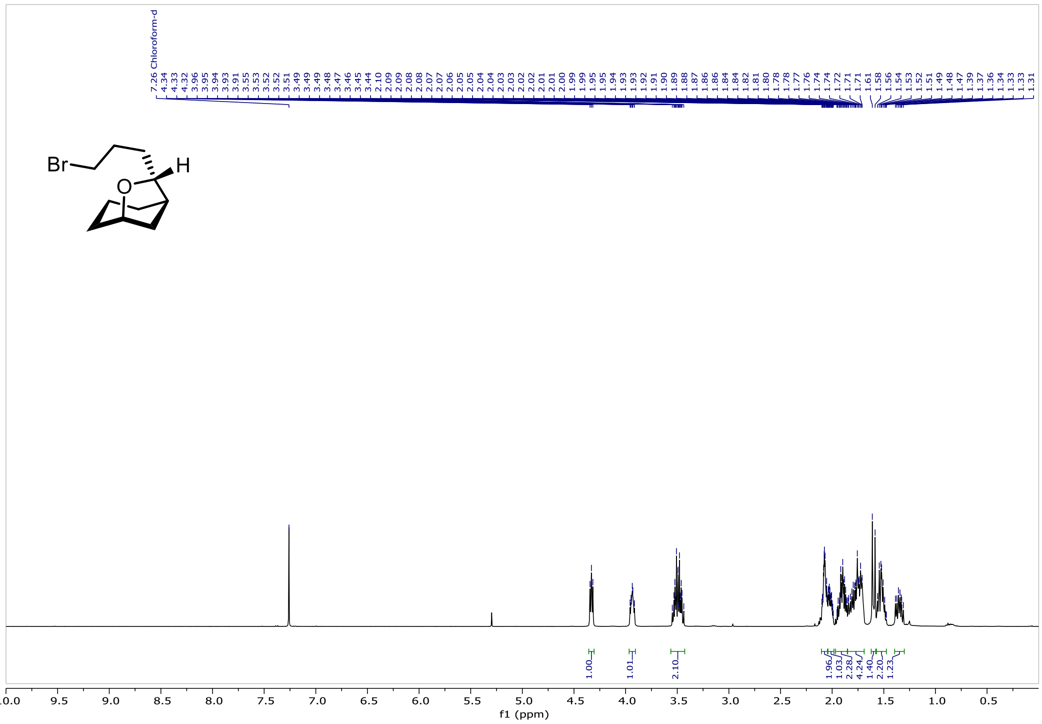

^{13}C NMR (101 MHz, $CDCl_3$)

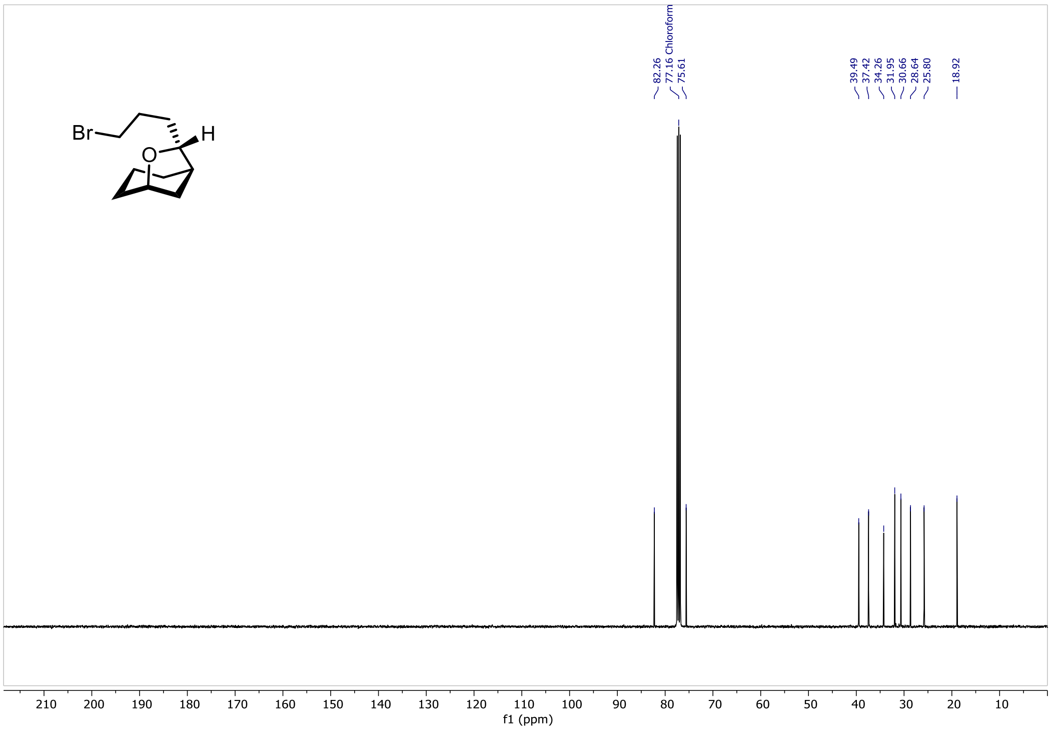

5y: 3-Phenyl-2-oxabicyclo[2.2.1]heptane

^{1}H NMR (400 MHz, $CDCl_3$)

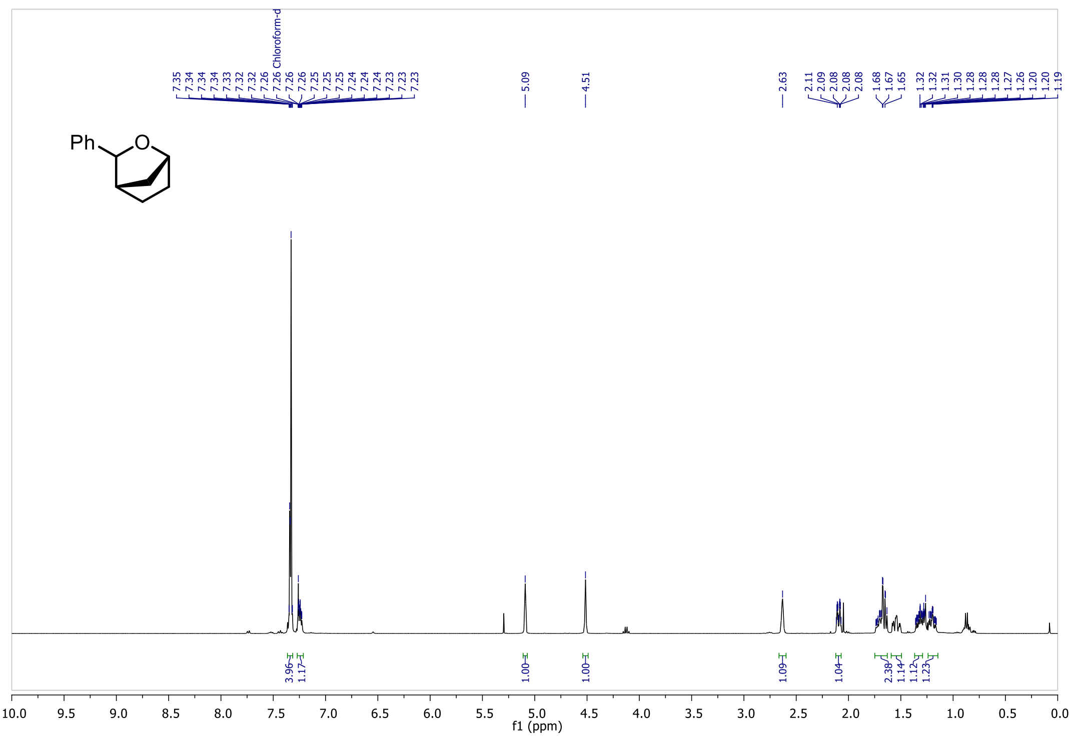

^{13}C NMR (101 MHz, $CDCl_3$)

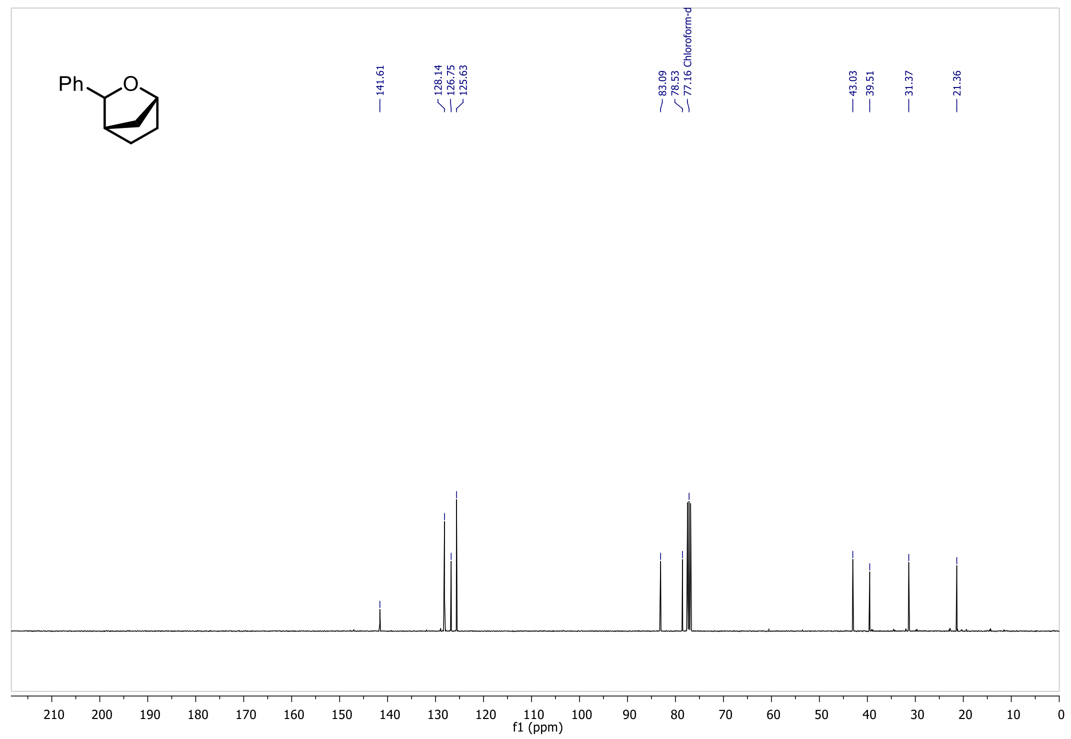

5z: 3-(2-Iodophenyl)-2-oxabicyclo[2.2.1]heptane

^{1}H NMR (400 MHz, $CDCl_3$)

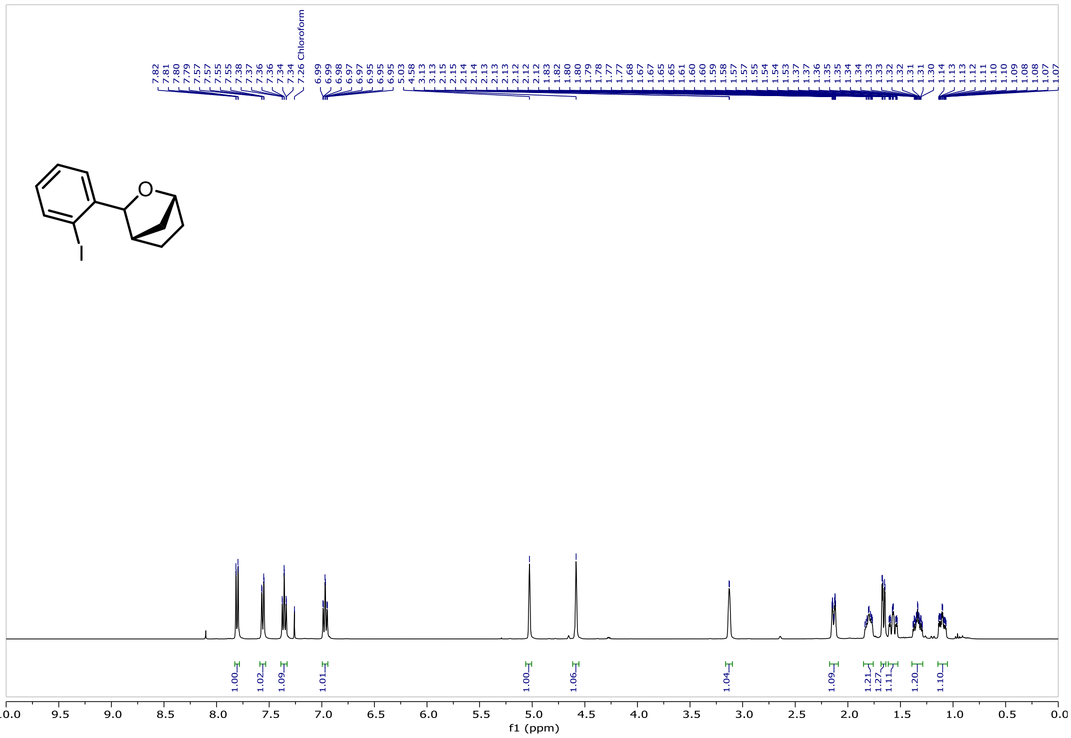

^{13}C NMR (101 MHz, $CDCl_3$)

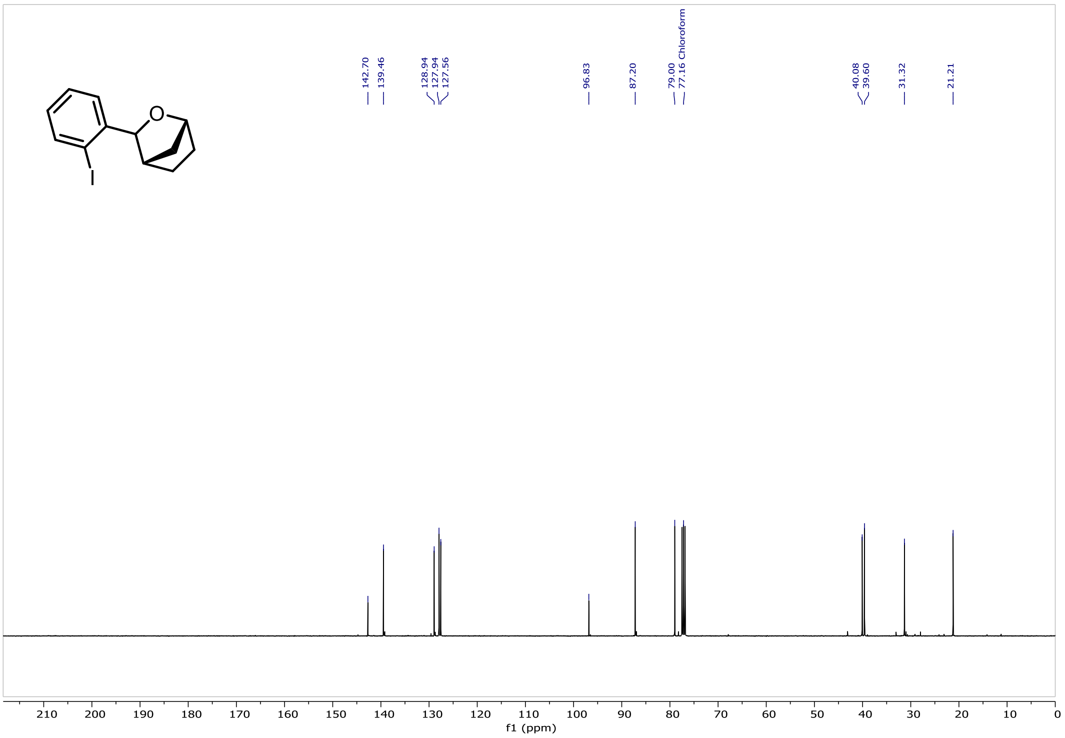

5aa:3-Undecyl-2-oxabicyclo[2.2.1]heptane

^{1}H NMR (400 MHz, $CDCl_3$)

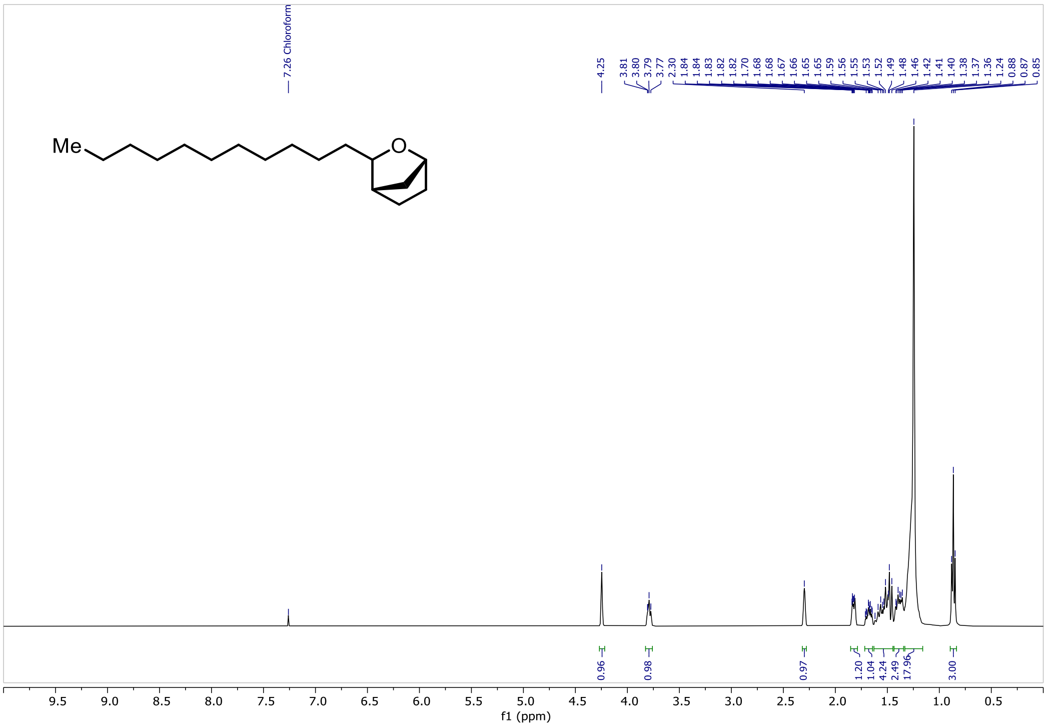

^{13}C NMR (101 MHz, $CDCl_3$)

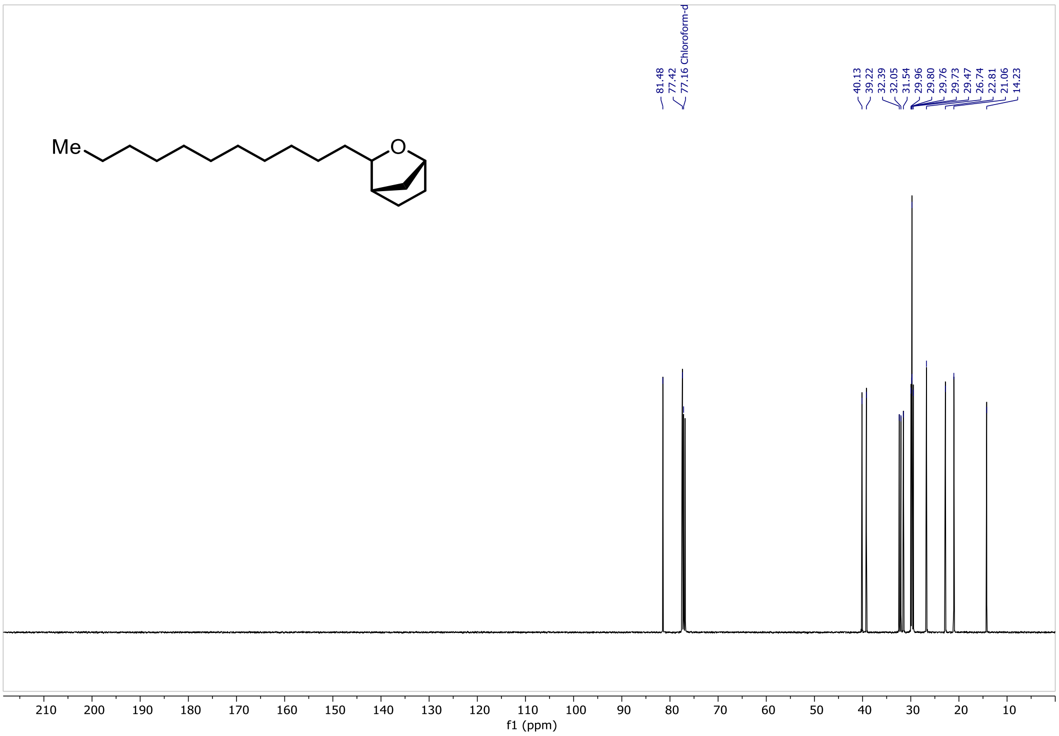

5ab: 13-Phenyl-12-oxabicyclo[9.2.1]tetradecane

^{1}H NMR (400 MHz, $CDCl_3$)

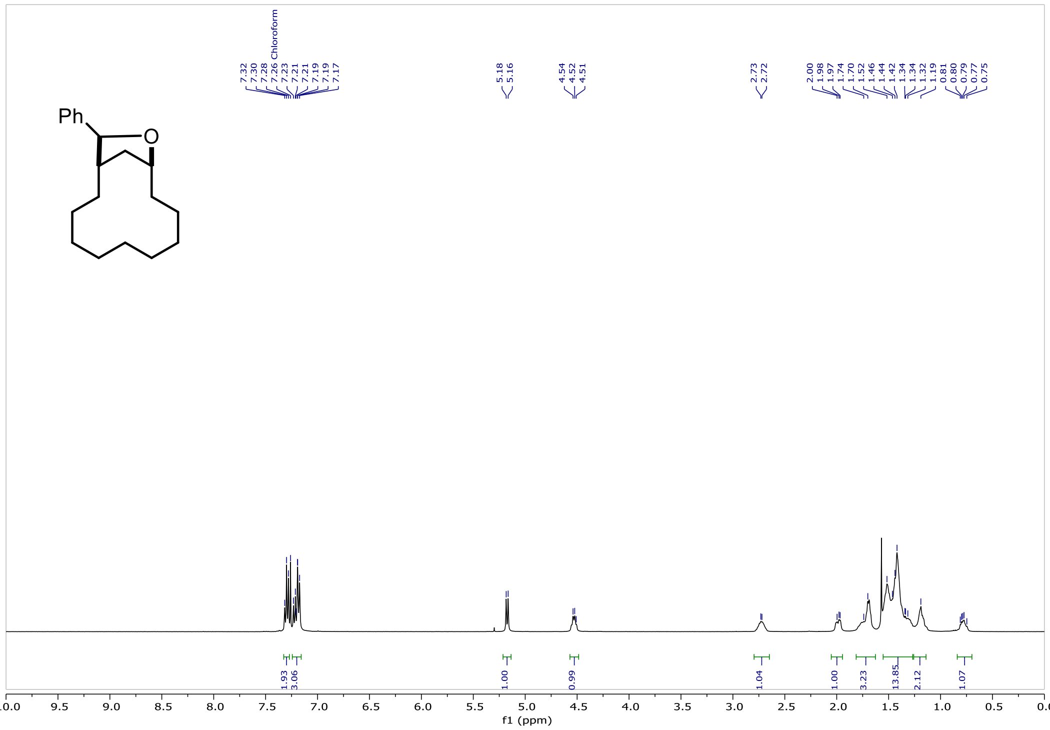

^{13}C NMR (101 MHz, $CDCl_3$)

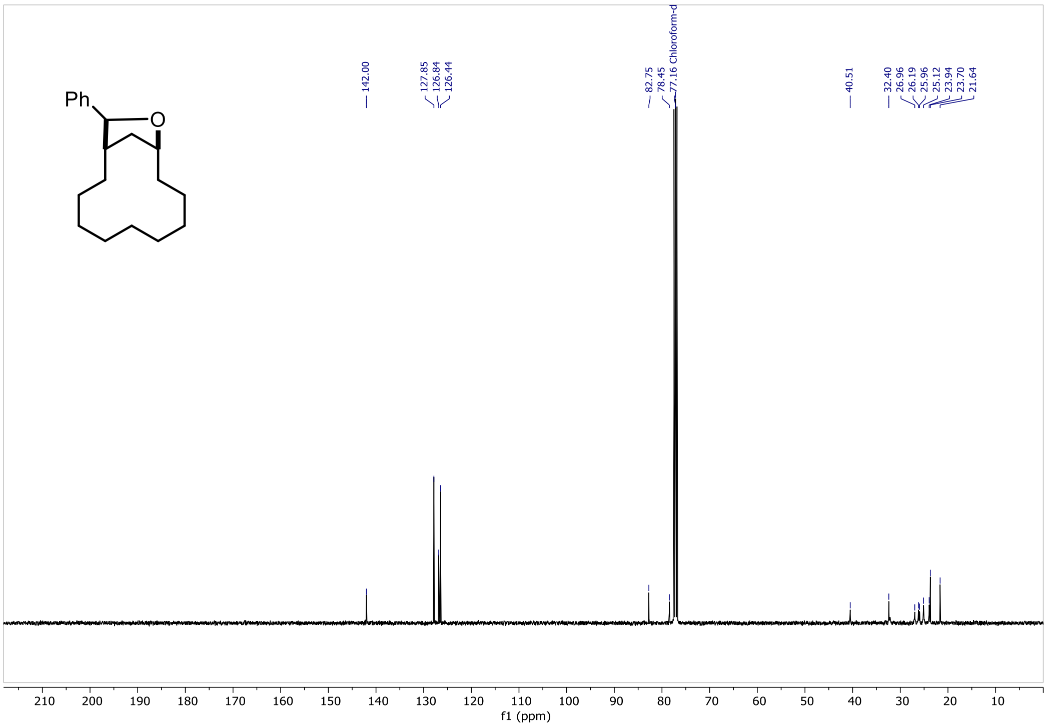

5ac: 2-phenyldecahydro-1,4:6,9-dimethanobenzo[d]oxepine

^{1}H NMR (400 MHz, $CDCl_3$)

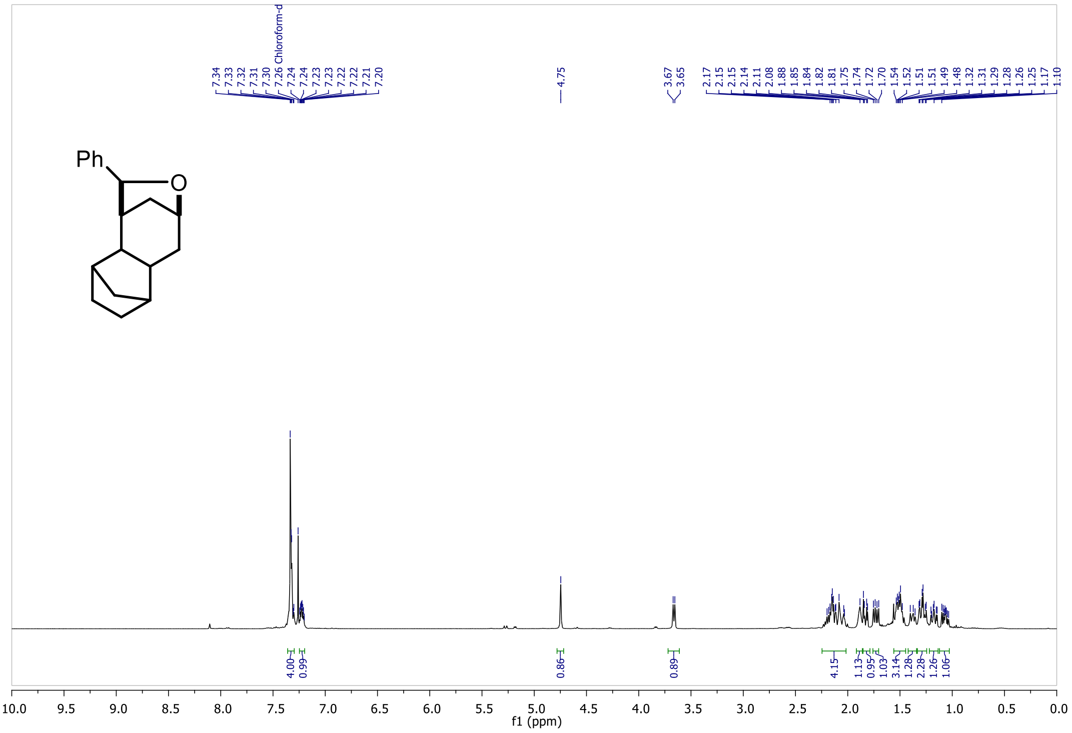

^{13}C NMR (101 MHz, $CDCl_3$)

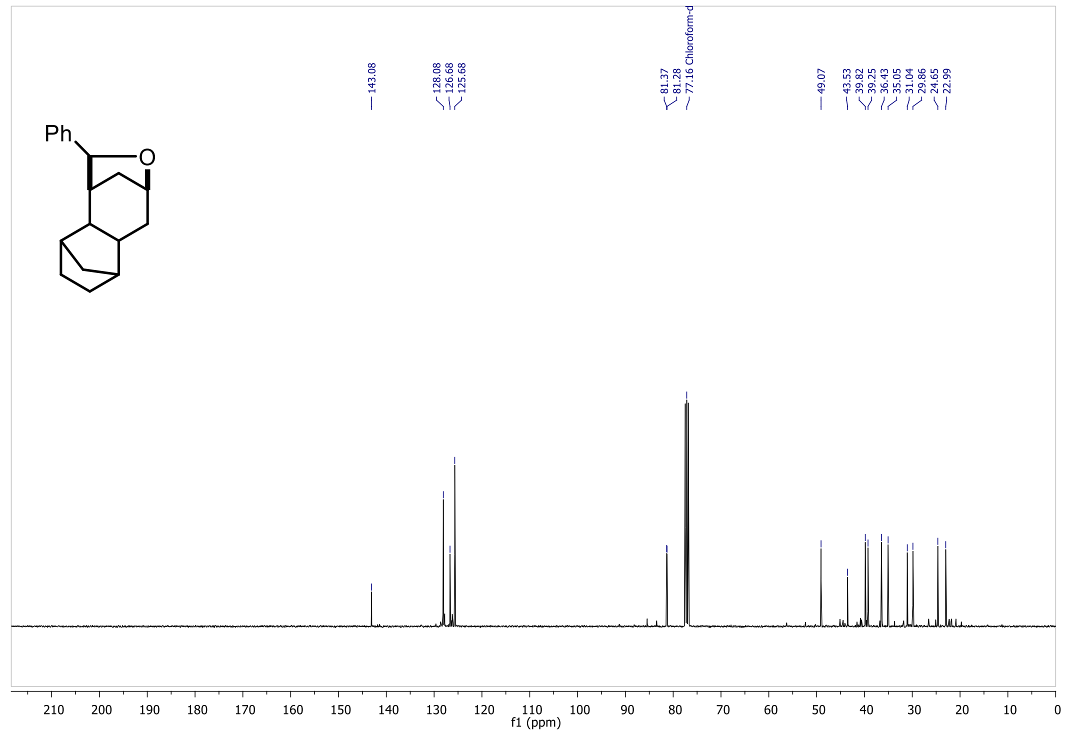

7a: 2-Phenyl-1-oxaspiro[4.5]decane

^{1}H NMR (400 MHz, $CDCl_3$)

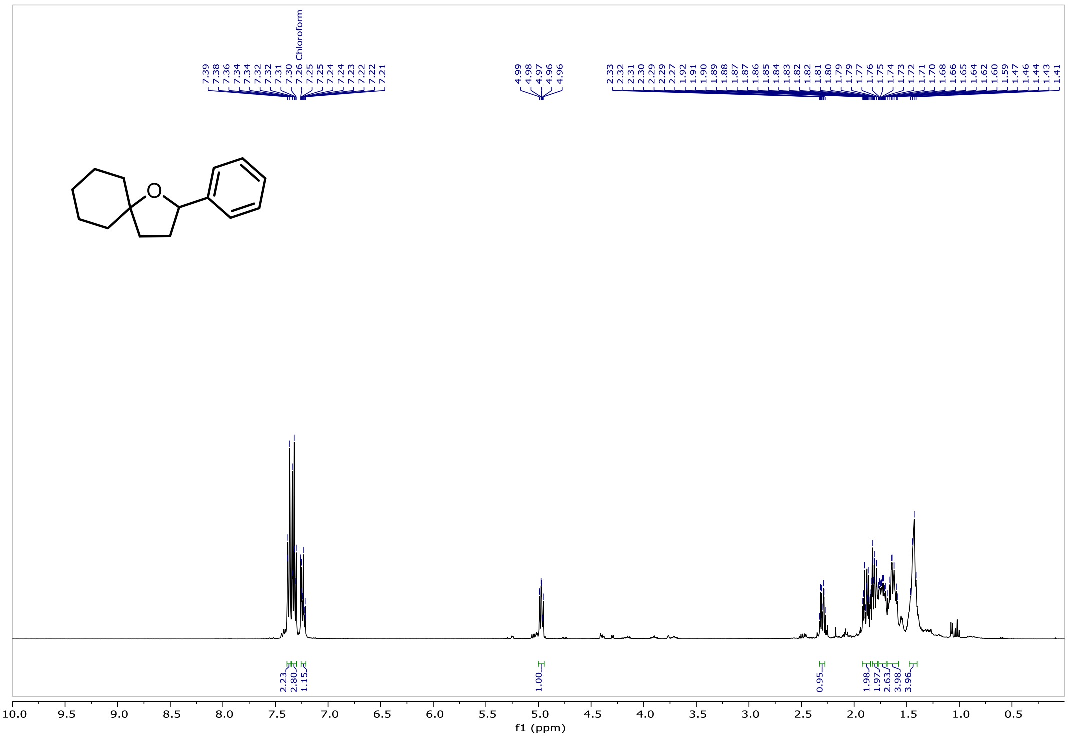

^{13}C NMR (101 MHz, $CDCl_3$)

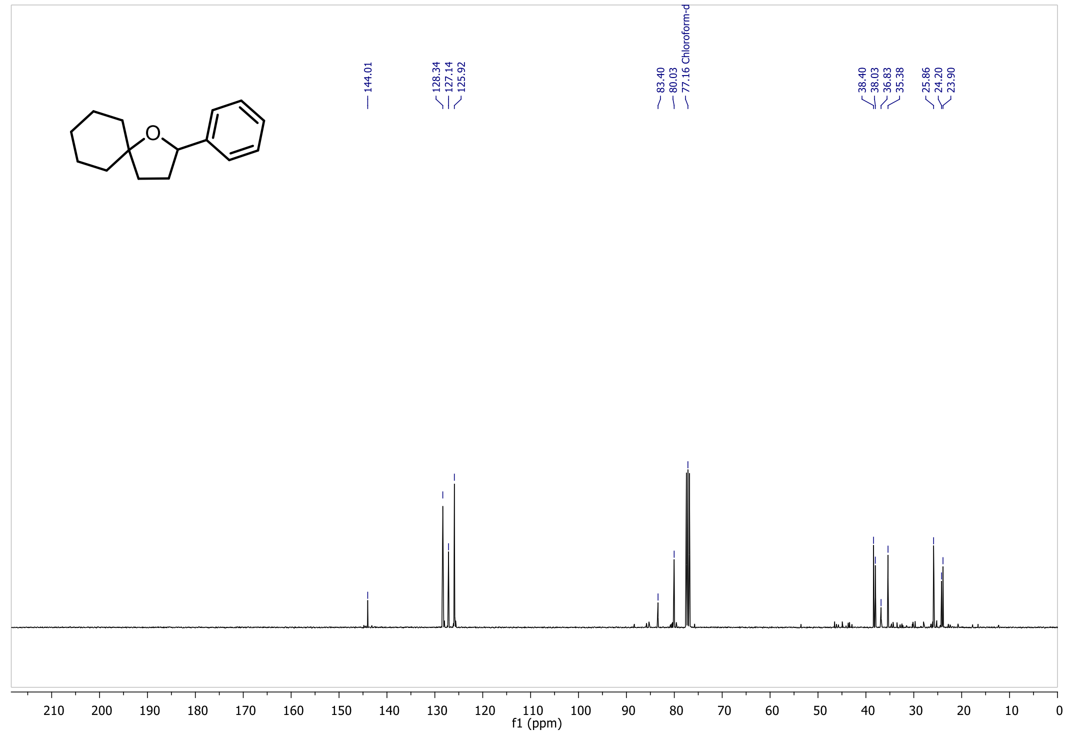

7b: 2-(2-Iodophenyl)-1-oxaspiro[4.5]decane

^{1}H NMR (400 MHz, $CDCl_3$)

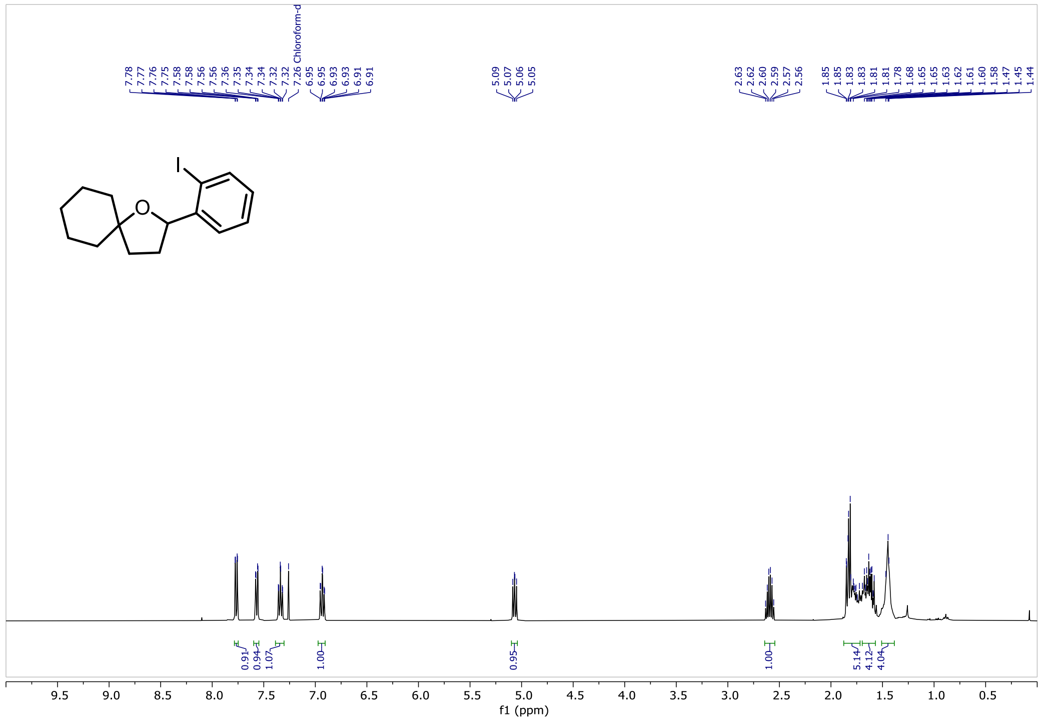

^{13}C NMR (101 MHz, $CDCl_3$)

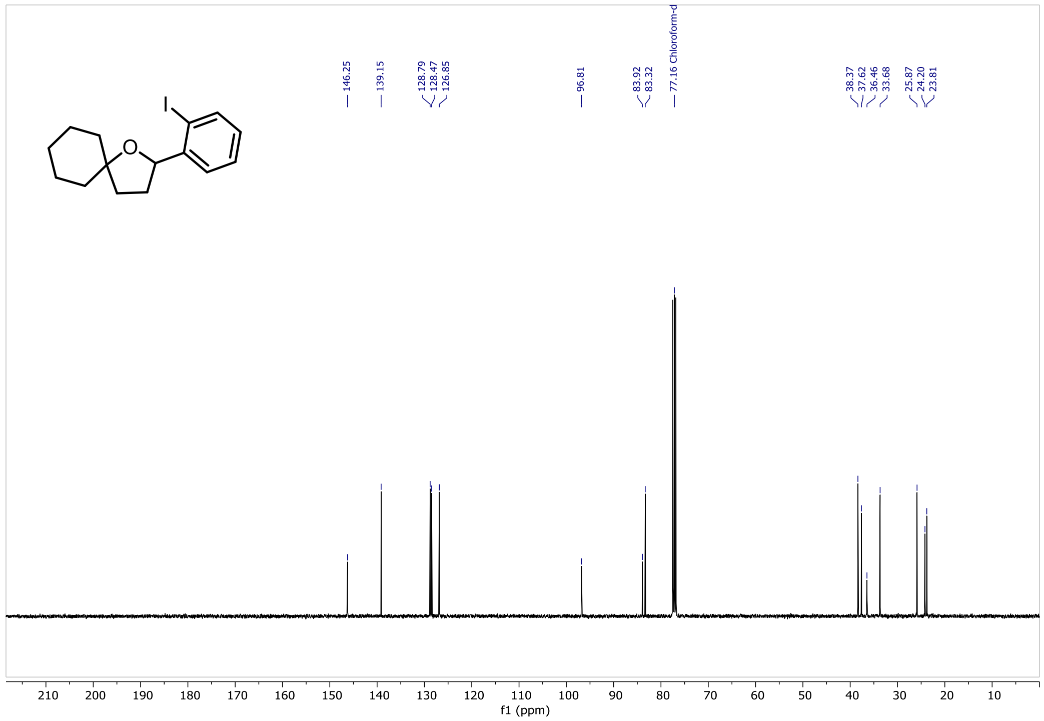

7c: 2-(4-Methoxyphenyl)-1-oxaspiro[4.5]decane

^{1}H NMR (400 MHz, $CDCl_3$)

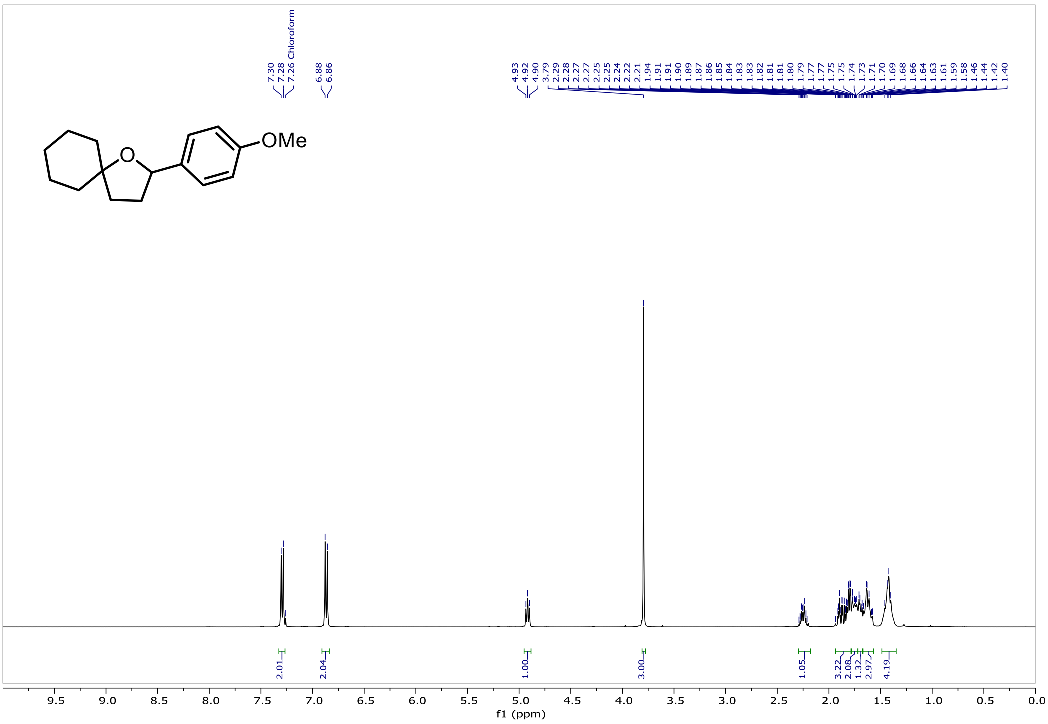

^{13}C NMR (101 MHz, $CDCl_3$)

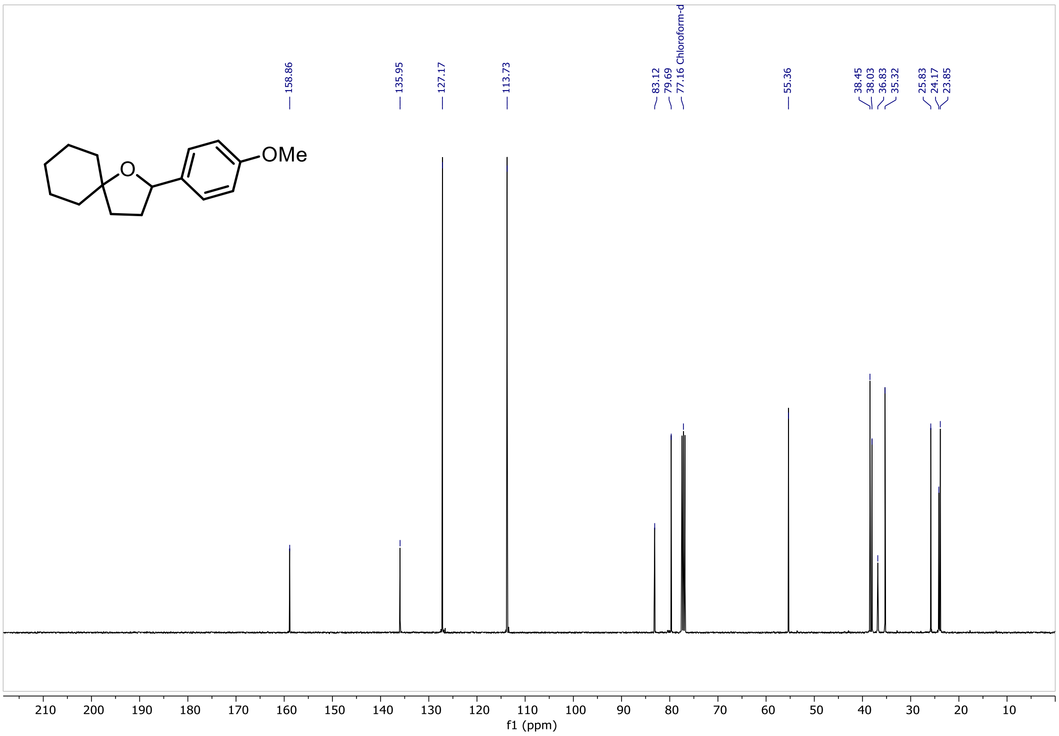

7d: 2-(Adamantan-1-yl)-1-oxaspiro[4.5]decane

^{1}H NMR (400 MHz, $CDCl_3$).

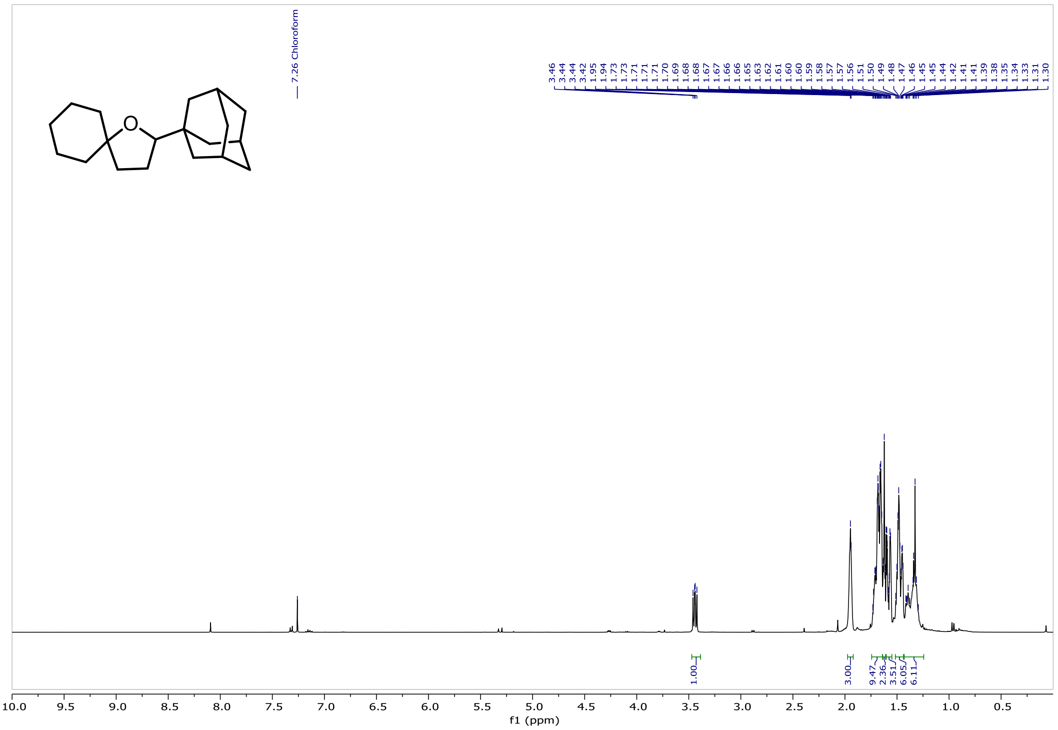

^{13}C NMR (101 MHz, $CDCl_3$).

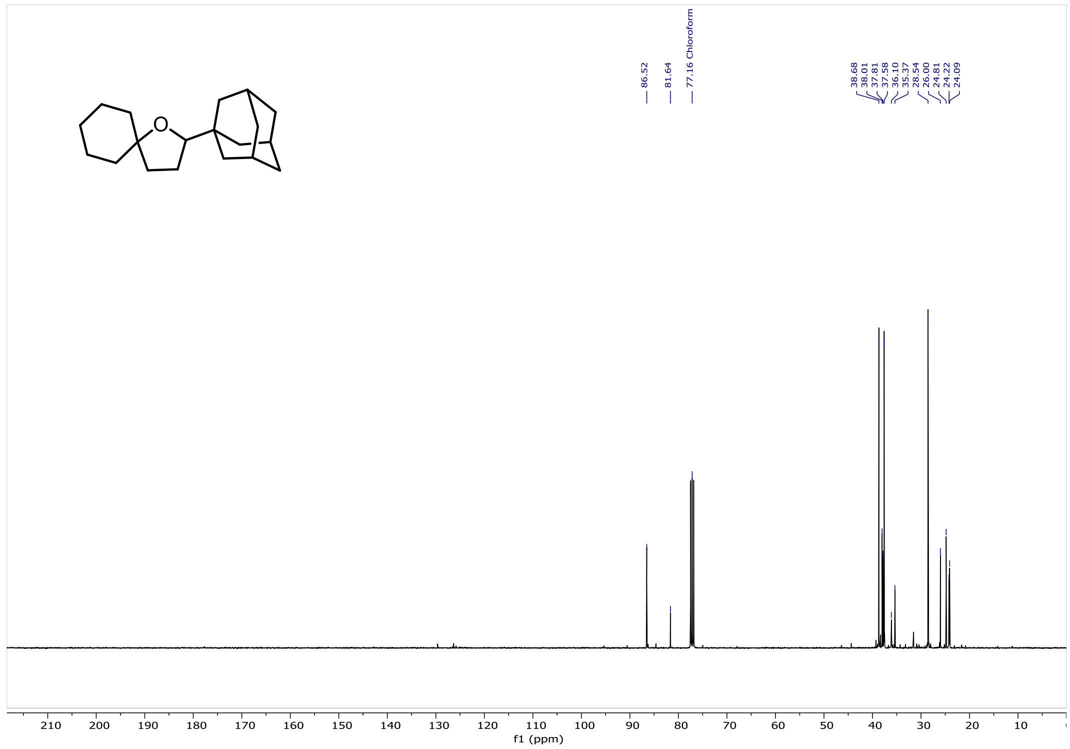

7e: 2-Cyclohexyl-1-oxaspiro[4.5]decane

^{1}H NMR (400 MHz, $CDCl_3$)

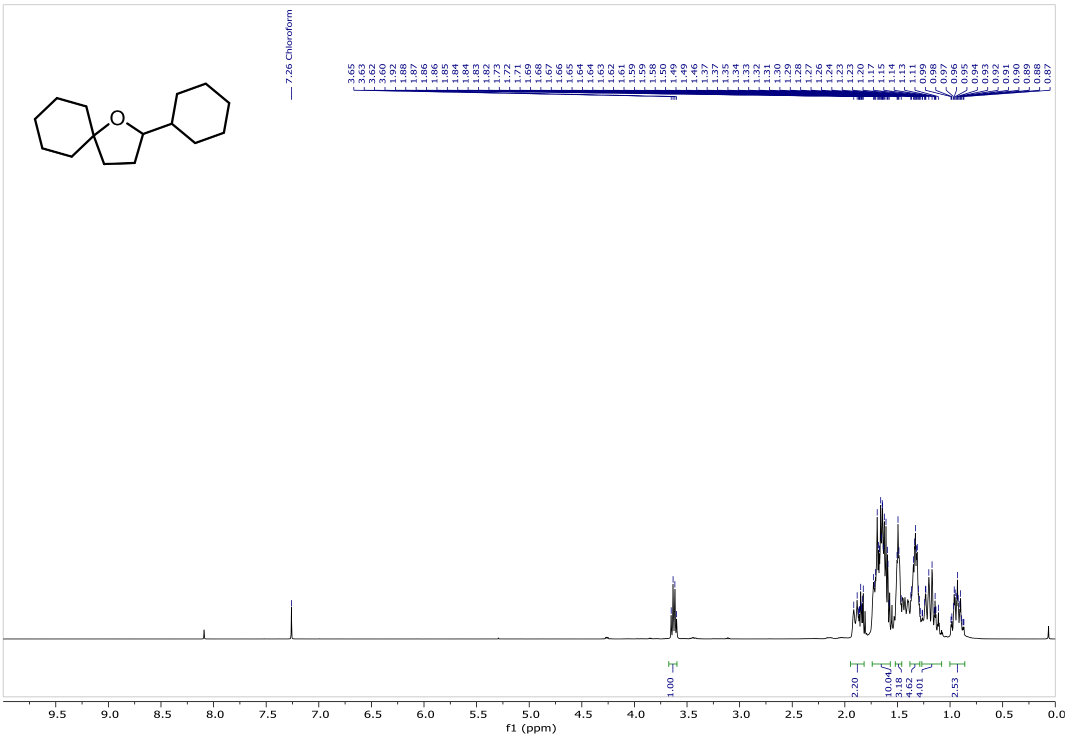

^{13}C NMR (101 MHz, $CDCl_3$)

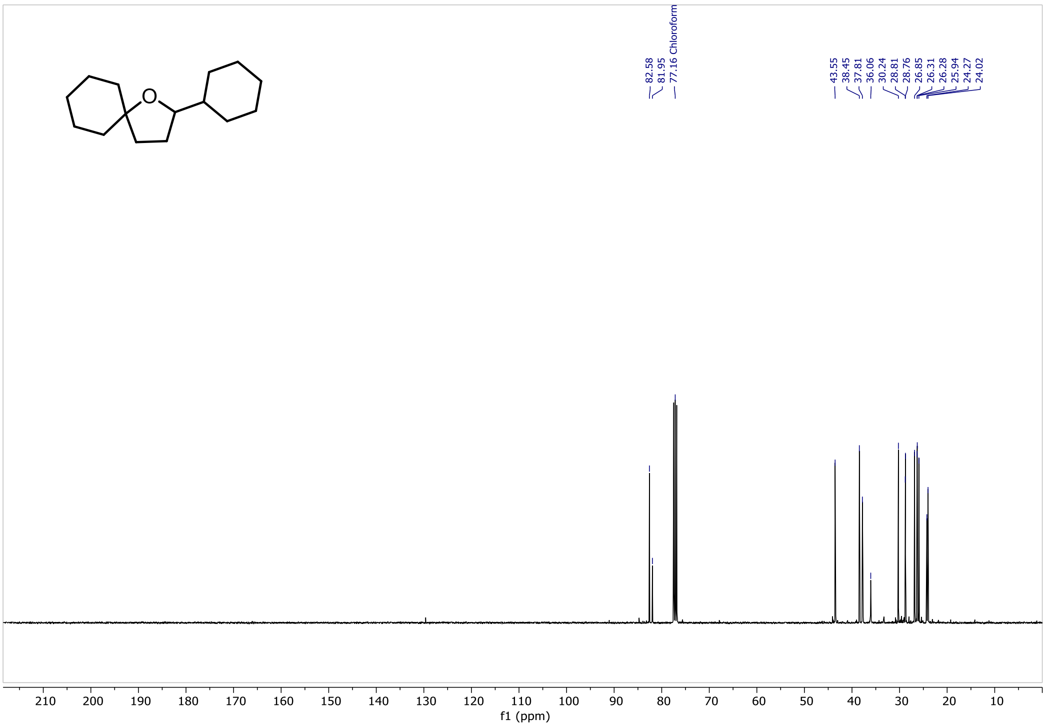

7f: 2-Undecyl-1-oxaspiro[4.5]decane

^{1}H NMR (400 MHz, $CDCl_3$)

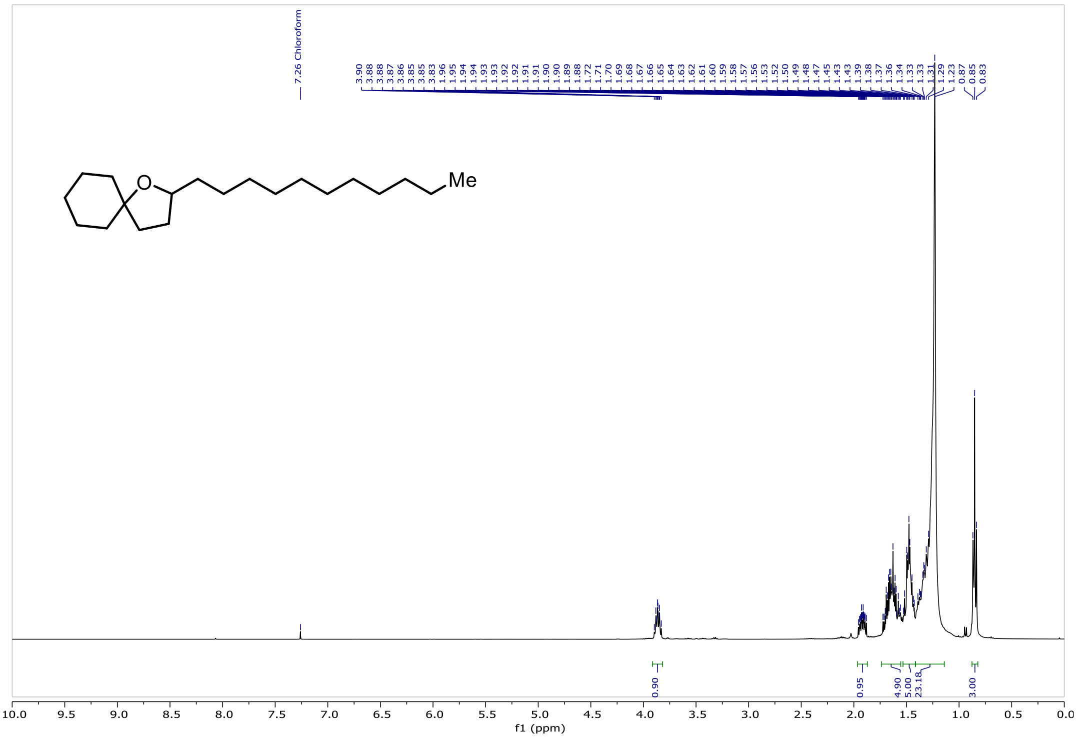

^{13}C NMR (101 MHz, $CDCl_3$)

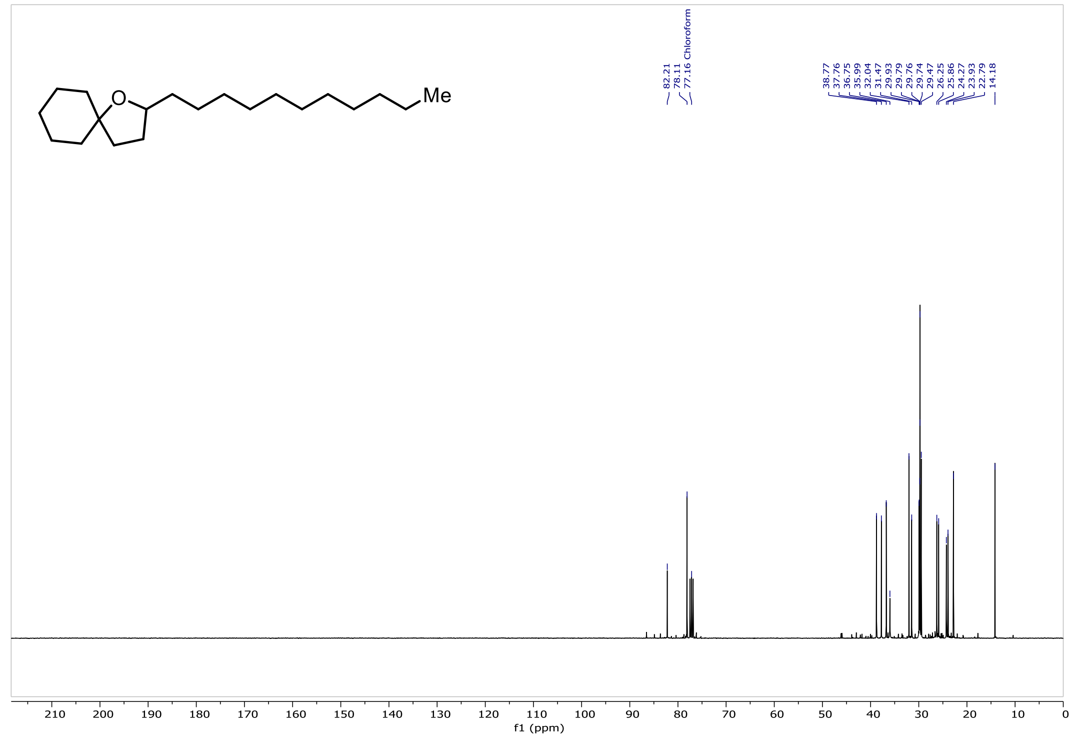

7g: 2-(5-Bromopentyl)-1-oxaspiro[4.5]decane

^{1}H NMR (400 MHz, $CDCl_3$)

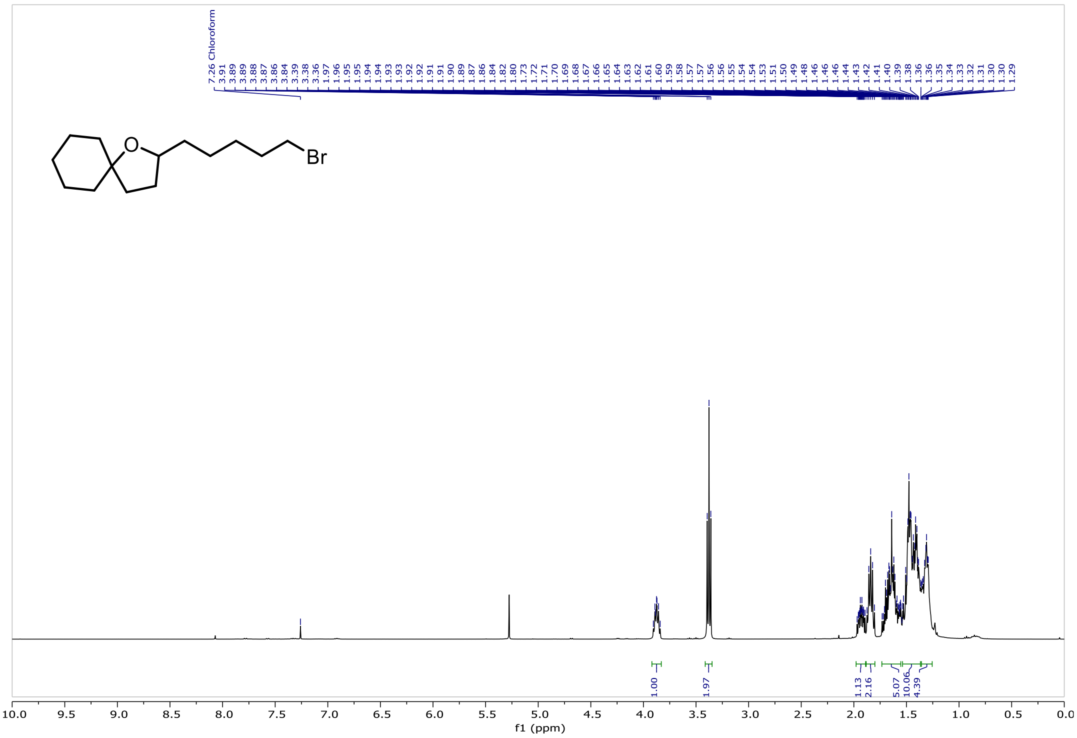

^{13}C NMR (101 MHz, $CDCl_3$)

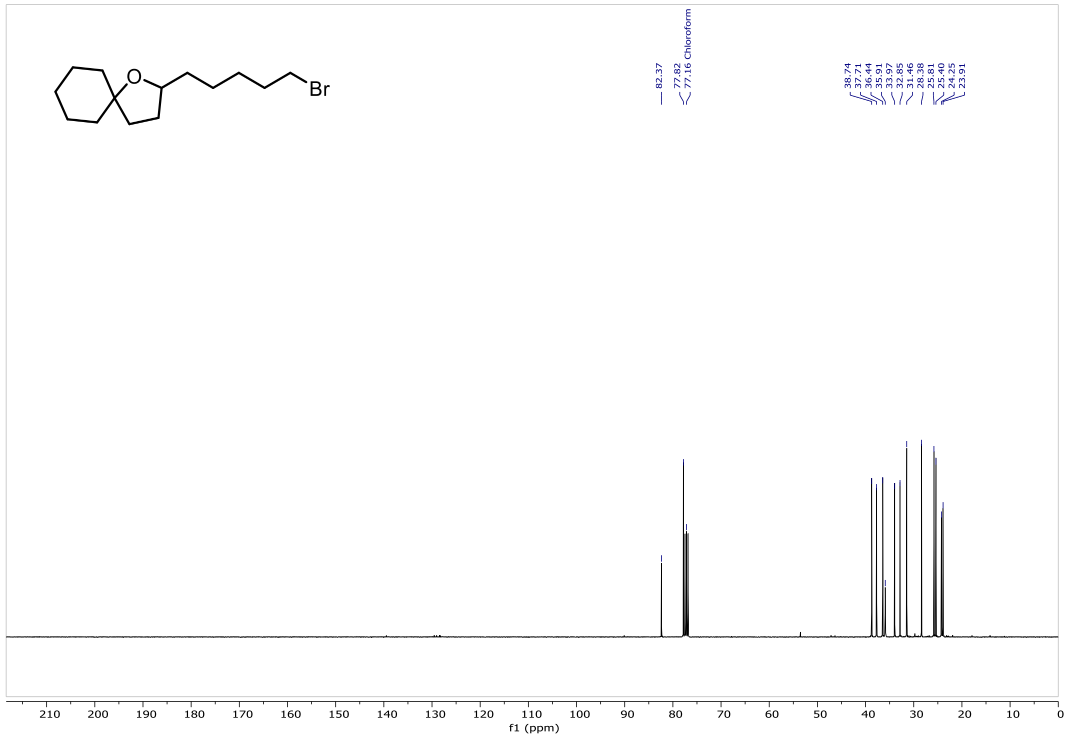

7h: 2-Phenethyl-1-oxaspiro[4.5]decane

^{1}H NMR (400 MHz, $CDCl_3$)

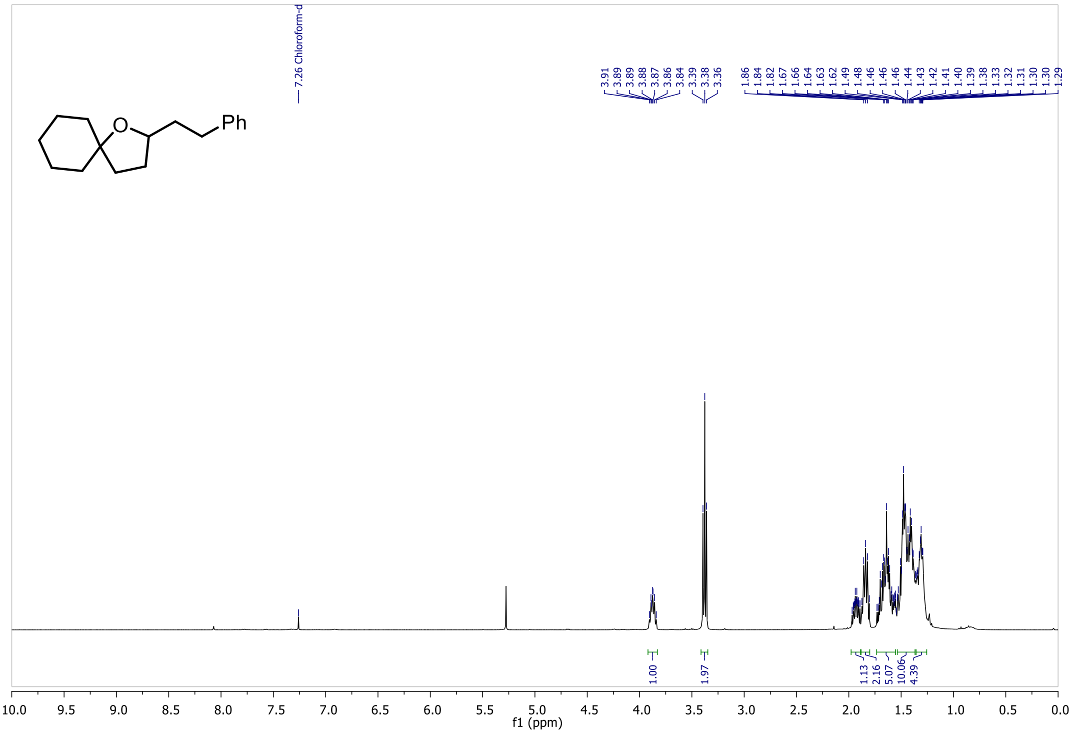

^{13}C NMR (101 MHz, $CDCl_3$)

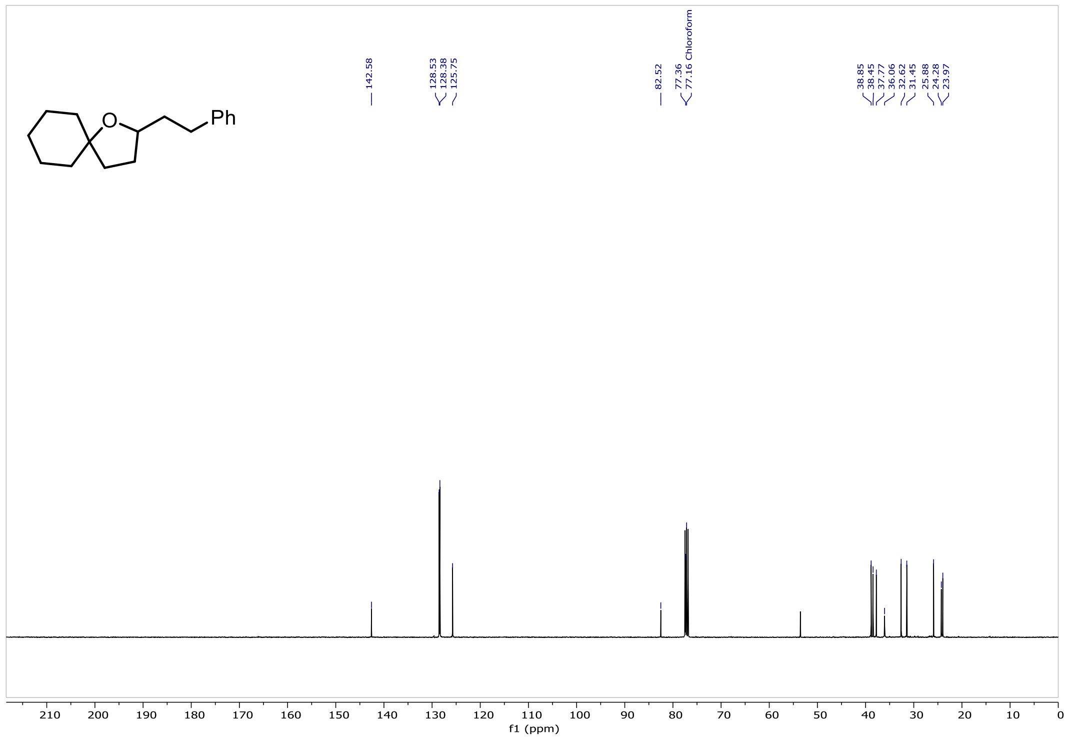

9. References

[24] D. A. Mundal, K. E. Lutz, R. J. Thomson, *J. Am. Chem. Soc.* **2012**, *134*, 5782–5785.

[43] A. F. G. Maier, S. Tussing, T. Schneider, U. Flörke, Z.-W. Qu, S. Grimme, J. Paradies, *Angew. Chem. Int. Ed.* **2016**, 55, 12219–12223.

[44] S. Qiu; H. Guo; P. Xu, *Org. Lett.* **2024**, 26, 31, 6730–6735.

[45] D. P. Hari, G. Pisella, M. D. Wodrich, A. V. Tsymbal, F. F. Tirani, R. Scopelliti, J. Waser, *Angew. Chem. Int. Ed.* **2021**, *60*, 5475–5481.

[46] D. P. Hari, J. Waser, *J. Am. Chem. Soc.* **2017**, 139, 8420−8423.

[47] J. Zhang, Y. Li, R. Y. Xu, Y. Y. Chen, *Angew. Chem. Int. Ed.* **2017**, *56*, 12619−12623.
